# Supplementary figures and images for: Microglia replacement by ER-Hoxb8 conditionally immortalized macrophages provides insight into Aicardi–Goutières syndrome neuropathology (part 2 of 2)
Source: eLife. 2026 Jan 27;14:RP102900. doi: 10.7554/eLife.102900 (PMC12844908; doi:10.7554/eLife.102900)

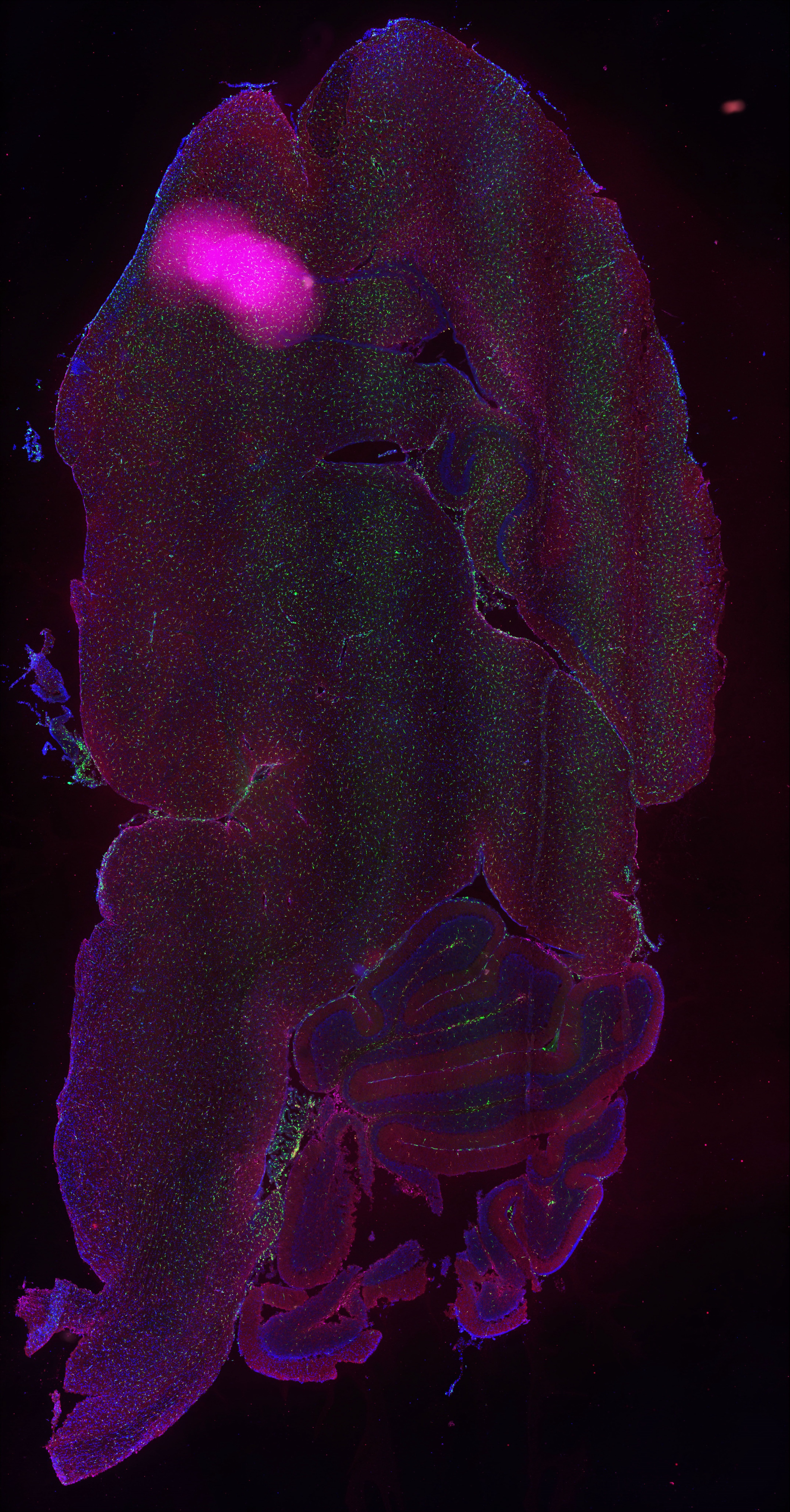

Supplement: Supplementary file 2. [file elife-102900-supp2.zip › Supplementary File 2/Raw Stitches/745.3 Stitch All.jpeg]

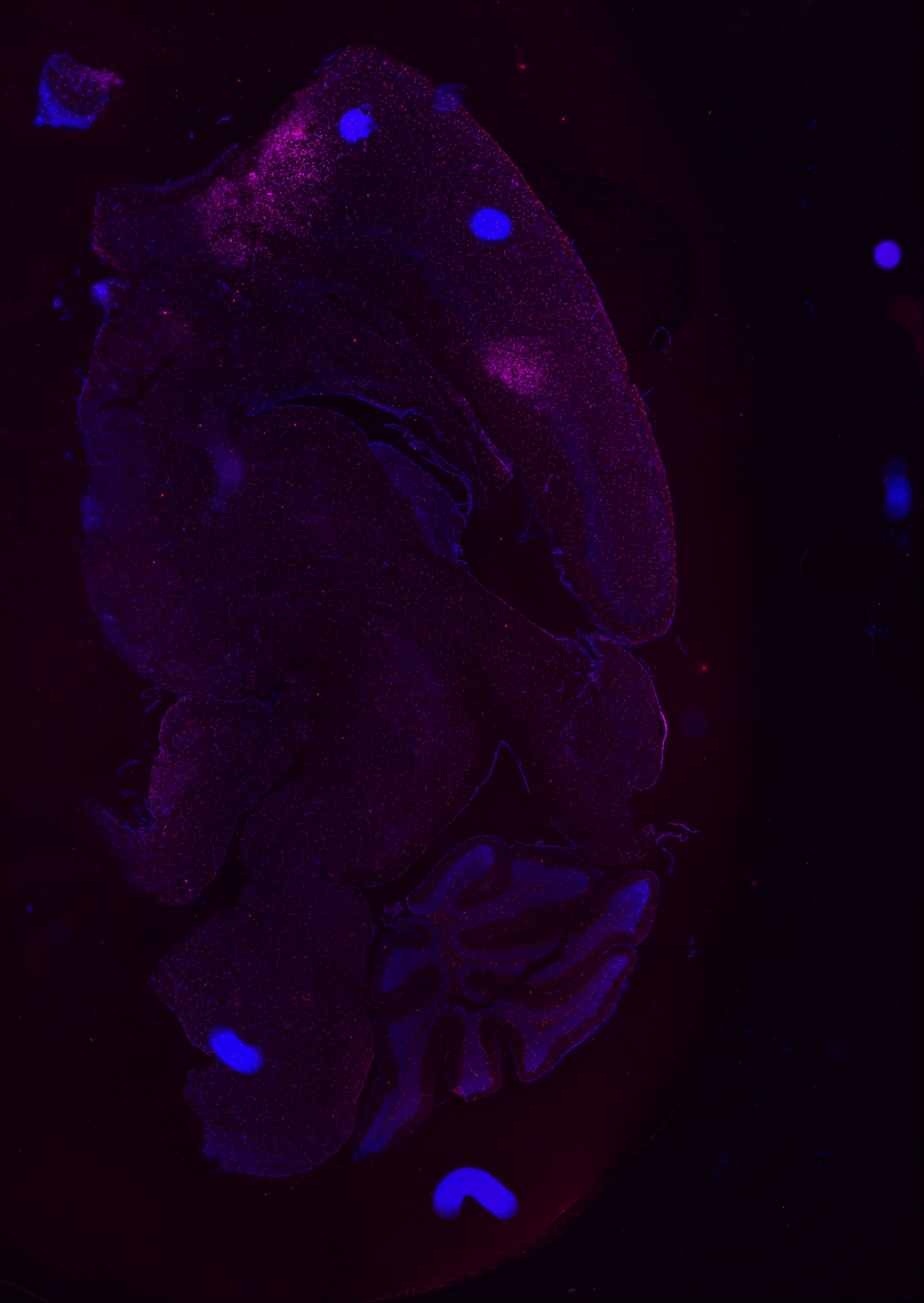

Supplement: Supplementary file 2. [file elife-102900-supp2.zip › Supplementary File 2/Raw Stitches/1138 ICT D1113H 13d 4x Stitch Overlay.jpeg]

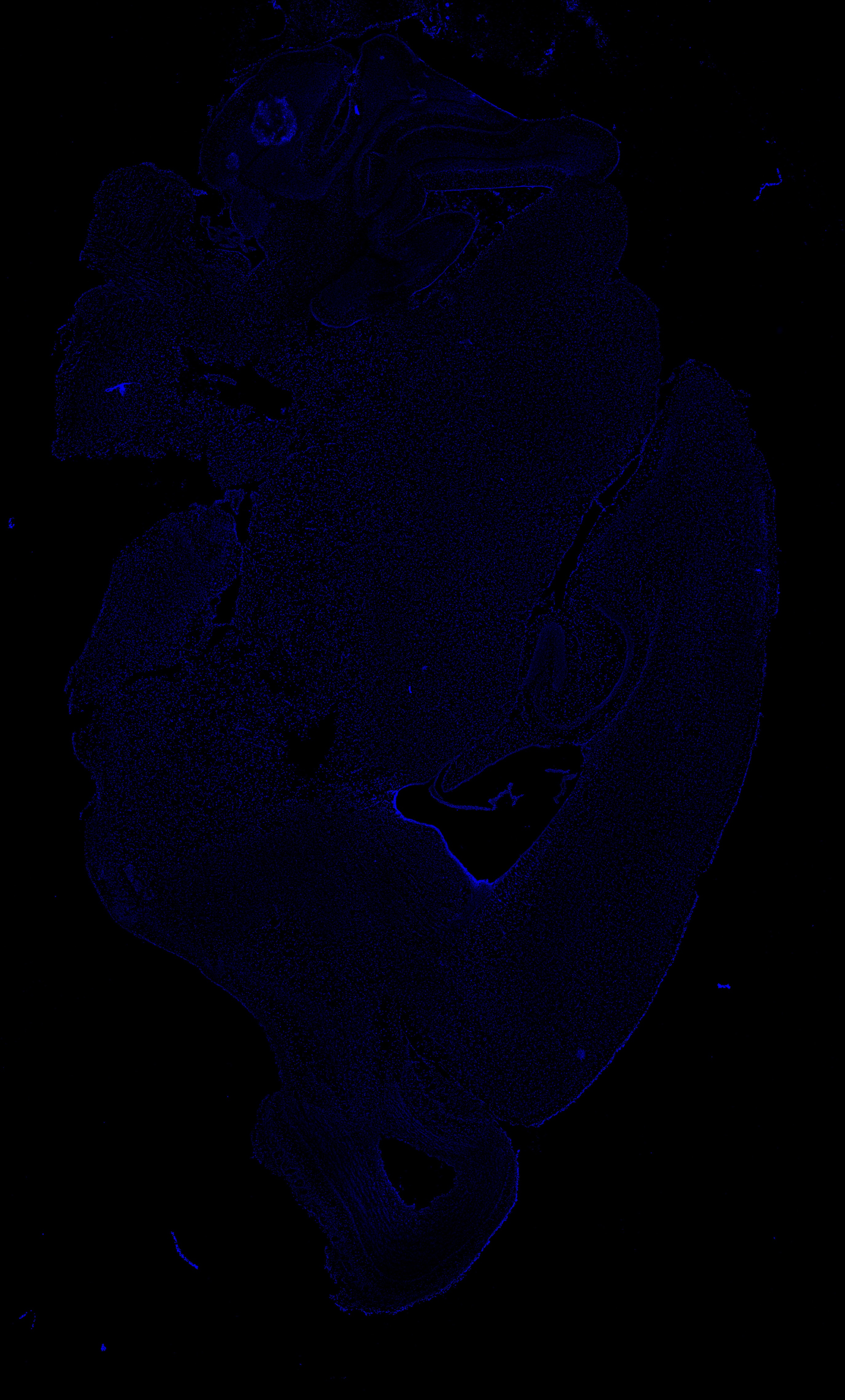

Supplement: Supplementary file 2. [file elife-102900-supp2.zip › Supplementary File 2/Raw Stitches/1224 Stitch DAPI.jpeg]

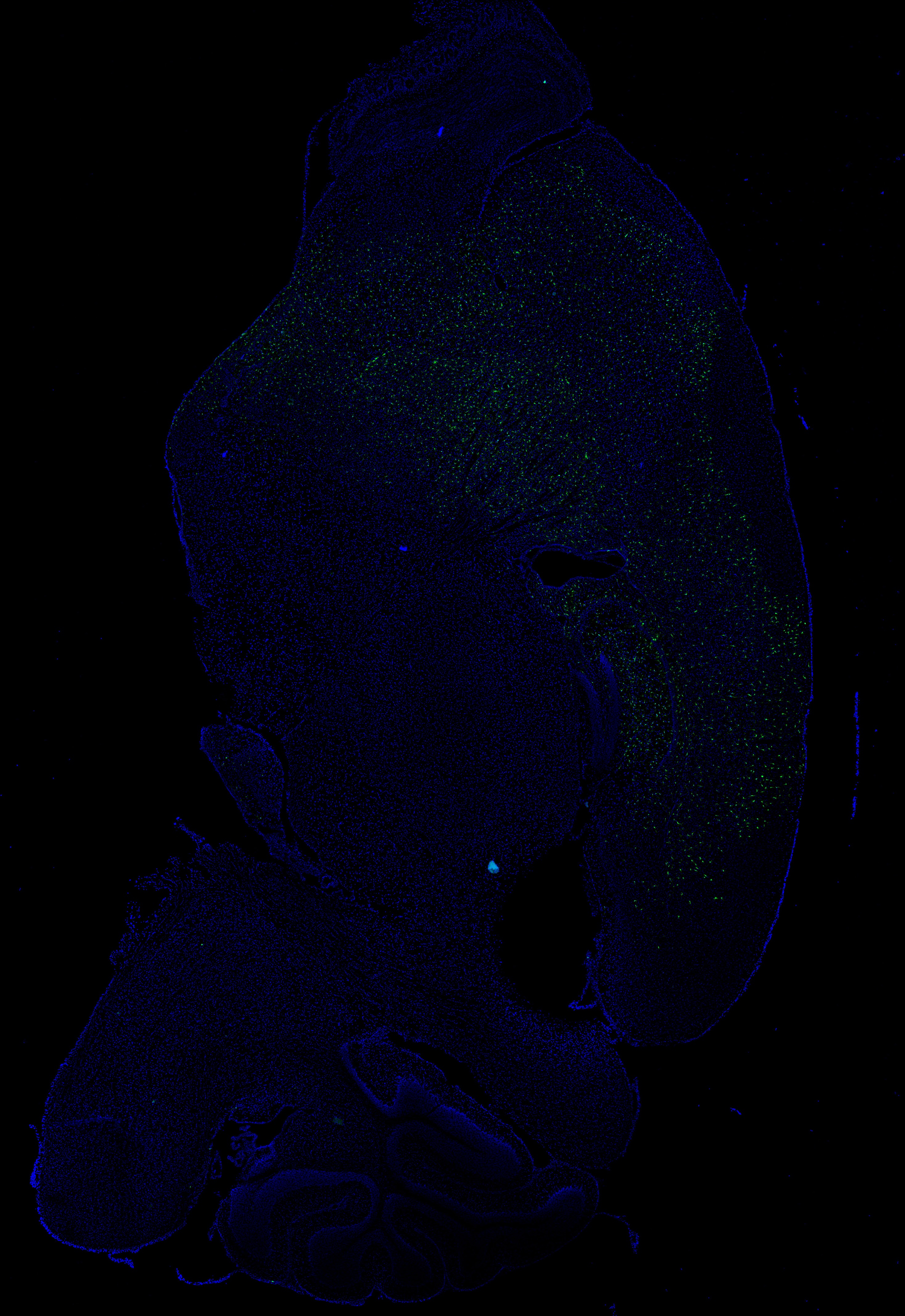

Supplement: Supplementary file 2. [file elife-102900-supp2.zip › Supplementary File 2/Raw Stitches/1076 Stitch Overlay.jpeg]

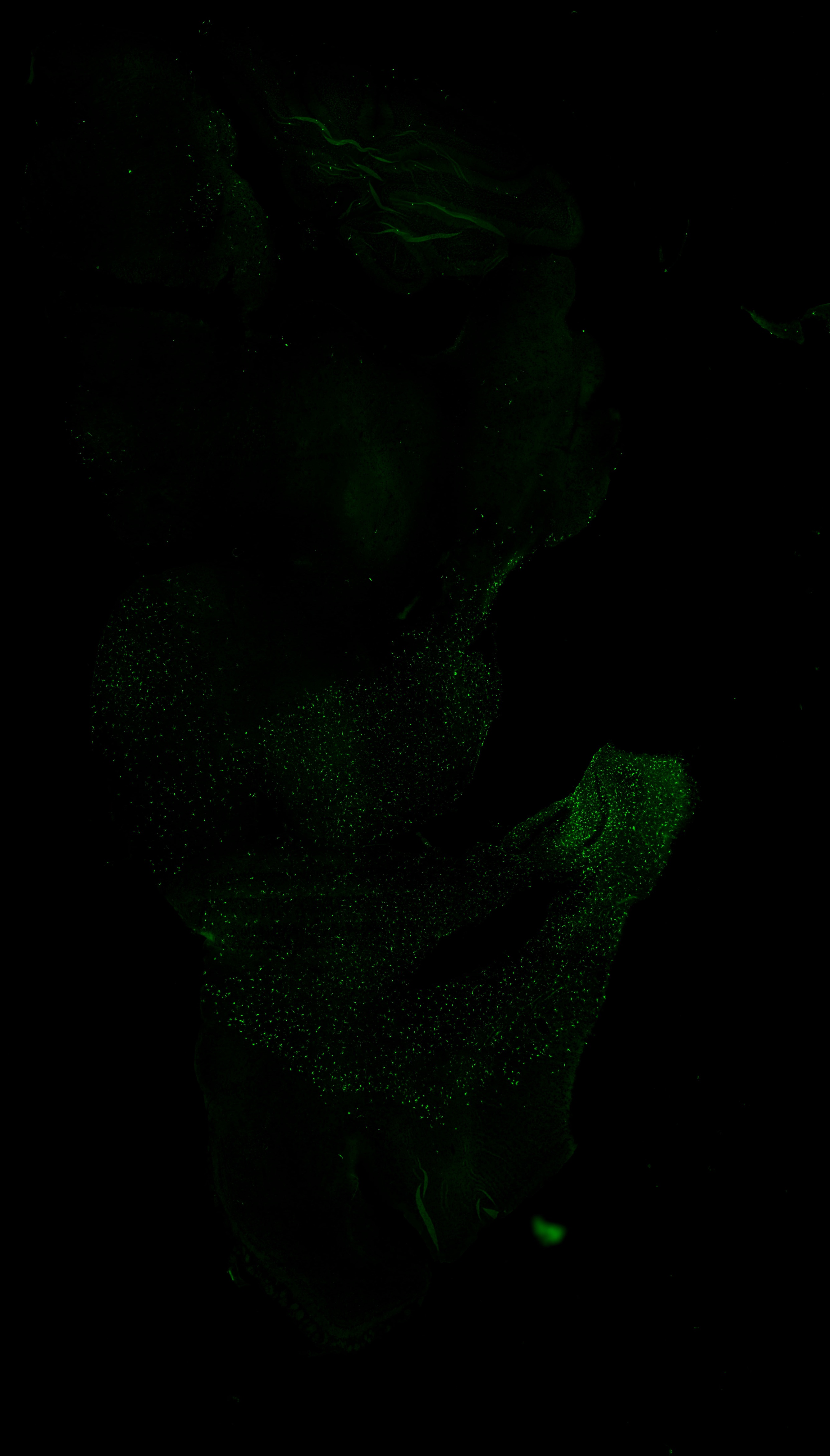

Supplement: Supplementary file 2. [file elife-102900-supp2.zip › Supplementary File 2/Raw Stitches/1071 Stitch GFP.jpeg]

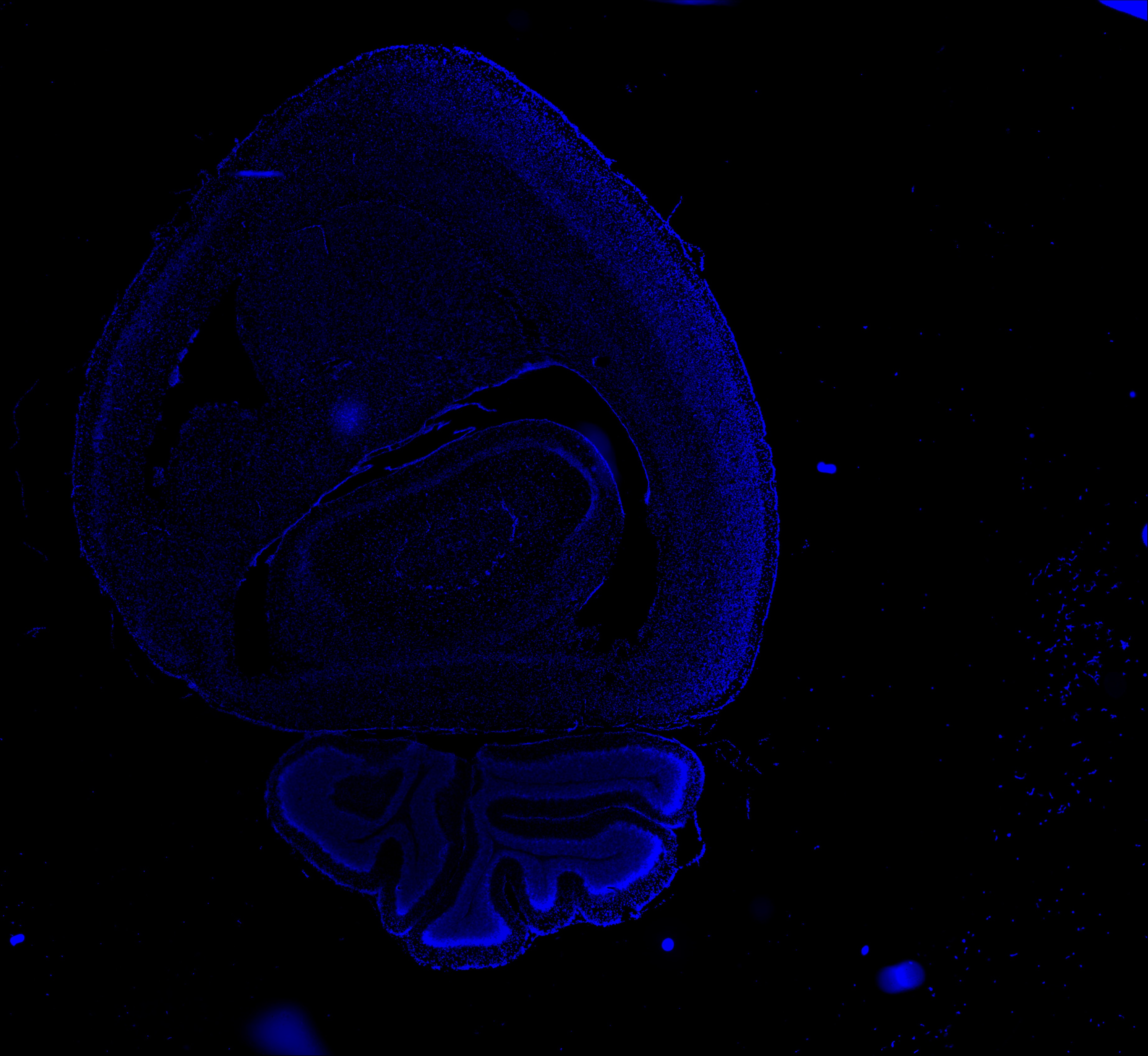

Supplement: Supplementary file 2. [file elife-102900-supp2.zip › Supplementary File 2/Raw Stitches/819 Stitch DAPI.jpeg]

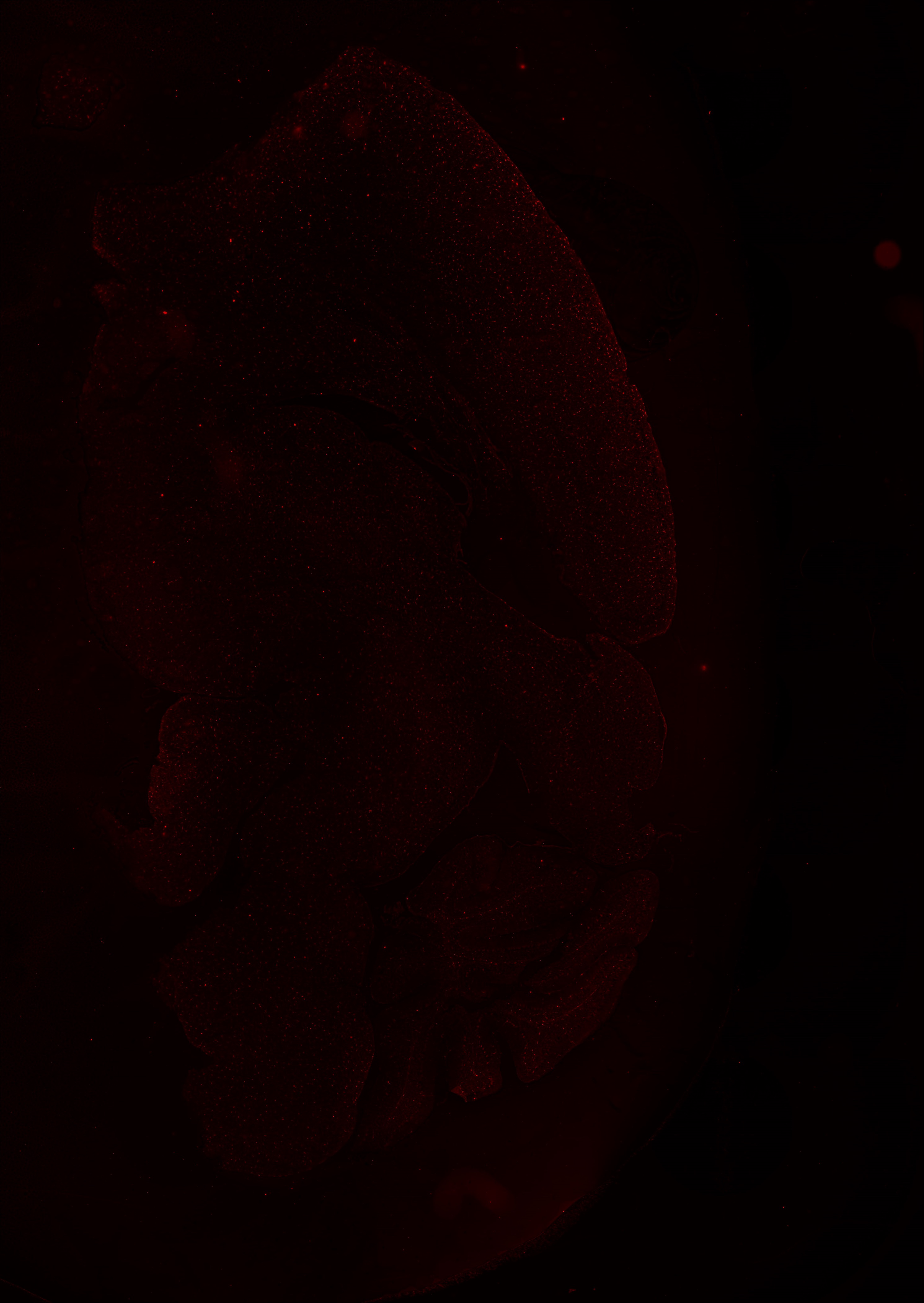

Supplement: Supplementary file 2. [file elife-102900-supp2.zip › Supplementary File 2/Raw Stitches/1138 ICT D1113H 13d 4x Stitch Iba.jpeg]

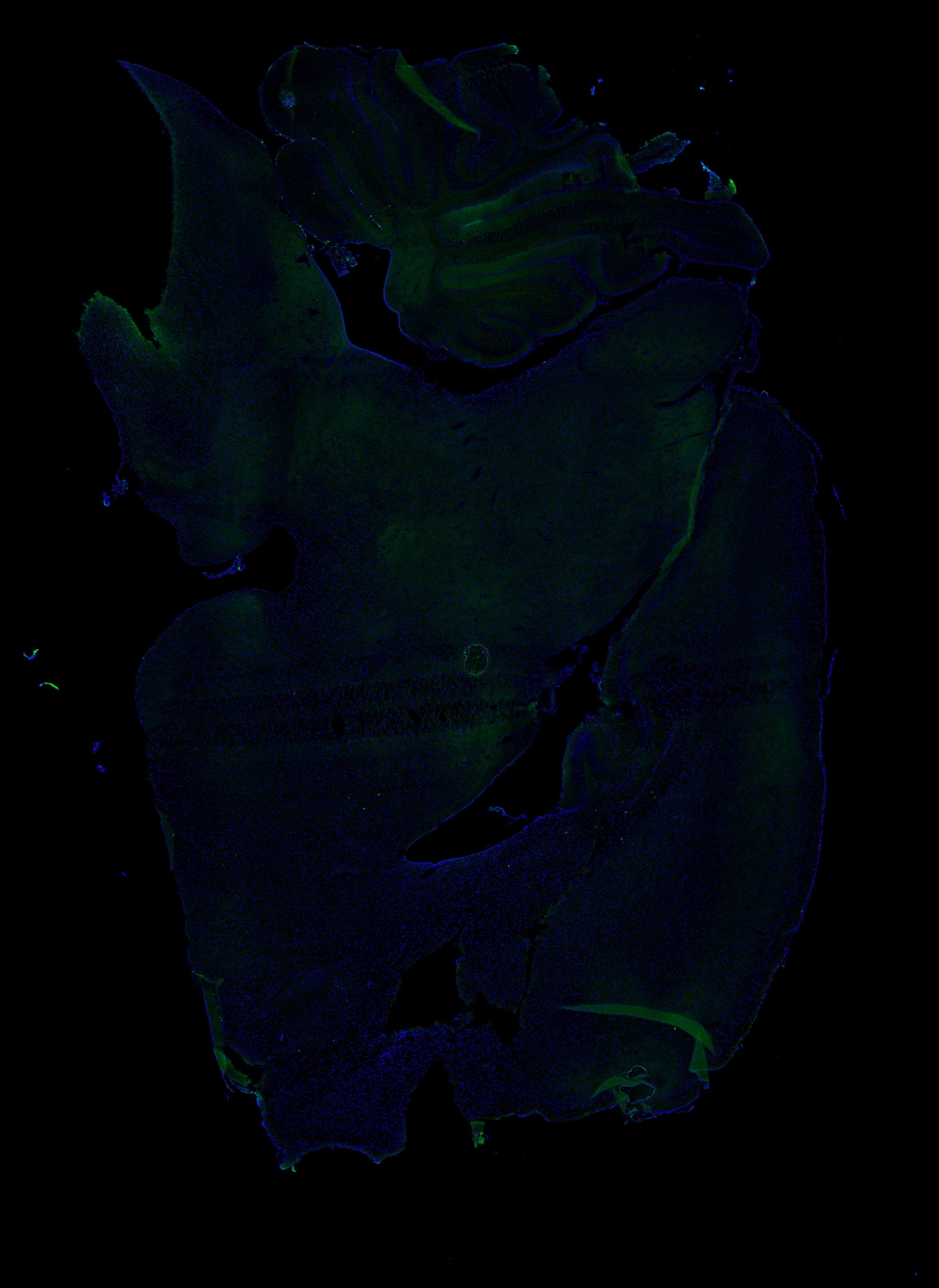

Supplement: Supplementary file 2. [file elife-102900-supp2.zip › Supplementary File 2/Raw Stitches/1237 Stitch Overlay.jpeg]

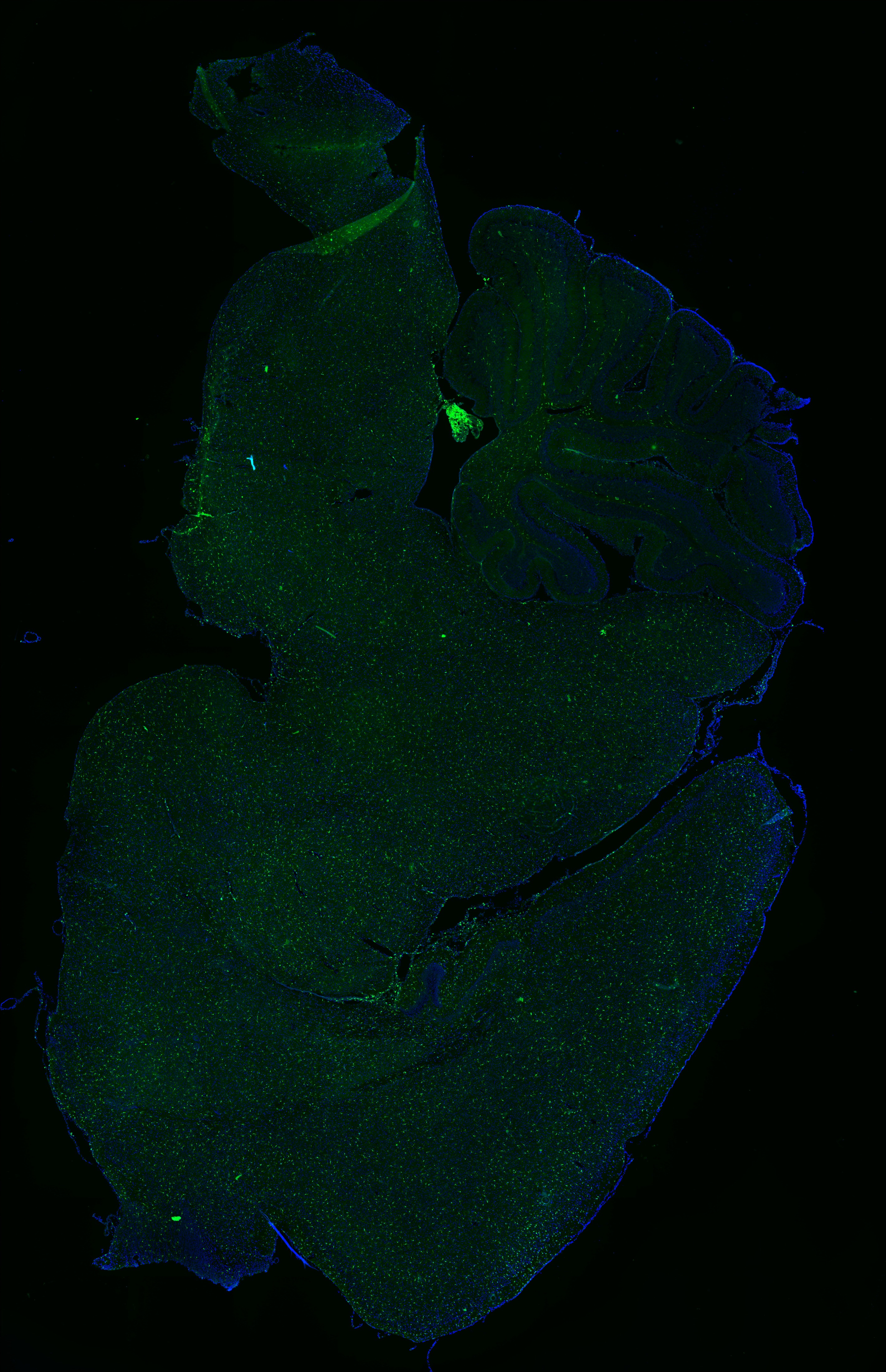

Supplement: Supplementary file 2. [file elife-102900-supp2.zip › Supplementary File 2/Raw Stitches/XY03 828 Stitch Overlay.jpeg]

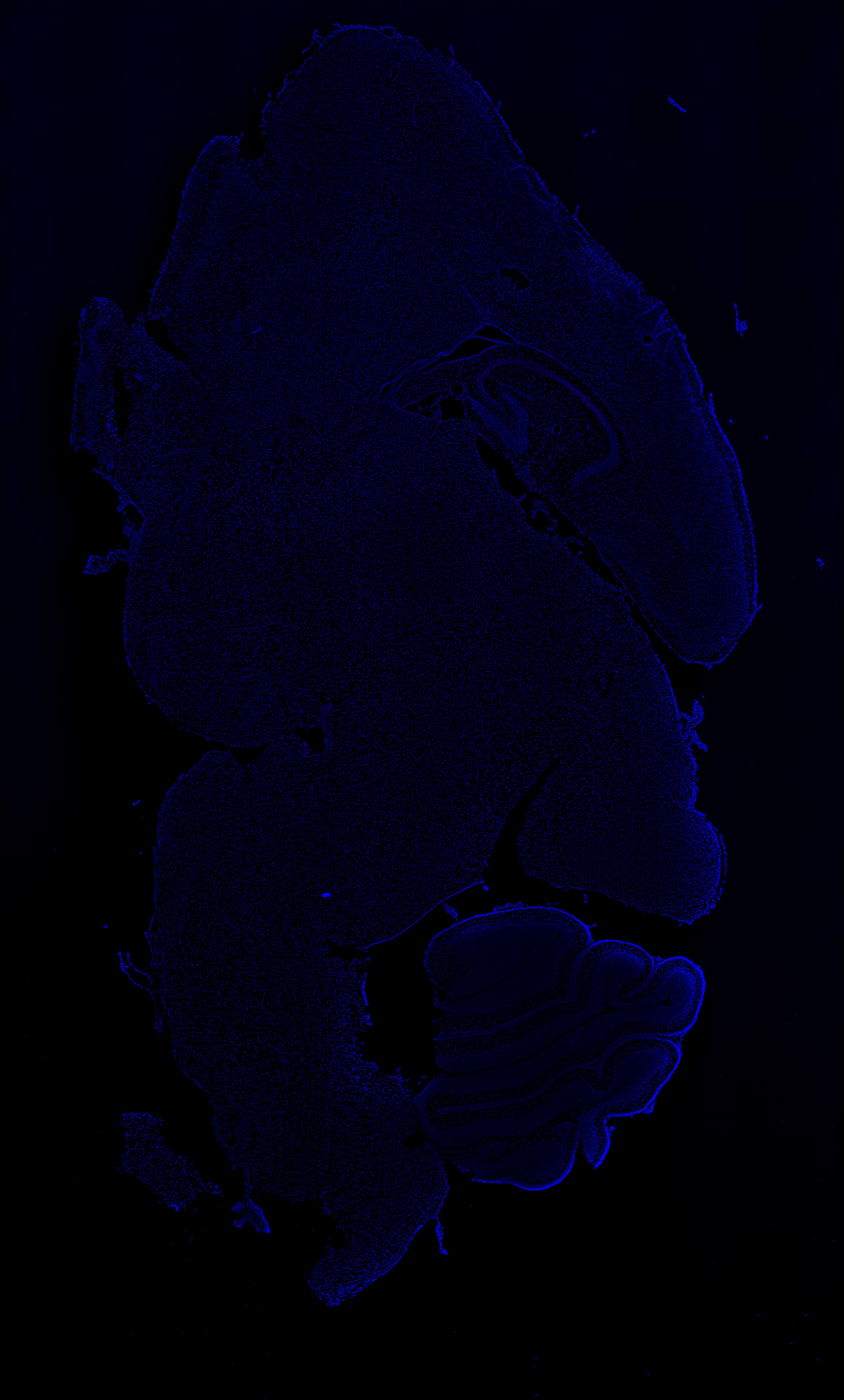

Supplement: Supplementary file 2. [file elife-102900-supp2.zip › Supplementary File 2/Raw Stitches/1029 Stitch DAPI.jpeg]

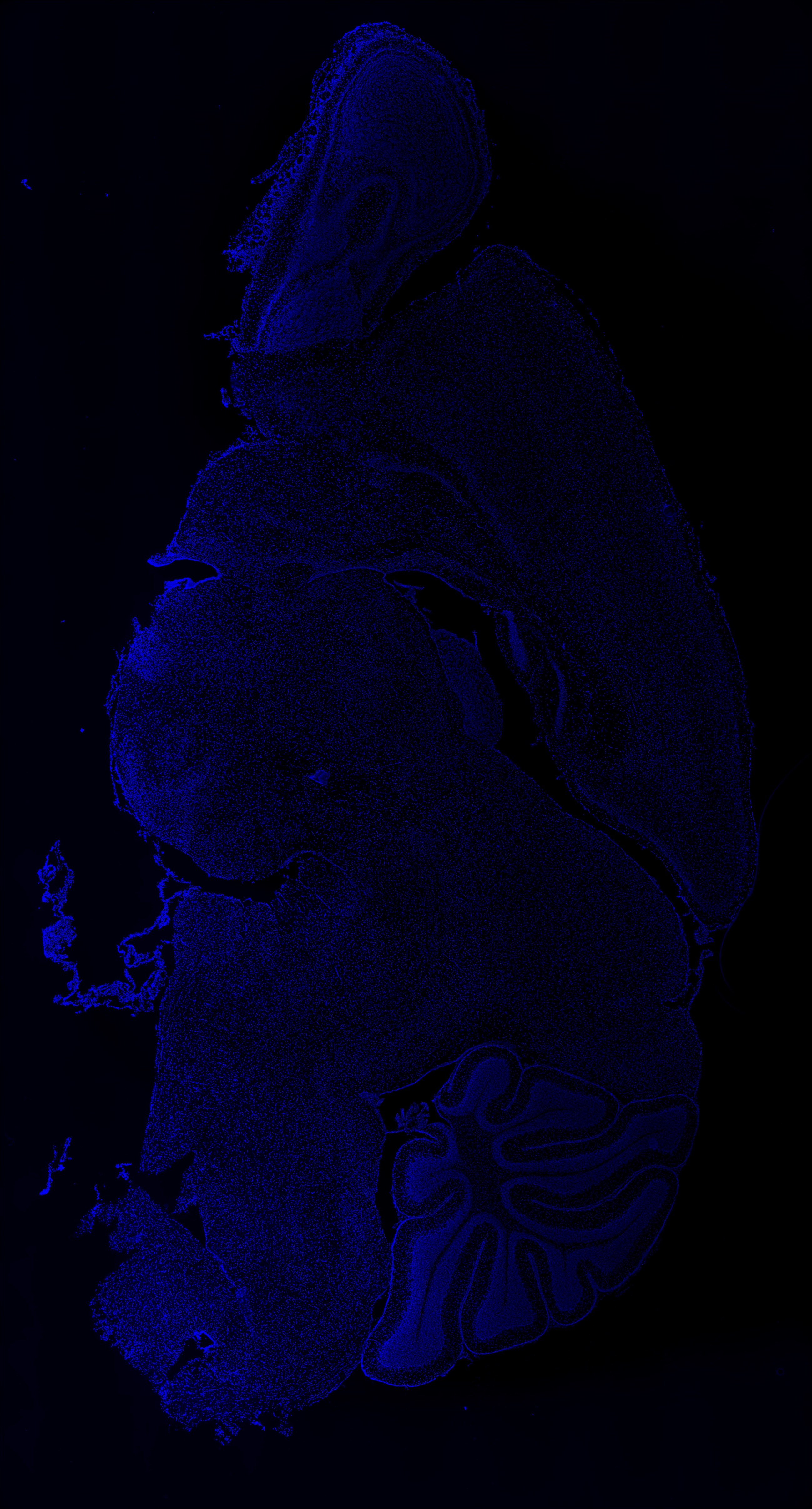

Supplement: Supplementary file 2. [file elife-102900-supp2.zip › Supplementary File 2/Raw Stitches/1017 Stitch DAPI.jpeg]

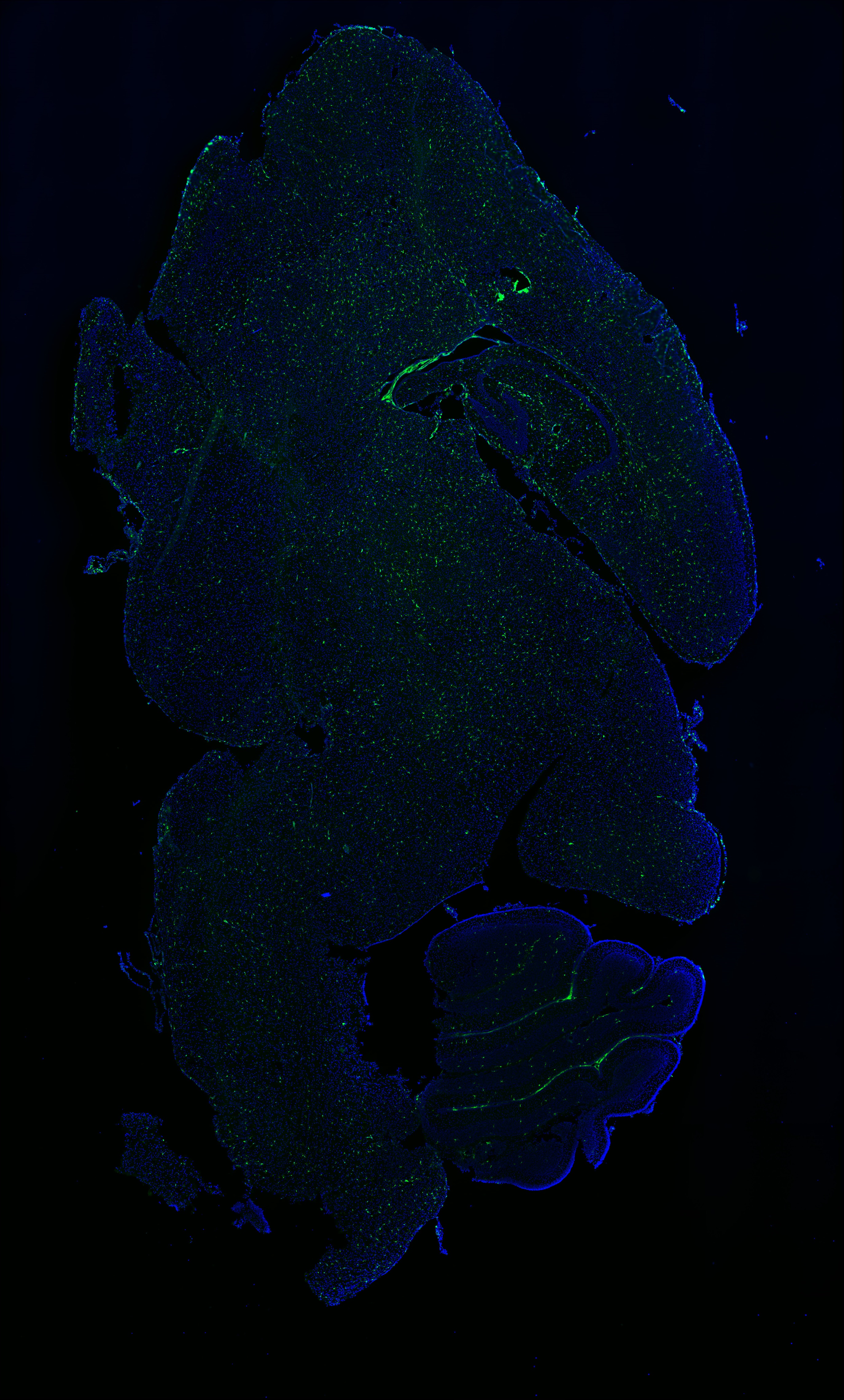

Supplement: Supplementary file 2. [file elife-102900-supp2.zip › Supplementary File 2/Raw Stitches/1029 Stitch Overlay.jpeg]

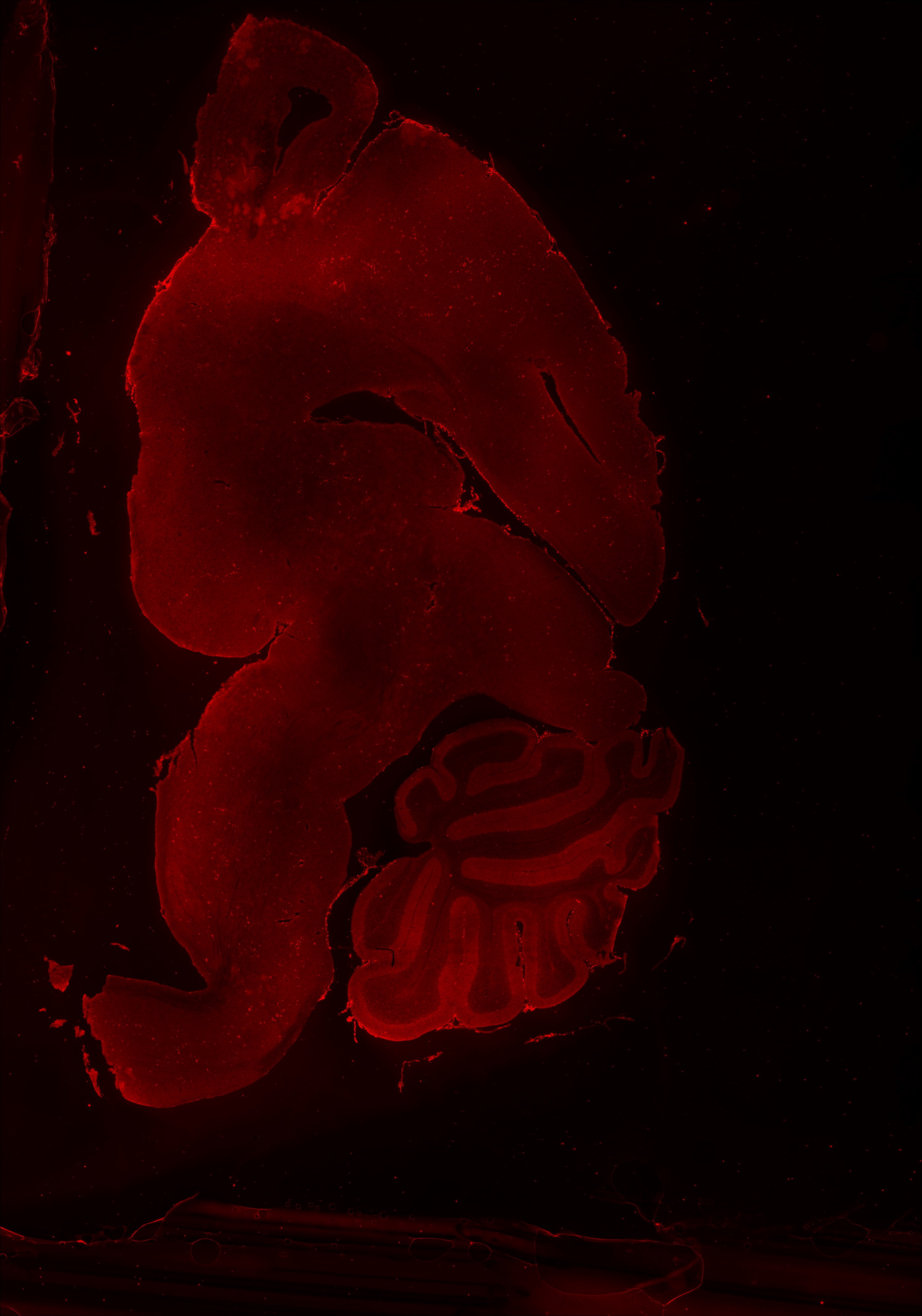

Supplement: Supplementary file 2. [file elife-102900-supp2.zip › Supplementary File 2/Raw Stitches/1264 Stitch Iba.jpeg]

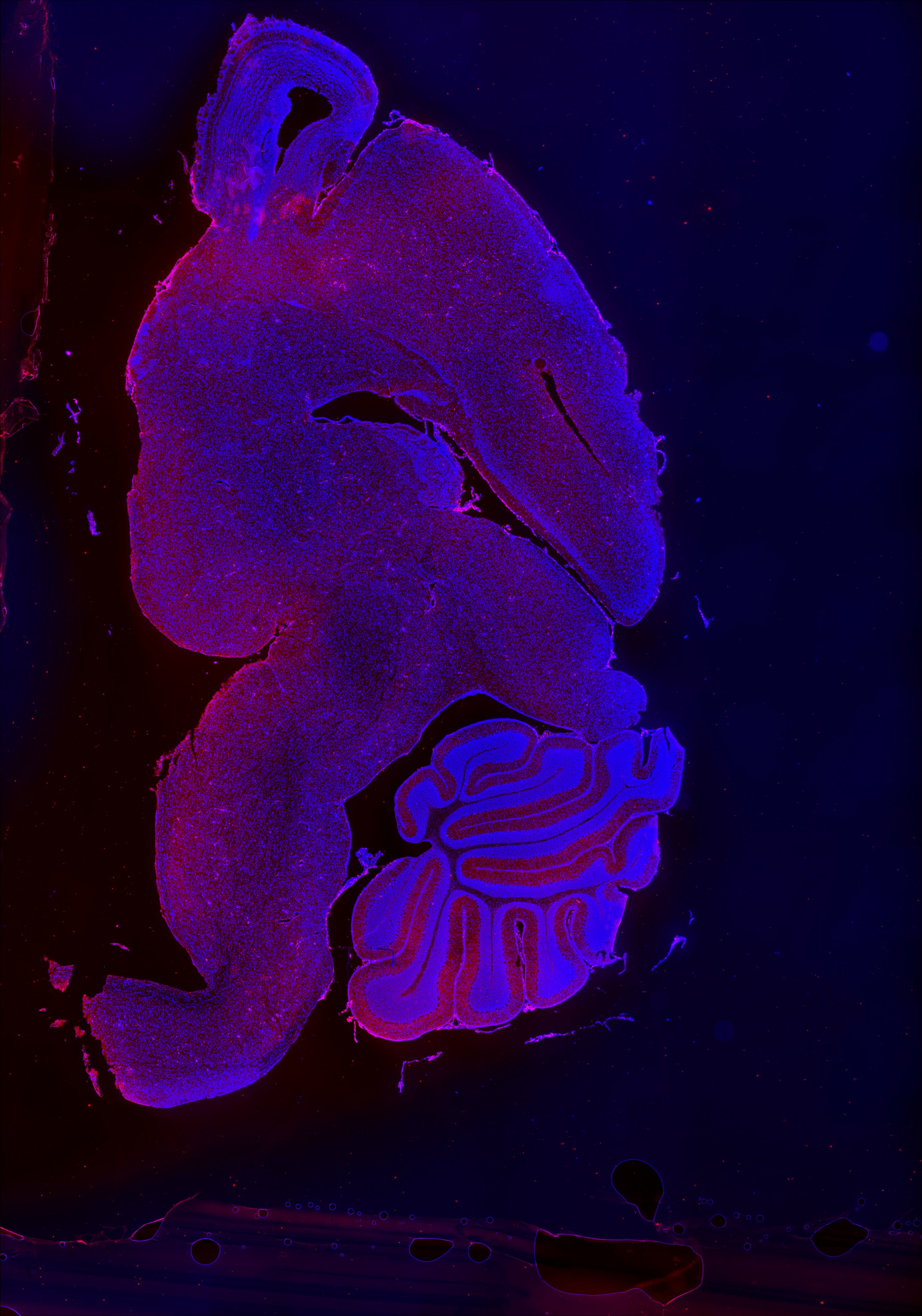

Supplement: Supplementary file 2. [file elife-102900-supp2.zip › Supplementary File 2/Raw Stitches/1264 Stitch Overlay.jpeg]

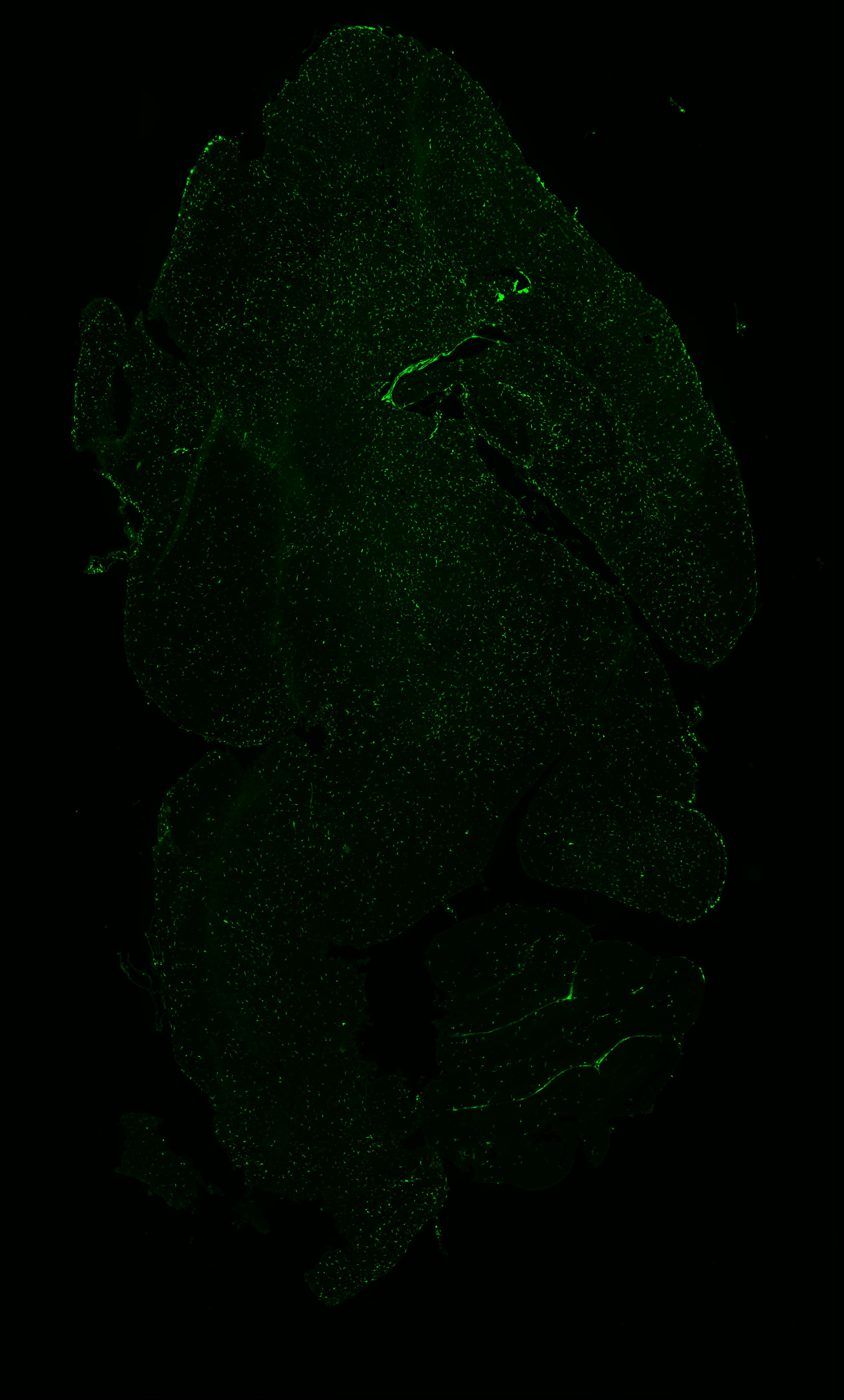

Supplement: Supplementary file 2. [file elife-102900-supp2.zip › Supplementary File 2/Raw Stitches/1029 Stitch GFP.jpeg]

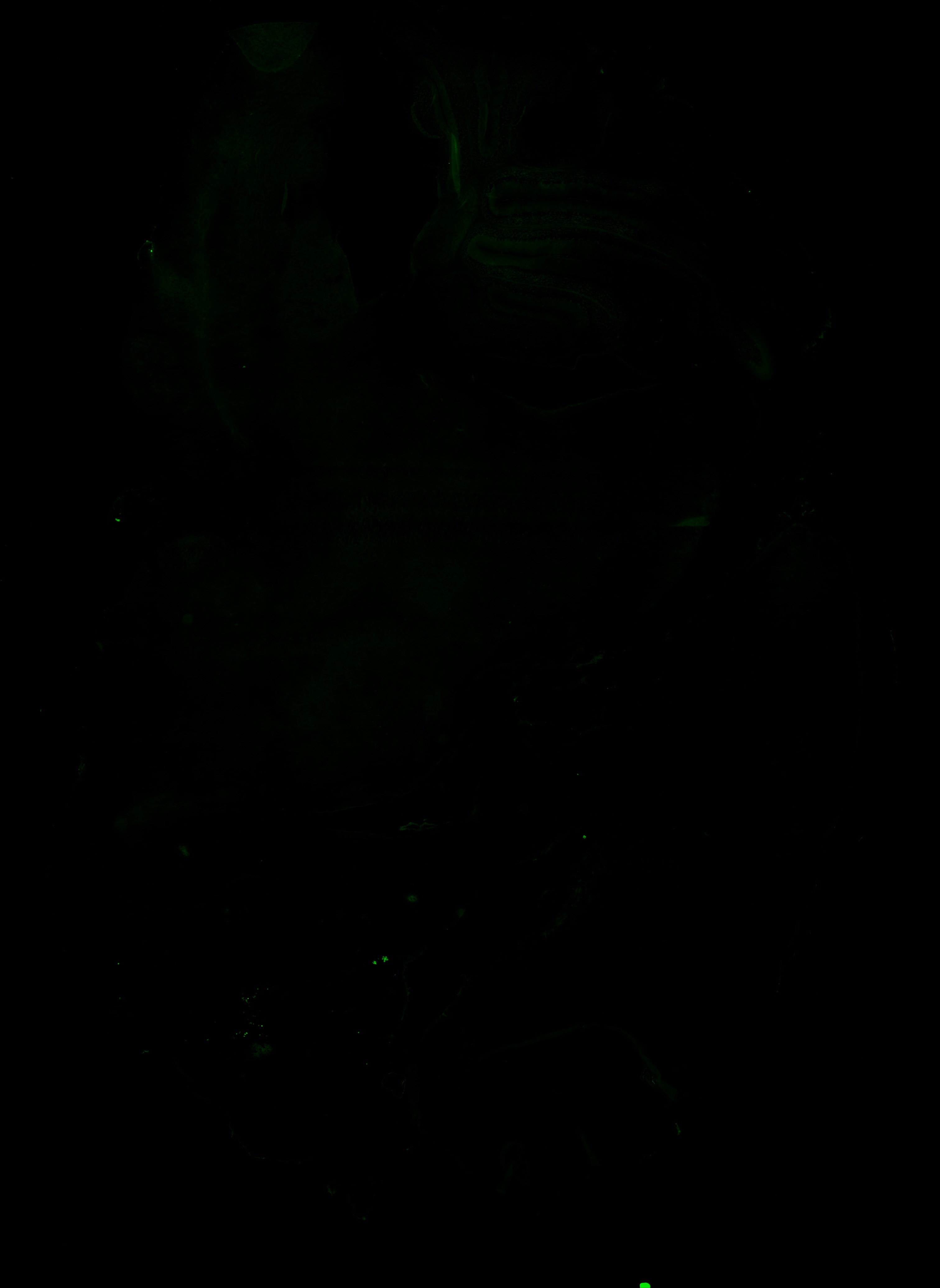

Supplement: Supplementary file 2. [file elife-102900-supp2.zip › Supplementary File 2/Raw Stitches/1239 Stitch GFP.jpeg]

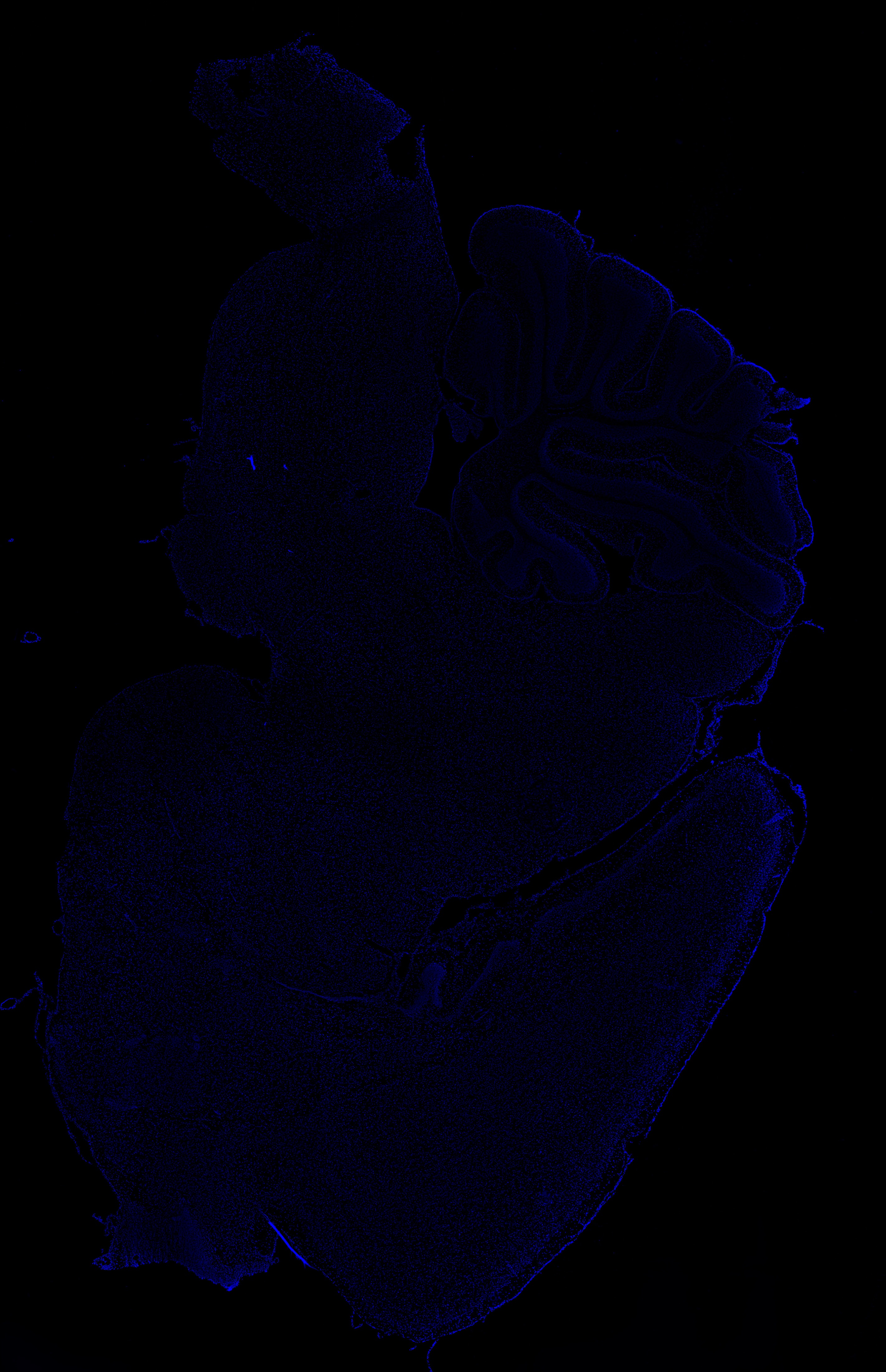

Supplement: Supplementary file 2. [file elife-102900-supp2.zip › Supplementary File 2/Raw Stitches/XY03 828 Stitch DAPI.jpeg]

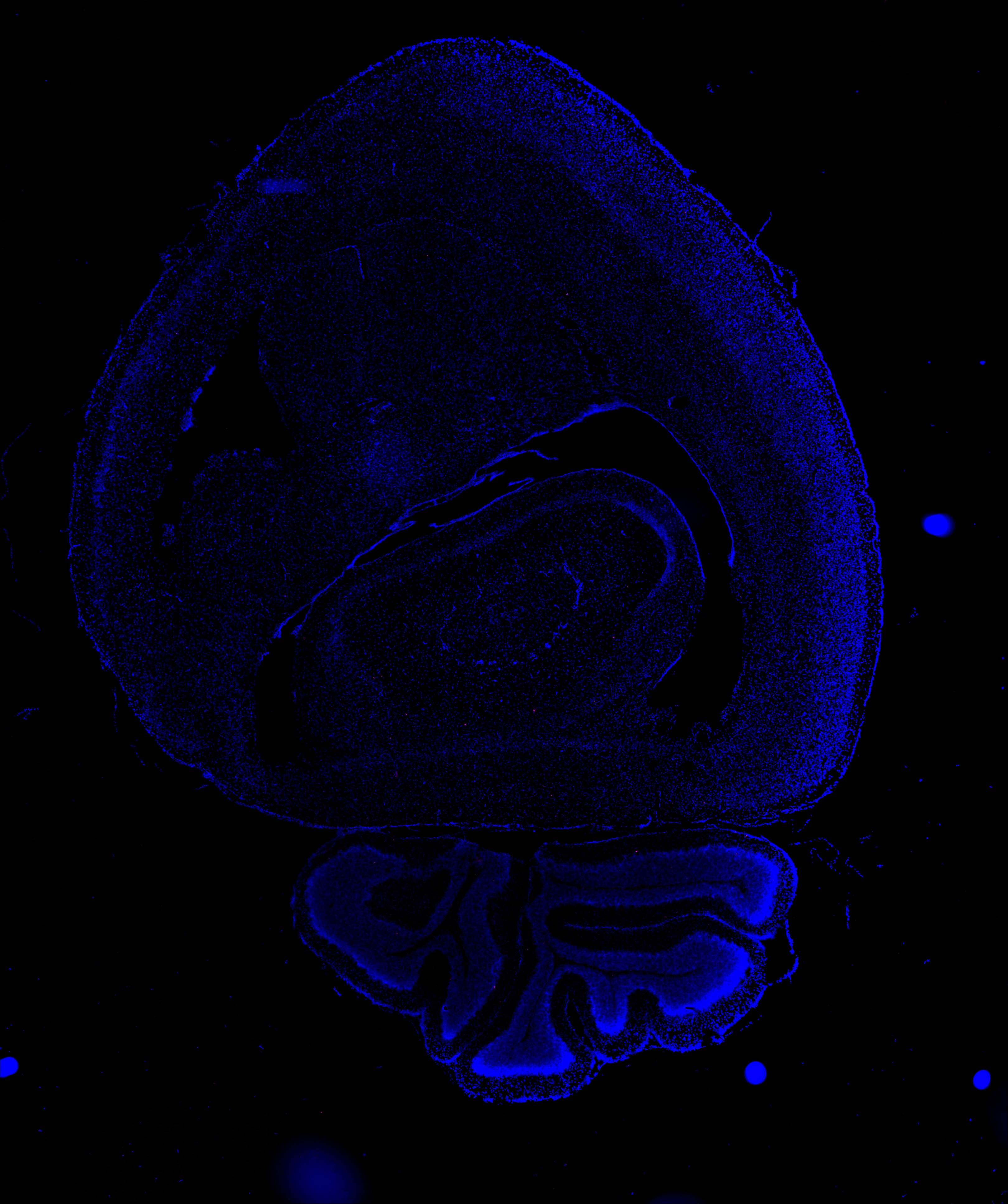

Supplement: Supplementary file 2. [file elife-102900-supp2.zip › Supplementary File 2/Raw Stitches/819 Stitch Overlay 2.jpeg]

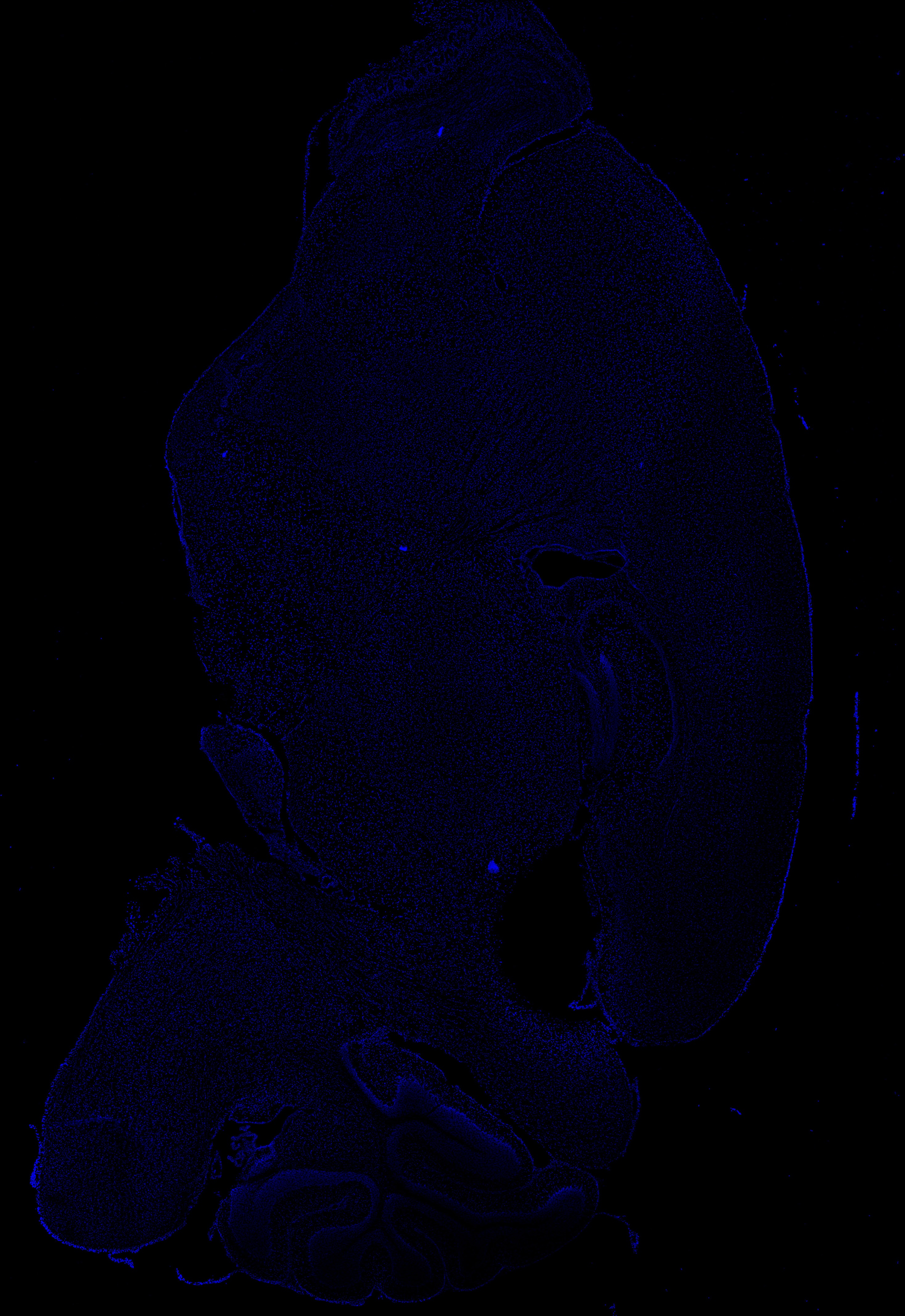

Supplement: Supplementary file 2. [file elife-102900-supp2.zip › Supplementary File 2/Raw Stitches/1076 Stitch DAPI.jpeg]

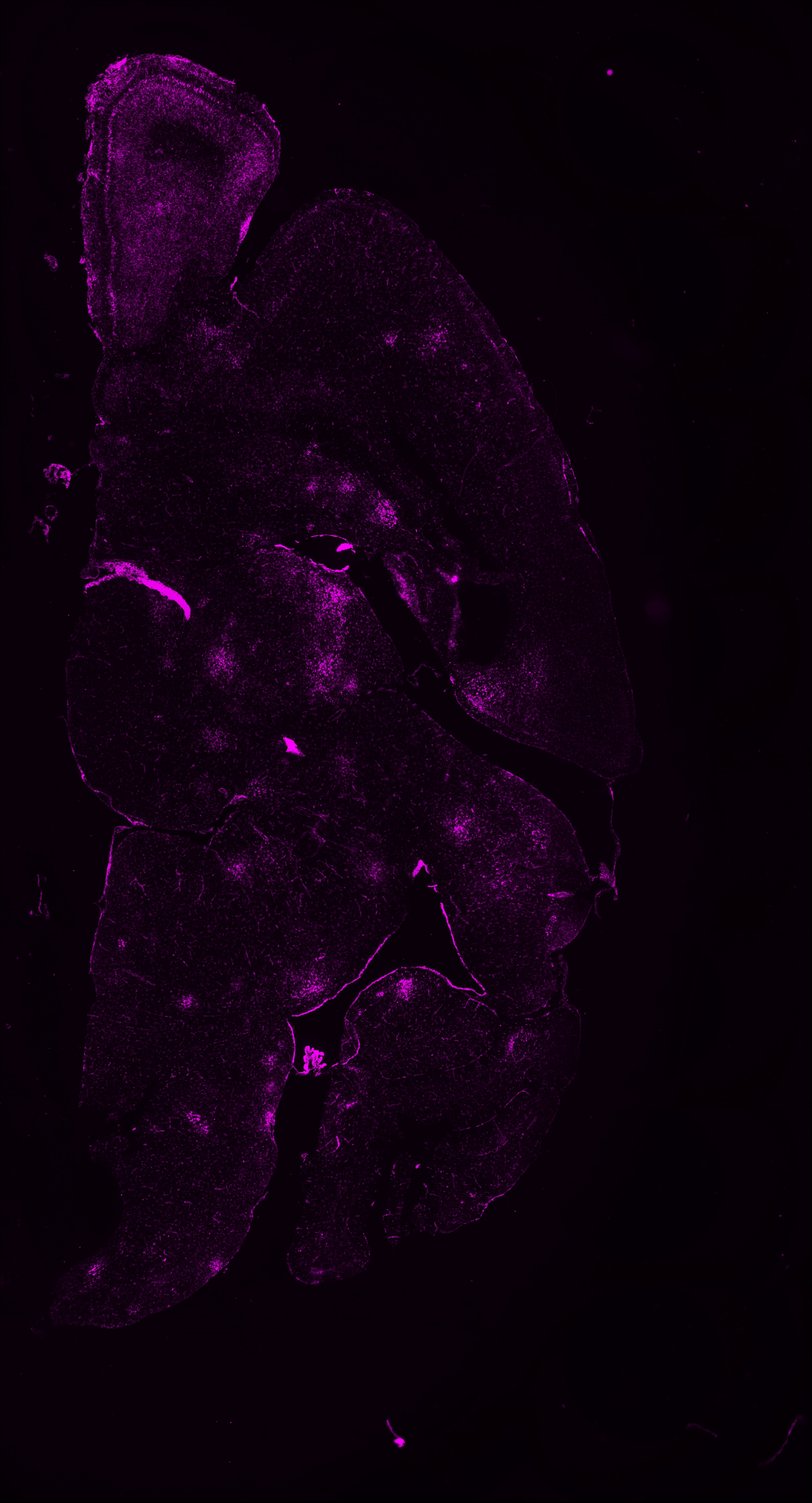

Supplement: Supplementary file 2. [file elife-102900-supp2.zip › Supplementary File 2/Raw Stitches/1180 Full D1113H 14d 4x Stitch Isg.jpeg]

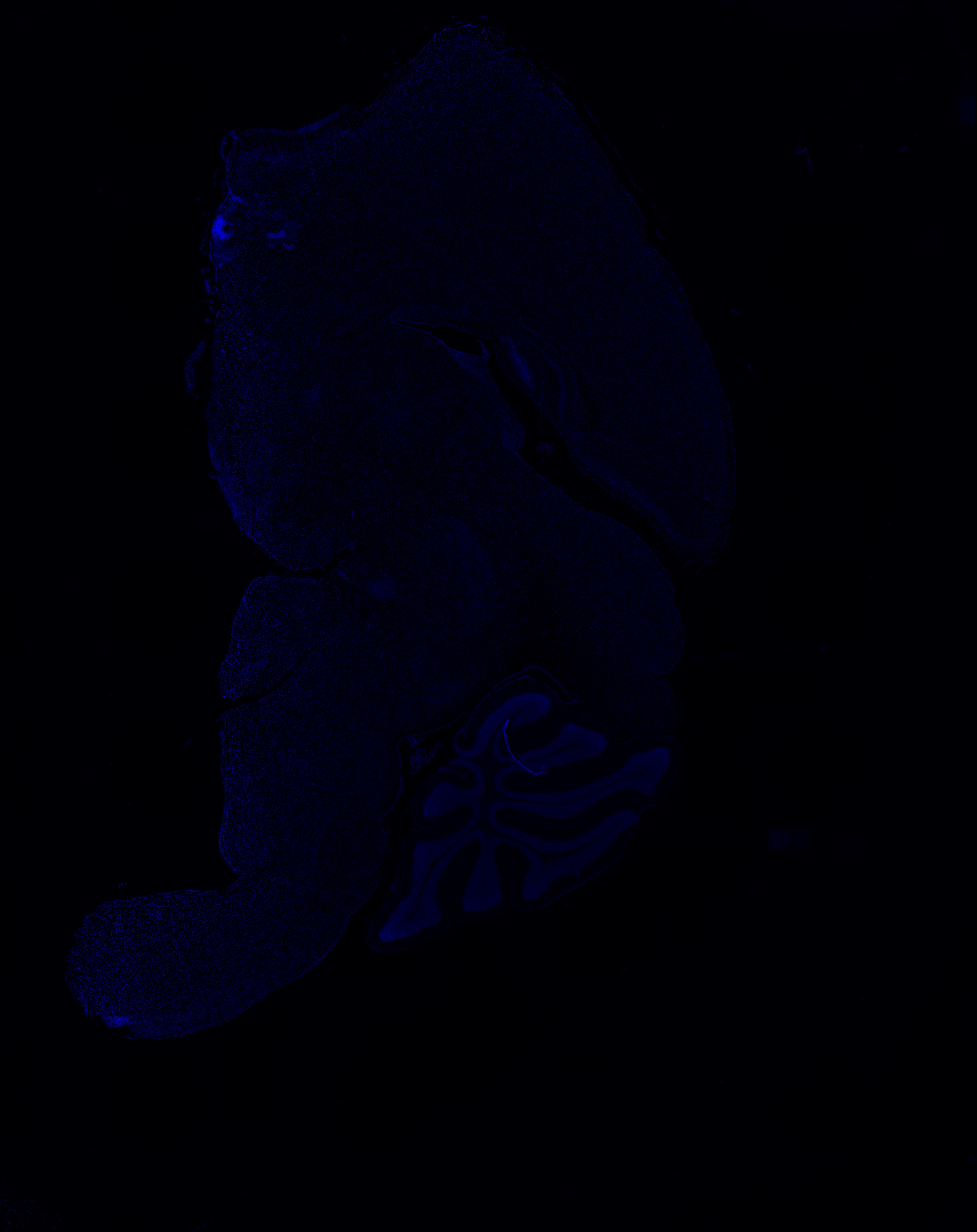

Supplement: Supplementary file 2. [file elife-102900-supp2.zip › Supplementary File 2/Raw Stitches/1144 ICT WT 13dpi 4x Stitch DAPI.jpeg]

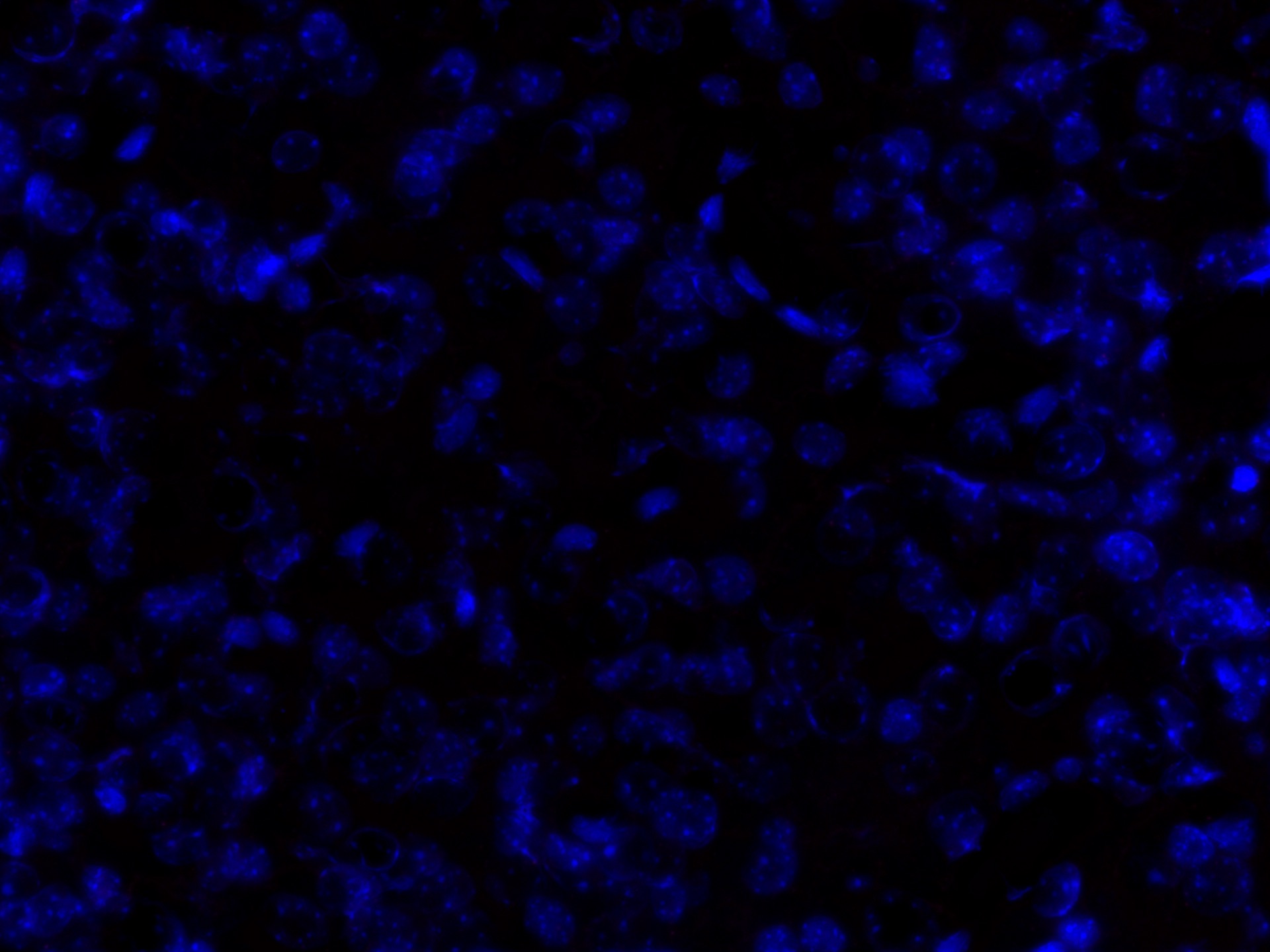

Supplement: Supplementary file 2. [file elife-102900-supp2.zip › Supplementary File 2/Raw IHC/819 Overlay 2.jpeg]

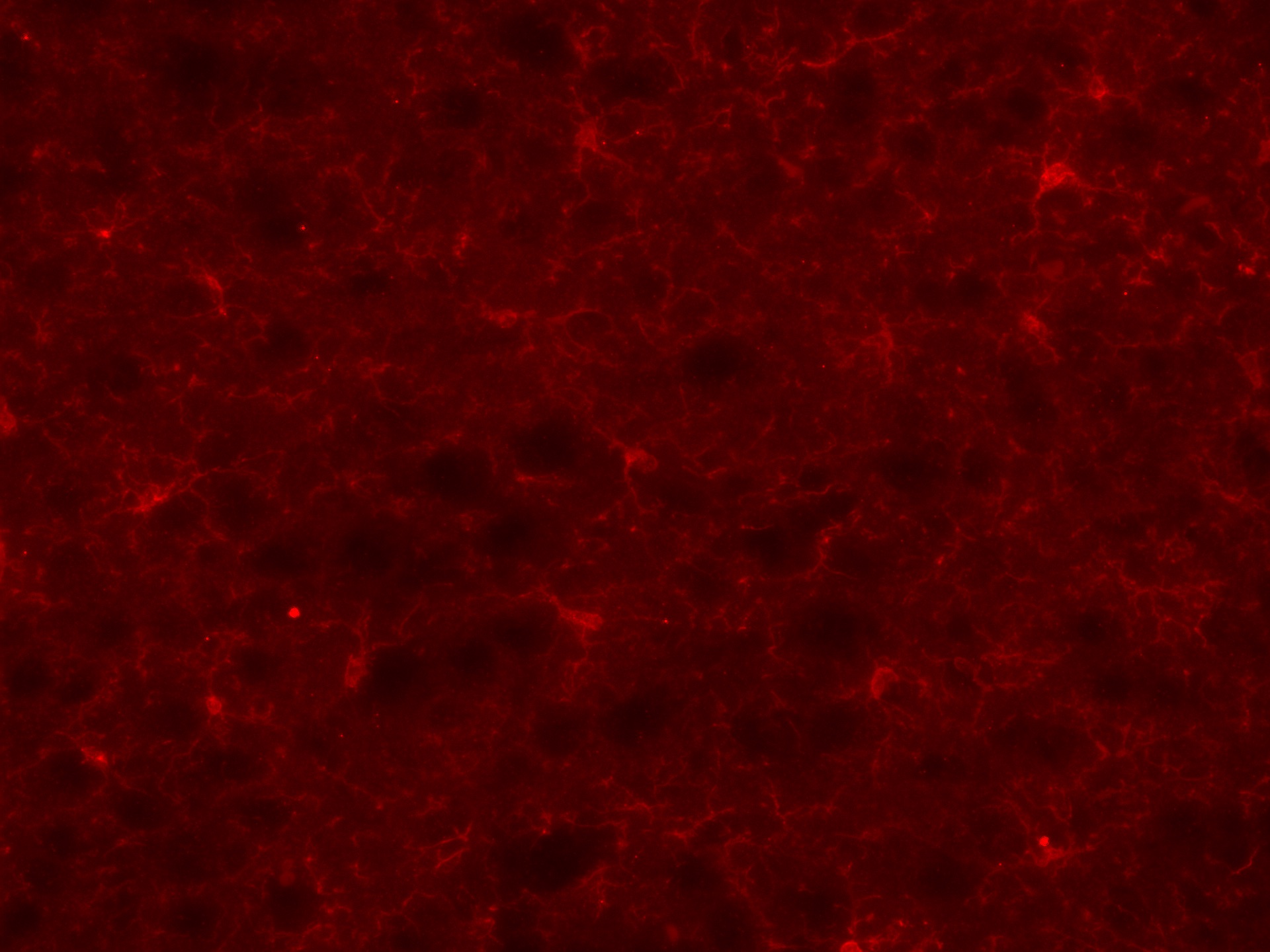

Supplement: Supplementary file 2. [file elife-102900-supp2.zip › Supplementary File 2/Raw IHC/FF_833 wt H8 02 iba1.jpeg]

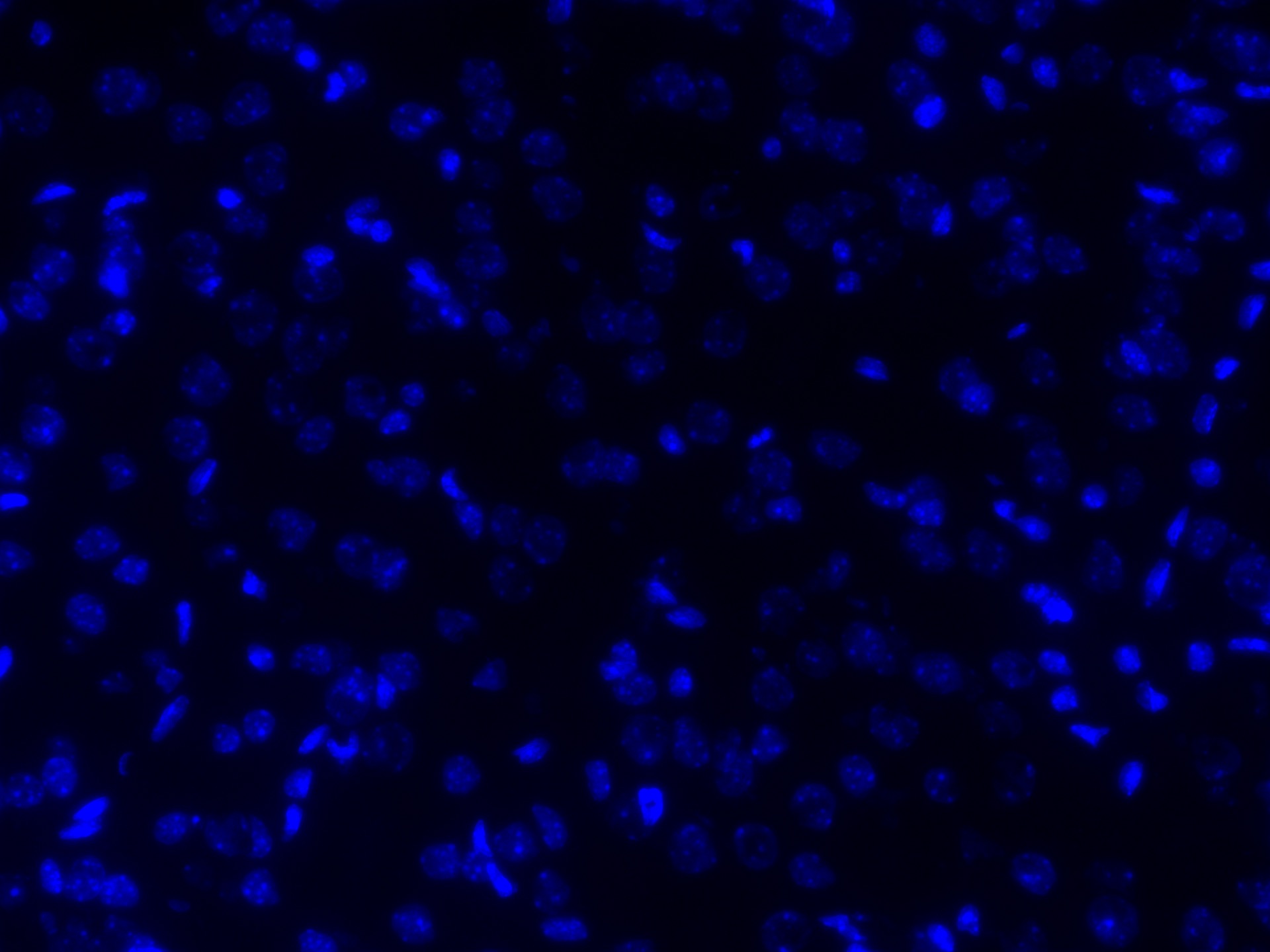

Supplement: Supplementary file 2. [file elife-102900-supp2.zip › Supplementary File 2/Raw IHC/dapi 2.jpeg]

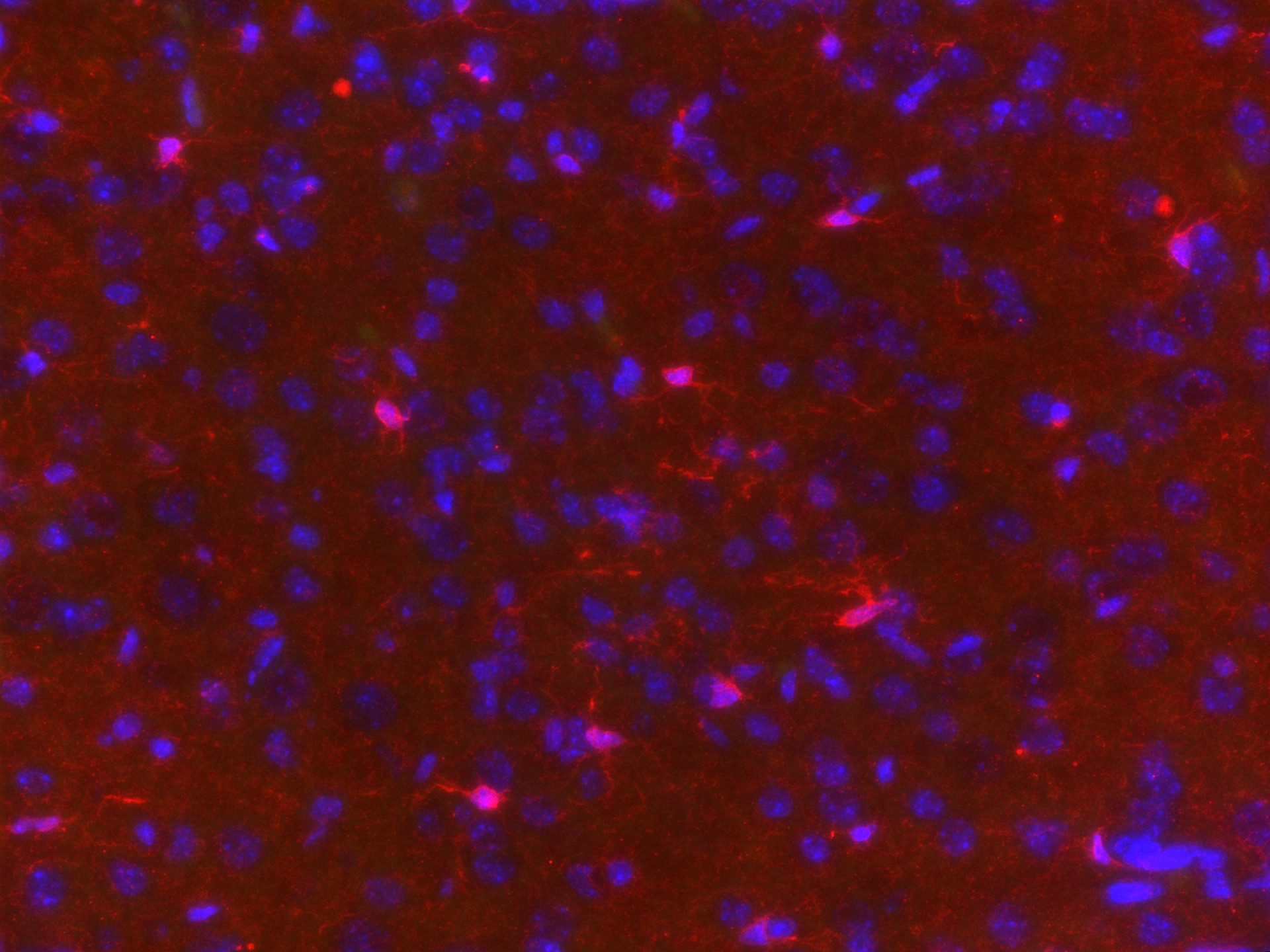

Supplement: Supplementary file 2. [file elife-102900-supp2.zip › Supplementary File 2/Raw IHC/Overlay 4.jpeg]

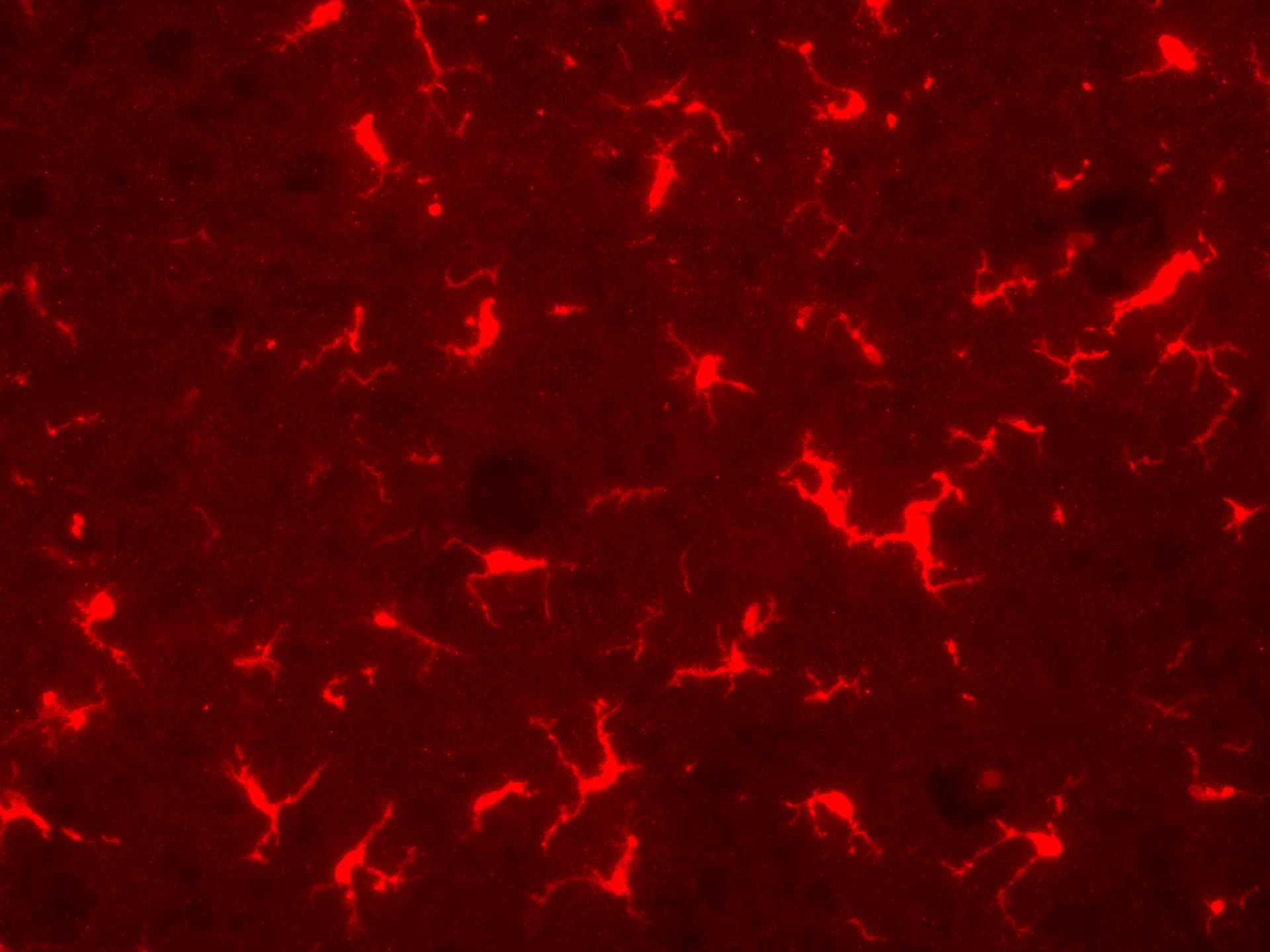

Supplement: Supplementary file 2. [file elife-102900-supp2.zip › Supplementary File 2/Raw IHC/FF_828_8 hoxb tx D8 iba1.jpeg]

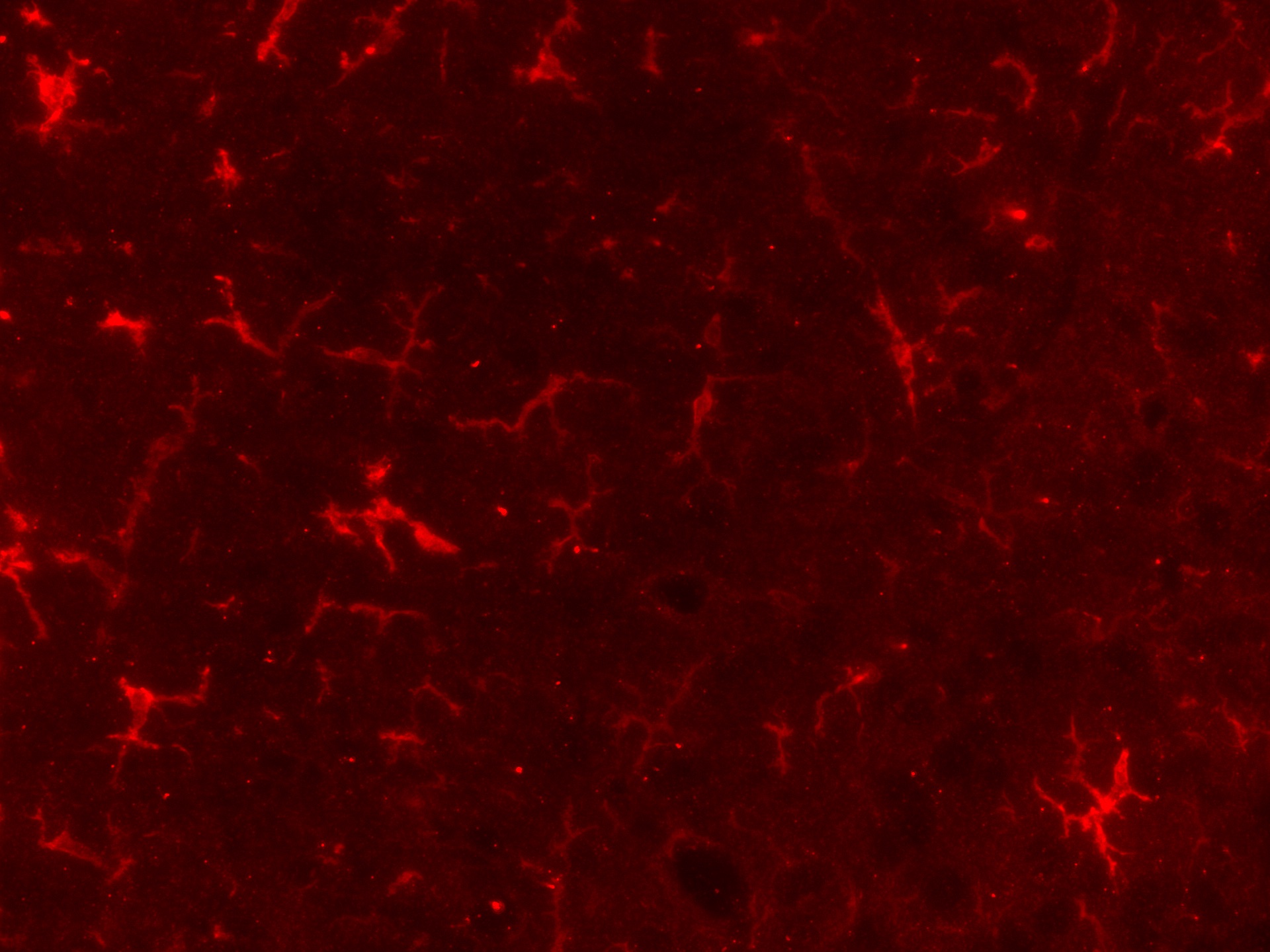

Supplement: Supplementary file 2. [file elife-102900-supp2.zip › Supplementary File 2/Raw IHC/Iba.jpeg]

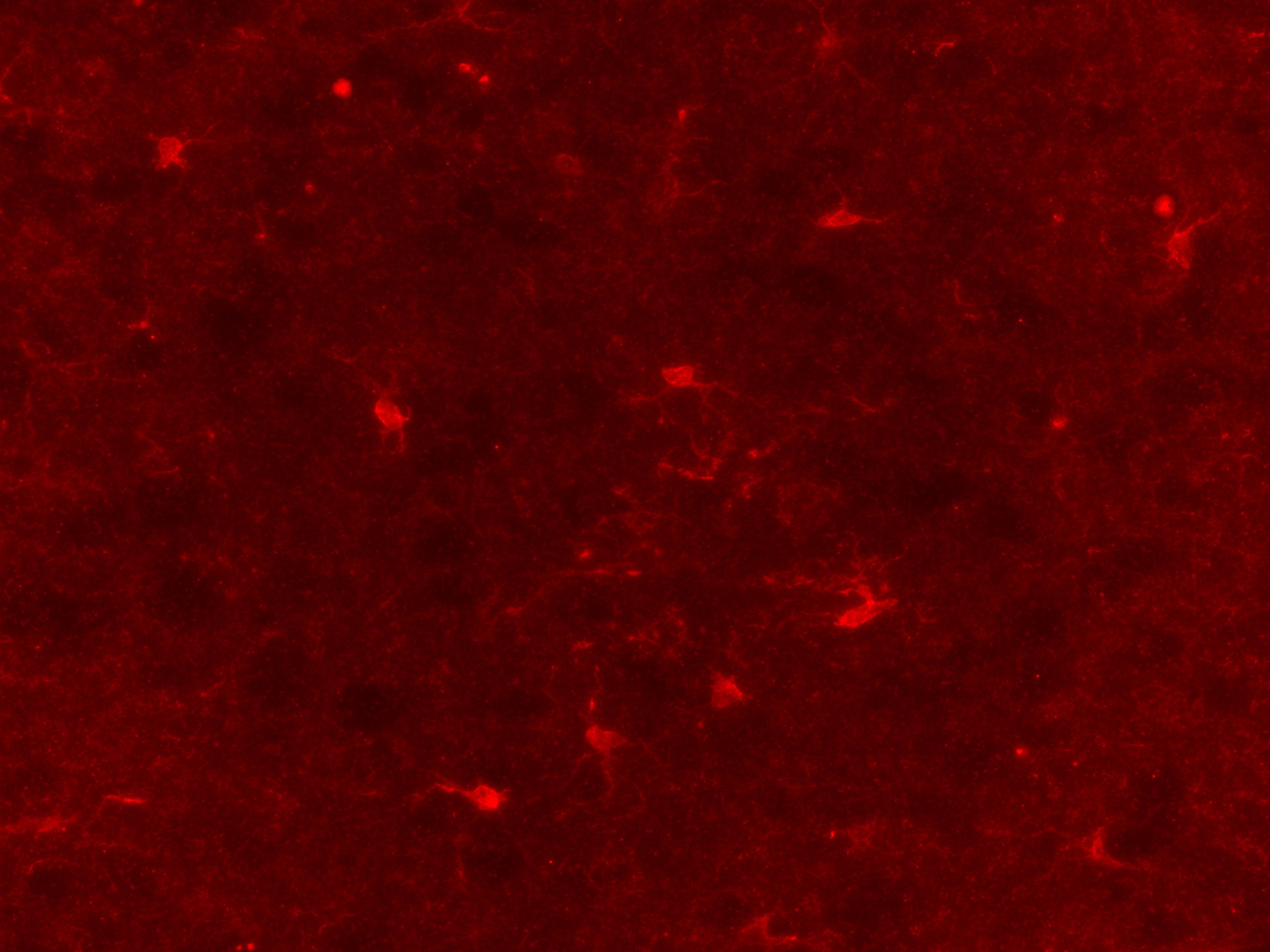

Supplement: Supplementary file 2. [file elife-102900-supp2.zip › Supplementary File 2/Raw IHC/Iba1.jpeg]

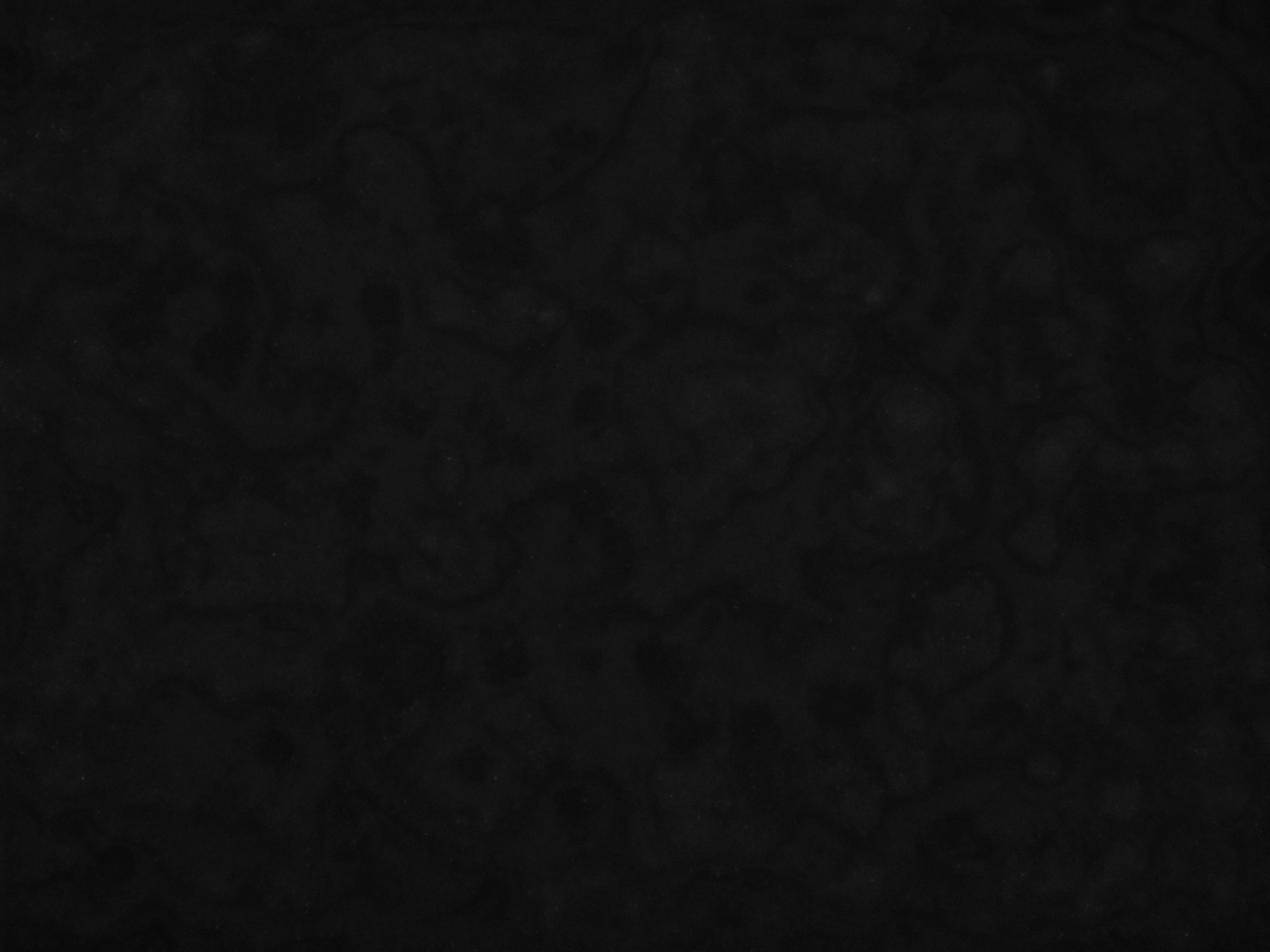

Supplement: Supplementary file 2. [file elife-102900-supp2.zip › Supplementary File 2/Raw IHC/FF_838_4 csf1r ko p2ry12.jpeg]

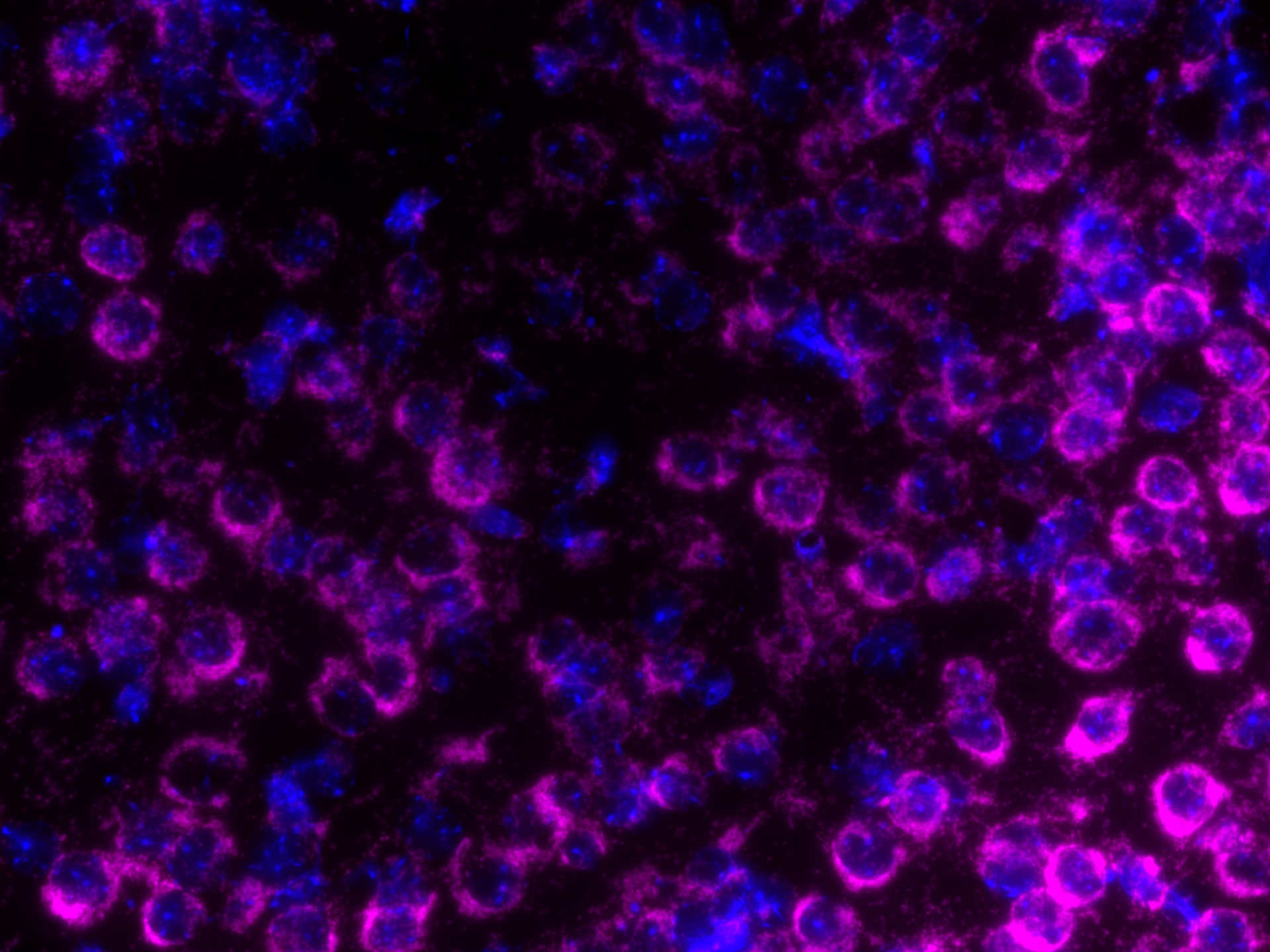

Supplement: Supplementary file 2. [file elife-102900-supp2.zip › Supplementary File 2/Raw IHC/1264 Overlay 2.jpeg]

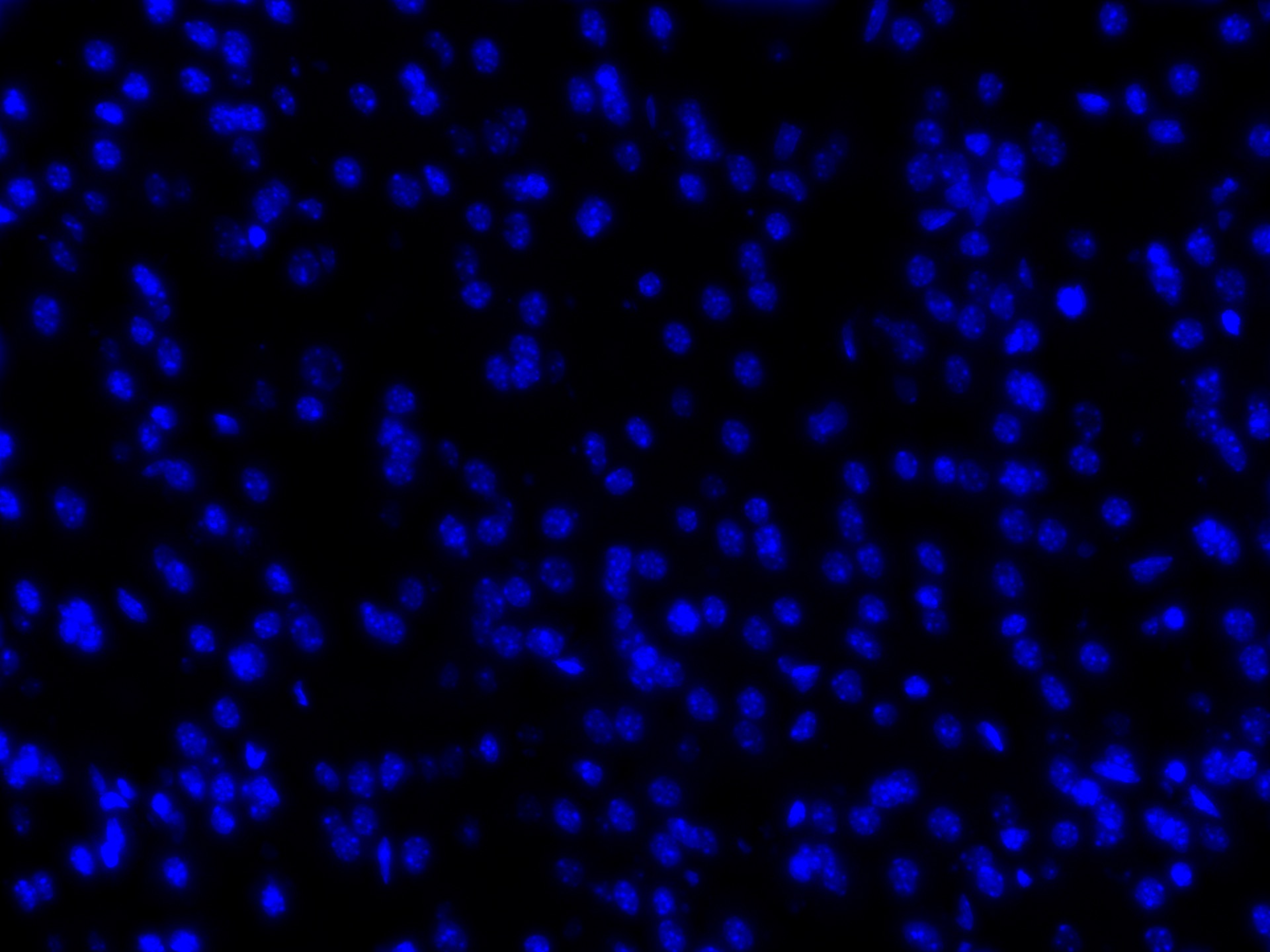

Supplement: Supplementary file 2. [file elife-102900-supp2.zip › Supplementary File 2/Raw IHC/dapi 3.jpeg]

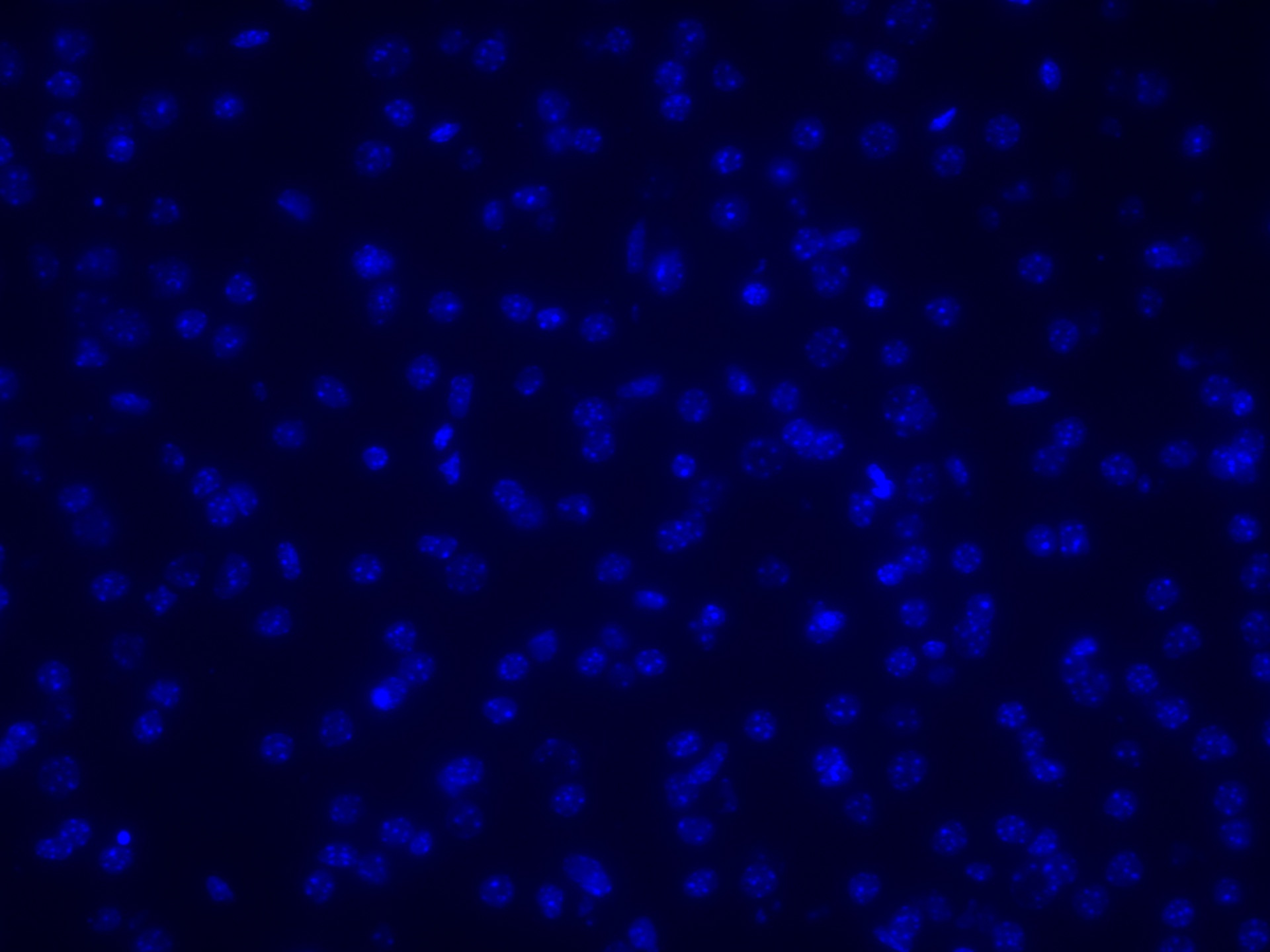

Supplement: Supplementary file 2. [file elife-102900-supp2.zip › Supplementary File 2/Raw IHC/FF_838_4 csf1r ko dapi.jpeg]

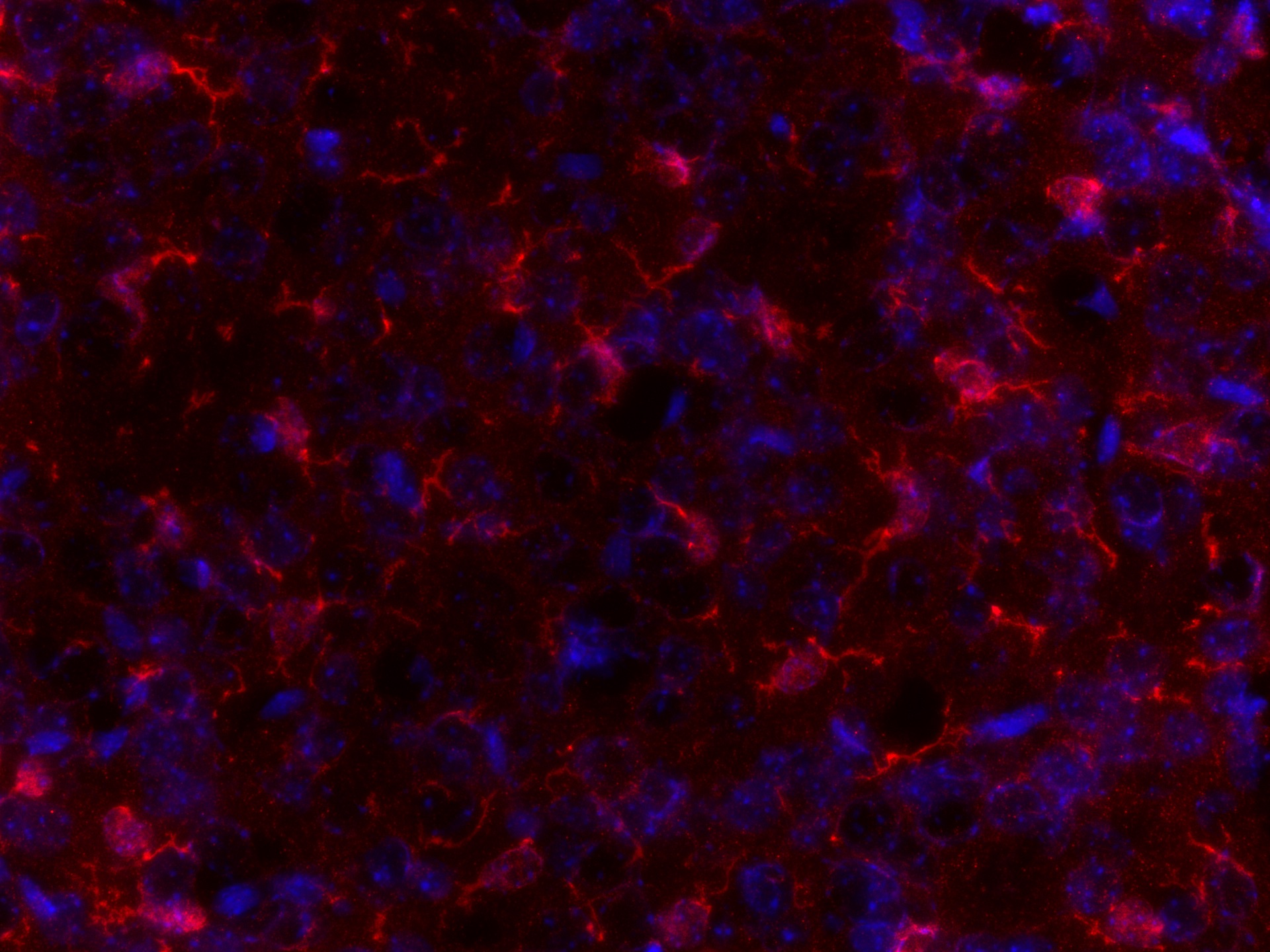

Supplement: Supplementary file 2. [file elife-102900-supp2.zip › Supplementary File 2/Raw IHC/819 Overlay.jpeg]

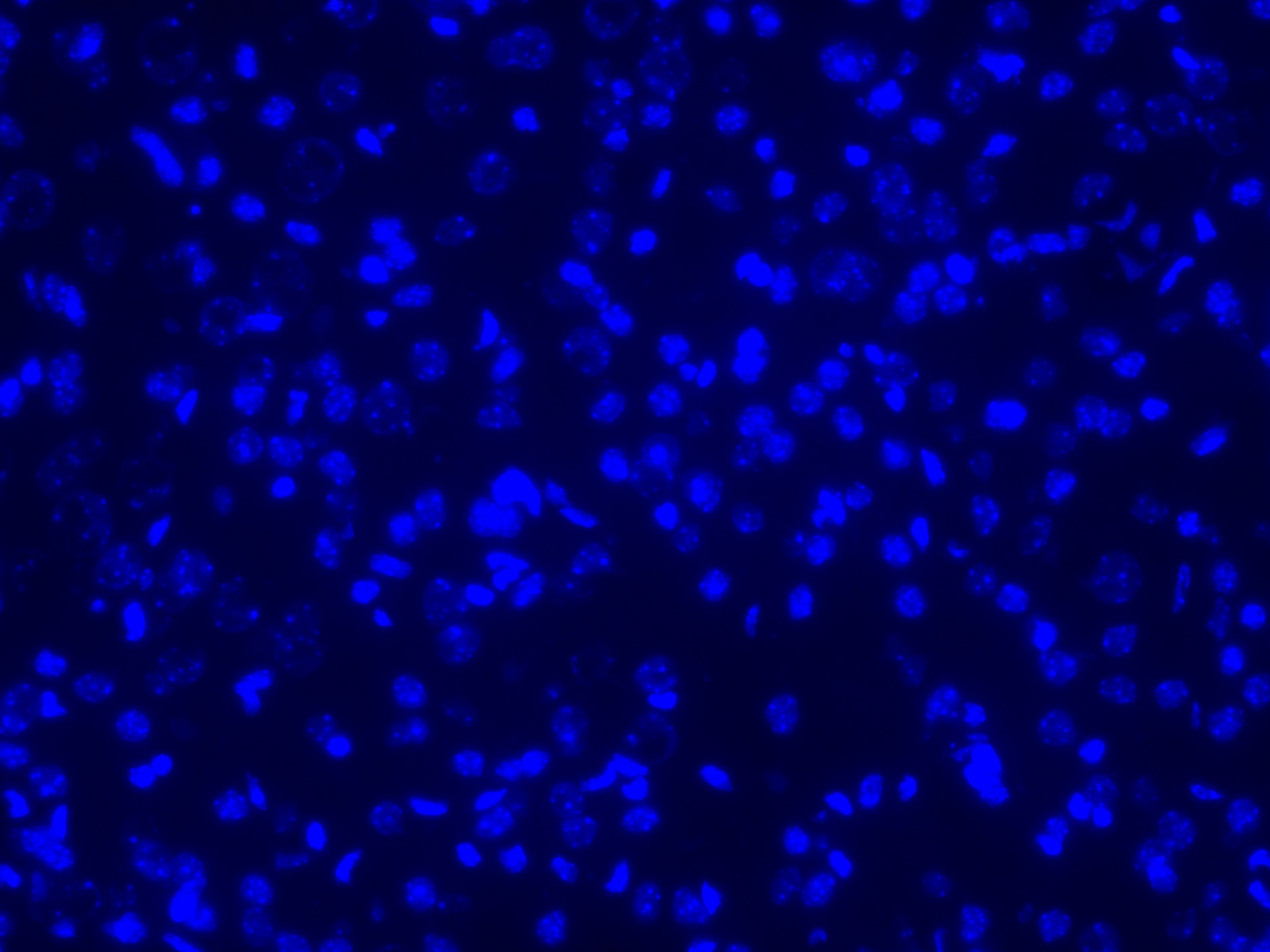

Supplement: Supplementary file 2. [file elife-102900-supp2.zip › Supplementary File 2/Raw IHC/FF_828_8 hoxb tx D8 dapi.jpeg]

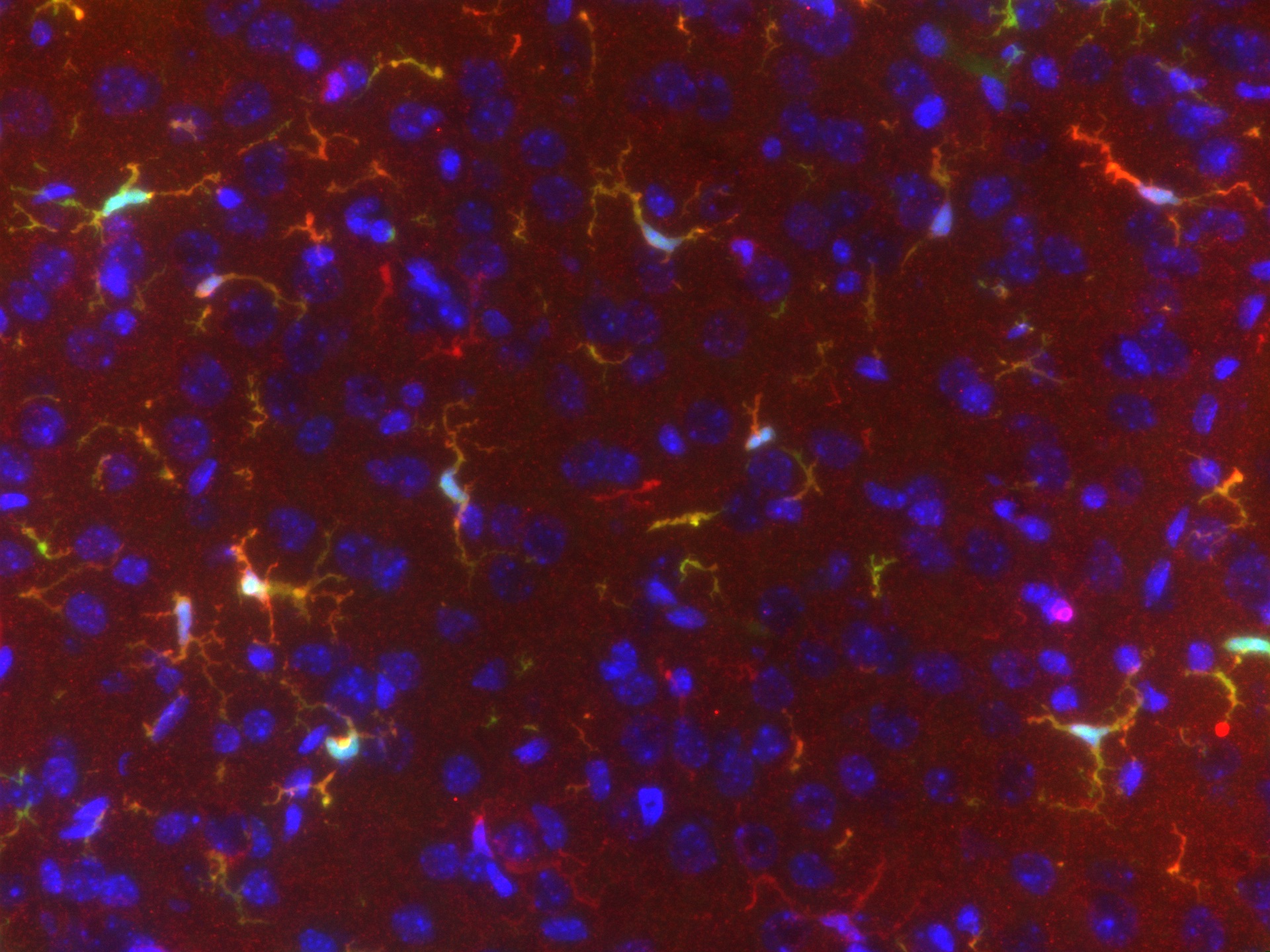

Supplement: Supplementary file 2. [file elife-102900-supp2.zip › Supplementary File 2/Raw IHC/overlay 2.jpeg]

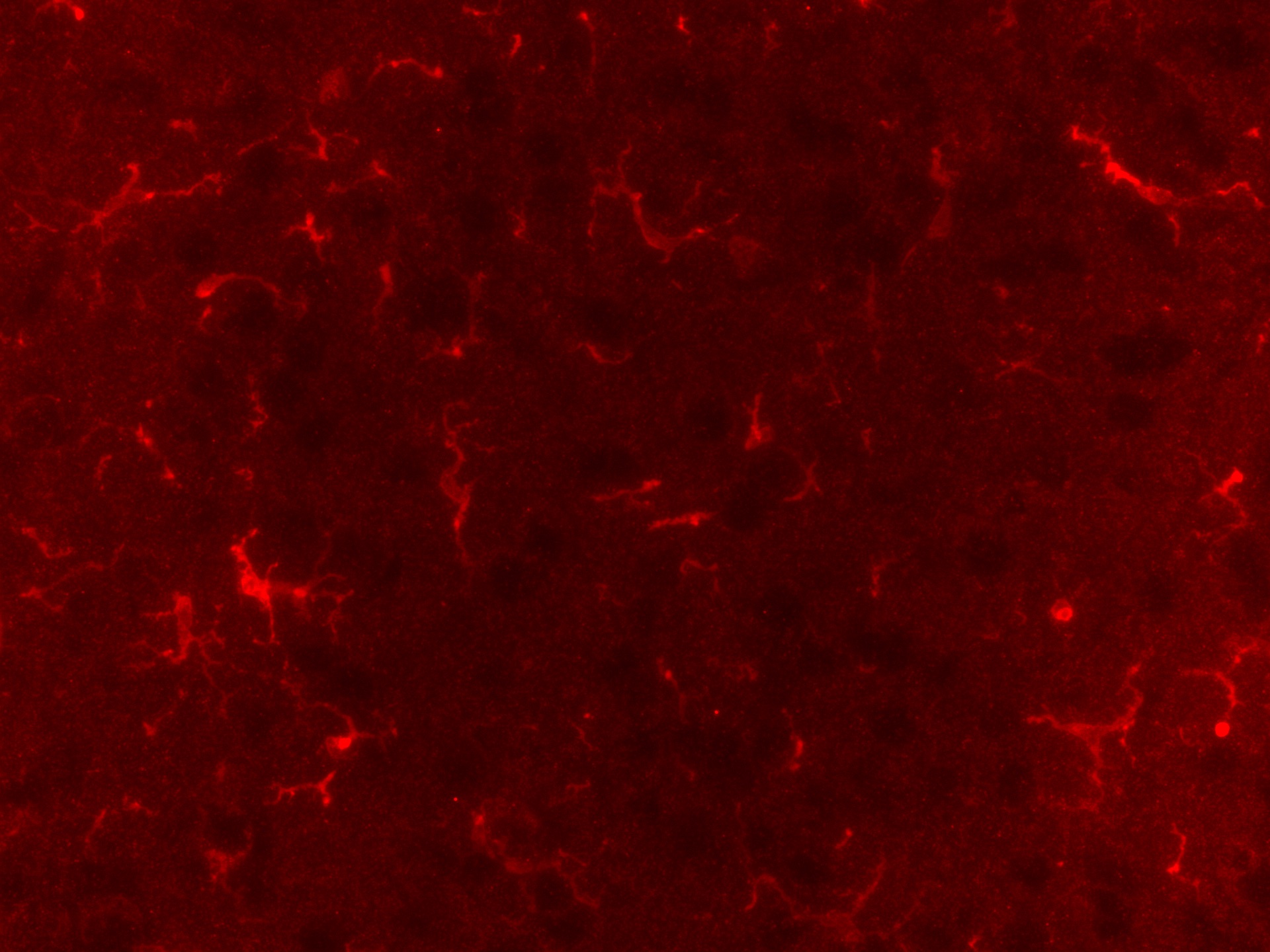

Supplement: Supplementary file 2. [file elife-102900-supp2.zip › Supplementary File 2/Raw IHC/Iba 2.jpeg]

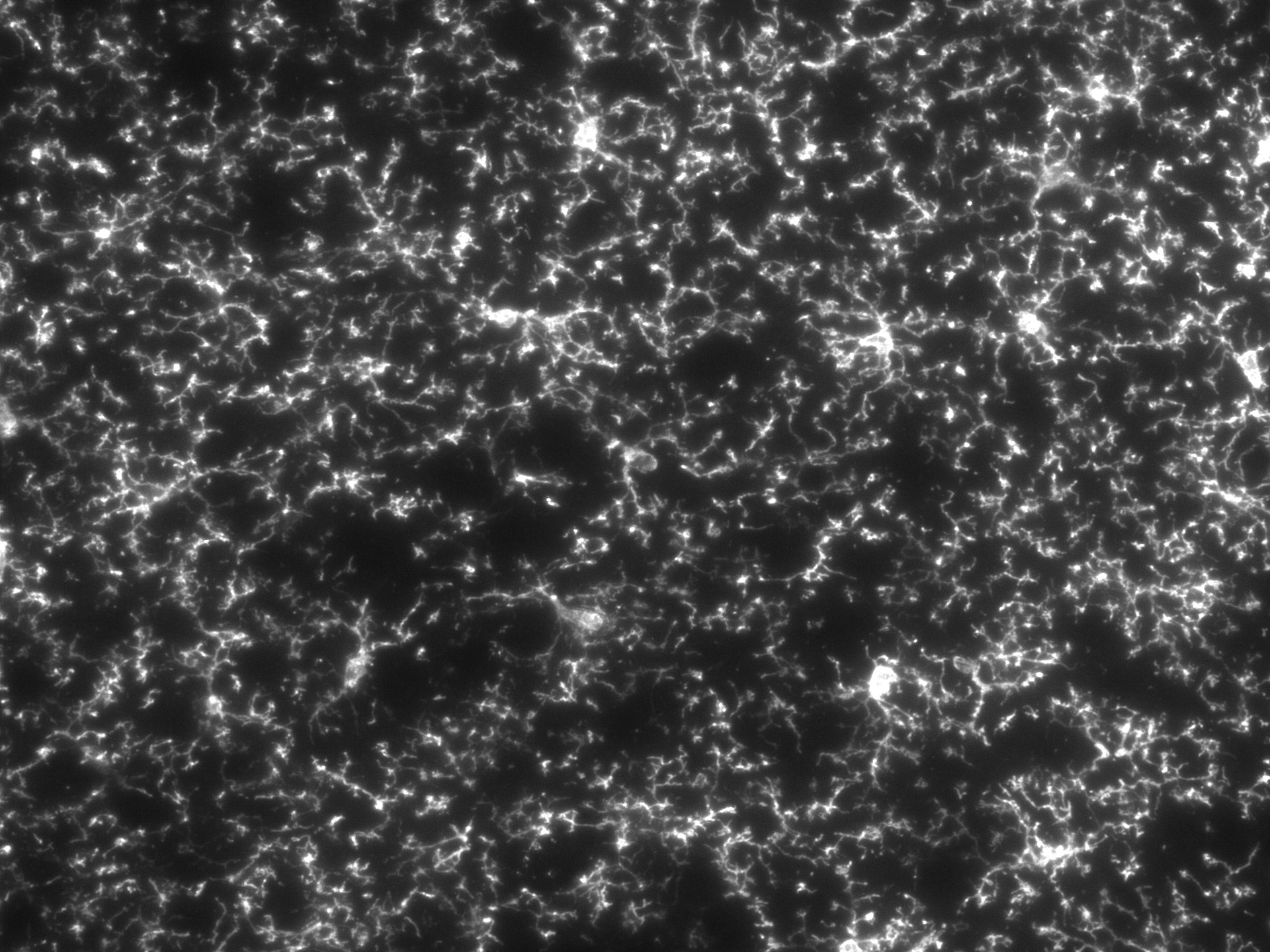

Supplement: Supplementary file 2. [file elife-102900-supp2.zip › Supplementary File 2/Raw IHC/FF_833 wt H8 02 p2ry12.jpeg]

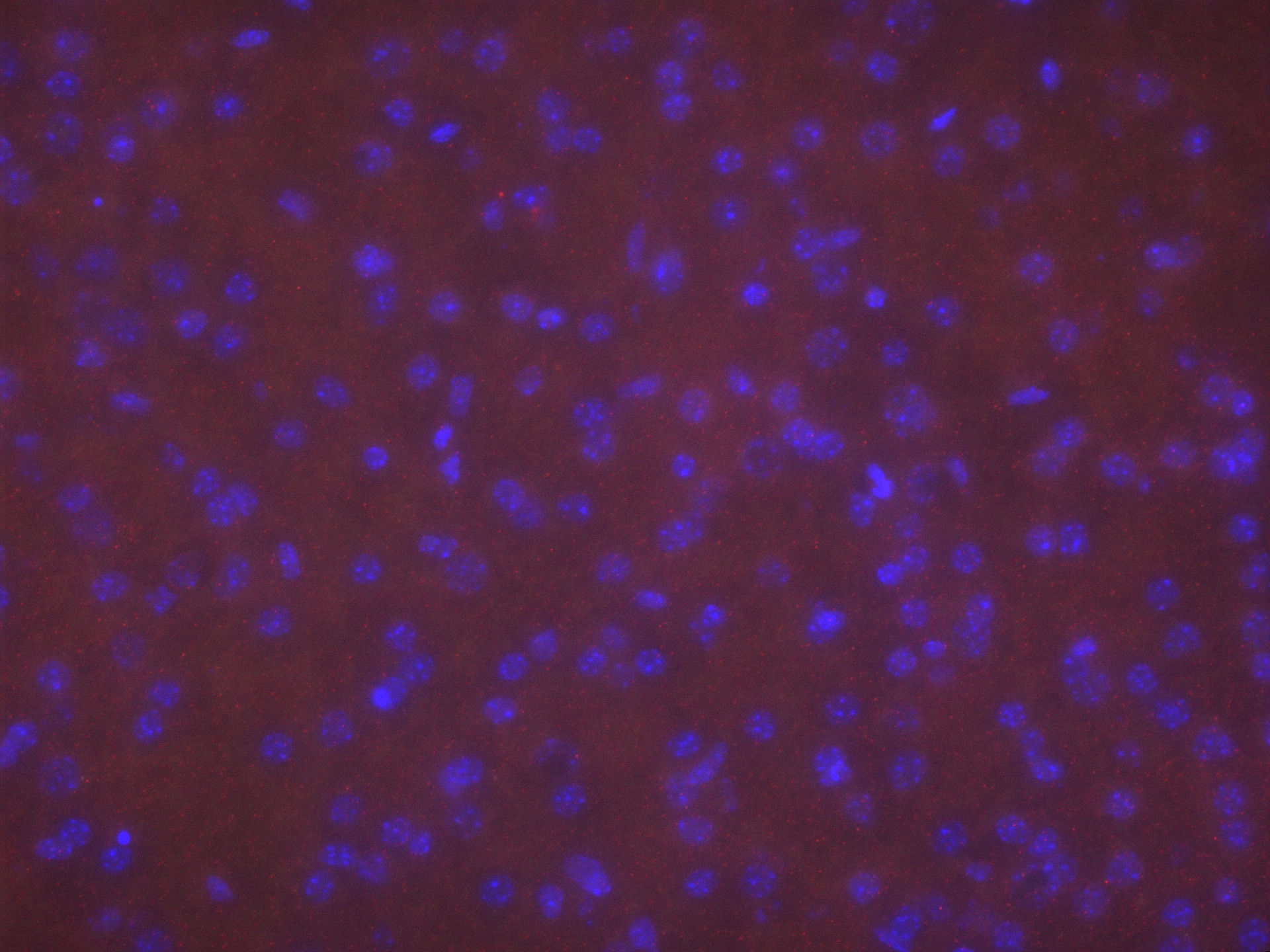

Supplement: Supplementary file 2. [file elife-102900-supp2.zip › Supplementary File 2/Raw IHC/FF_838_4 csf1r ko overlay.jpeg]

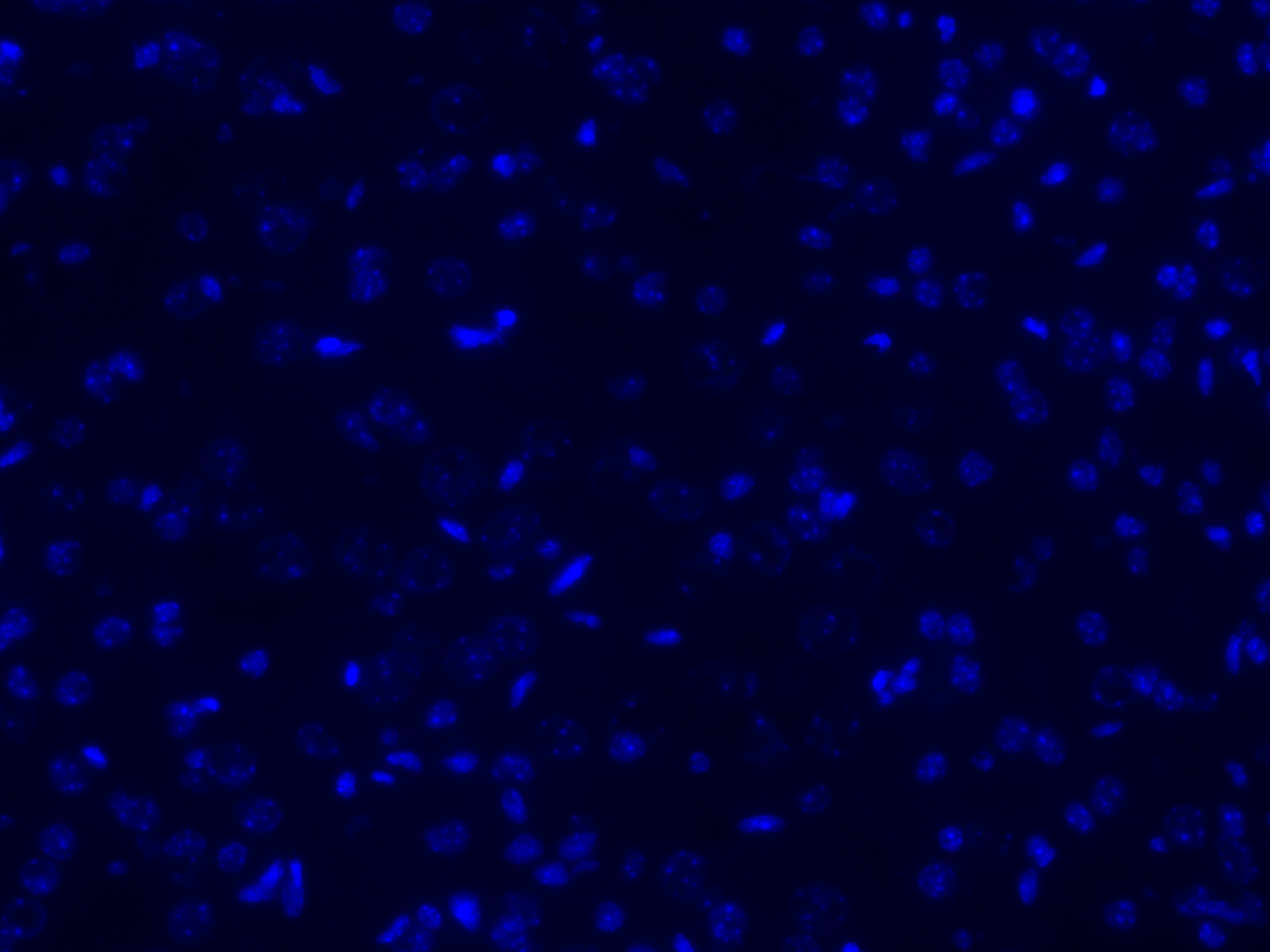

Supplement: Supplementary file 2. [file elife-102900-supp2.zip › Supplementary File 2/Raw IHC/FF_833 wt H8 02 dapi.jpeg]

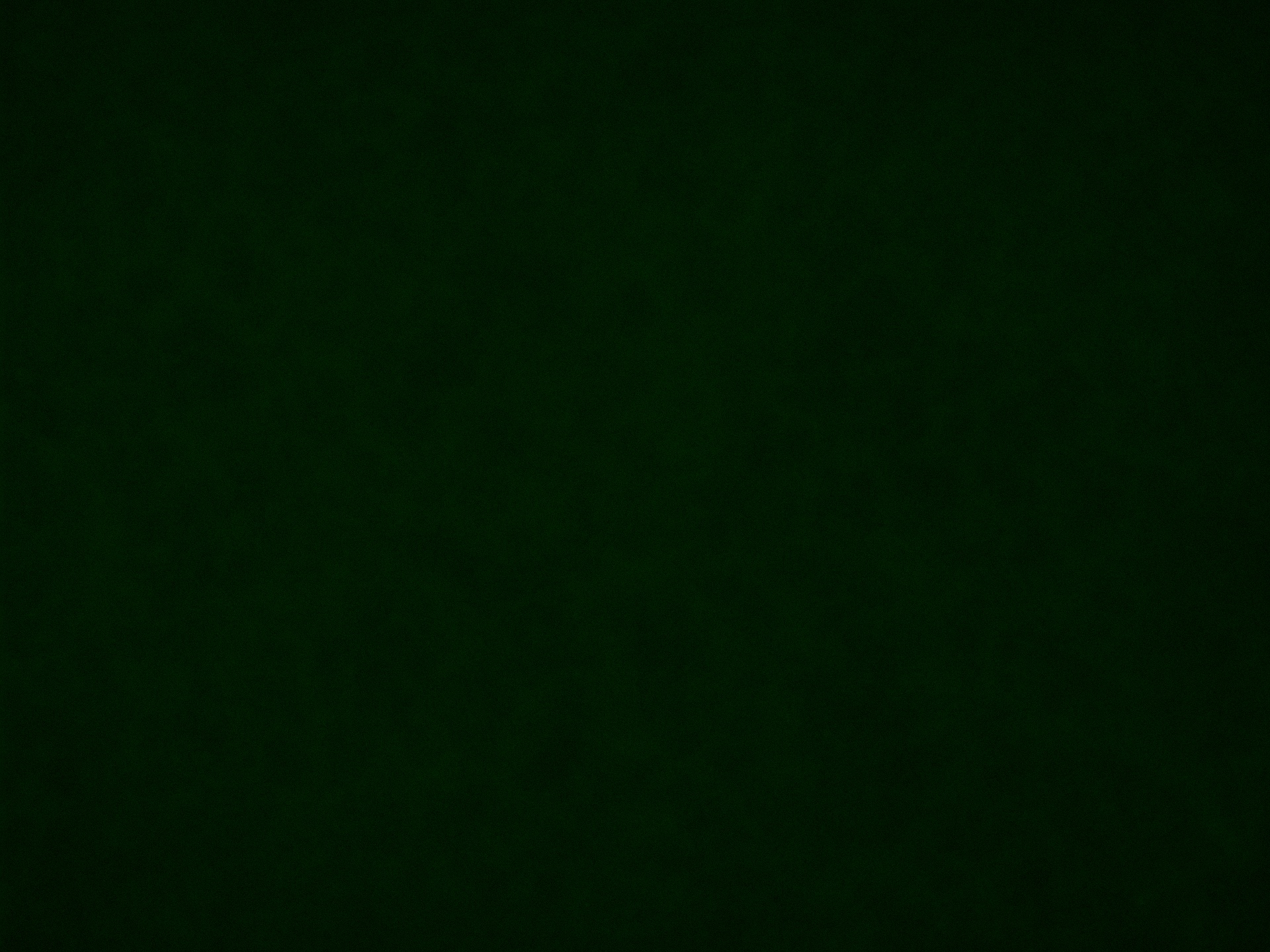

Supplement: Supplementary file 2. [file elife-102900-supp2.zip › Supplementary File 2/Raw IHC/FF_838_4 csf1r ko gfp.jpeg]

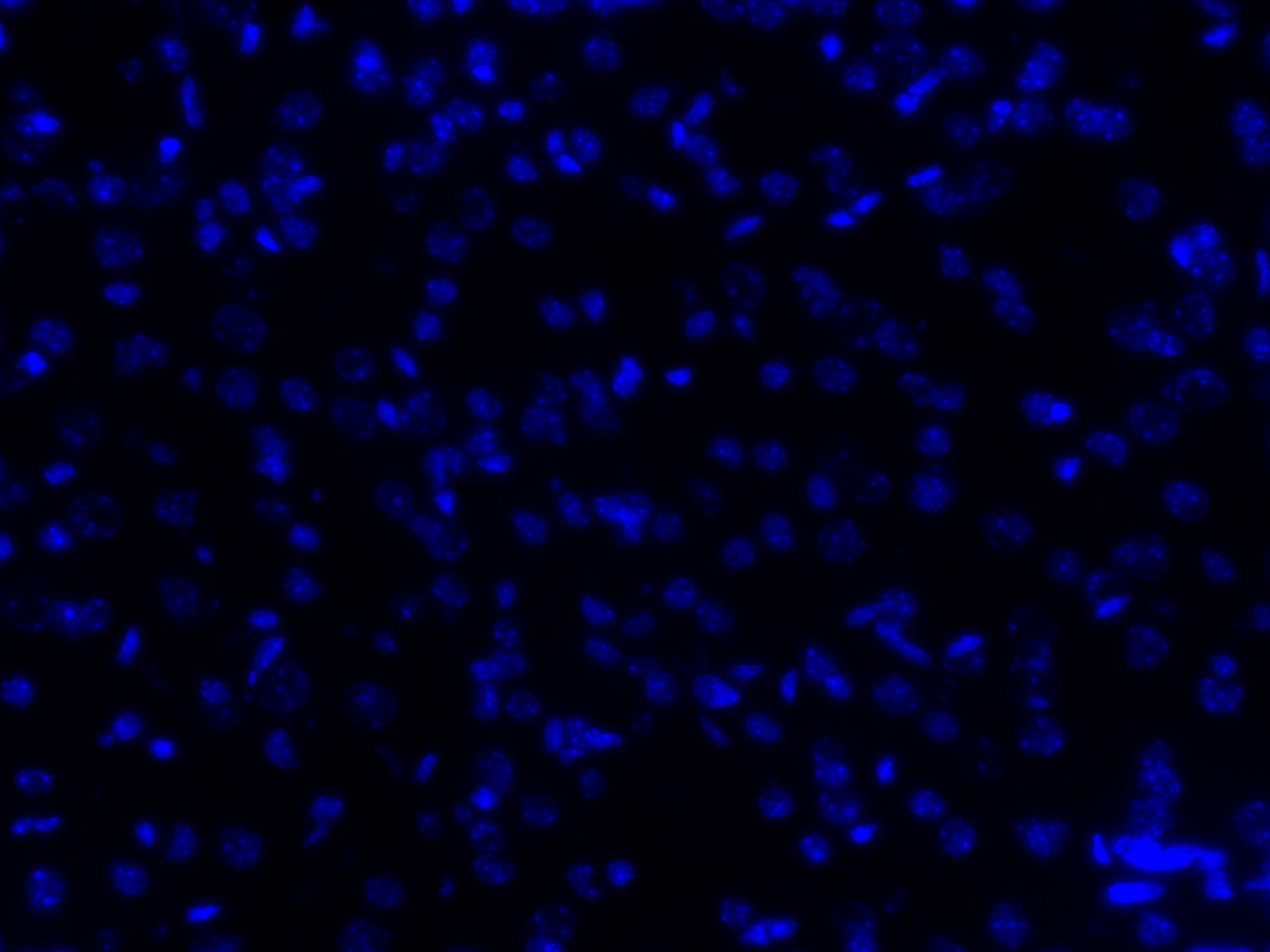

Supplement: Supplementary file 2. [file elife-102900-supp2.zip › Supplementary File 2/Raw IHC/DAPI 4.jpeg]

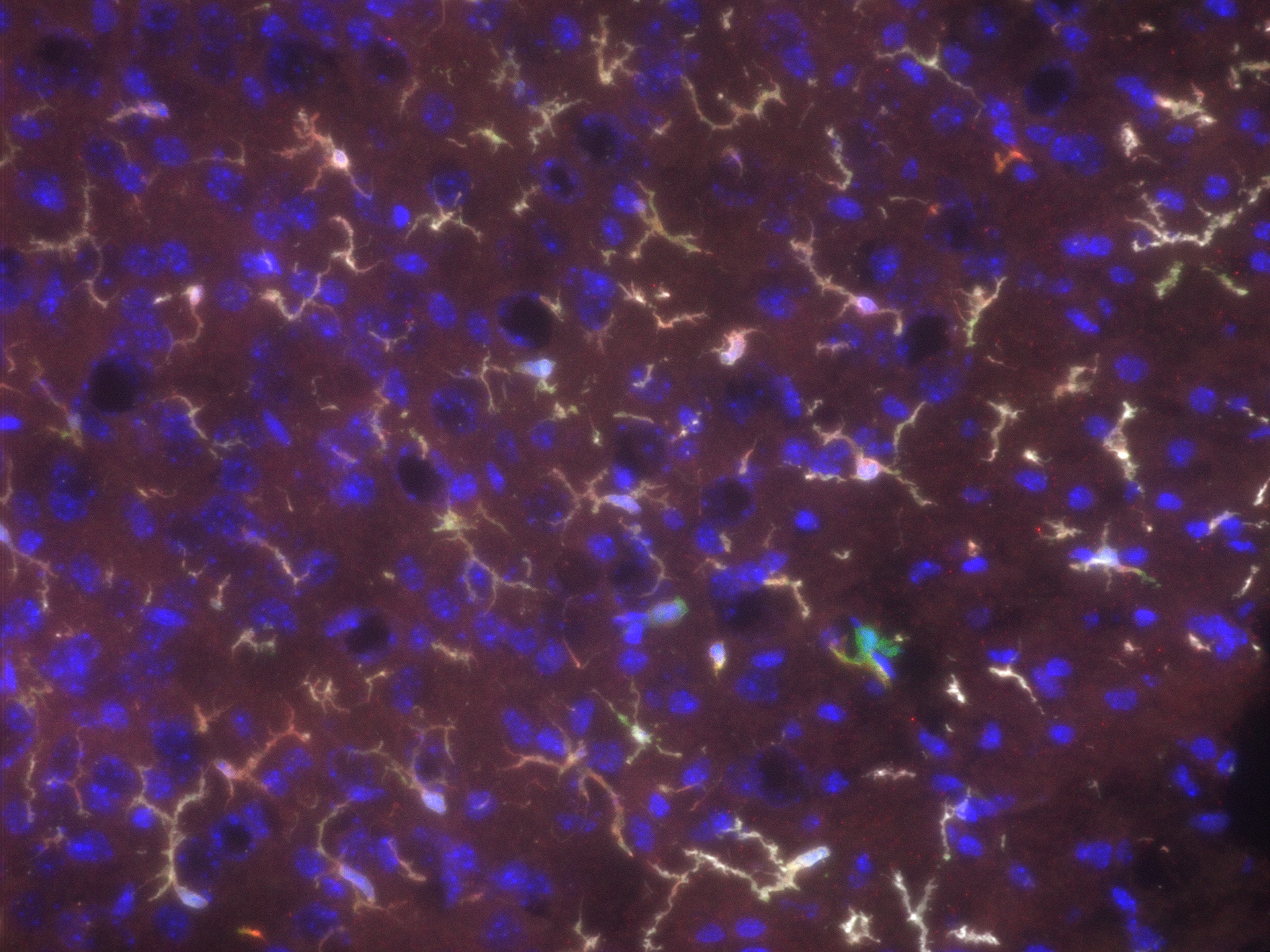

Supplement: Supplementary file 2. [file elife-102900-supp2.zip › Supplementary File 2/Raw IHC/FF_748_5 bm tx H top 8 overlay.jpeg]

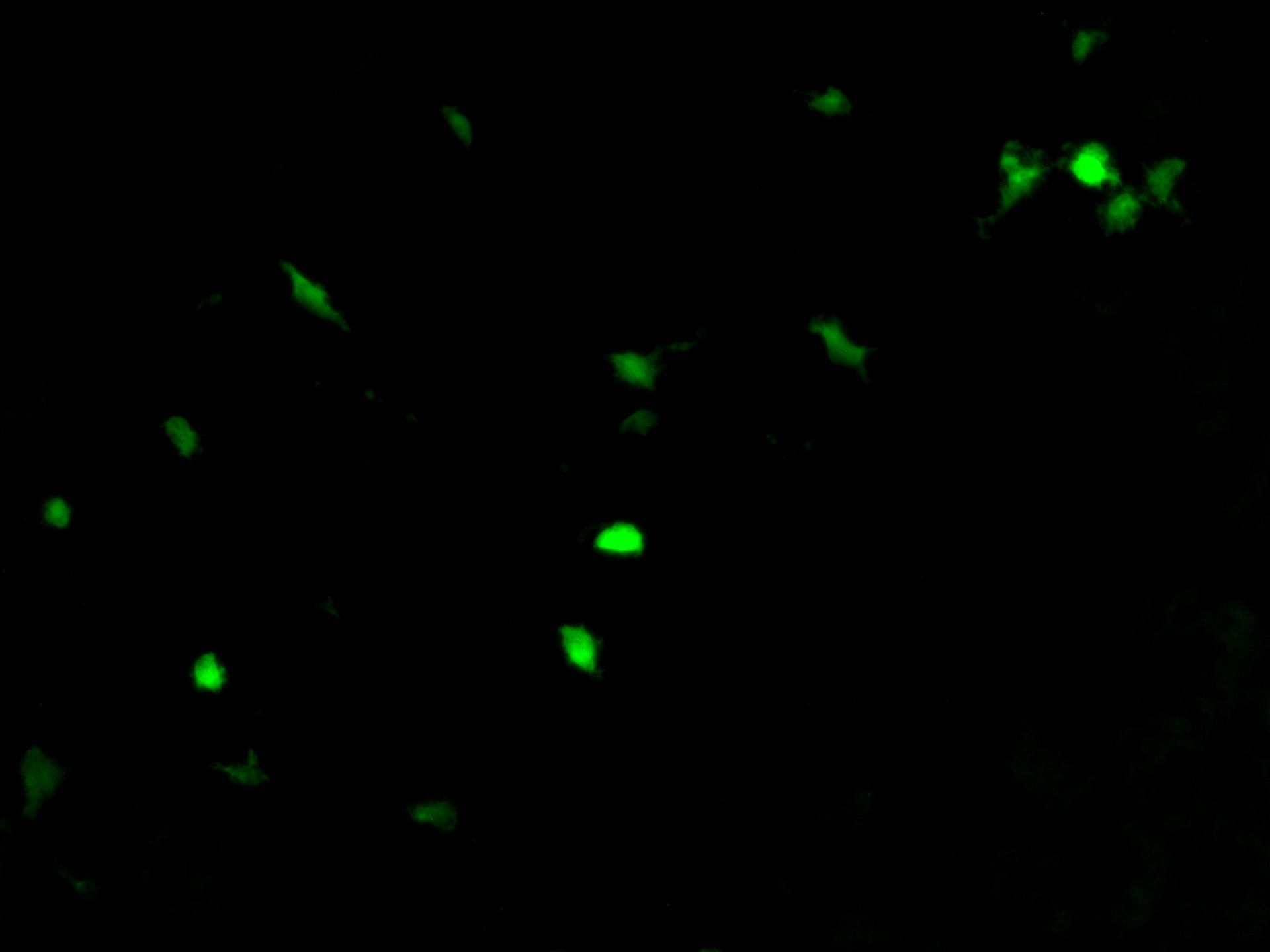

Supplement: Supplementary file 2. [file elife-102900-supp2.zip › Supplementary File 2/Raw IHC/1224 Z GFP.jpeg]

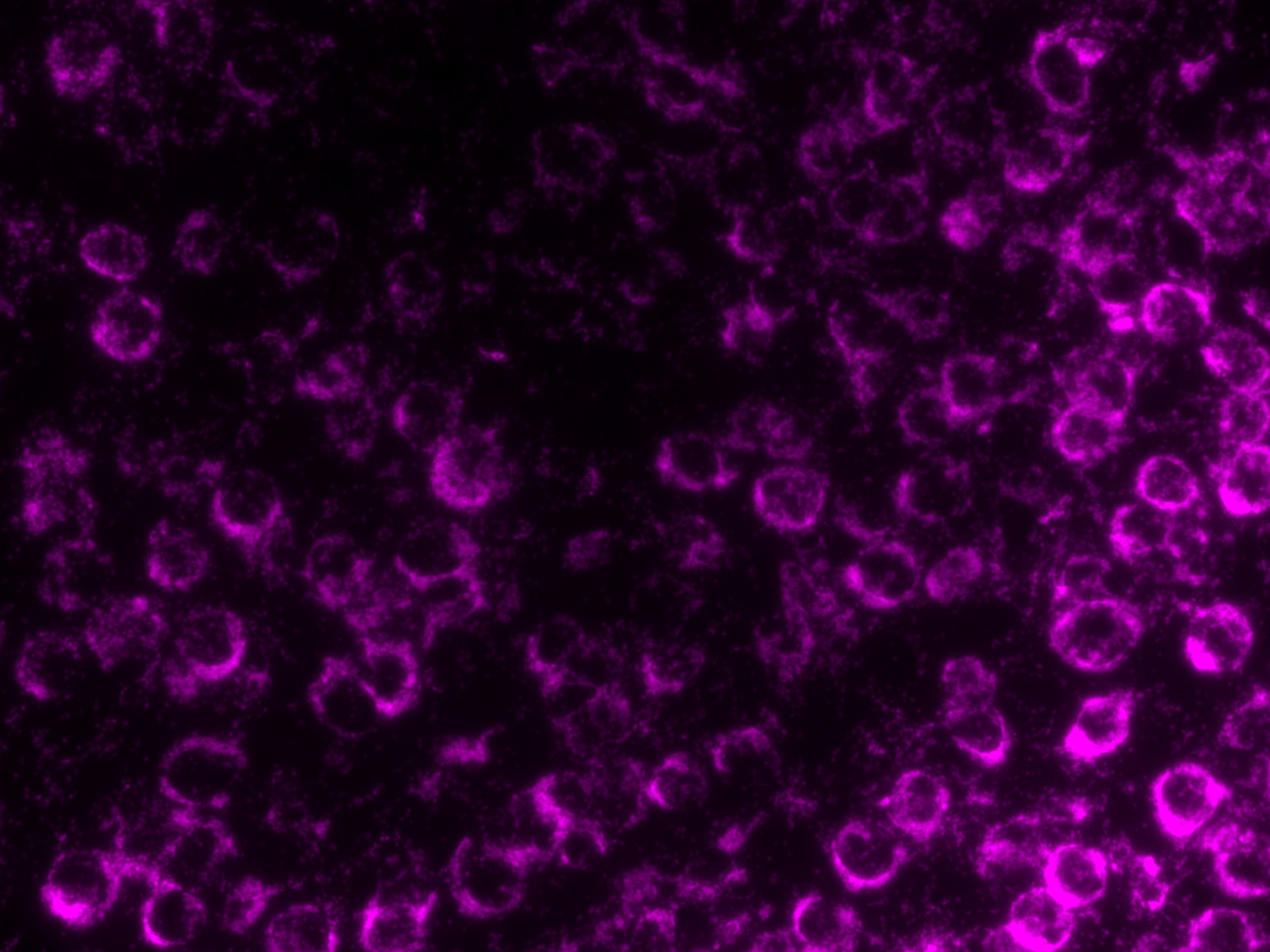

Supplement: Supplementary file 2. [file elife-102900-supp2.zip › Supplementary File 2/Raw IHC/1264 Isg.jpeg]

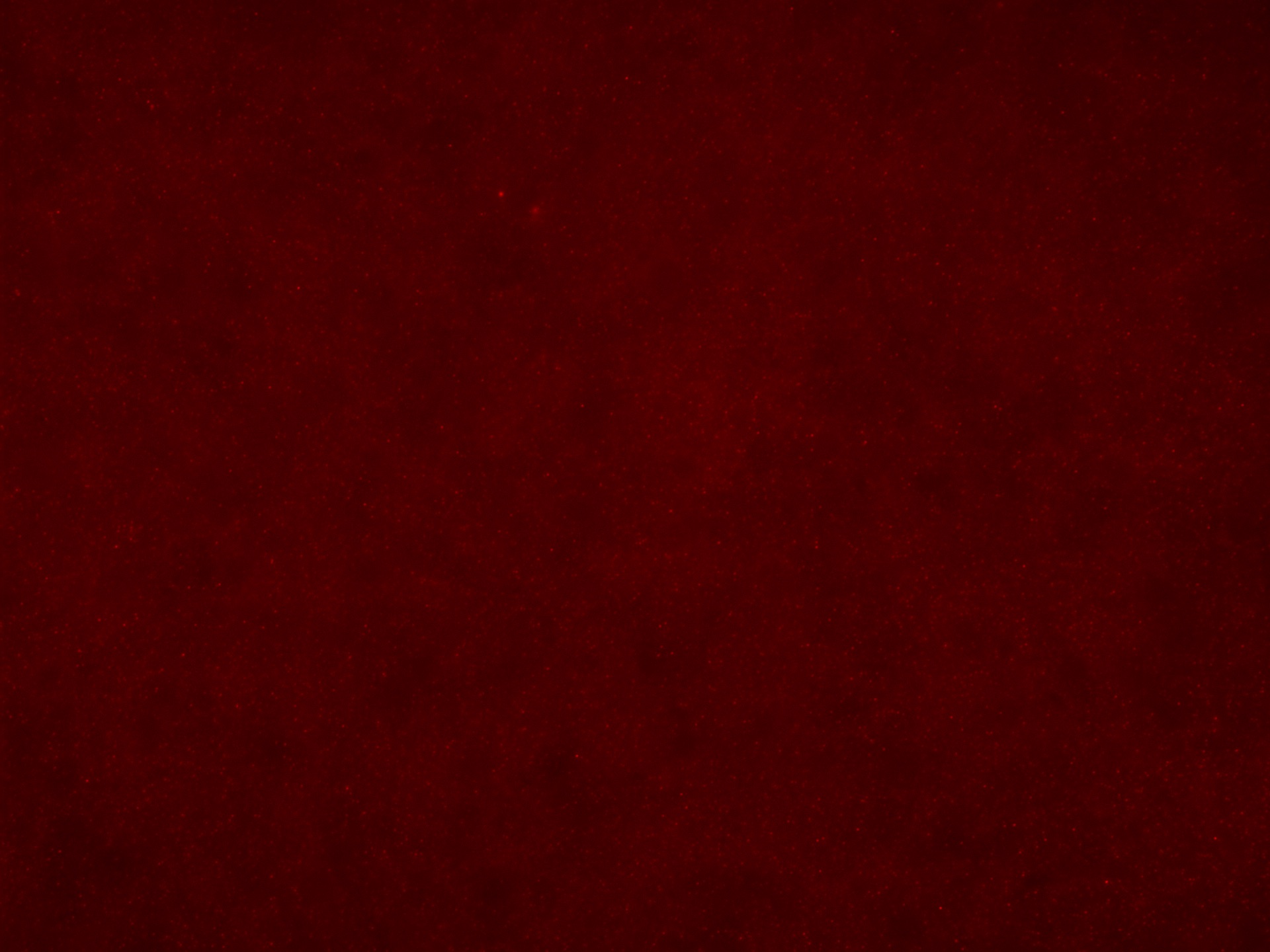

Supplement: Supplementary file 2. [file elife-102900-supp2.zip › Supplementary File 2/Raw IHC/FF_838_4 csf1r ko iba1.jpeg]

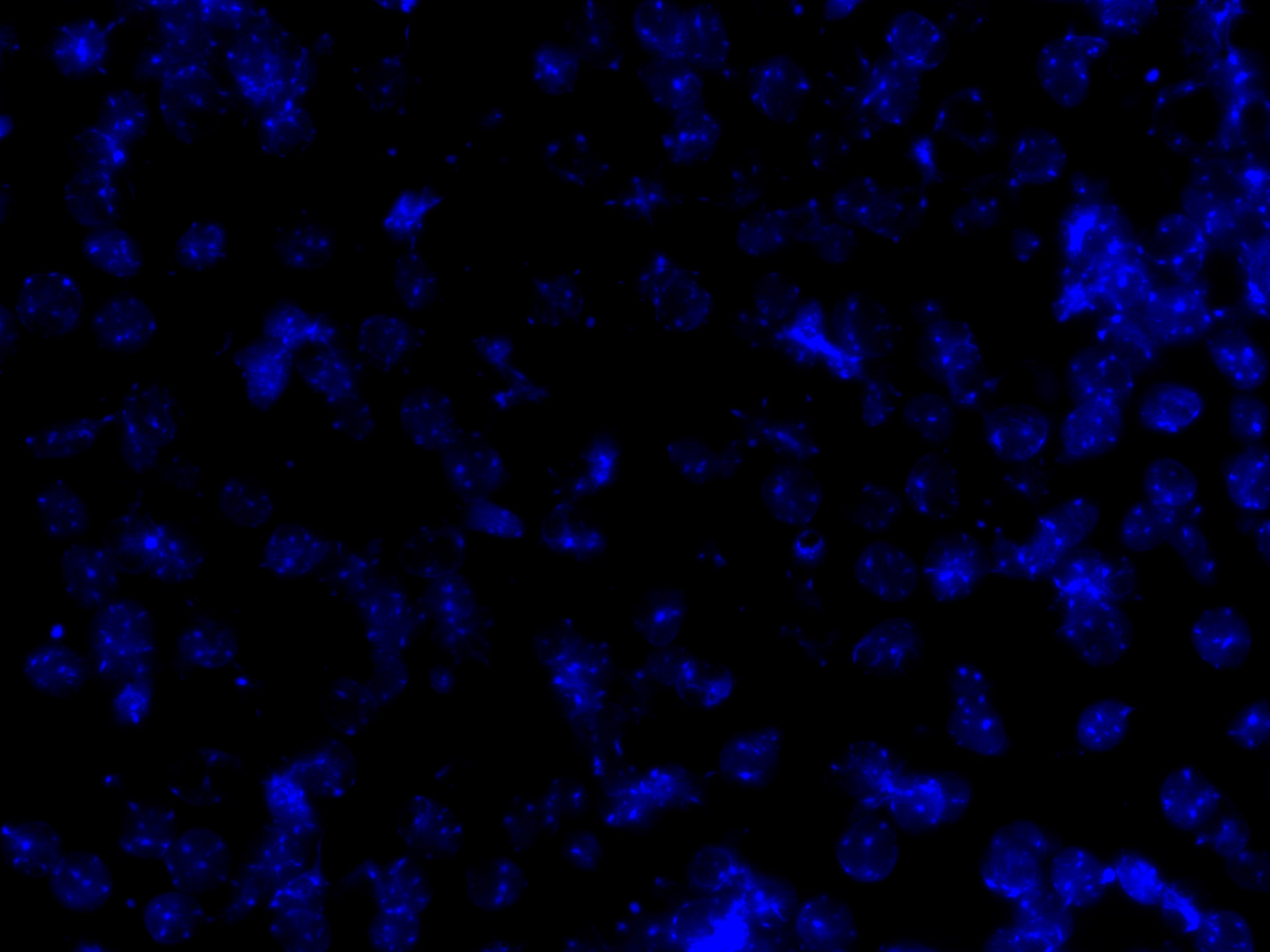

Supplement: Supplementary file 2. [file elife-102900-supp2.zip › Supplementary File 2/Raw IHC/1264 DAPI.jpeg]

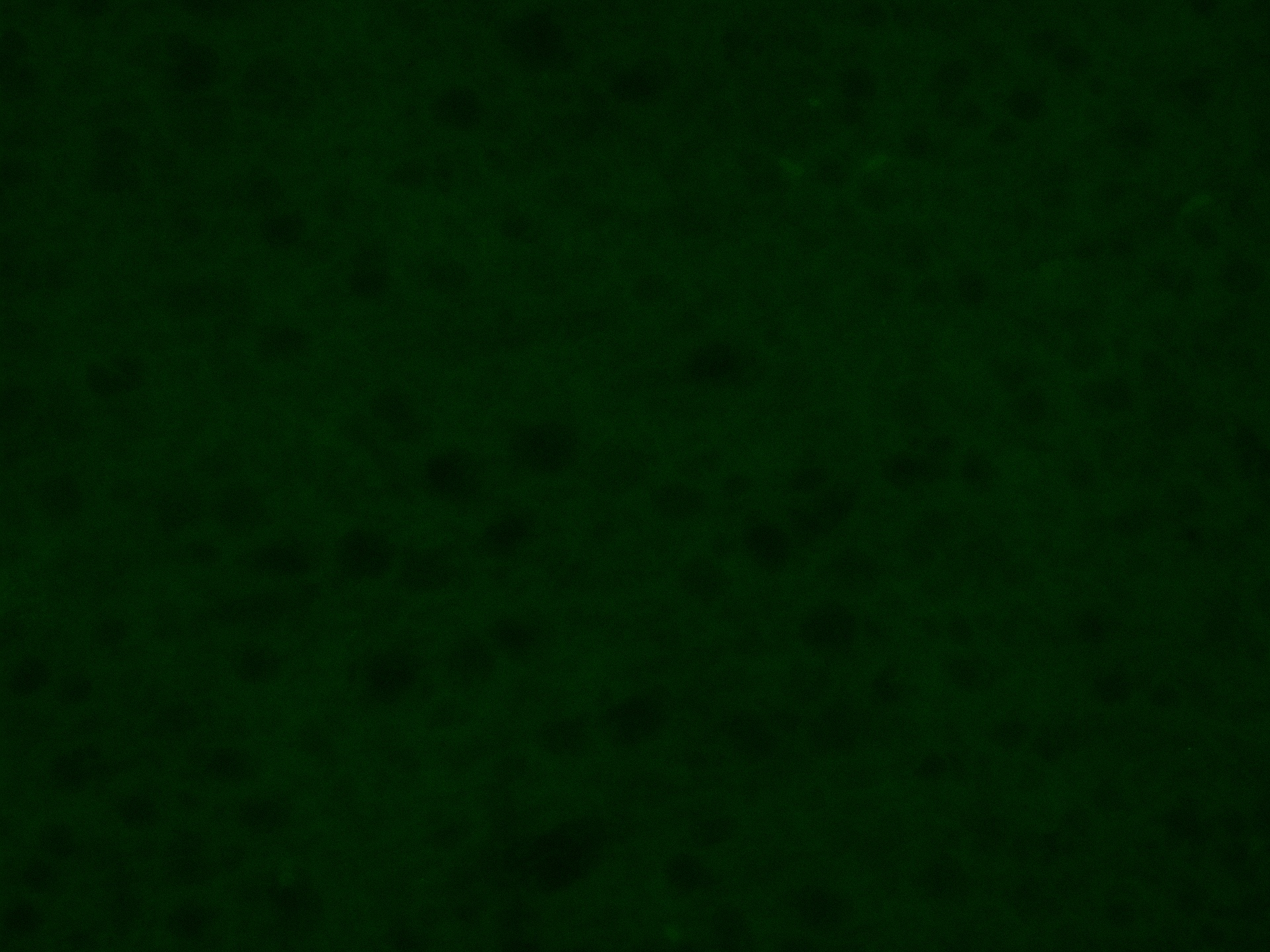

Supplement: Supplementary file 2. [file elife-102900-supp2.zip › Supplementary File 2/Raw IHC/FF_833 wt H8 02 gfp.jpeg]

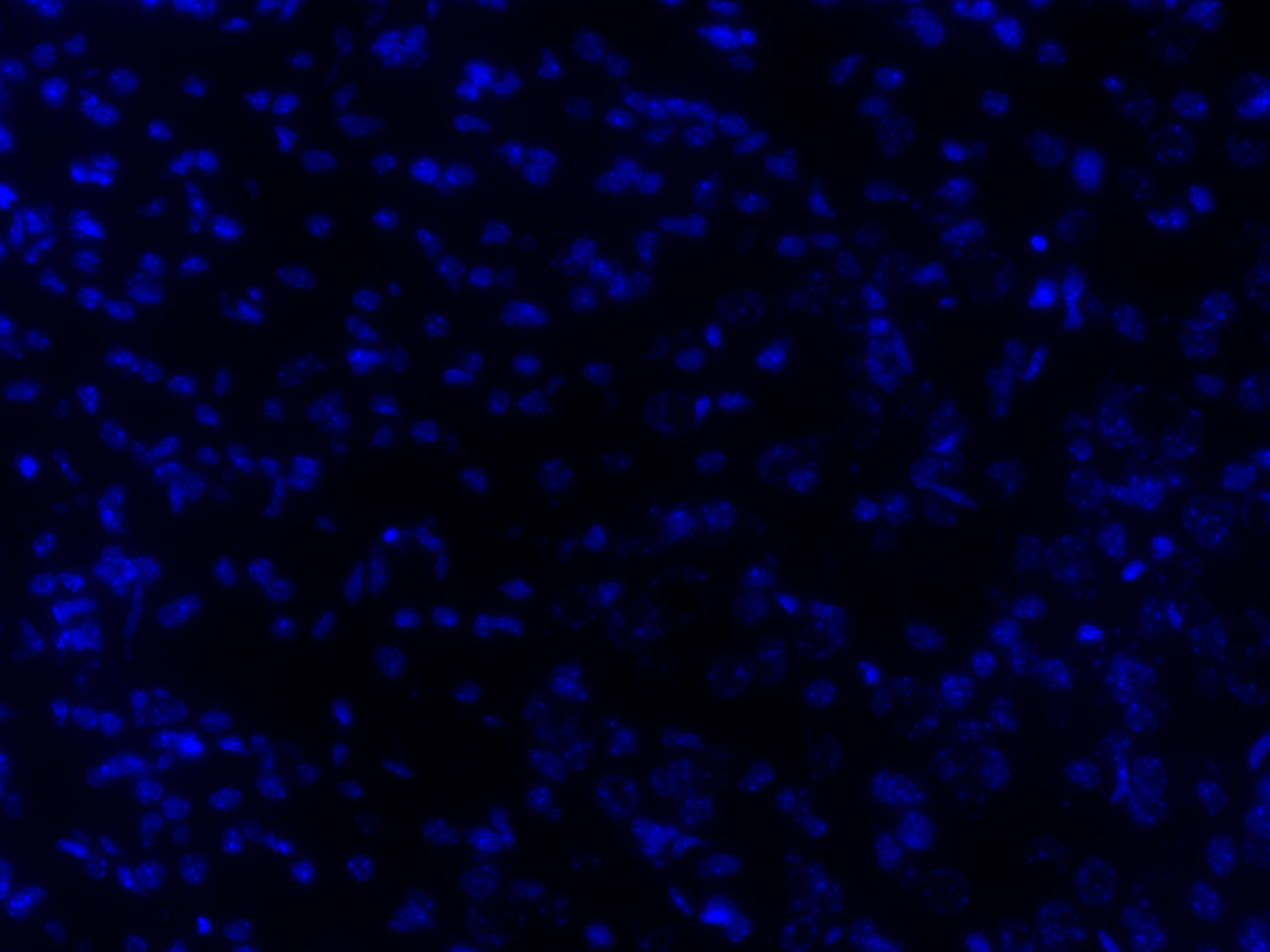

Supplement: Supplementary file 2. [file elife-102900-supp2.zip › Supplementary File 2/Raw IHC/Dapi.jpeg]

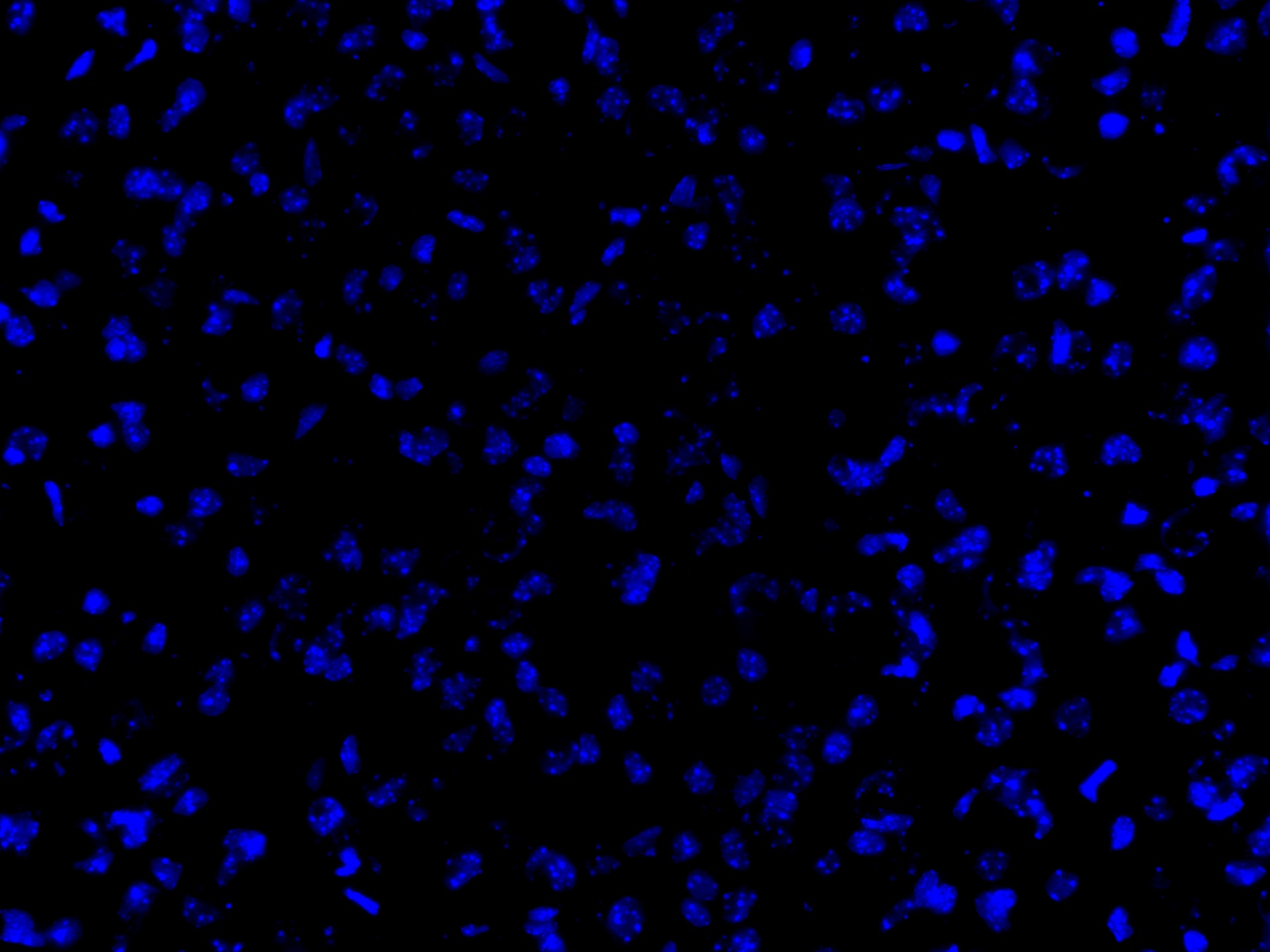

Supplement: Supplementary file 2. [file elife-102900-supp2.zip › Supplementary File 2/Raw IHC/1076 Z DAPI.jpeg]

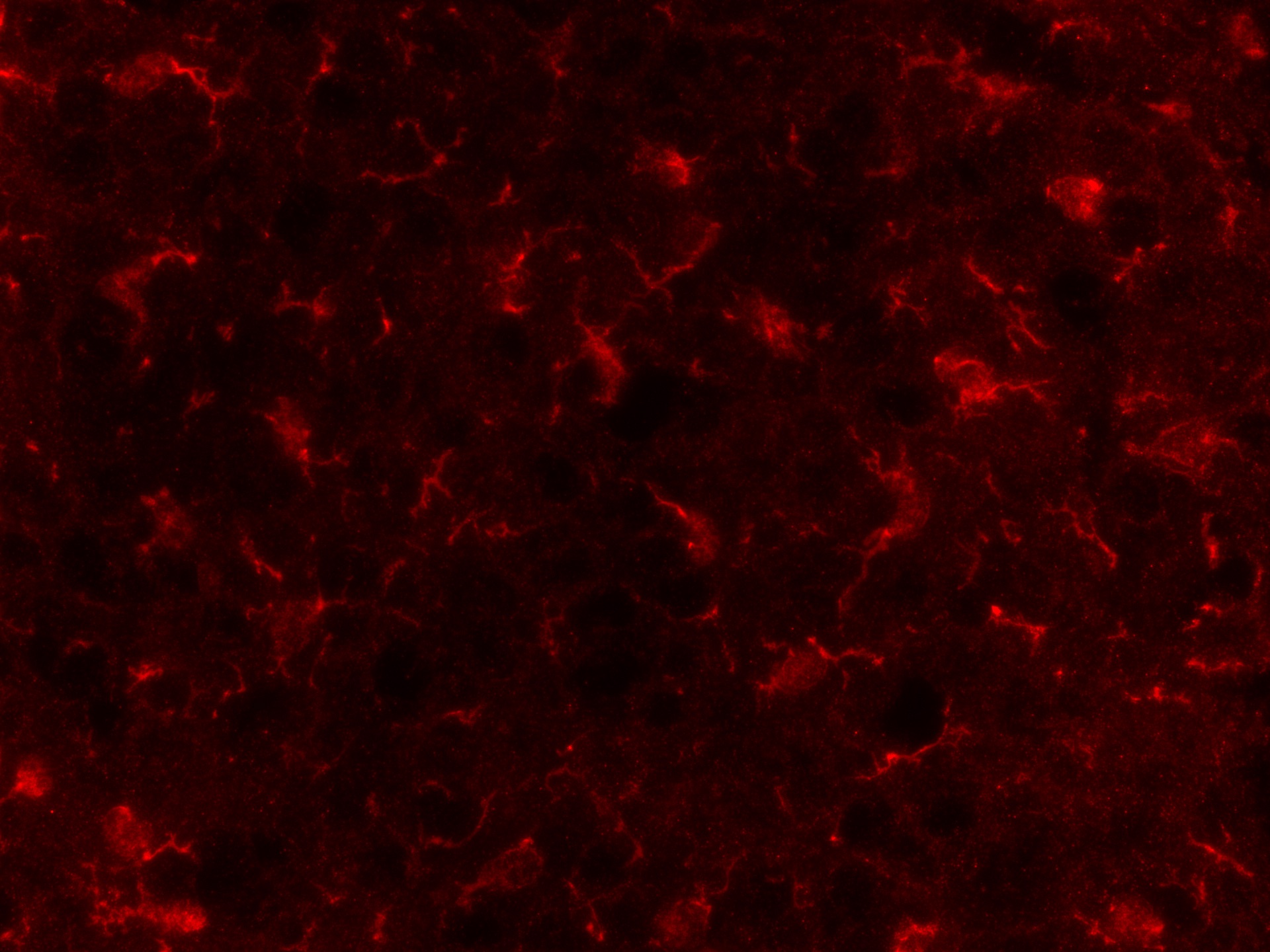

Supplement: Supplementary file 2. [file elife-102900-supp2.zip › Supplementary File 2/Raw IHC/819 Iba.jpeg]

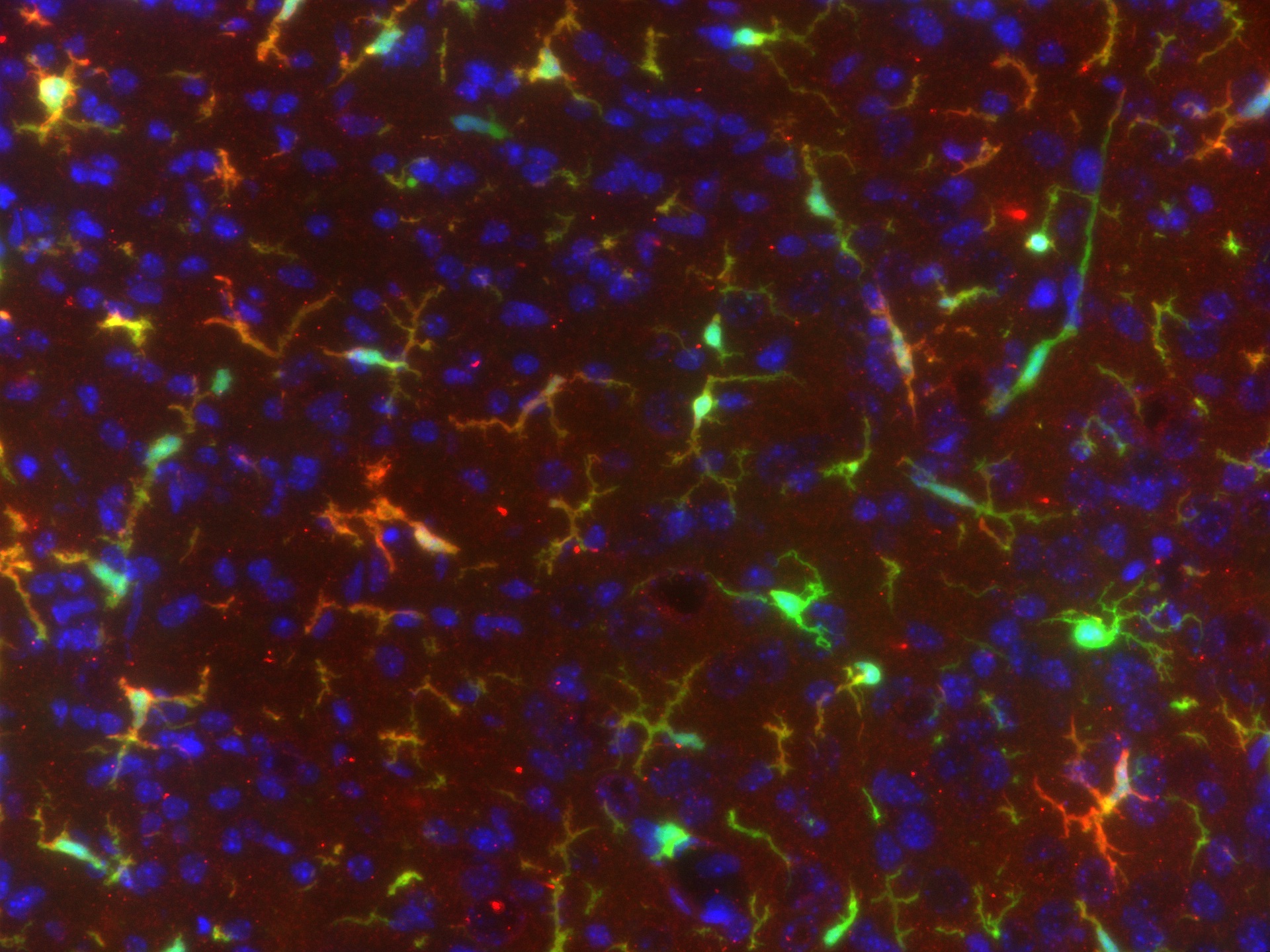

Supplement: Supplementary file 2. [file elife-102900-supp2.zip › Supplementary File 2/Raw IHC/Overlay.jpeg]

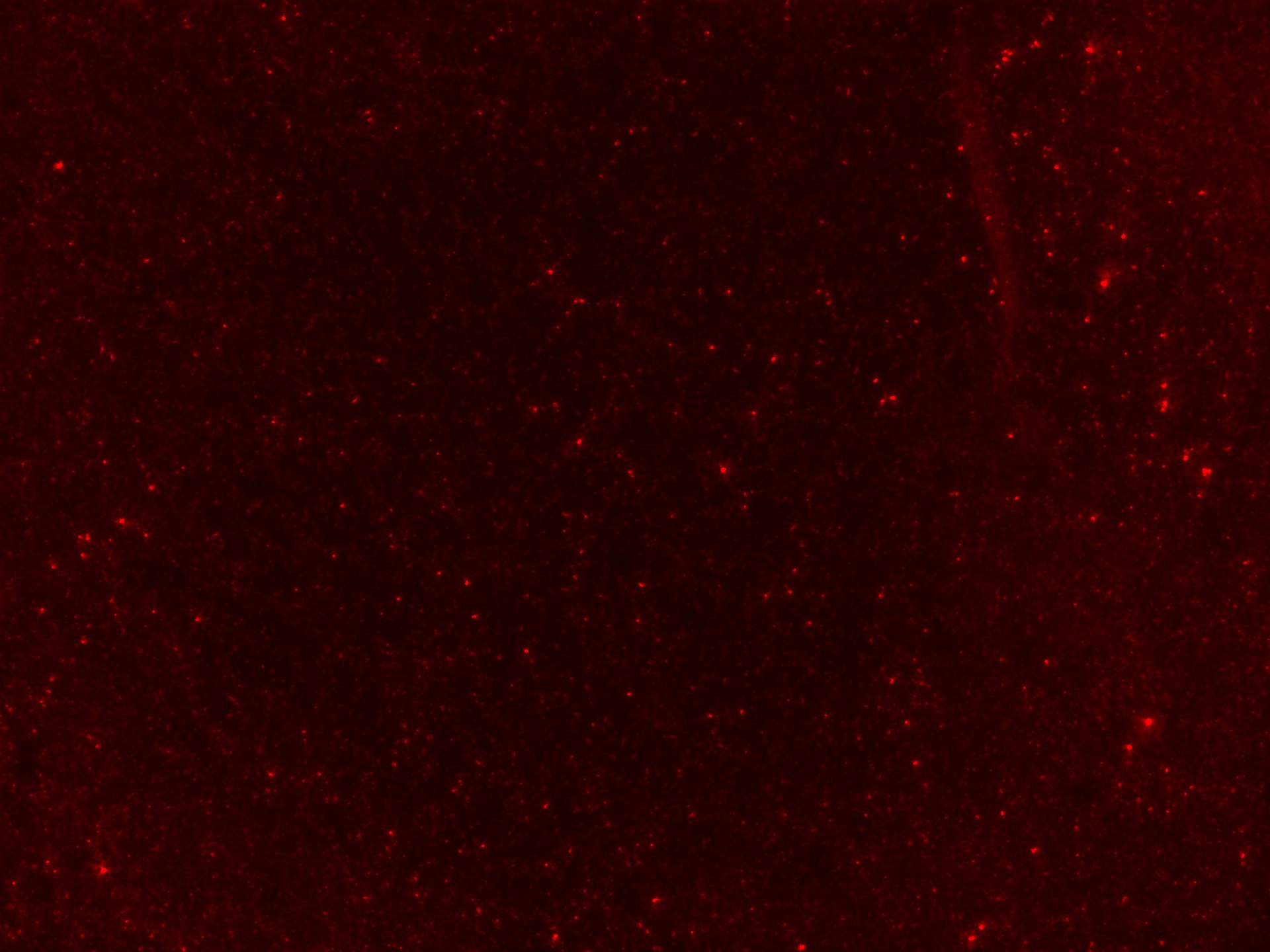

Supplement: Supplementary file 2. [file elife-102900-supp2.zip › Supplementary File 2/Raw IHC/iba 3.jpeg]

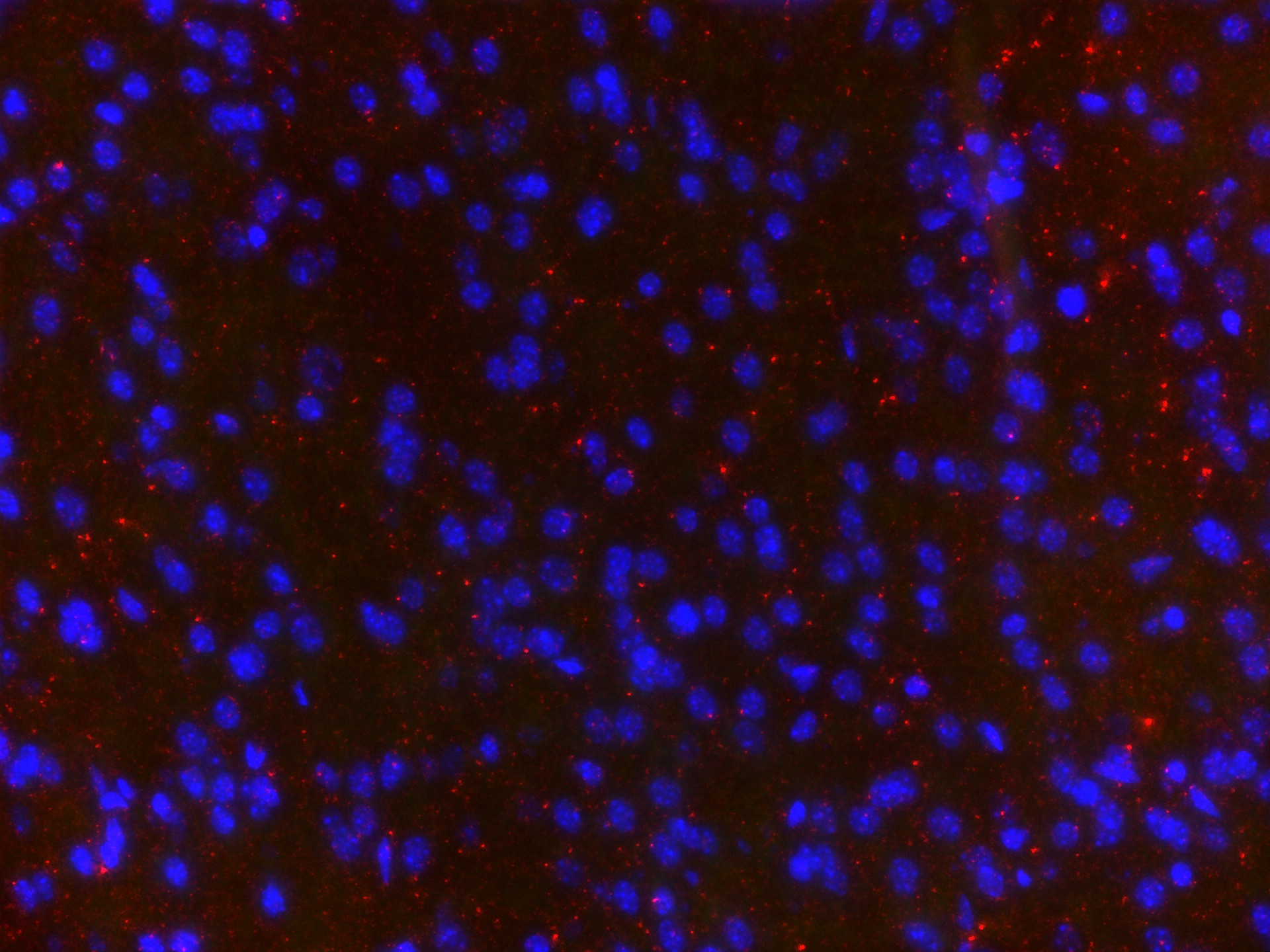

Supplement: Supplementary file 2. [file elife-102900-supp2.zip › Supplementary File 2/Raw IHC/overlay 3.jpeg]

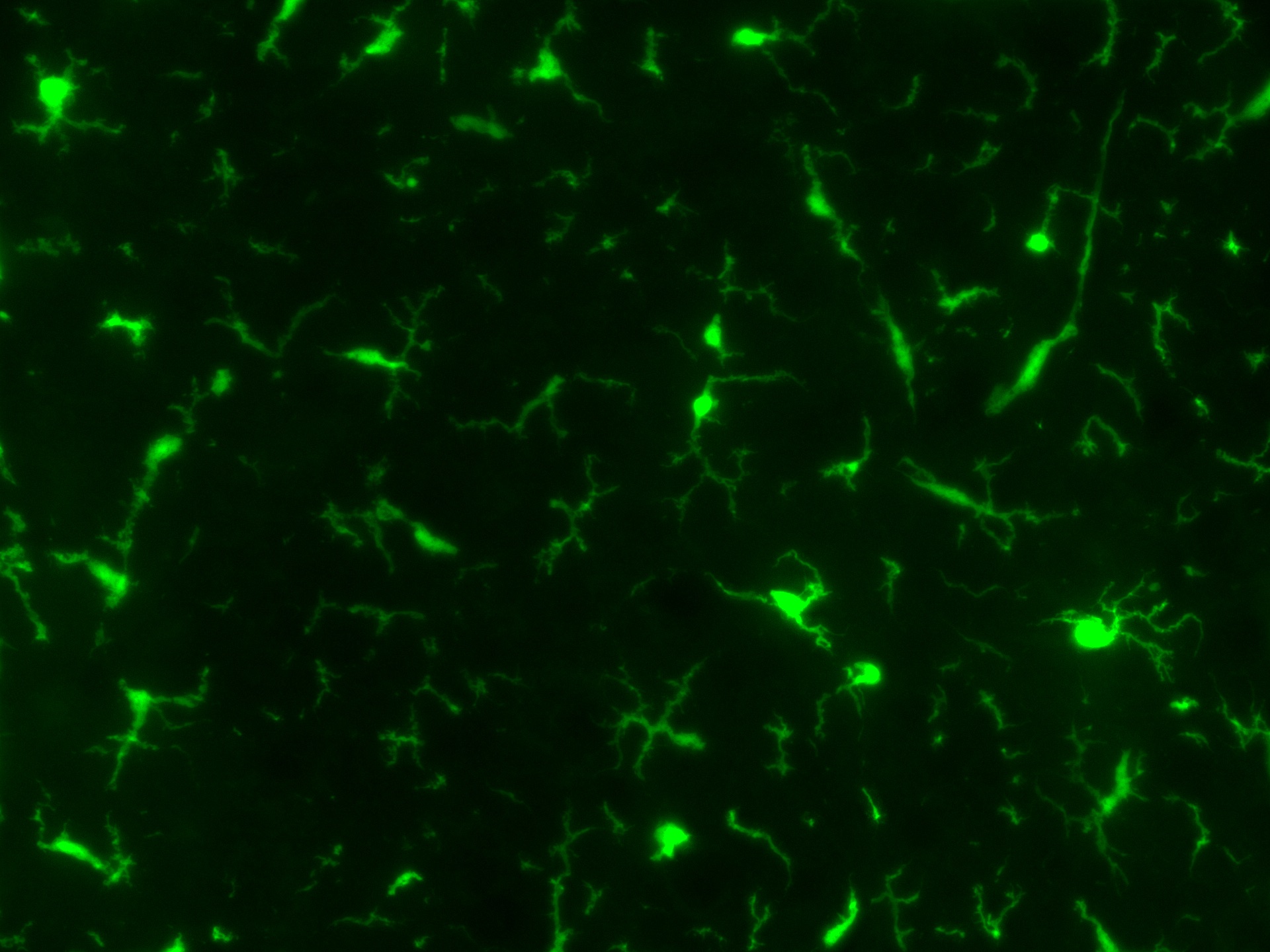

Supplement: Supplementary file 2. [file elife-102900-supp2.zip › Supplementary File 2/Raw IHC/GFP.jpeg]

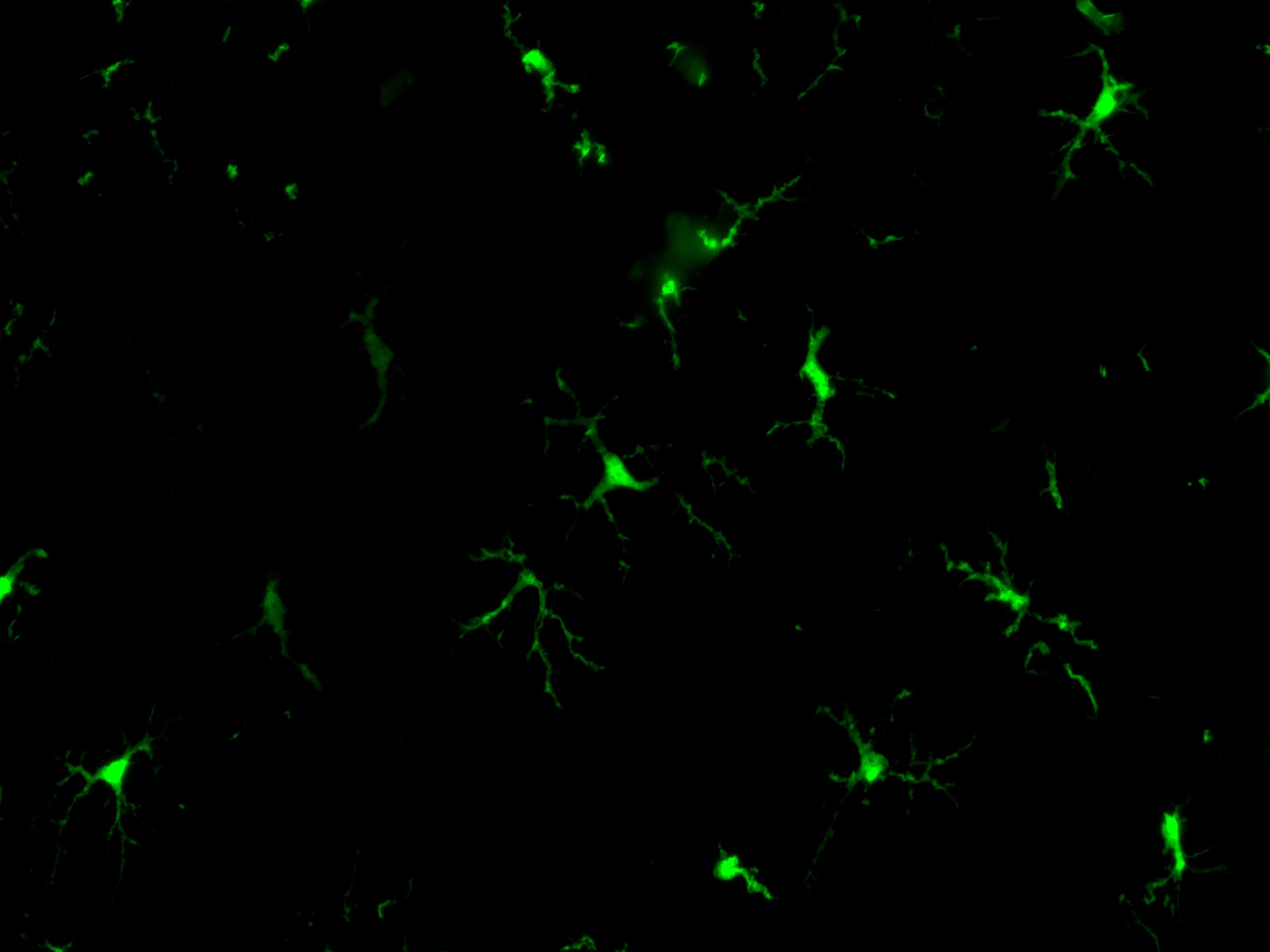

Supplement: Supplementary file 2. [file elife-102900-supp2.zip › Supplementary File 2/Raw IHC/1071 Z GFP.jpeg]

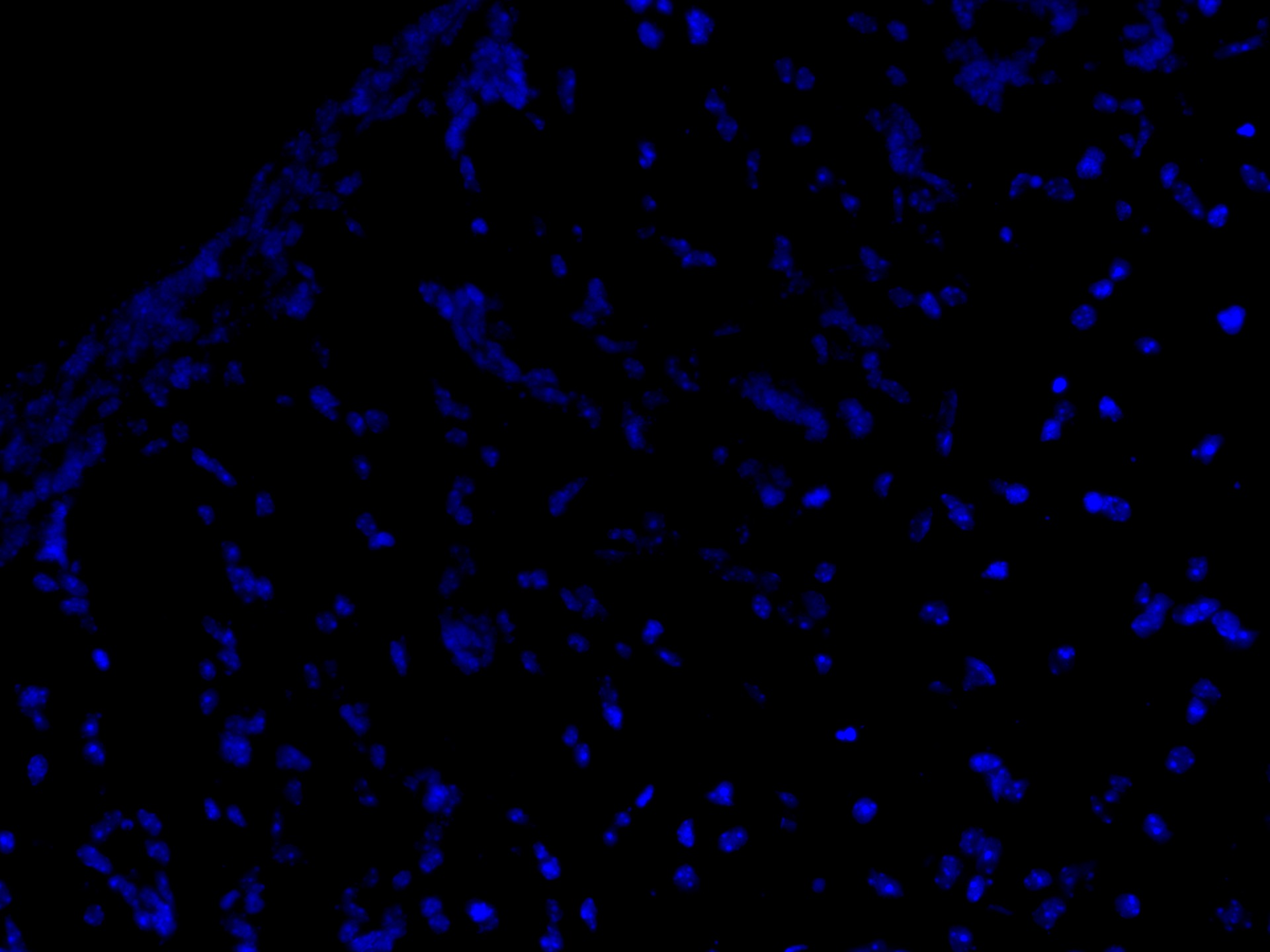

Supplement: Supplementary file 2. [file elife-102900-supp2.zip › Supplementary File 2/Raw IHC/1224 Z DAPI.jpeg]

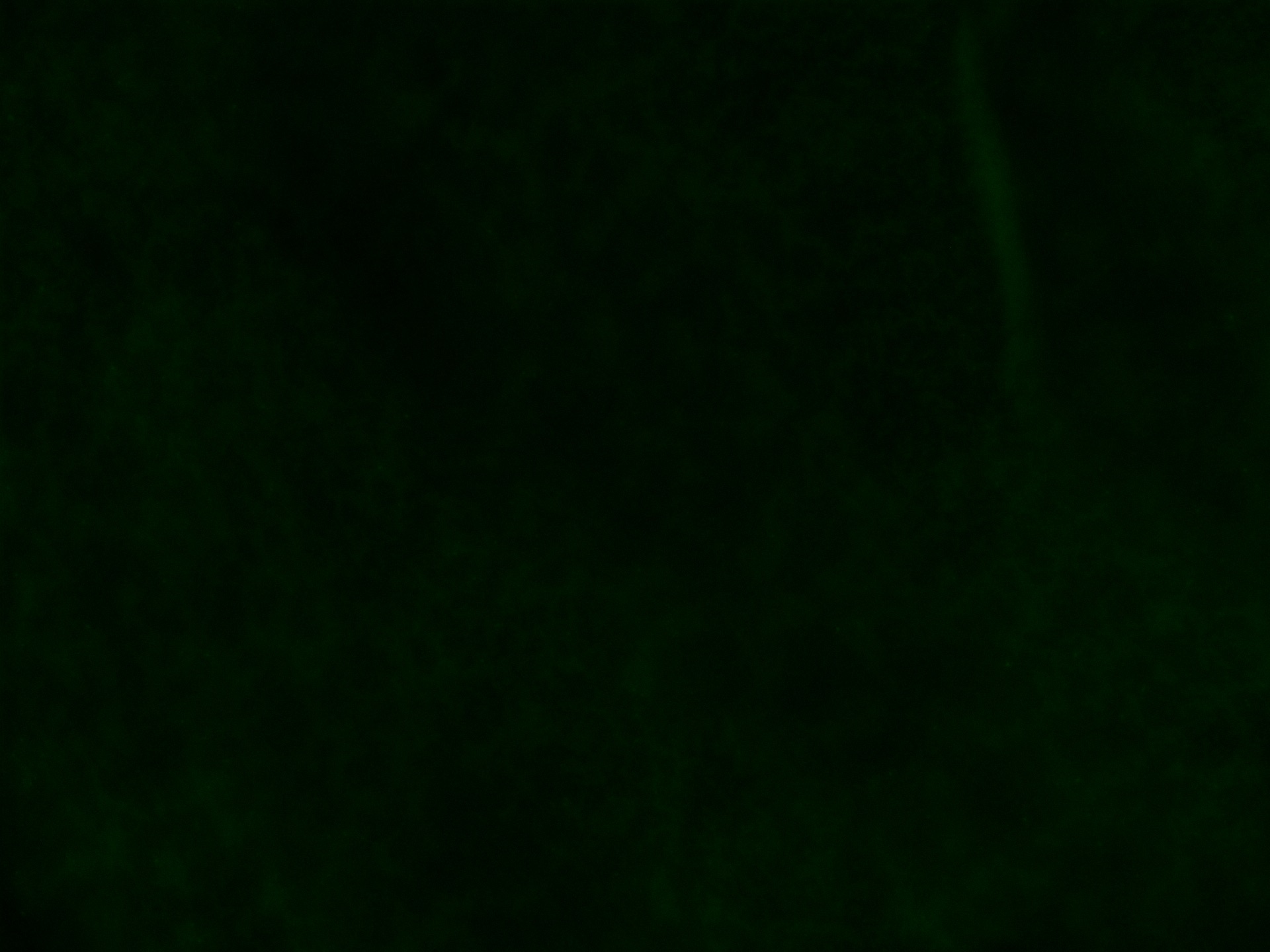

Supplement: Supplementary file 2. [file elife-102900-supp2.zip › Supplementary File 2/Raw IHC/gfp 3.jpeg]

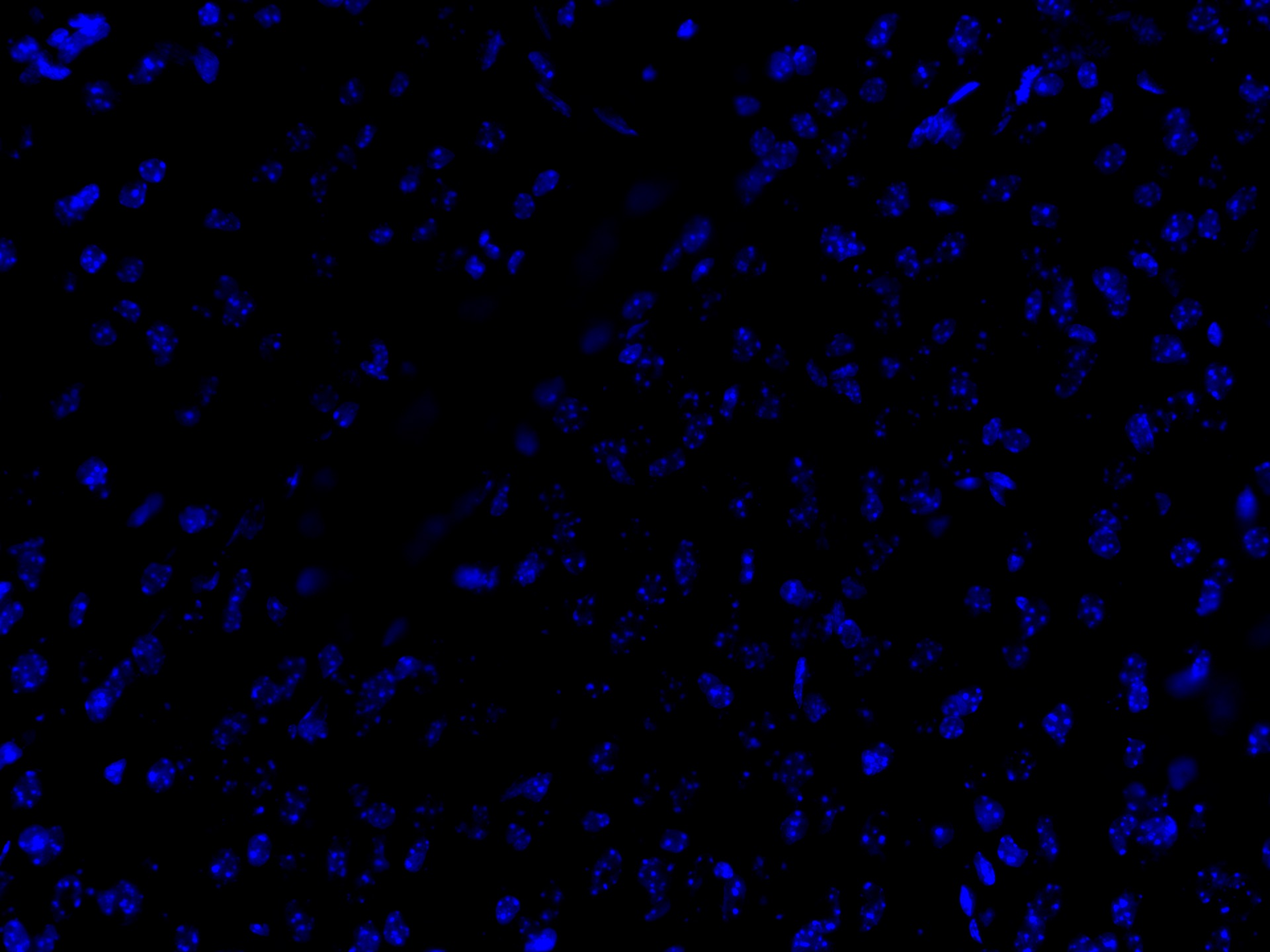

Supplement: Supplementary file 2. [file elife-102900-supp2.zip › Supplementary File 2/Raw IHC/1071 Z DAPI.jpeg]

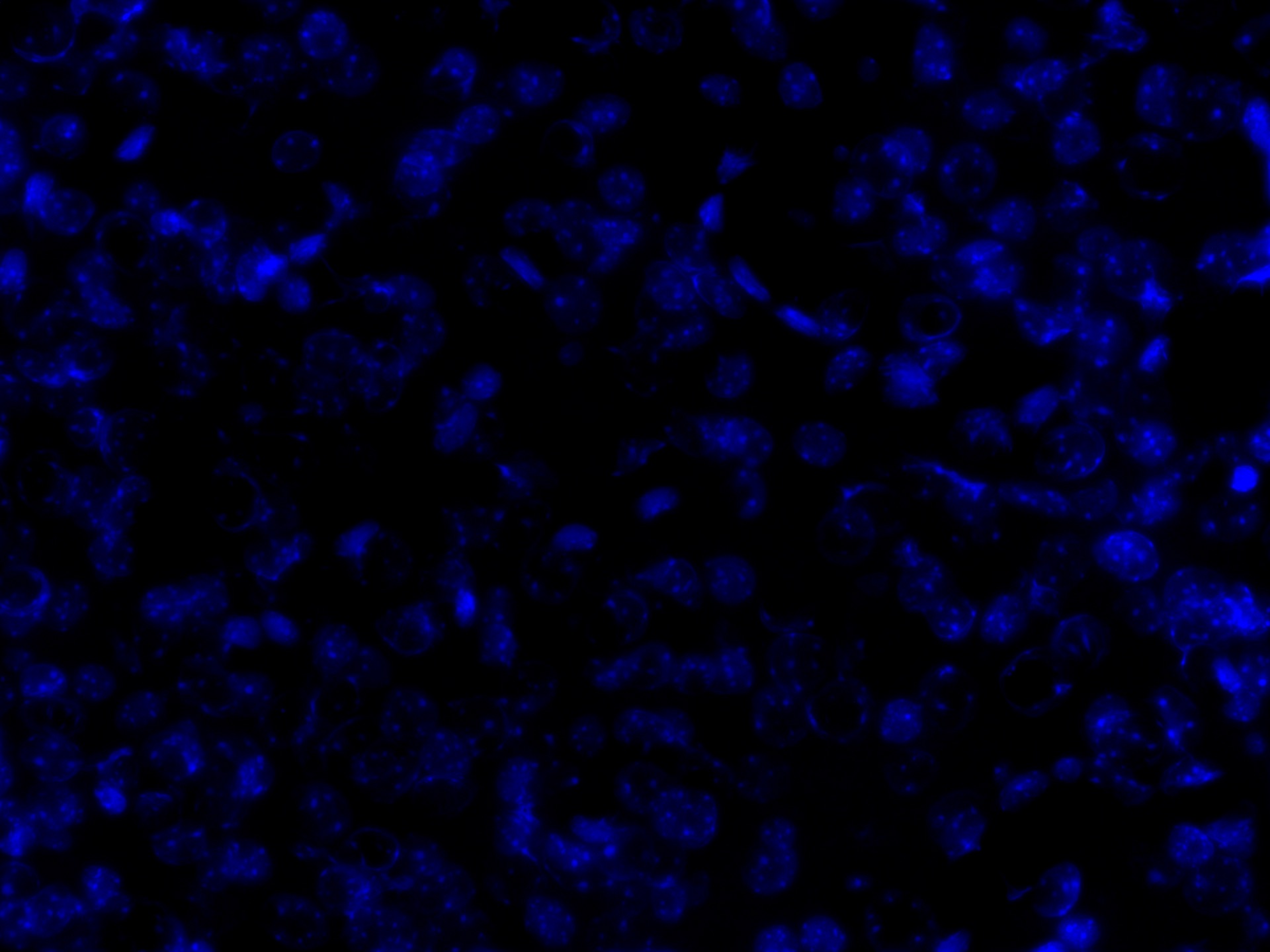

Supplement: Supplementary file 2. [file elife-102900-supp2.zip › Supplementary File 2/Raw IHC/819 DAPI 2.jpeg]

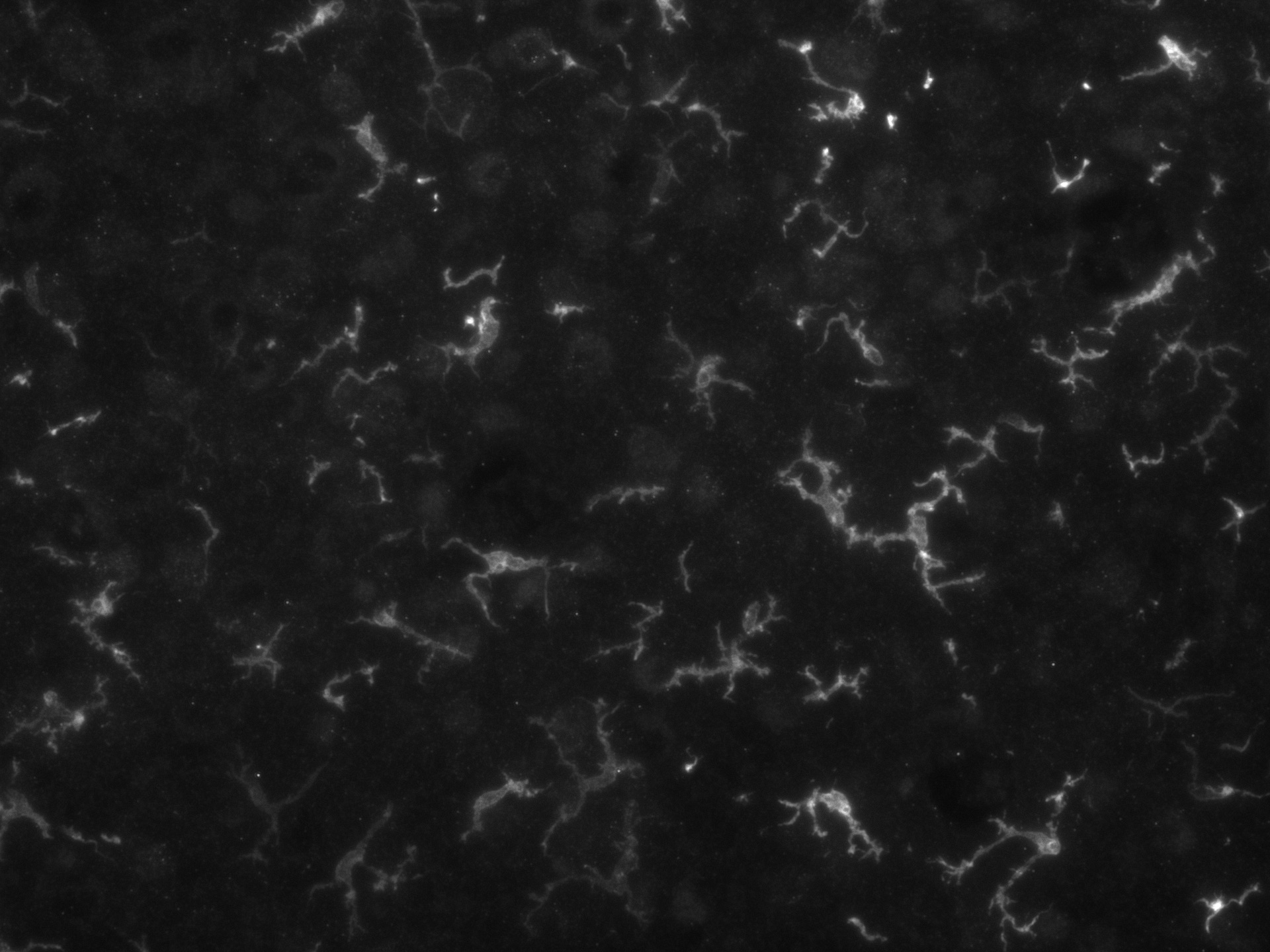

Supplement: Supplementary file 2. [file elife-102900-supp2.zip › Supplementary File 2/Raw IHC/FF_828_8 hoxb tx D8 p2ry12.jpeg]

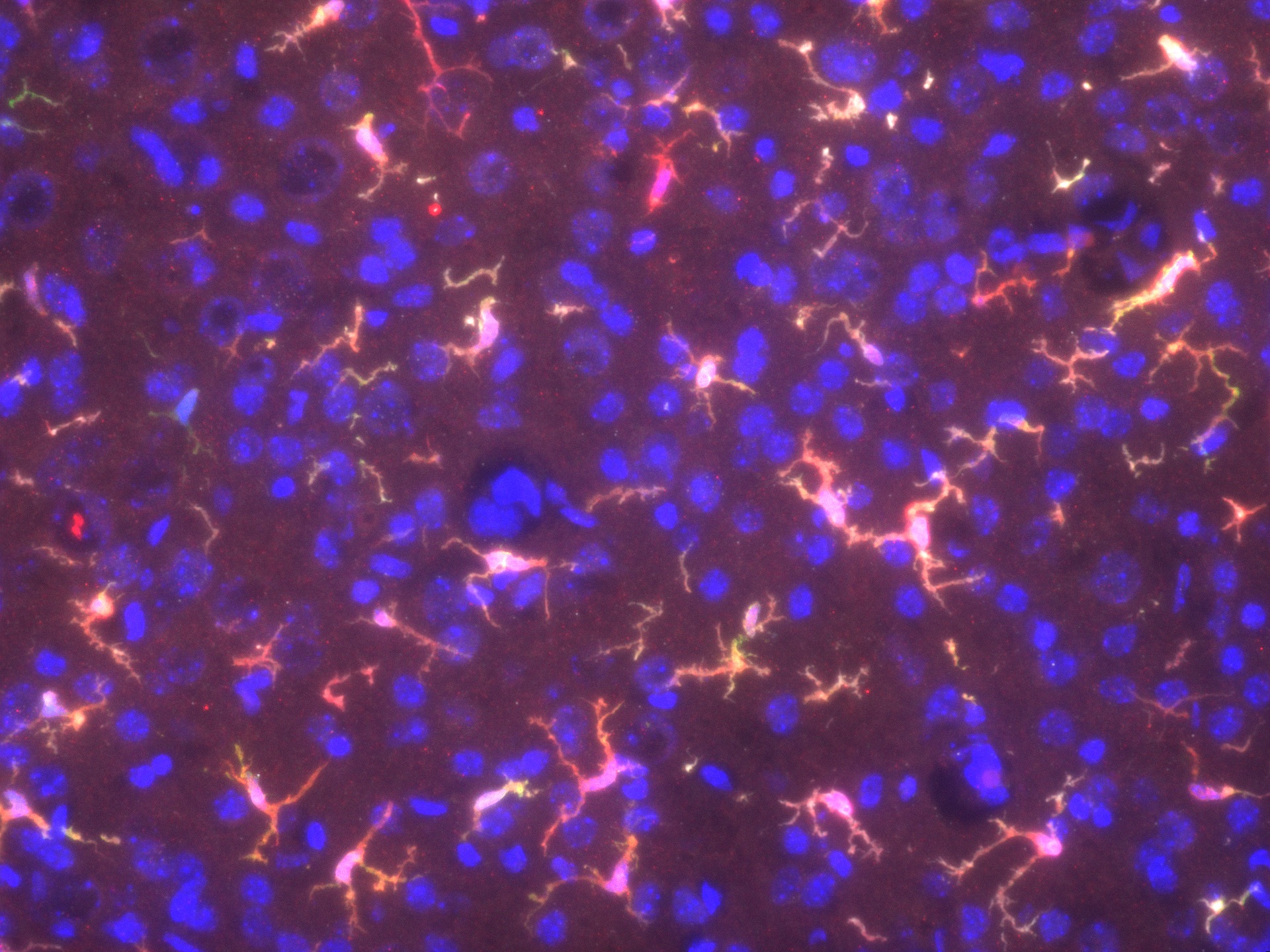

Supplement: Supplementary file 2. [file elife-102900-supp2.zip › Supplementary File 2/Raw IHC/FF_828_8 hoxb tx D8 overlay.jpeg]

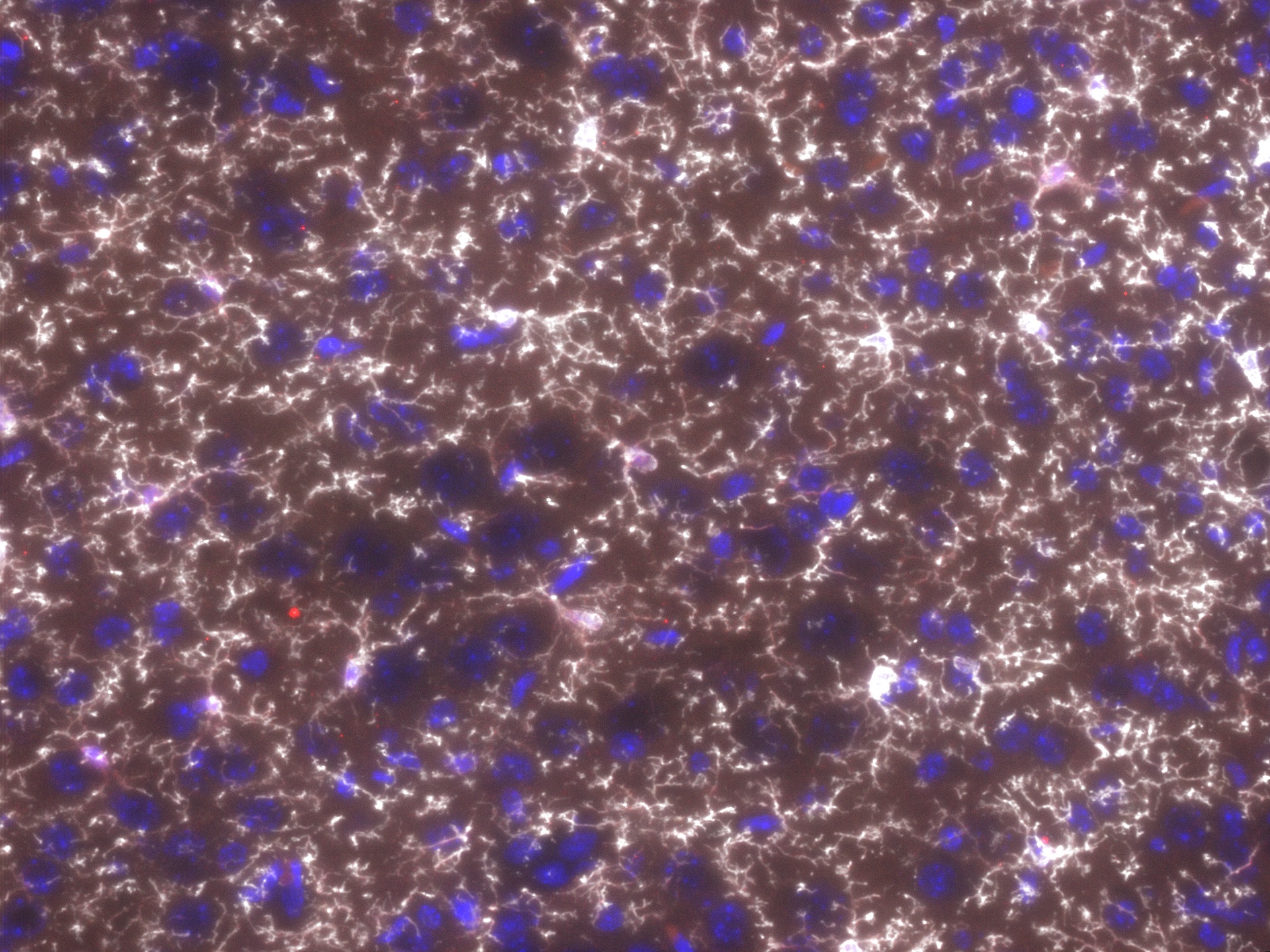

Supplement: Supplementary file 2. [file elife-102900-supp2.zip › Supplementary File 2/Raw IHC/FF_833 wt H8 02 overlay.jpeg]

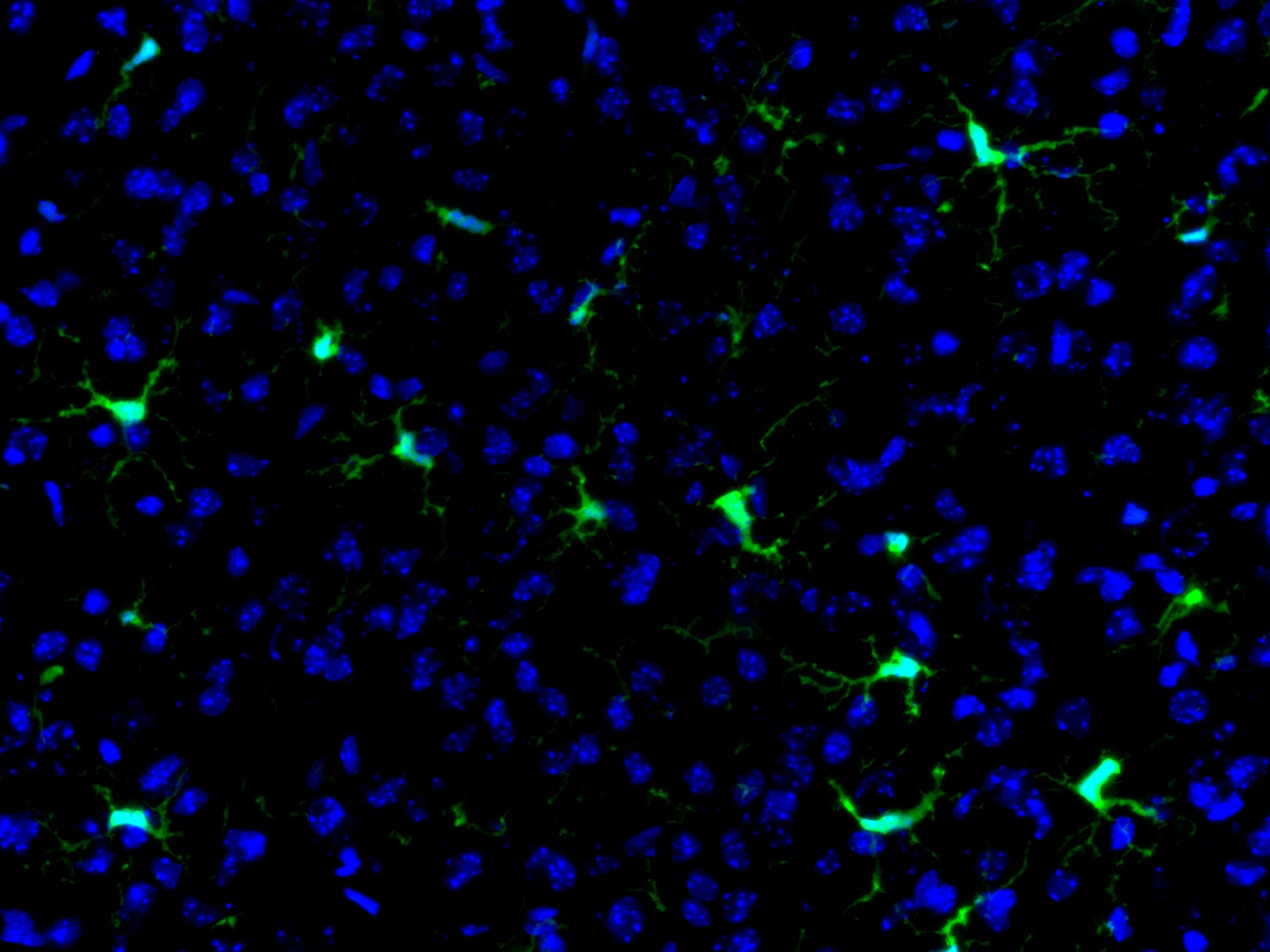

Supplement: Supplementary file 2. [file elife-102900-supp2.zip › Supplementary File 2/Raw IHC/1076 Z Overlay.jpeg]

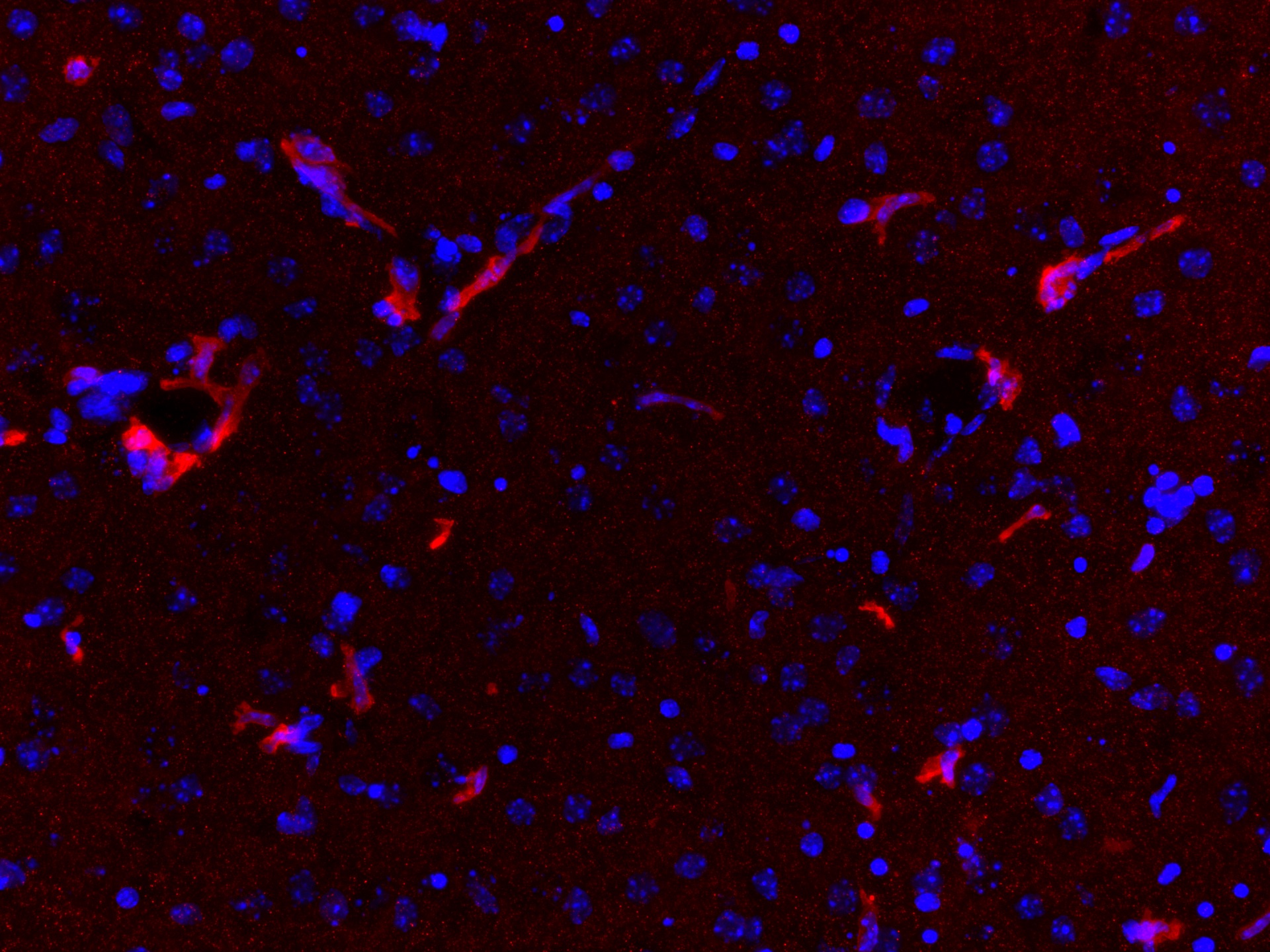

Supplement: Supplementary file 2. [file elife-102900-supp2.zip › Supplementary File 2/Raw IHC/1264 Overlay.jpeg]

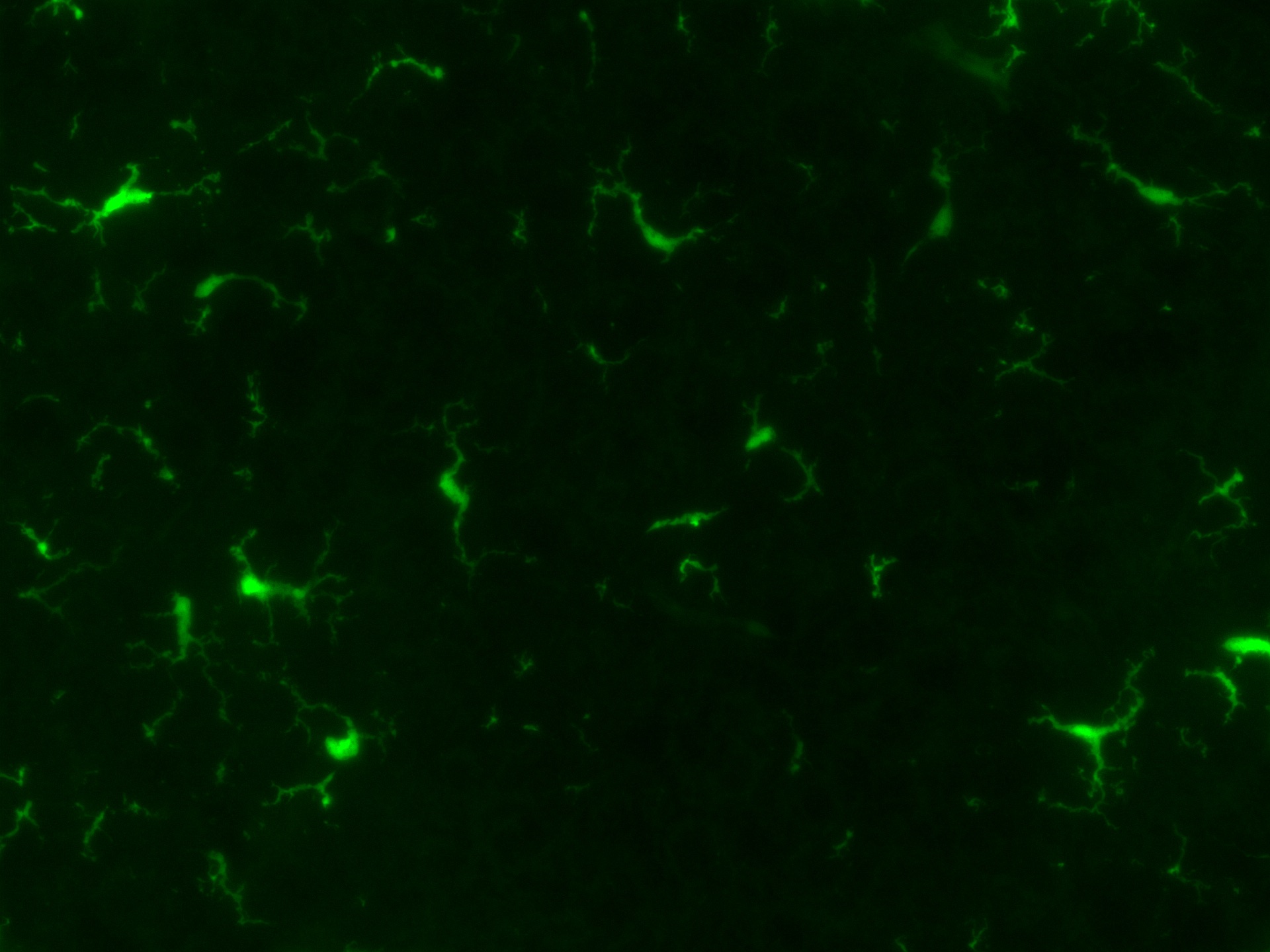

Supplement: Supplementary file 2. [file elife-102900-supp2.zip › Supplementary File 2/Raw IHC/gfp 2.jpeg]

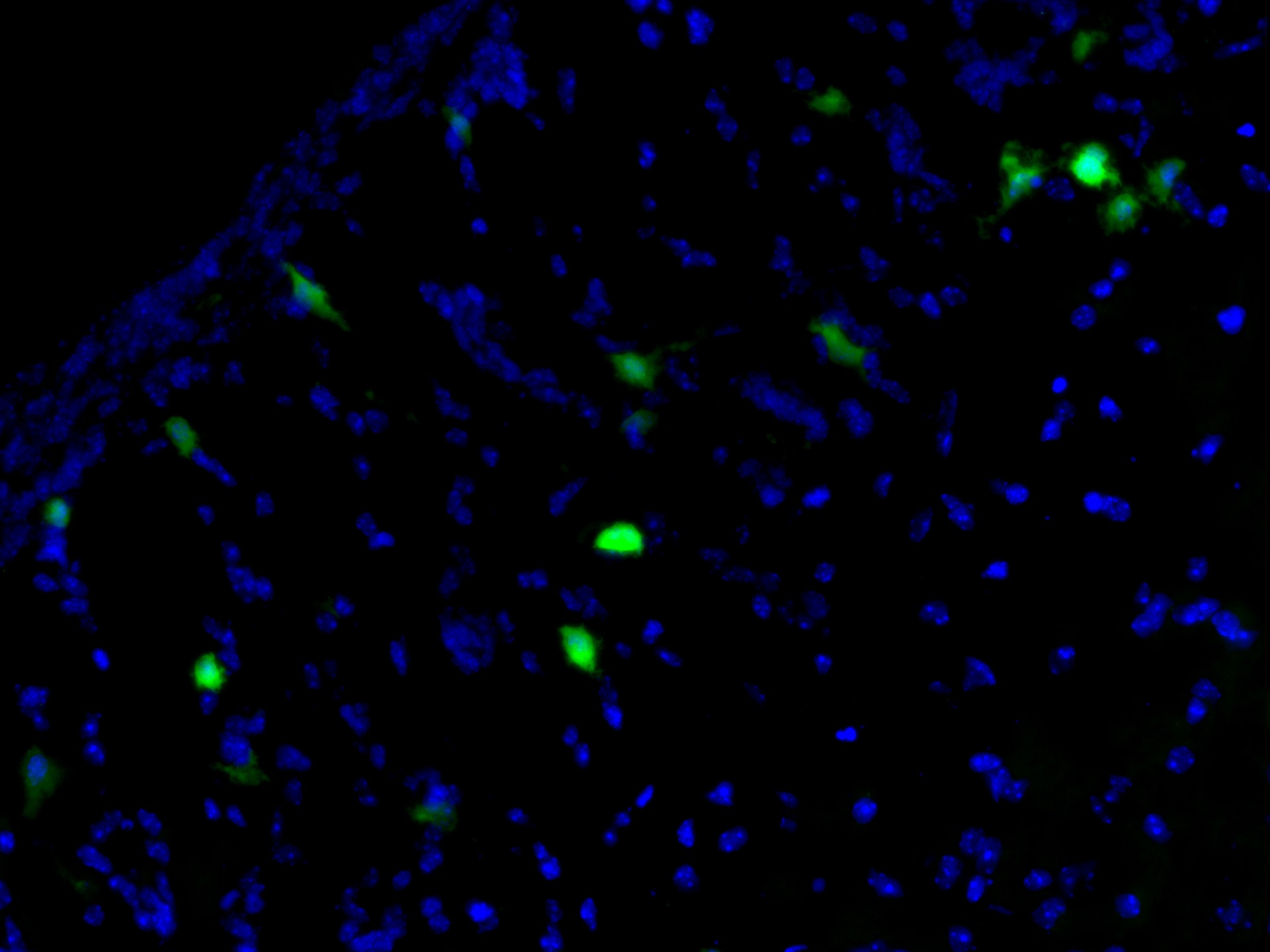

Supplement: Supplementary file 2. [file elife-102900-supp2.zip › Supplementary File 2/Raw IHC/1224 Z Overlay.jpeg]

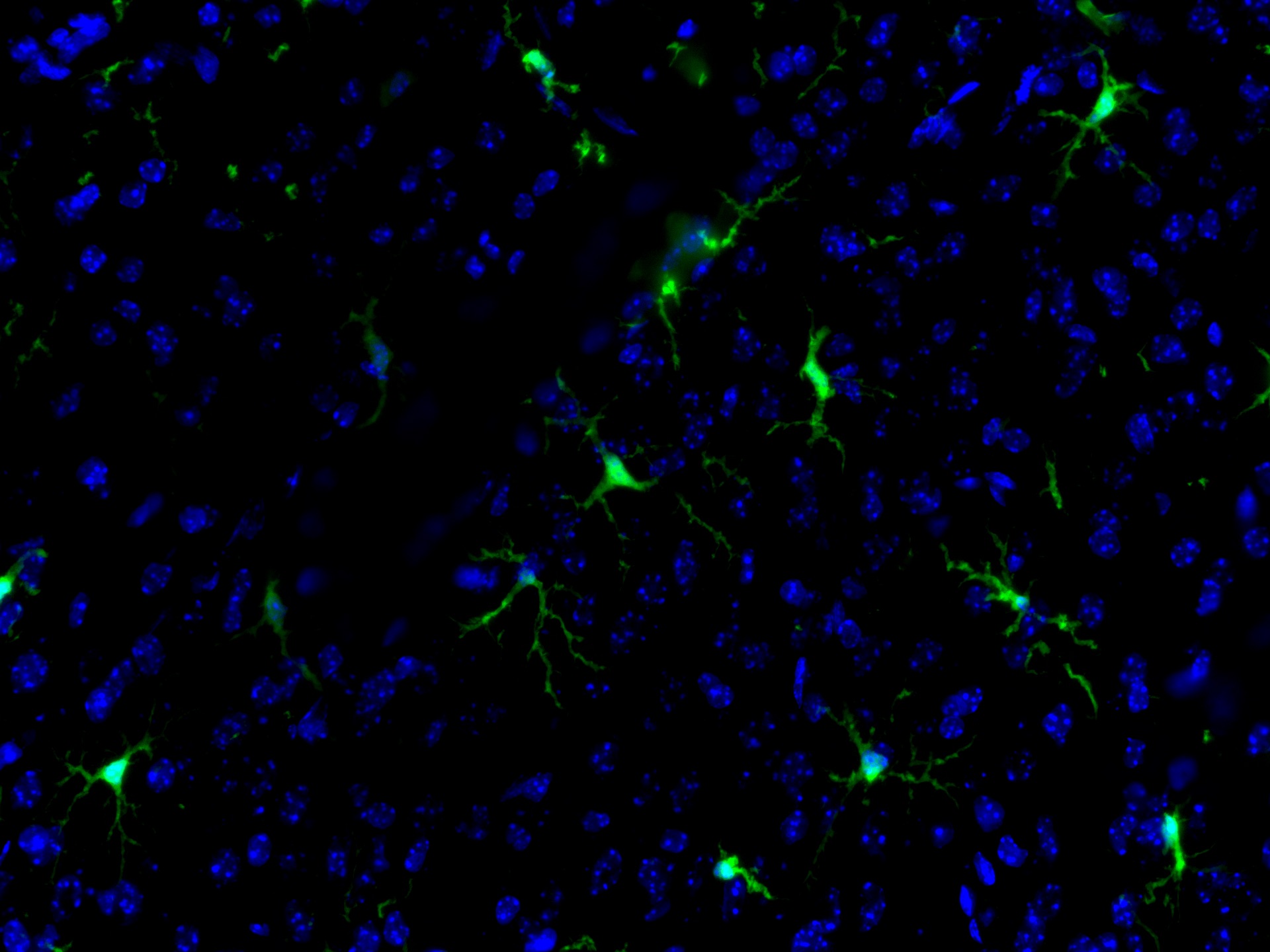

Supplement: Supplementary file 2. [file elife-102900-supp2.zip › Supplementary File 2/Raw IHC/1071 Z Overlay.jpeg]

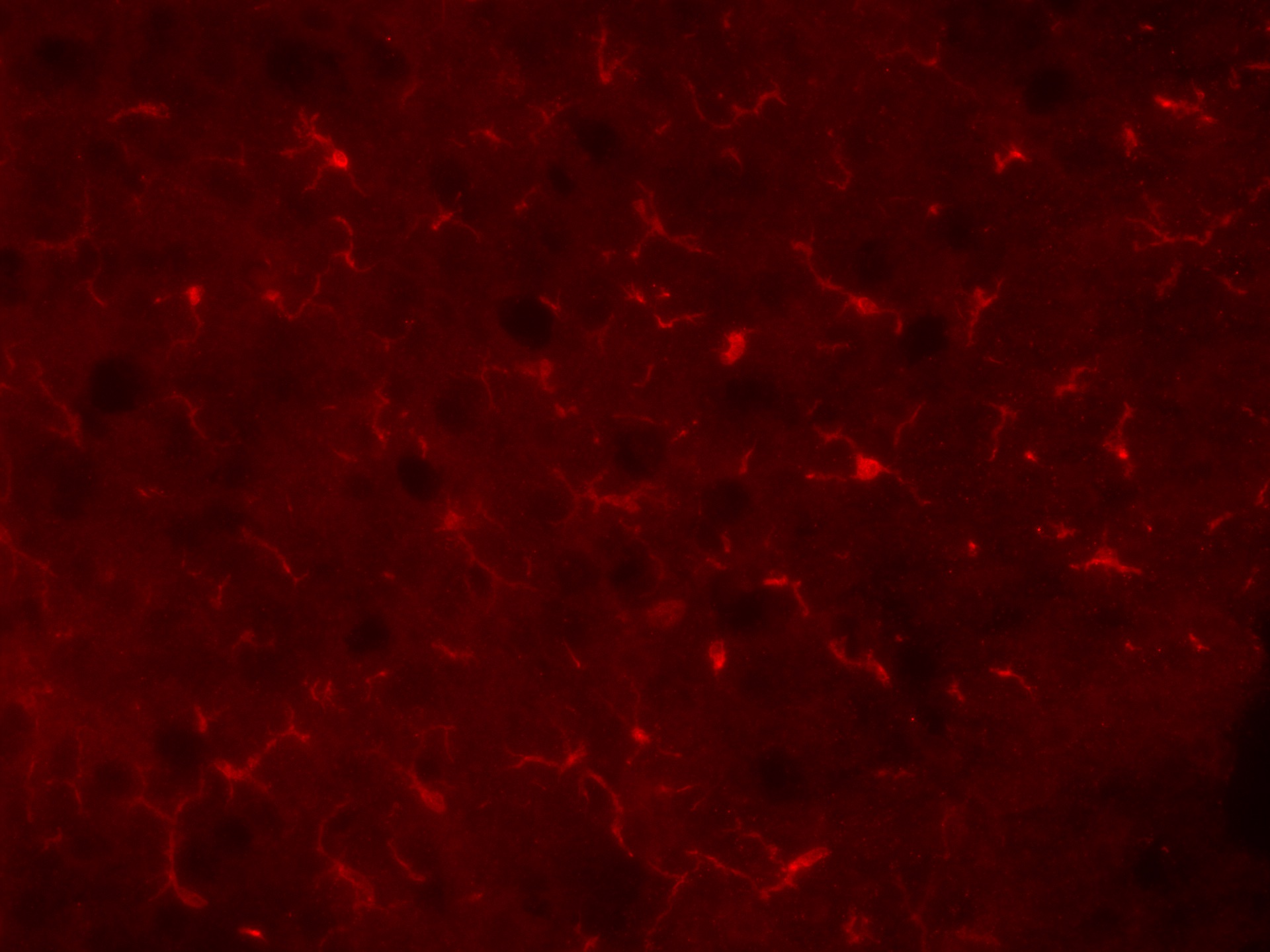

Supplement: Supplementary file 2. [file elife-102900-supp2.zip › Supplementary File 2/Raw IHC/FF_748_5 bm tx H top 8 iba1.jpeg]

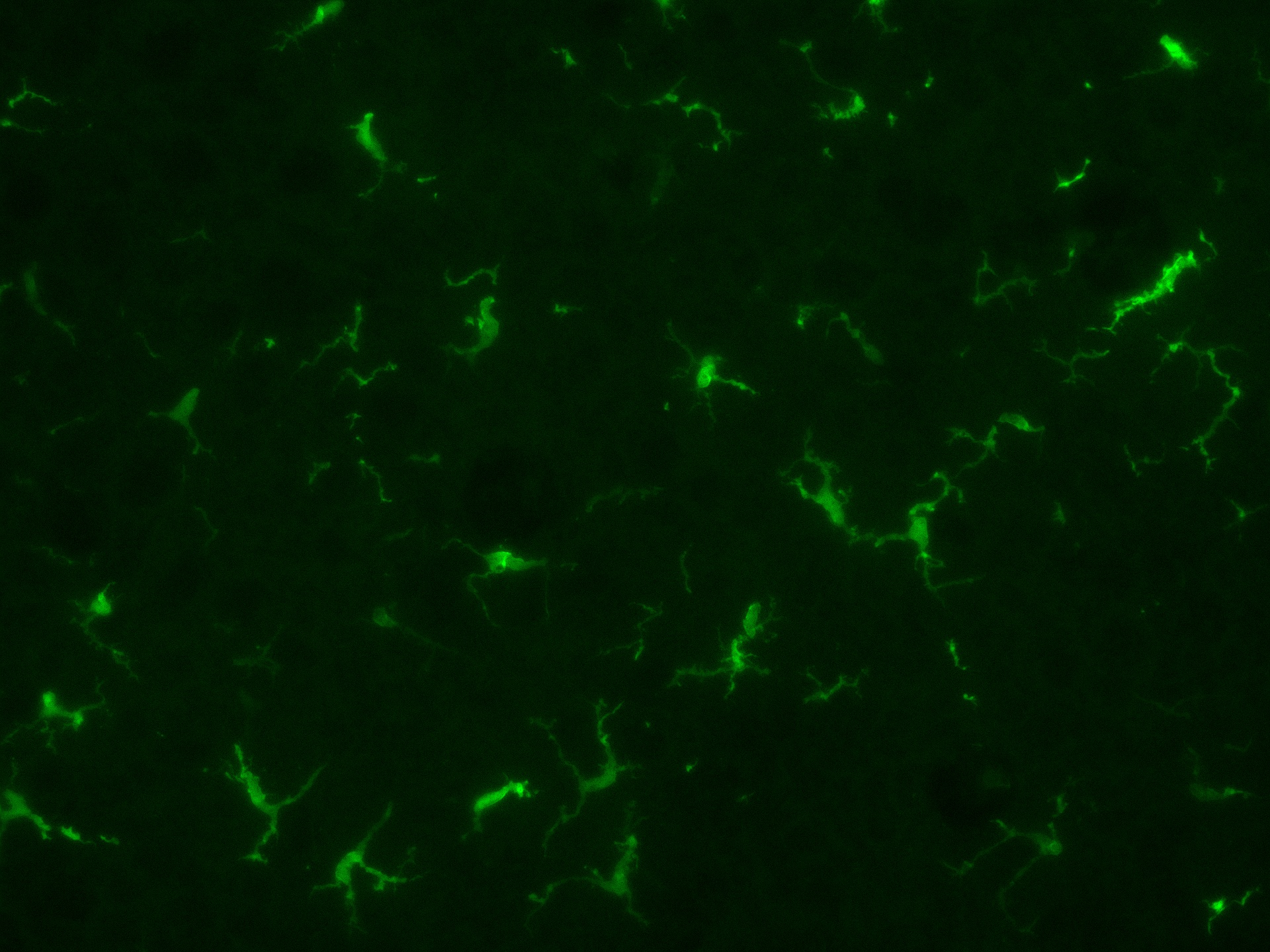

Supplement: Supplementary file 2. [file elife-102900-supp2.zip › Supplementary File 2/Raw IHC/FF_828_8 hoxb tx D8 gfp.jpeg]

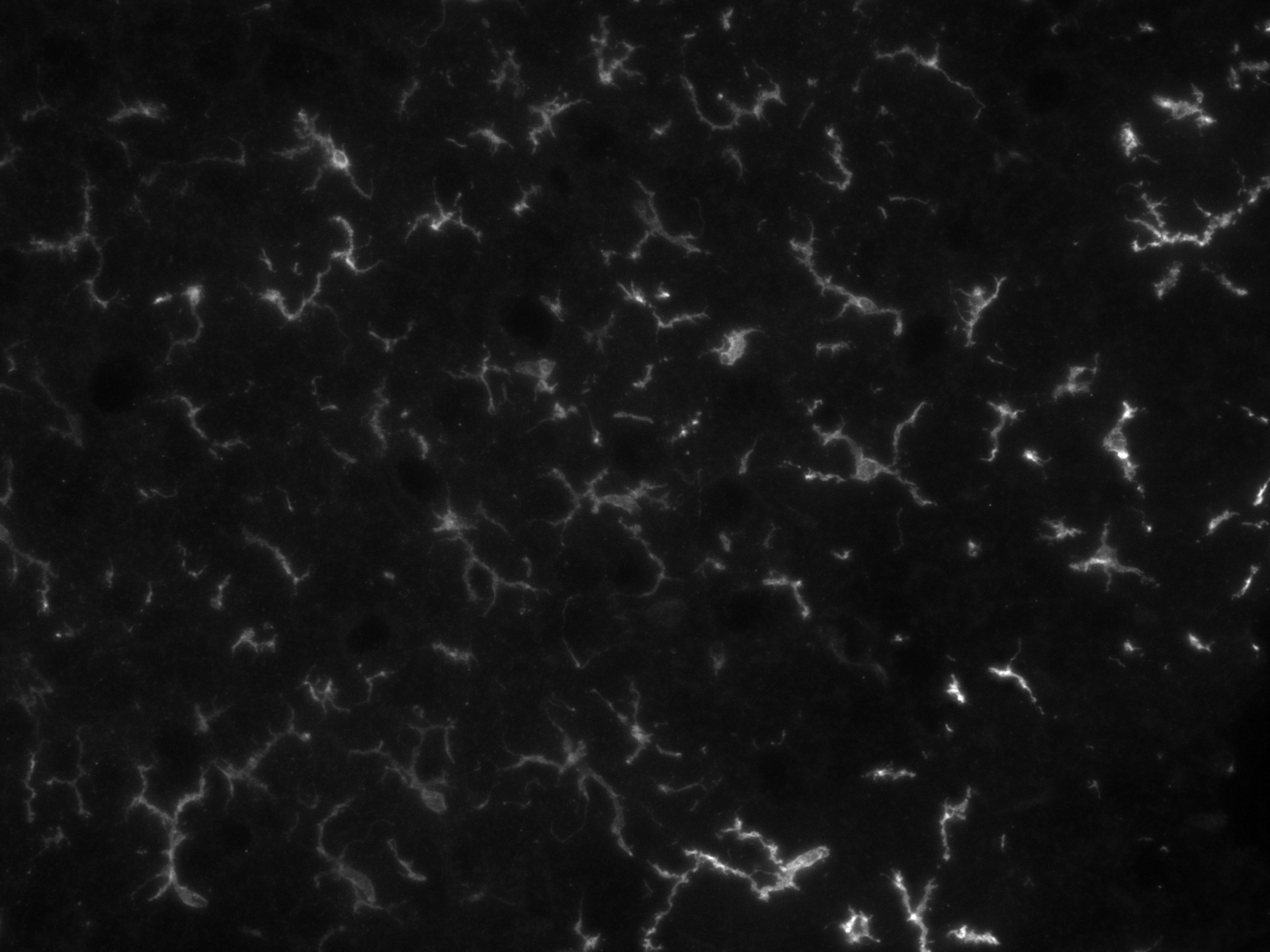

Supplement: Supplementary file 2. [file elife-102900-supp2.zip › Supplementary File 2/Raw IHC/FF_748_5 bm tx H top 8 p2ry12.jpeg]

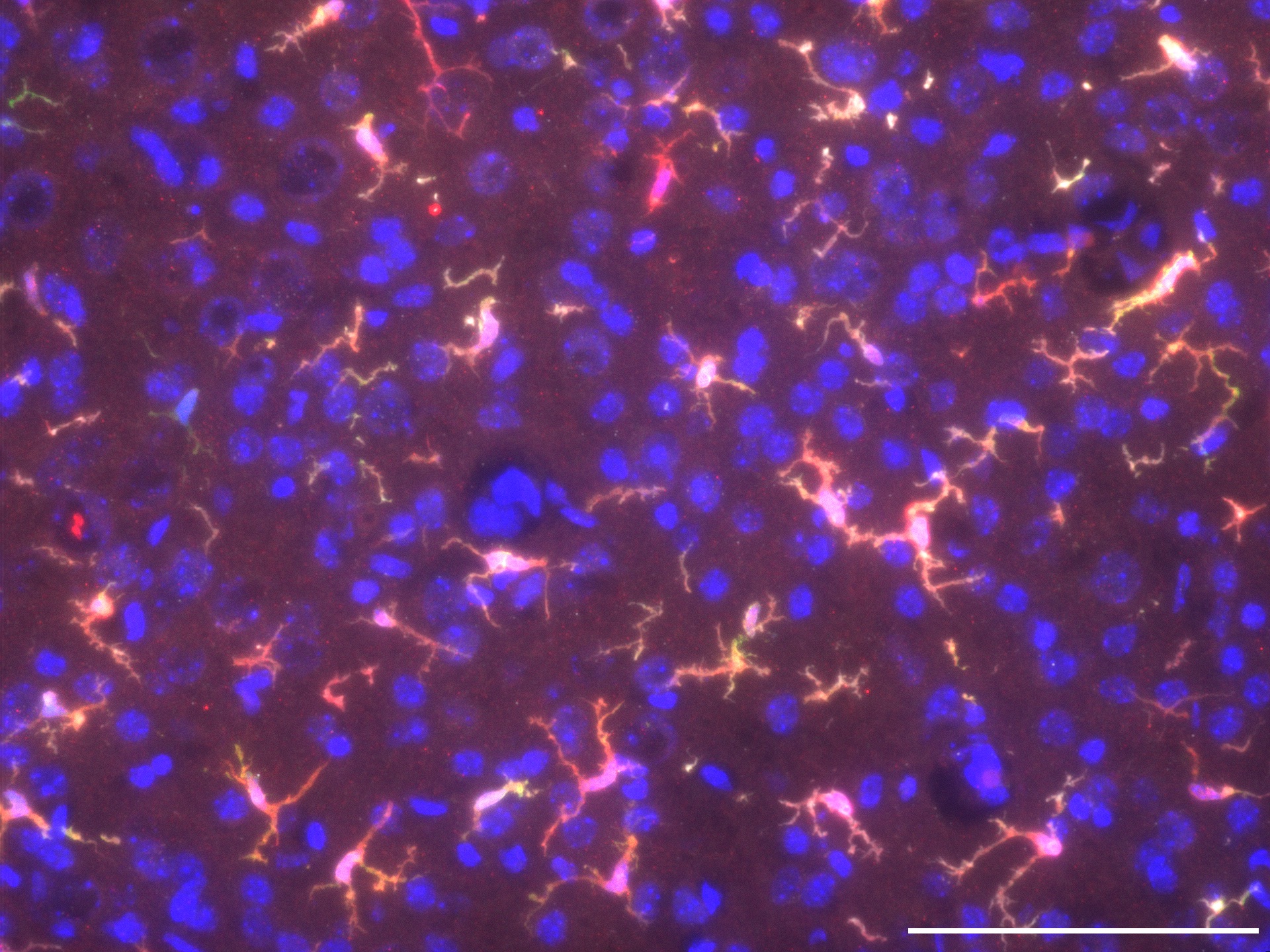

Supplement: Supplementary file 2. [file elife-102900-supp2.zip › Supplementary File 2/Raw IHC/FF_828_8 hoxb tx D8 overlay with 100um scale bar.jpeg]

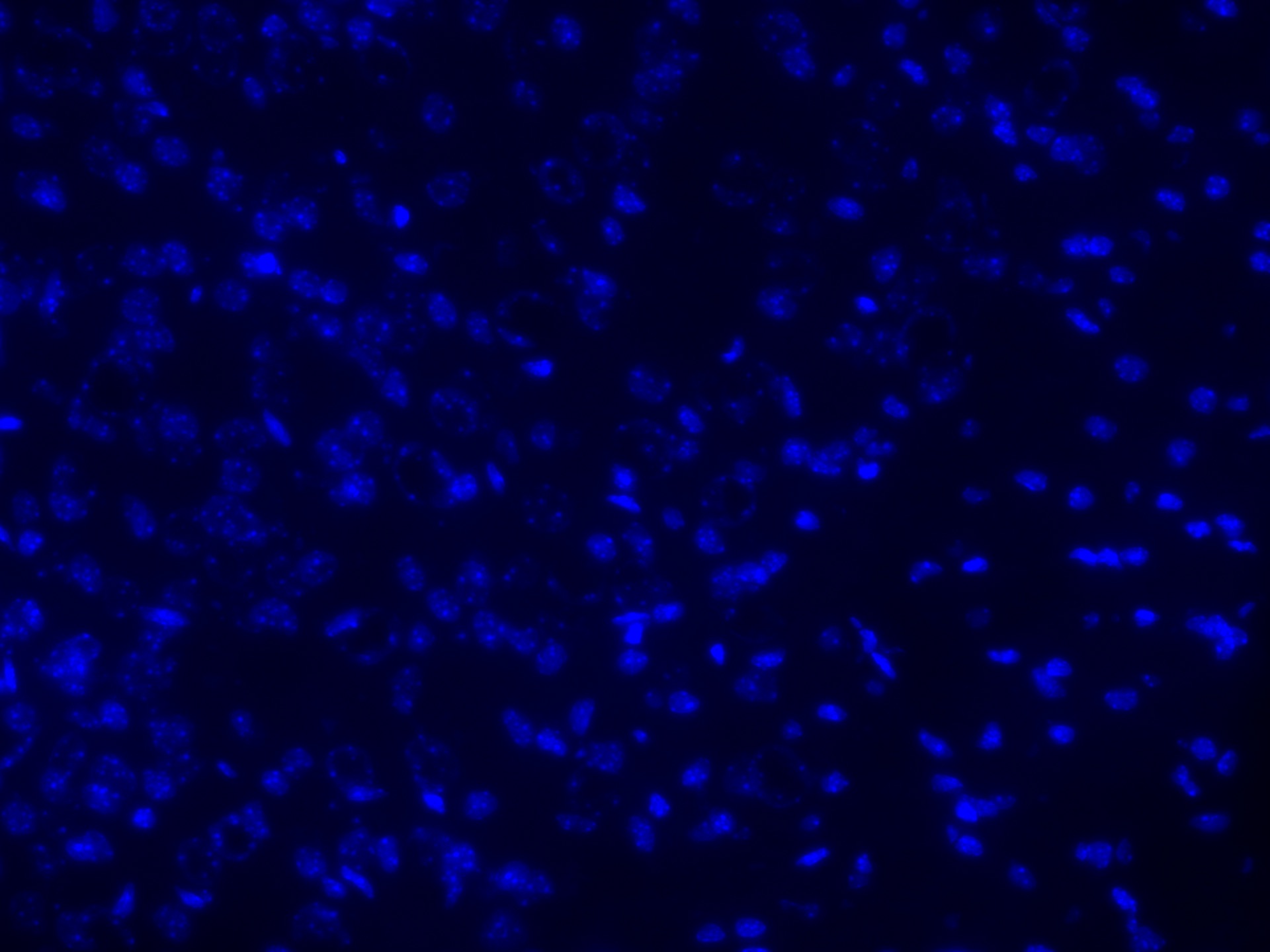

Supplement: Supplementary file 2. [file elife-102900-supp2.zip › Supplementary File 2/Raw IHC/FF_748_5 bm tx H top 8 dapi.jpeg]

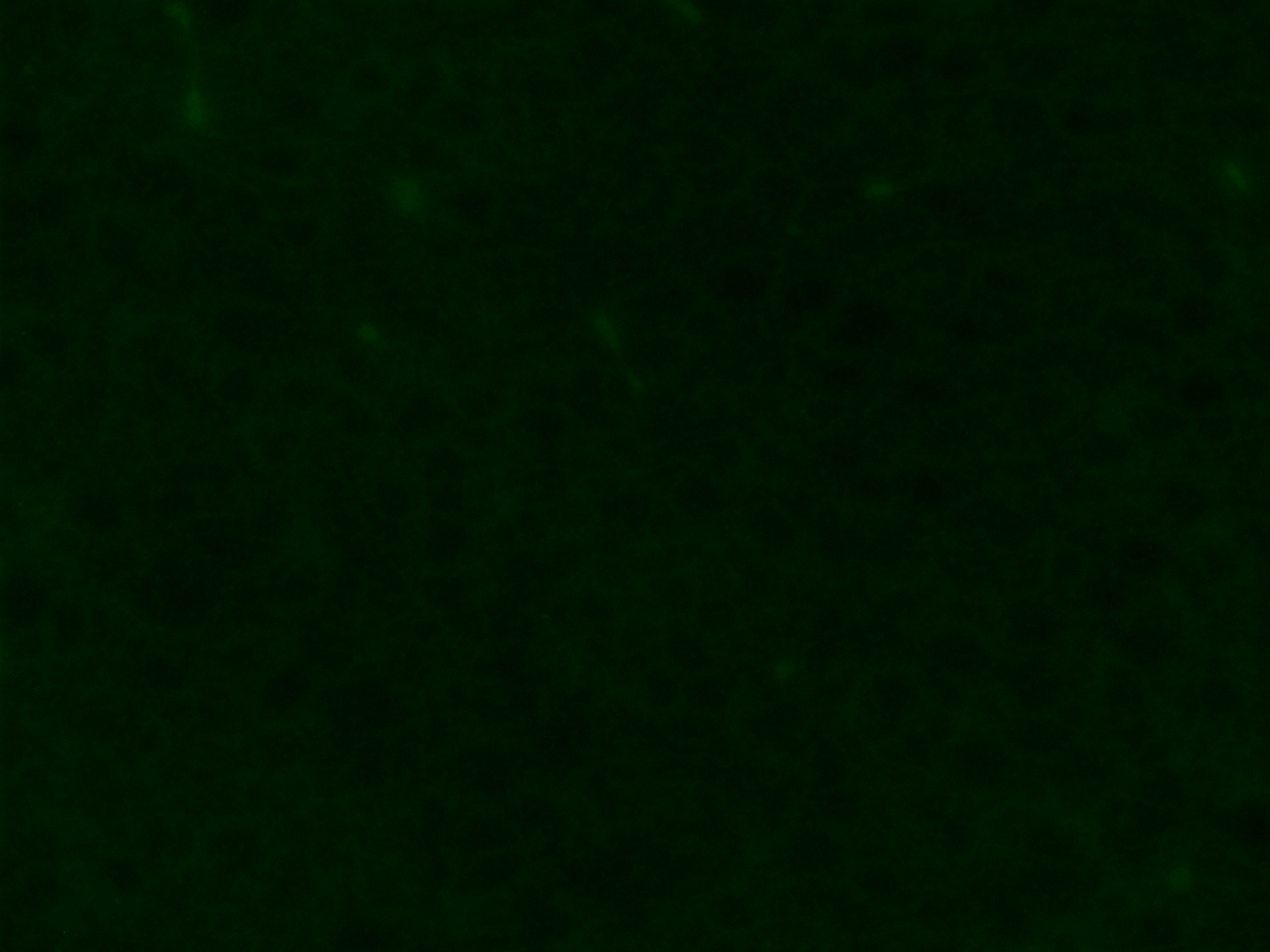

Supplement: Supplementary file 2. [file elife-102900-supp2.zip › Supplementary File 2/Raw IHC/GFP 4.jpeg]

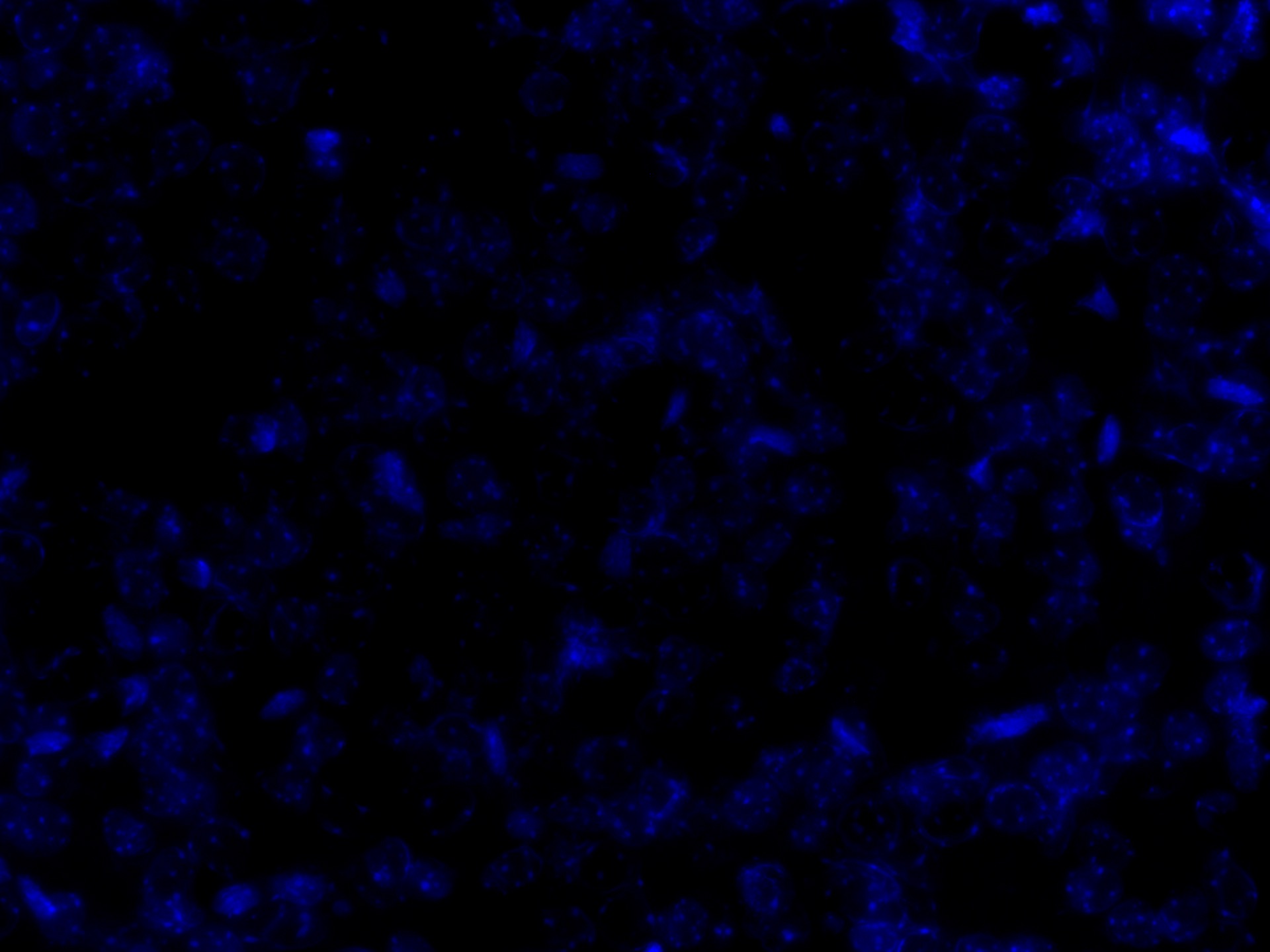

Supplement: Supplementary file 2. [file elife-102900-supp2.zip › Supplementary File 2/Raw IHC/819 DAPI.jpeg]

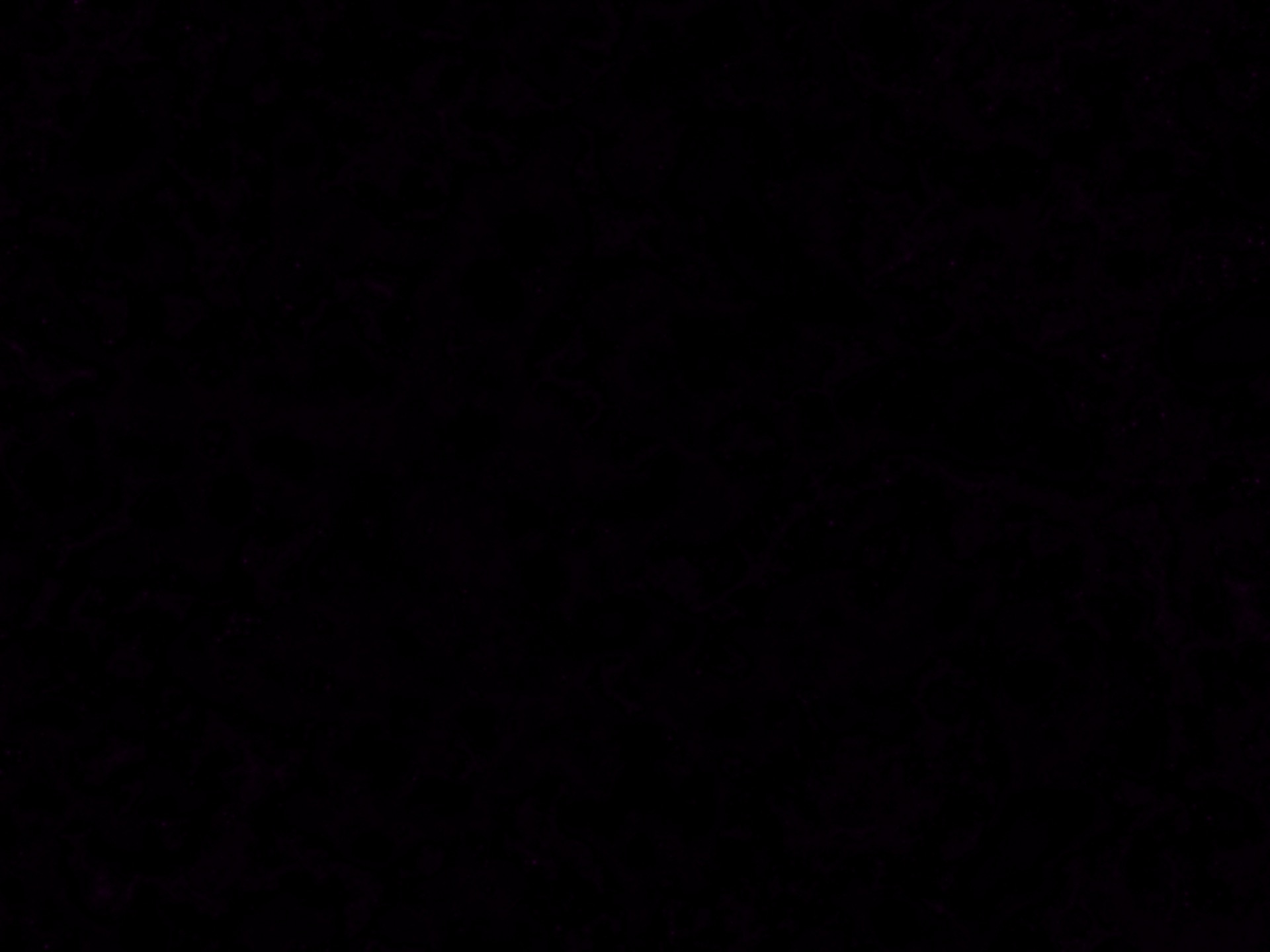

Supplement: Supplementary file 2. [file elife-102900-supp2.zip › Supplementary File 2/Raw IHC/819 Isg.jpeg]

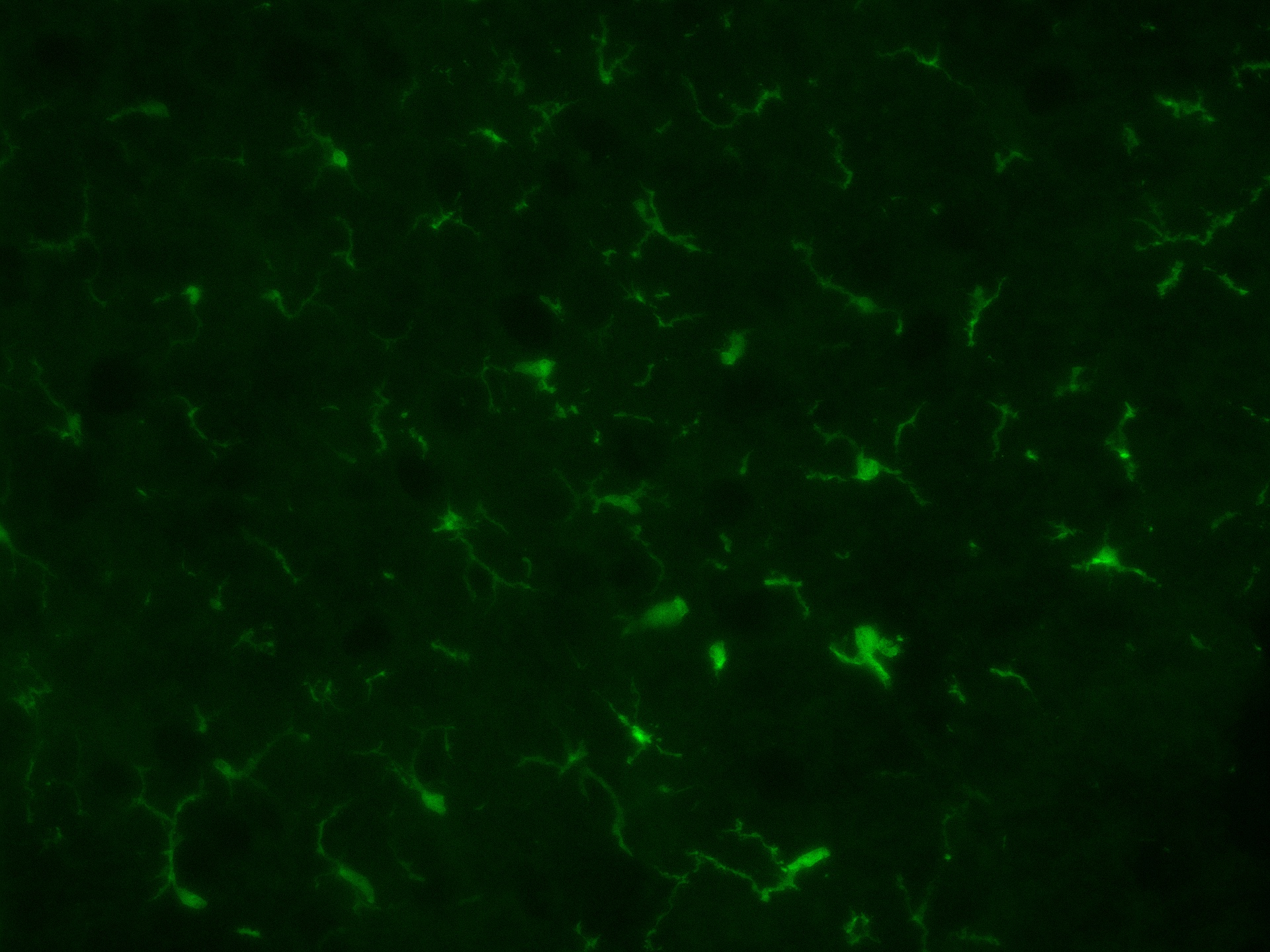

Supplement: Supplementary file 2. [file elife-102900-supp2.zip › Supplementary File 2/Raw IHC/FF_748_5 bm tx H top 8 gfp.jpeg]

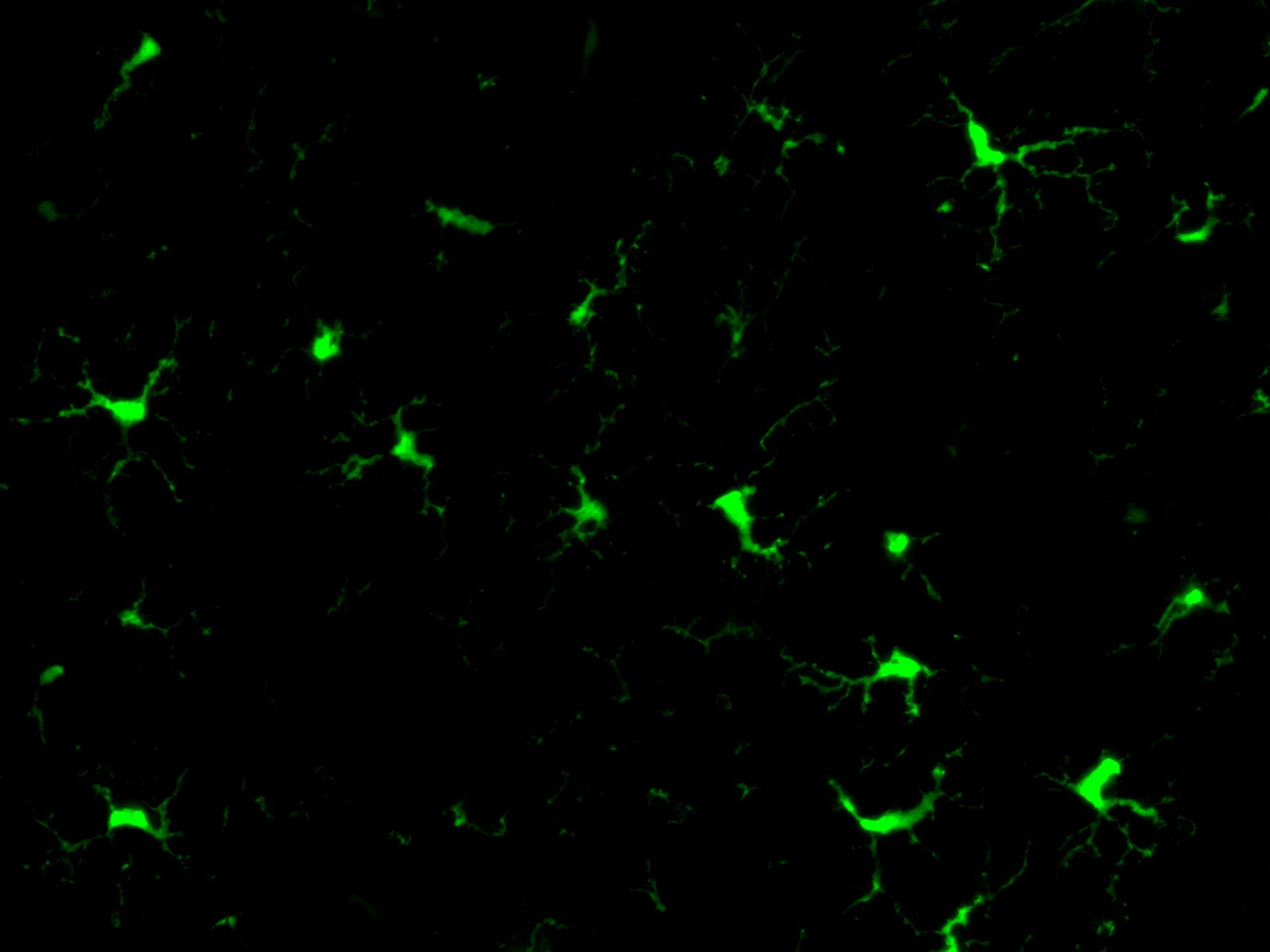

Supplement: Supplementary file 2. [file elife-102900-supp2.zip › Supplementary File 2/Raw IHC/1076 Z GFP.jpeg]
