# Supplementary figures and images for: Microglia replacement by ER-Hoxb8 conditionally immortalized macrophages provides insight into Aicardi–Goutières syndrome neuropathology (part 1 of 2)
Source: eLife. 2026 Jan 27;14:RP102900. doi: 10.7554/eLife.102900 (PMC12844908; doi:10.7554/eLife.102900)

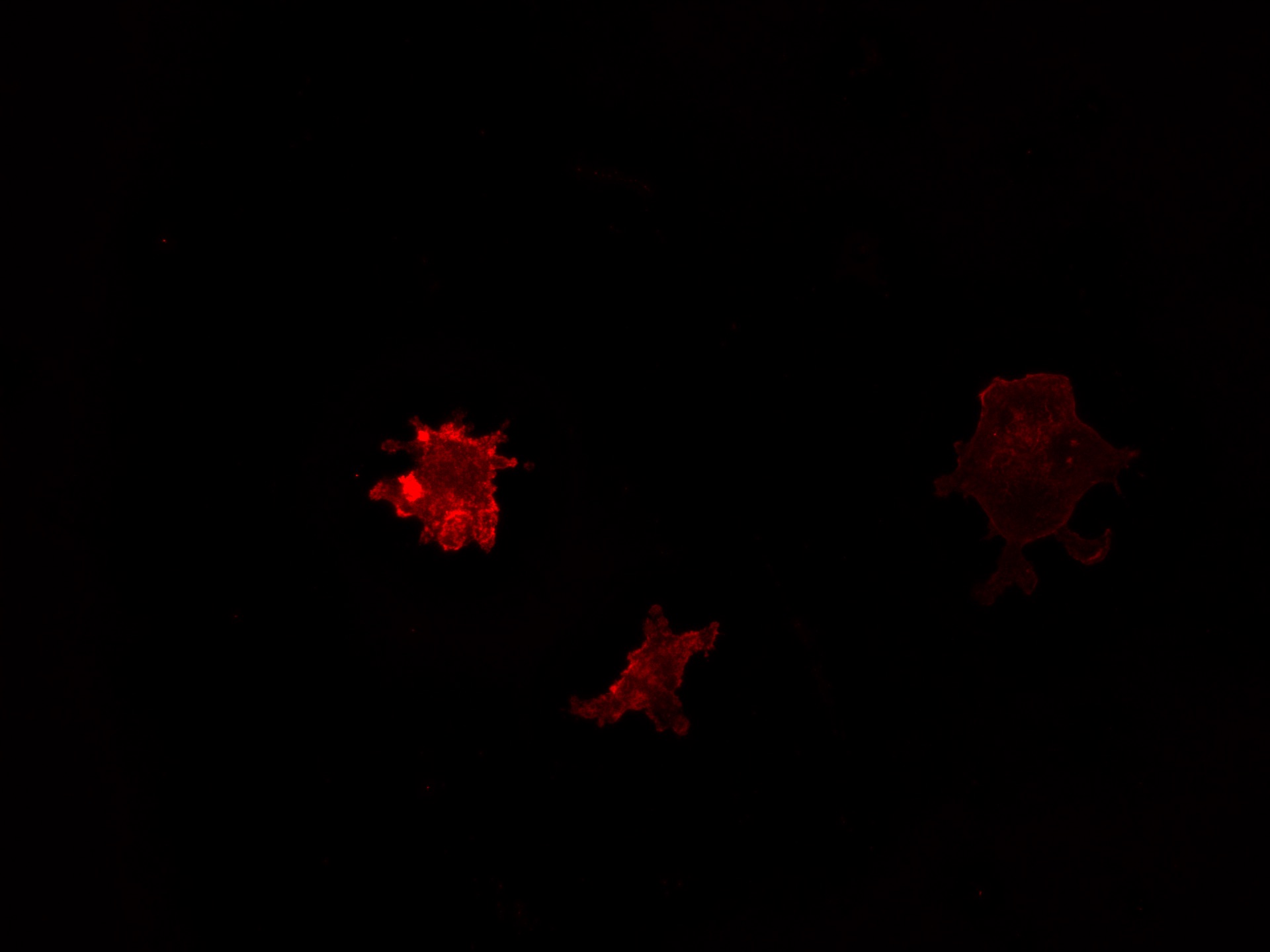

Supplement: Supplementary file 2. [file elife-102900-supp2.zip › Supplementary File 2/Raw ICC/A2 40x Z Cd11b.jpeg]

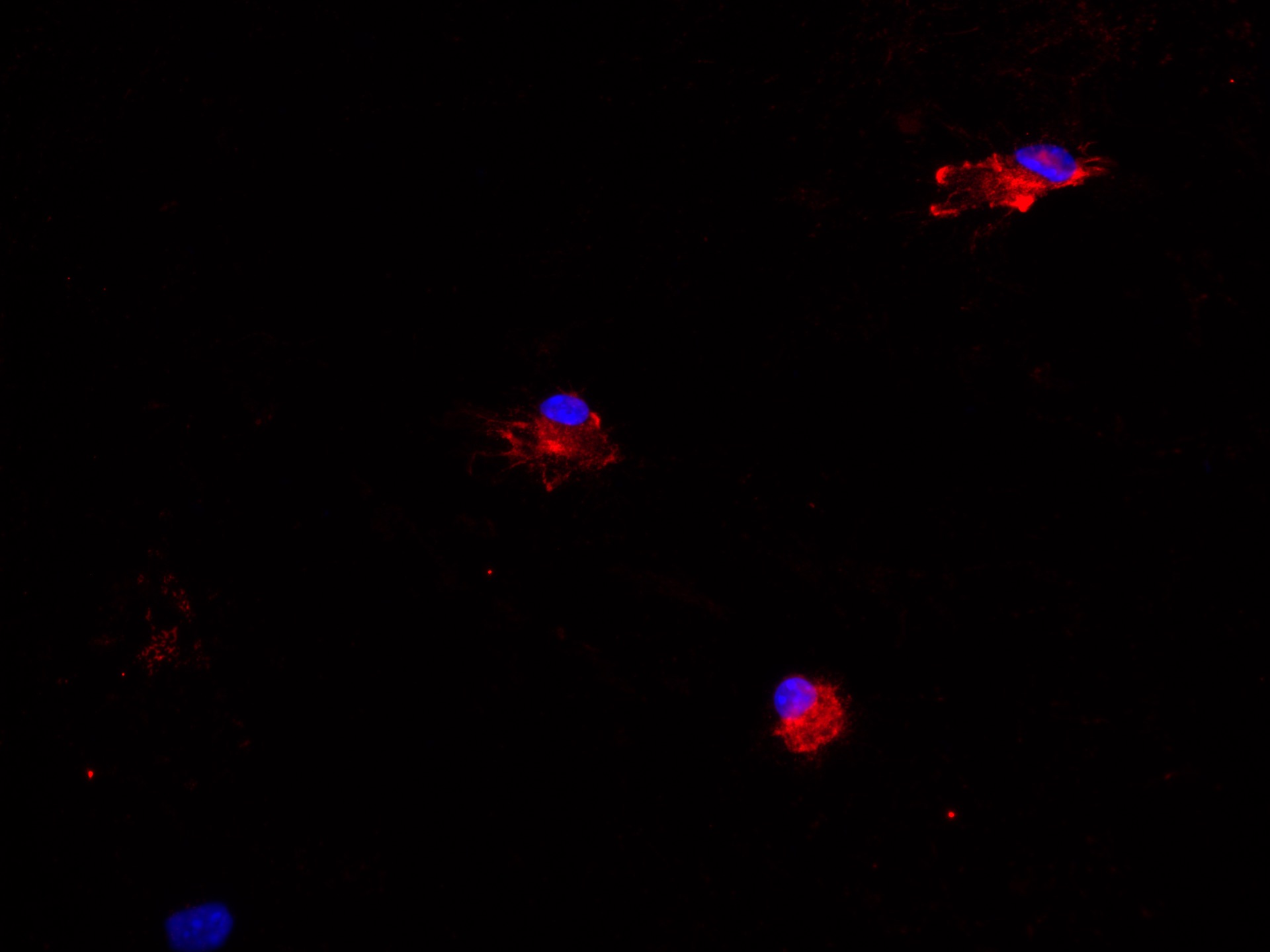

Supplement: Supplementary file 2. [file elife-102900-supp2.zip › Supplementary File 2/Raw ICC/Image_Overlay.jpeg]

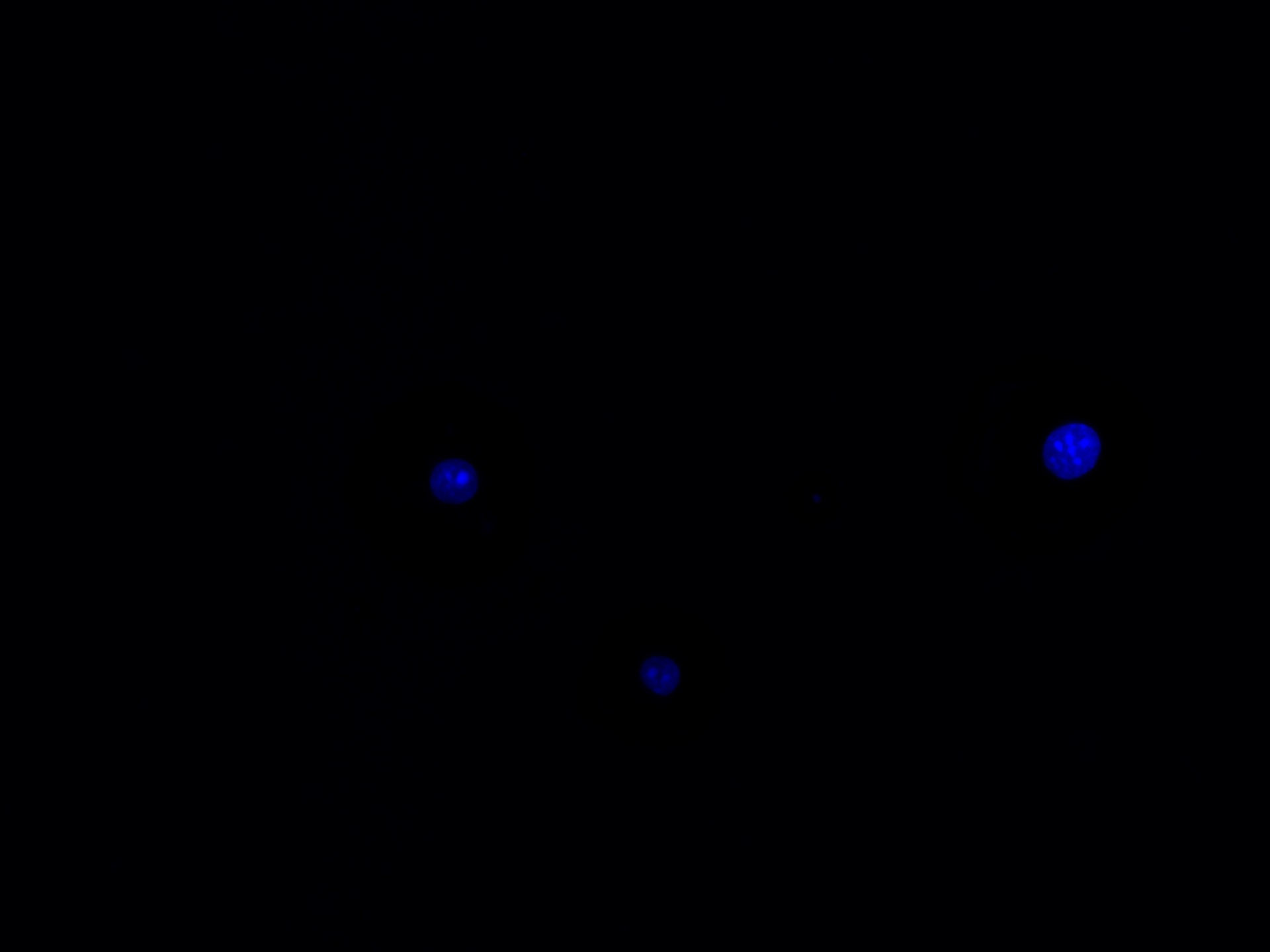

Supplement: Supplementary file 2. [file elife-102900-supp2.zip › Supplementary File 2/Raw ICC/A2 40x Z DAPI.jpeg]

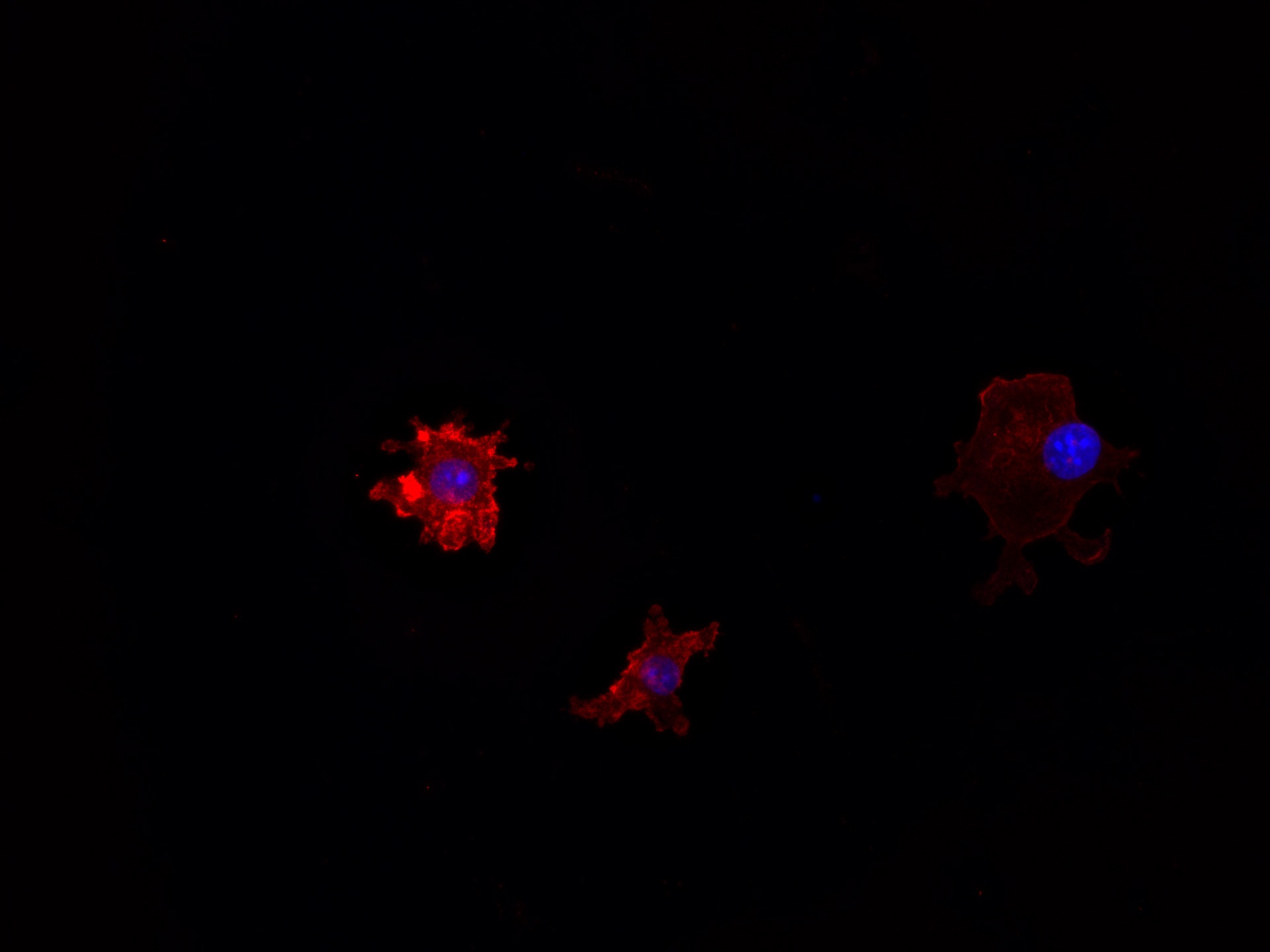

Supplement: Supplementary file 2. [file elife-102900-supp2.zip › Supplementary File 2/Raw ICC/A2 40x Z Overlay.jpeg]

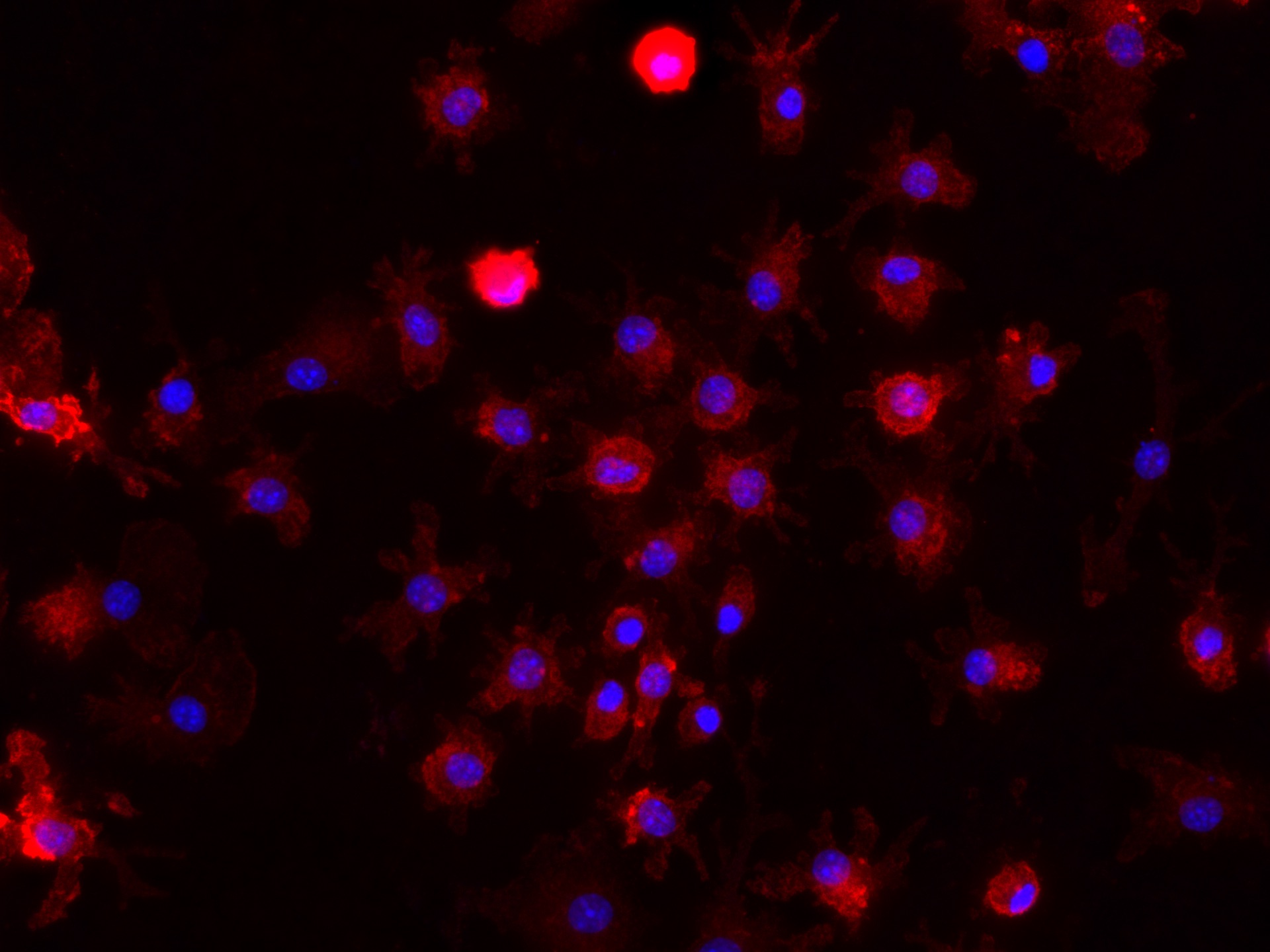

Supplement: Supplementary file 2. [file elife-102900-supp2.zip › Supplementary File 2/Raw ICC/NTC 40x Z Overlay.jpeg]

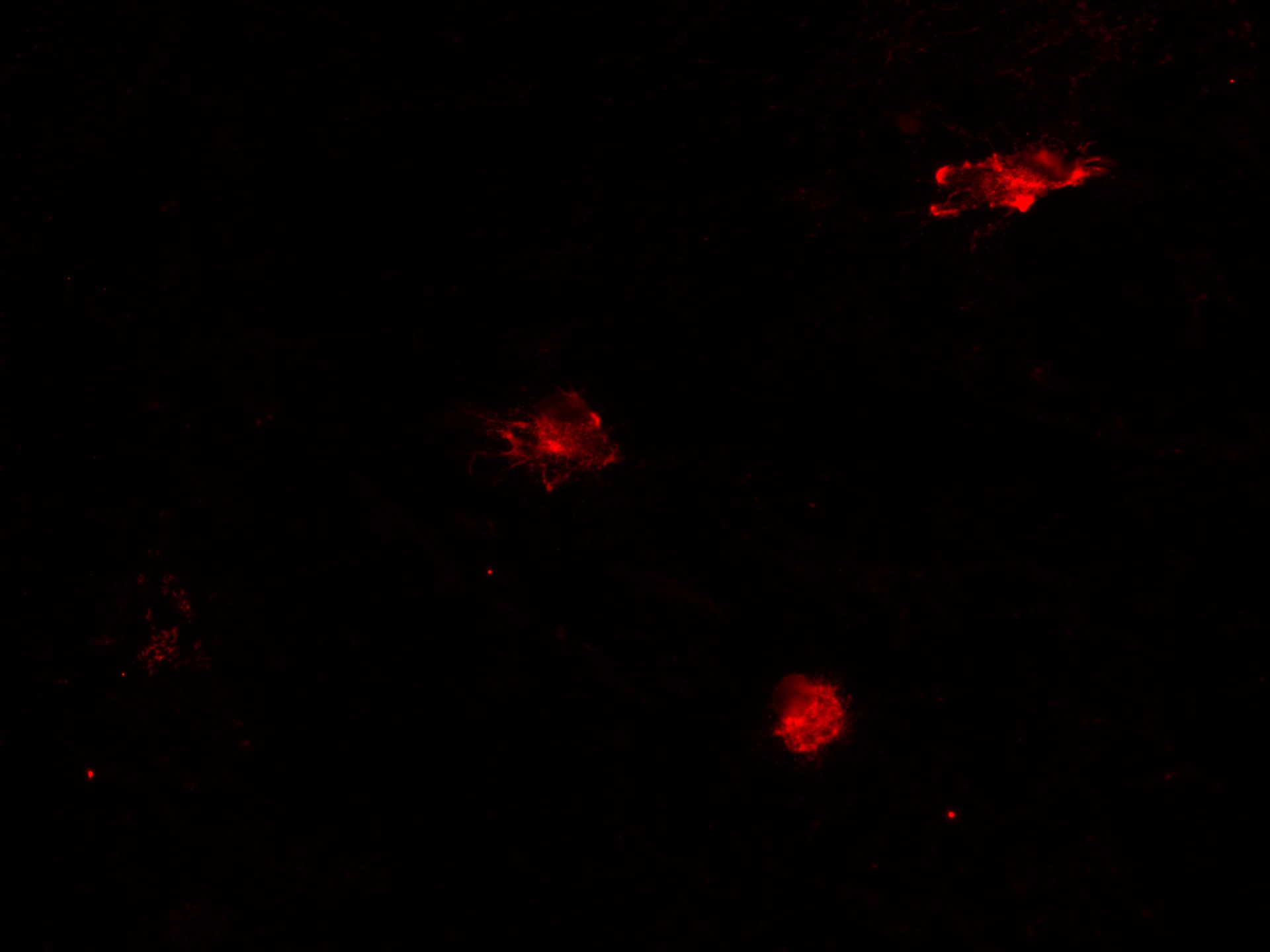

Supplement: Supplementary file 2. [file elife-102900-supp2.zip › Supplementary File 2/Raw ICC/Image_CH1.jpeg]

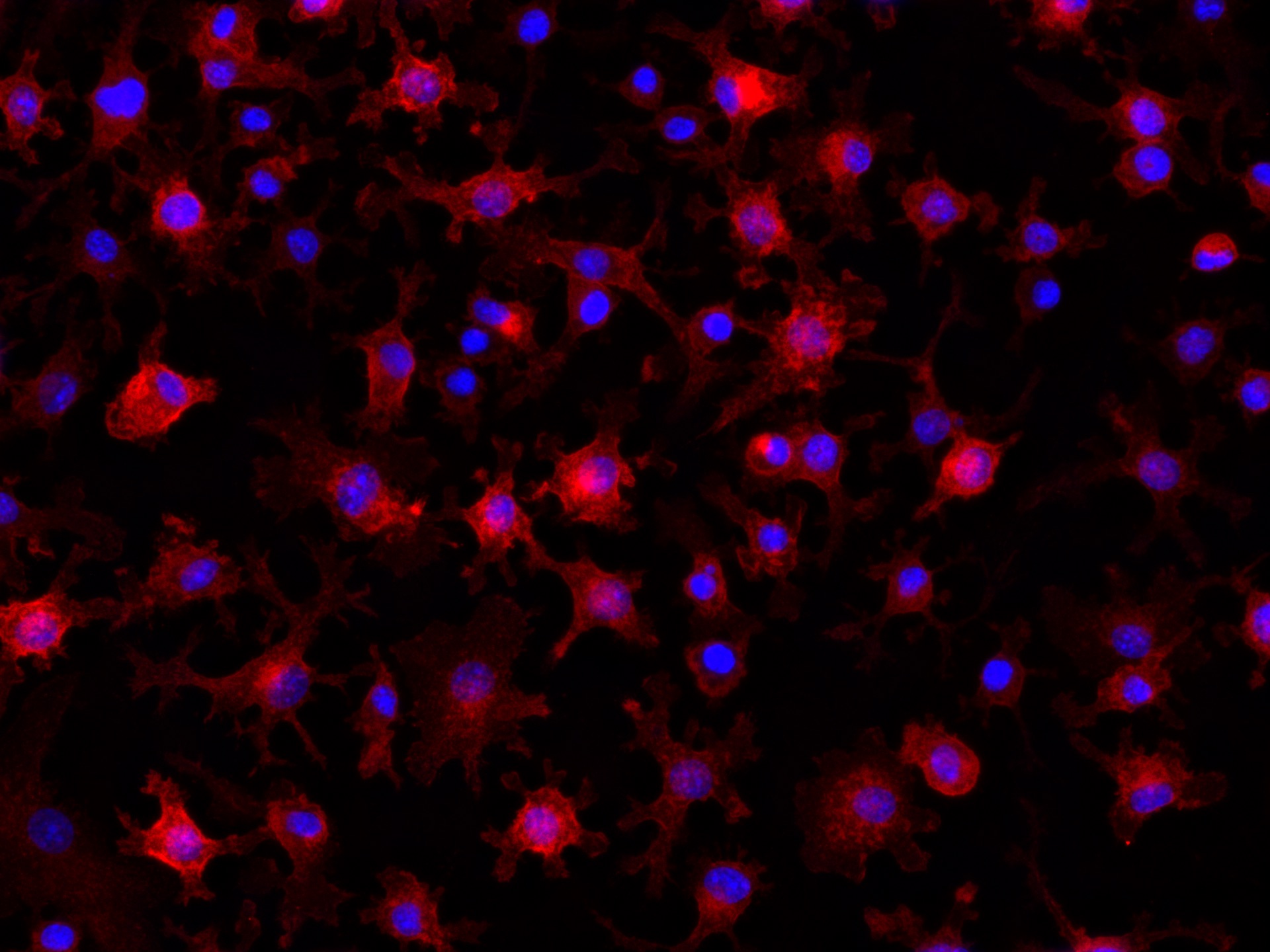

Supplement: Supplementary file 2. [file elife-102900-supp2.zip › Supplementary File 2/Raw ICC/overlay stitch.jpeg]

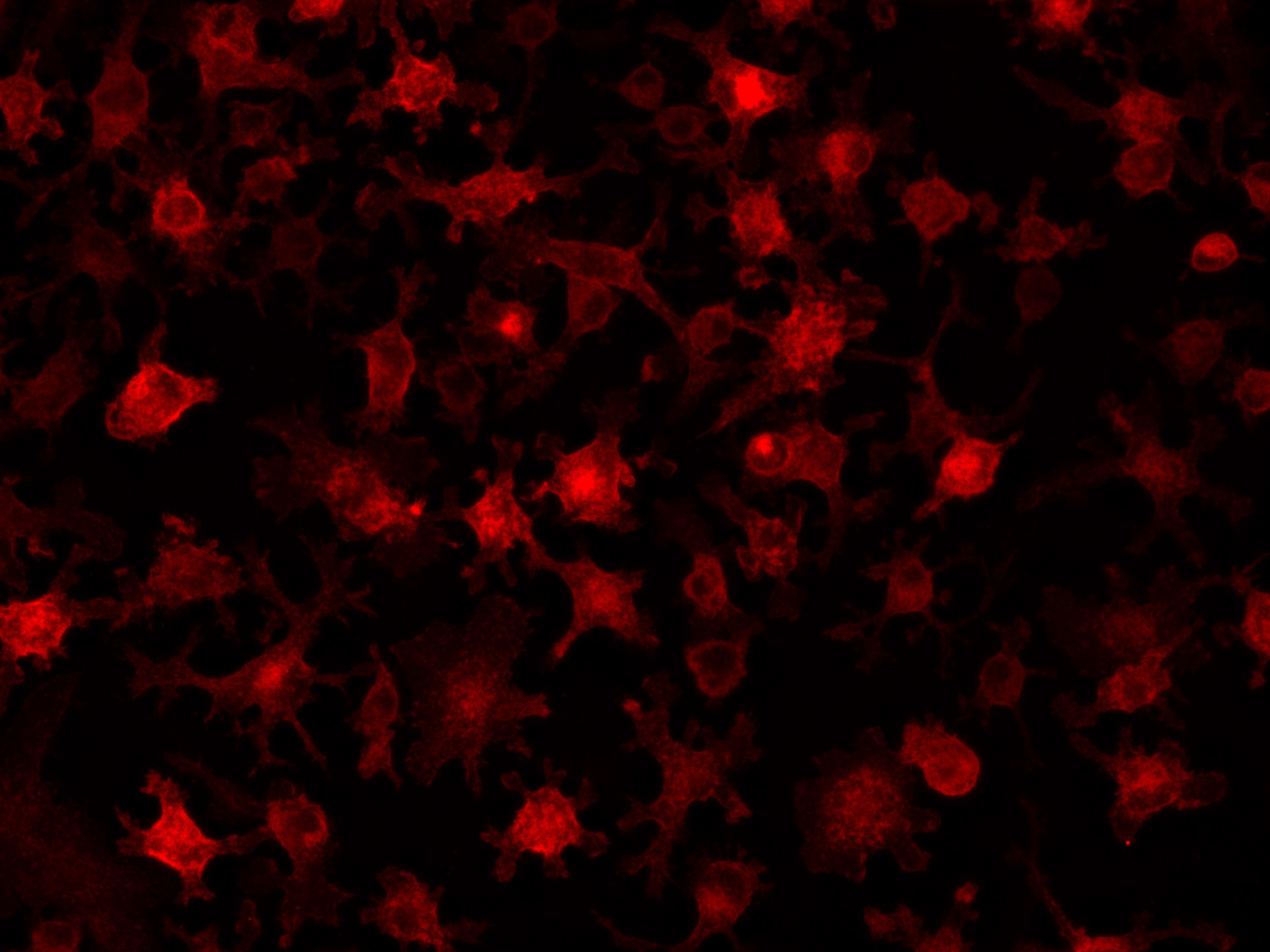

Supplement: Supplementary file 2. [file elife-102900-supp2.zip › Supplementary File 2/Raw ICC/cd11b stitch.jpeg]

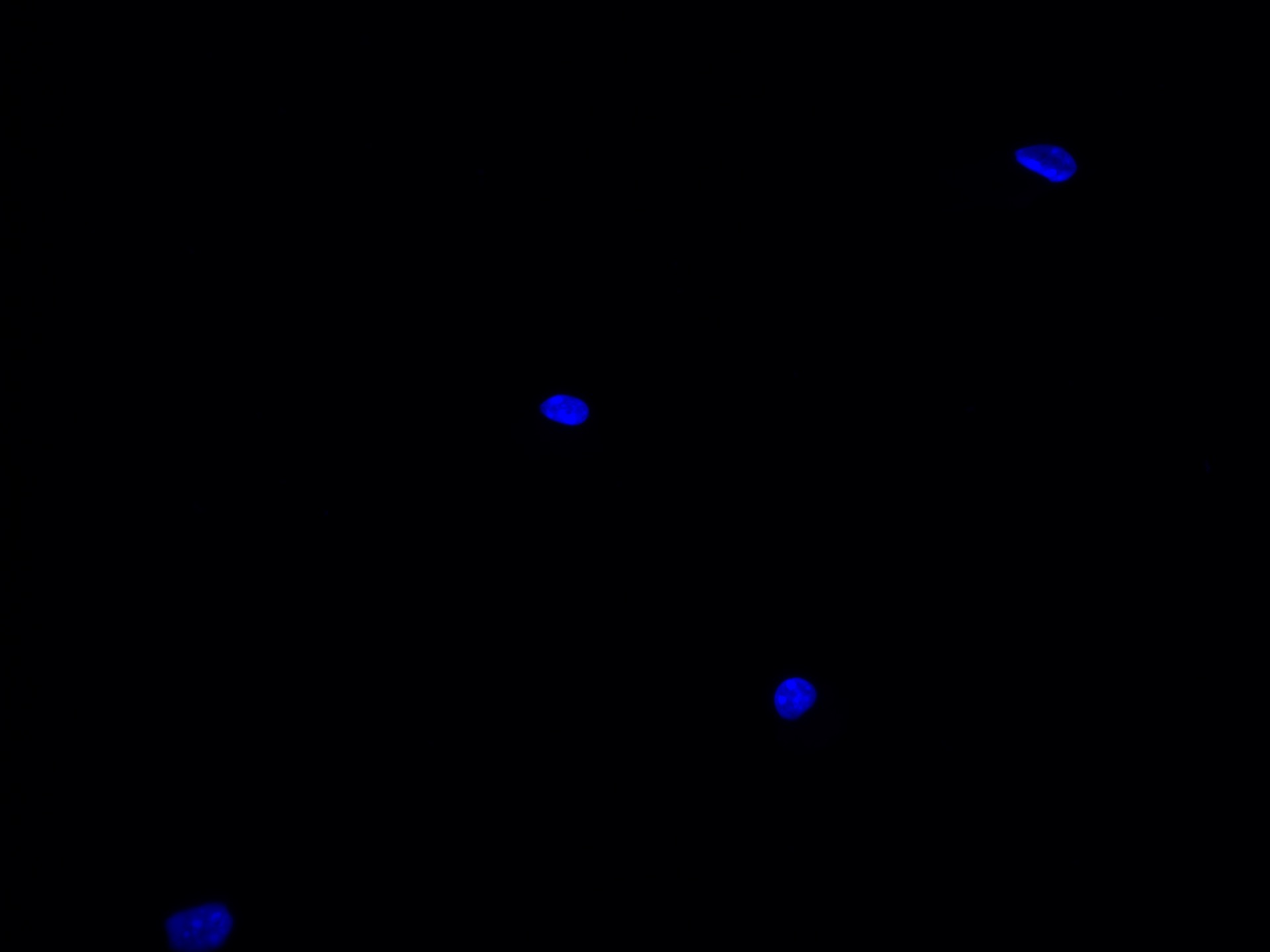

Supplement: Supplementary file 2. [file elife-102900-supp2.zip › Supplementary File 2/Raw ICC/Image_CH2.jpeg]

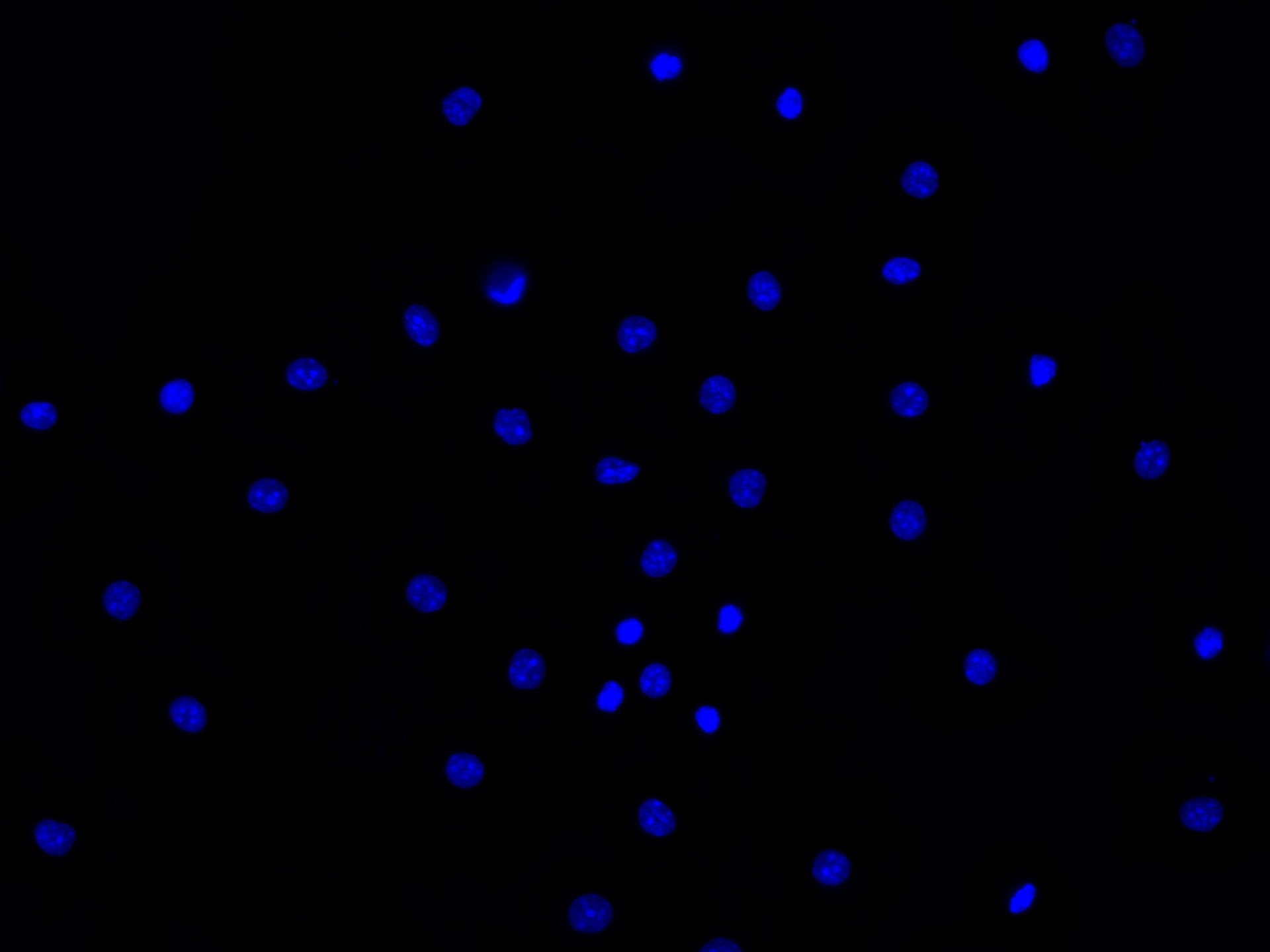

Supplement: Supplementary file 2. [file elife-102900-supp2.zip › Supplementary File 2/Raw ICC/NTC 40x Z DAPI.jpeg]

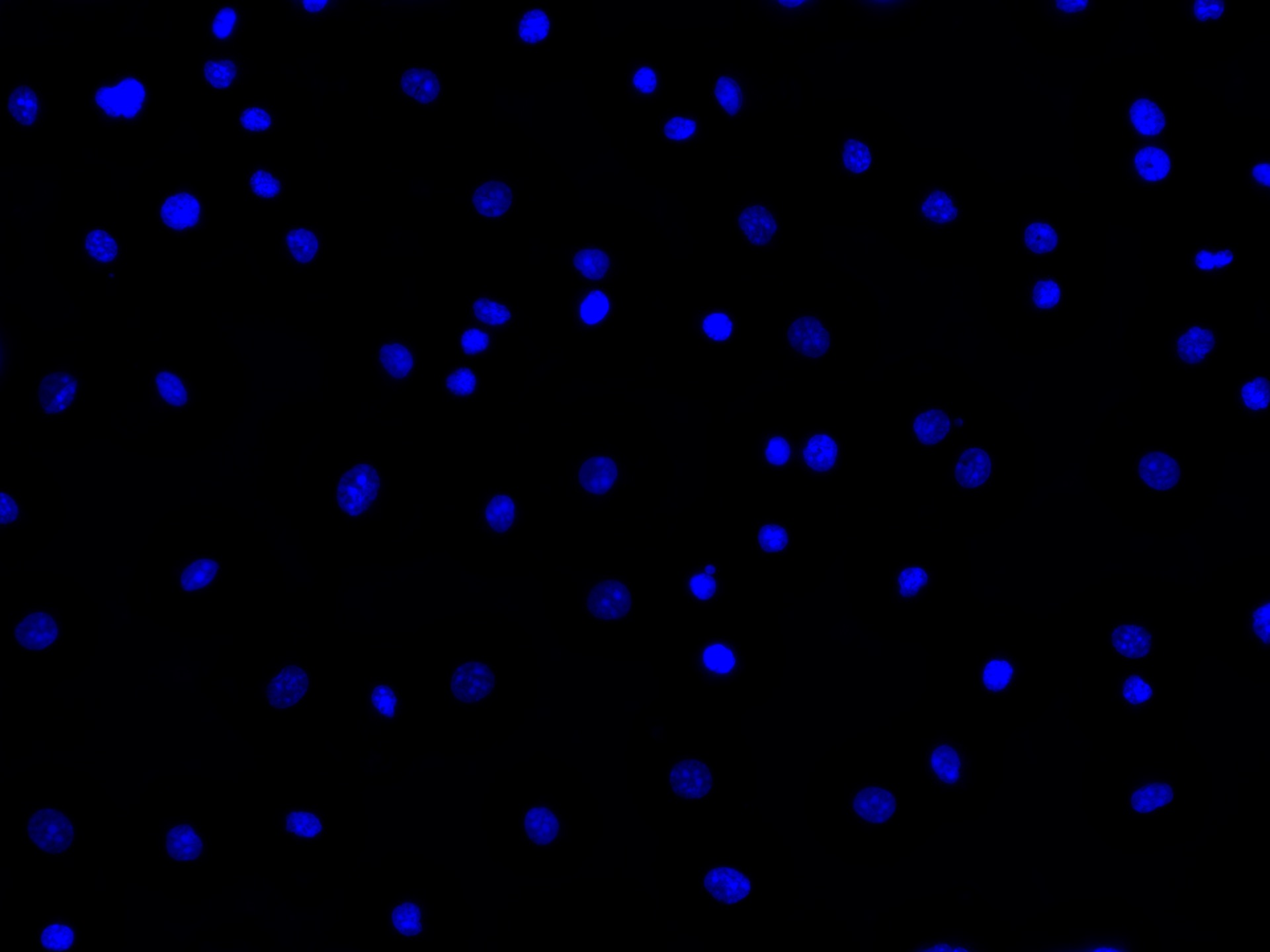

Supplement: Supplementary file 2. [file elife-102900-supp2.zip › Supplementary File 2/Raw ICC/dapi stitch.jpeg]

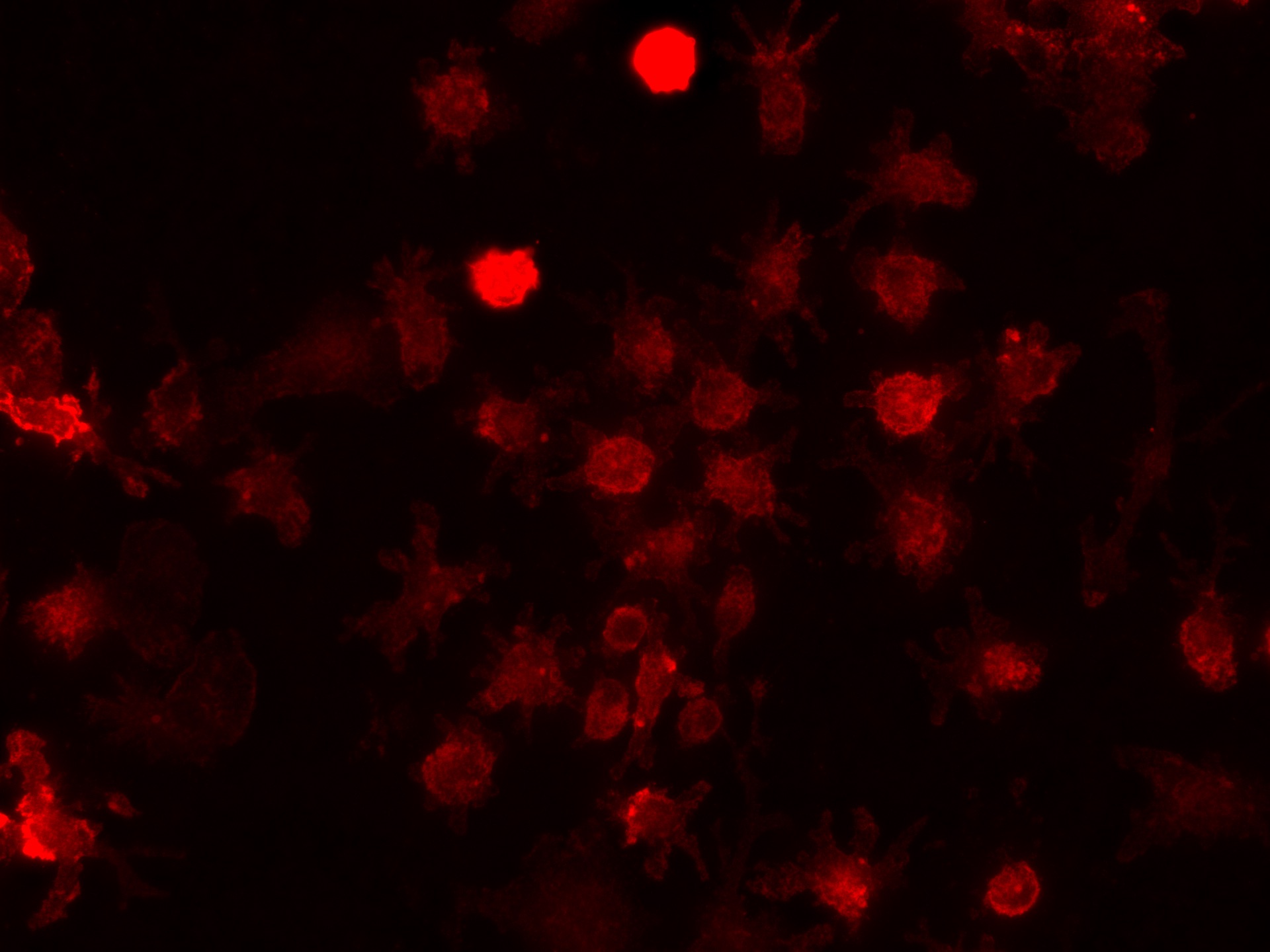

Supplement: Supplementary file 2. [file elife-102900-supp2.zip › Supplementary File 2/Raw ICC/NTC 40x Z Cd11b.jpeg]

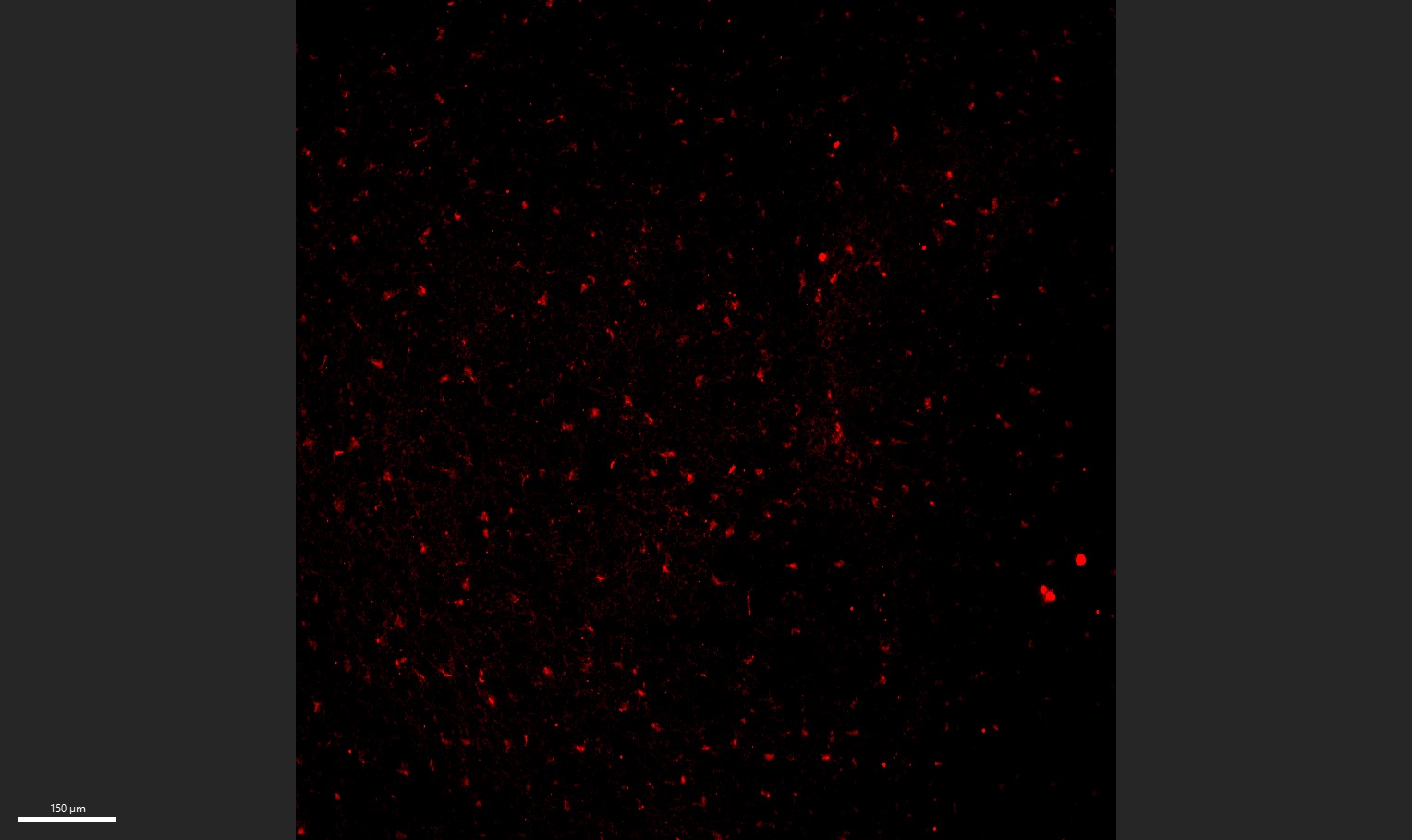

Supplement: Supplementary file 2. [file elife-102900-supp2.zip › Supplementary File 2/Raw RNAScope/1172_full_d1113h_10x_02_2024-07-30_14.00.56_2024-08-02T11-51-03.362.jpeg]

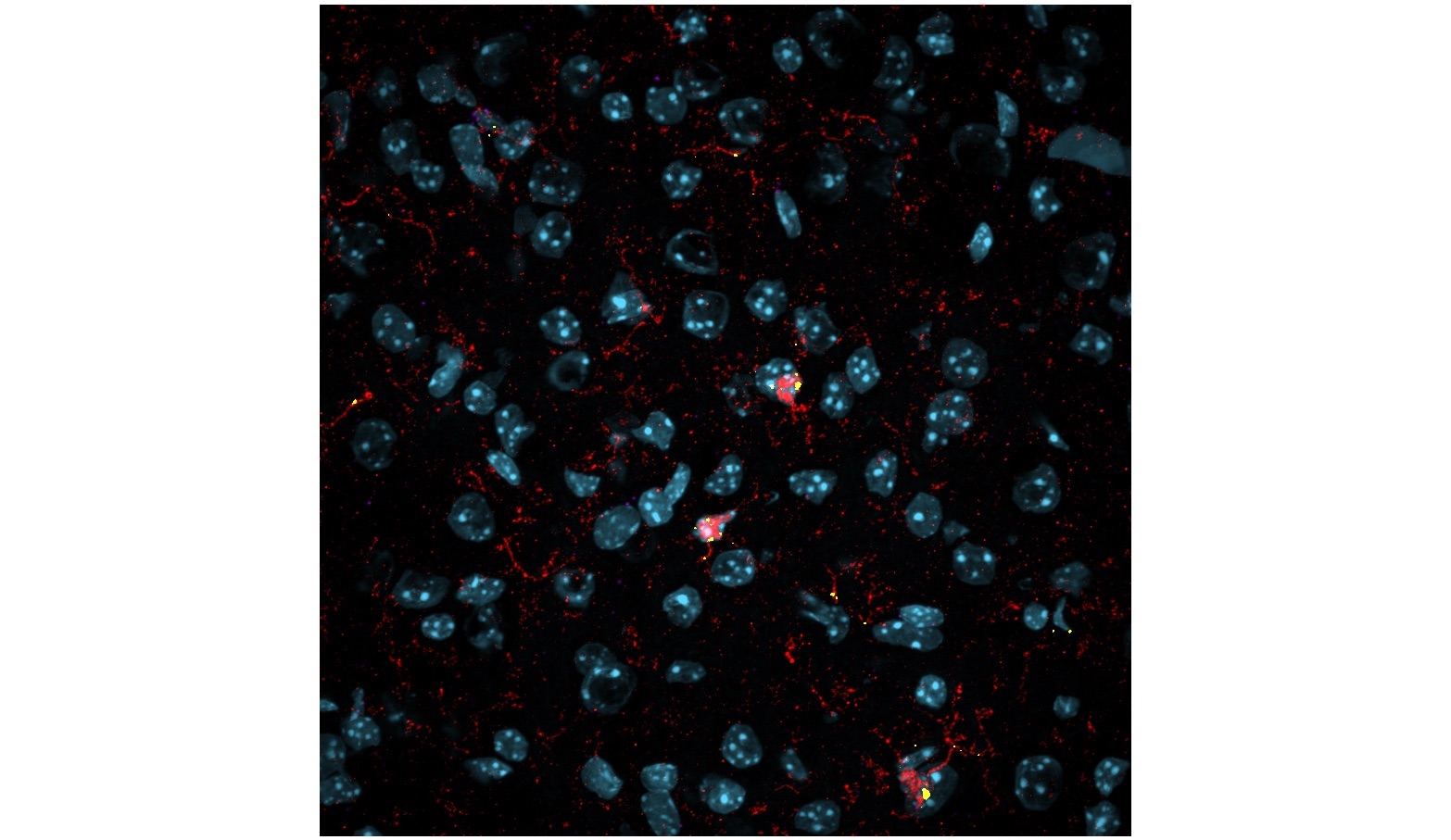

Supplement: Supplementary file 2. [file elife-102900-supp2.zip › Supplementary File 2/Raw RNAScope/1214_tamsham_60x_02-1_2024-07-30_15.00.14_2024-08-06T15-11-36.867.jpeg]

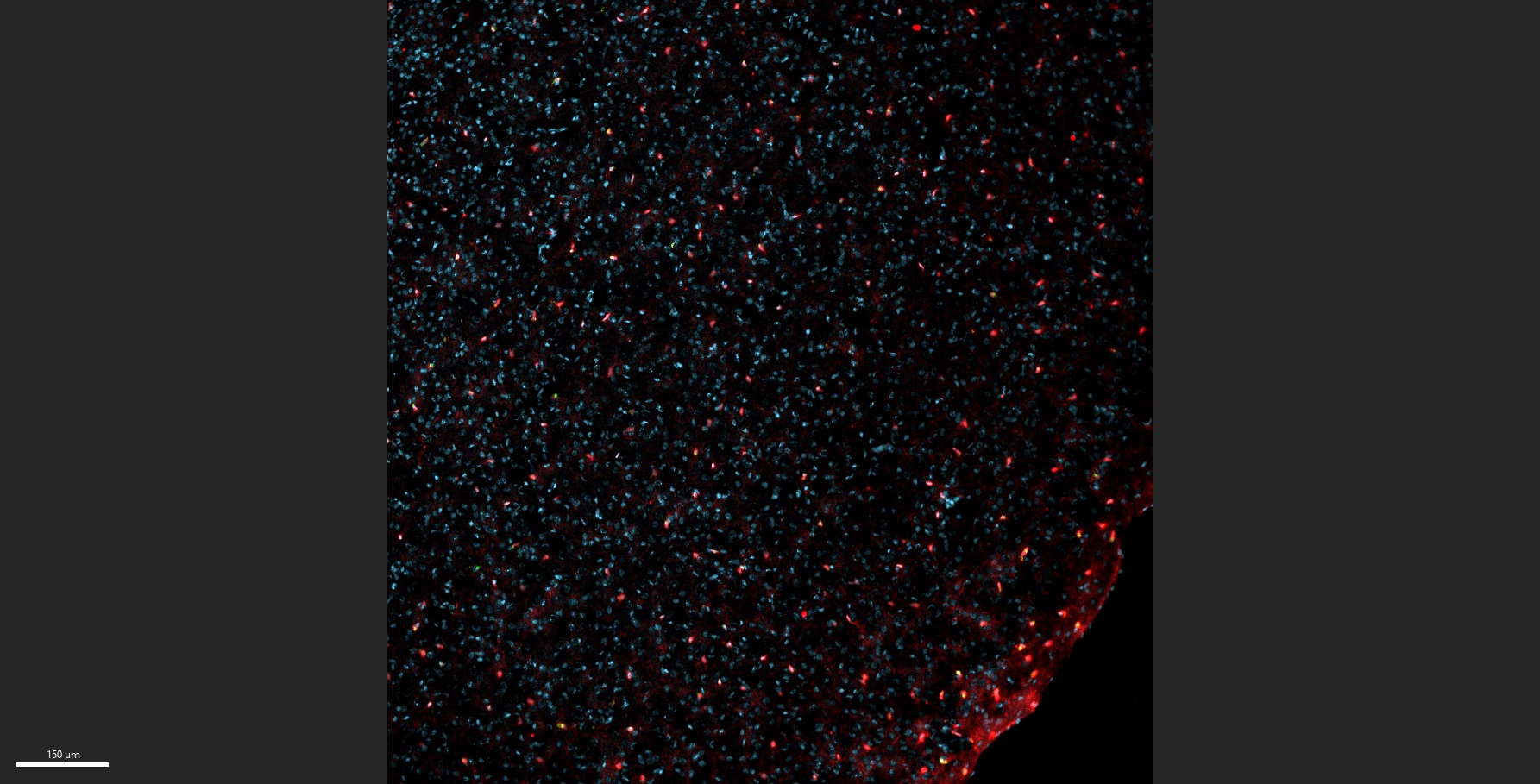

Supplement: Supplementary file 2. [file elife-102900-supp2.zip › Supplementary File 2/Raw RNAScope/1214_tamsham_10x_02_2024-07-30_14.13.16_2024-08-05T16-17-39.351.jpeg]

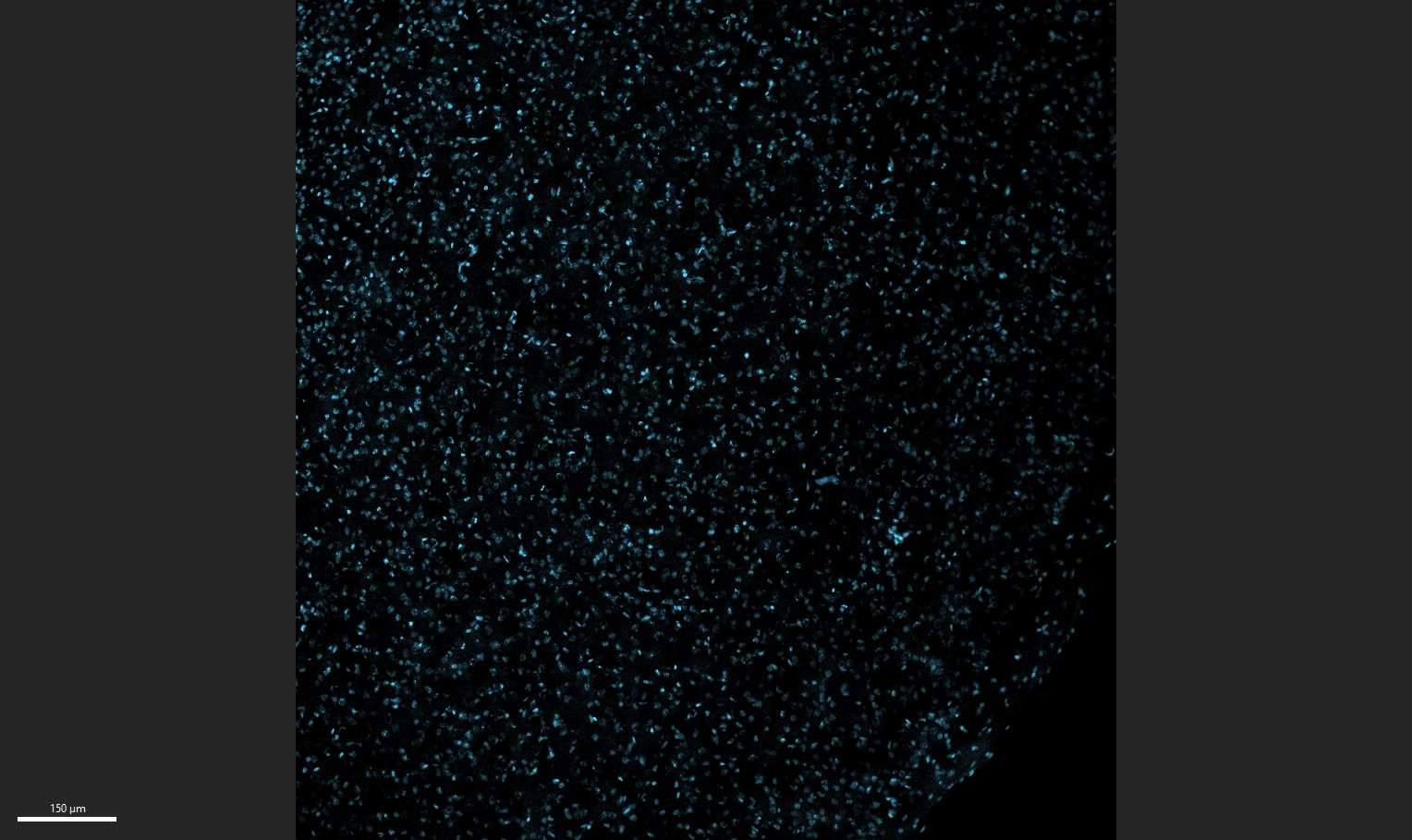

Supplement: Supplementary file 2. [file elife-102900-supp2.zip › Supplementary File 2/Raw RNAScope/1214_tamsham_10x_02_2024-07-30_14.13.16_2024-08-02T11-56-44.433.jpeg]

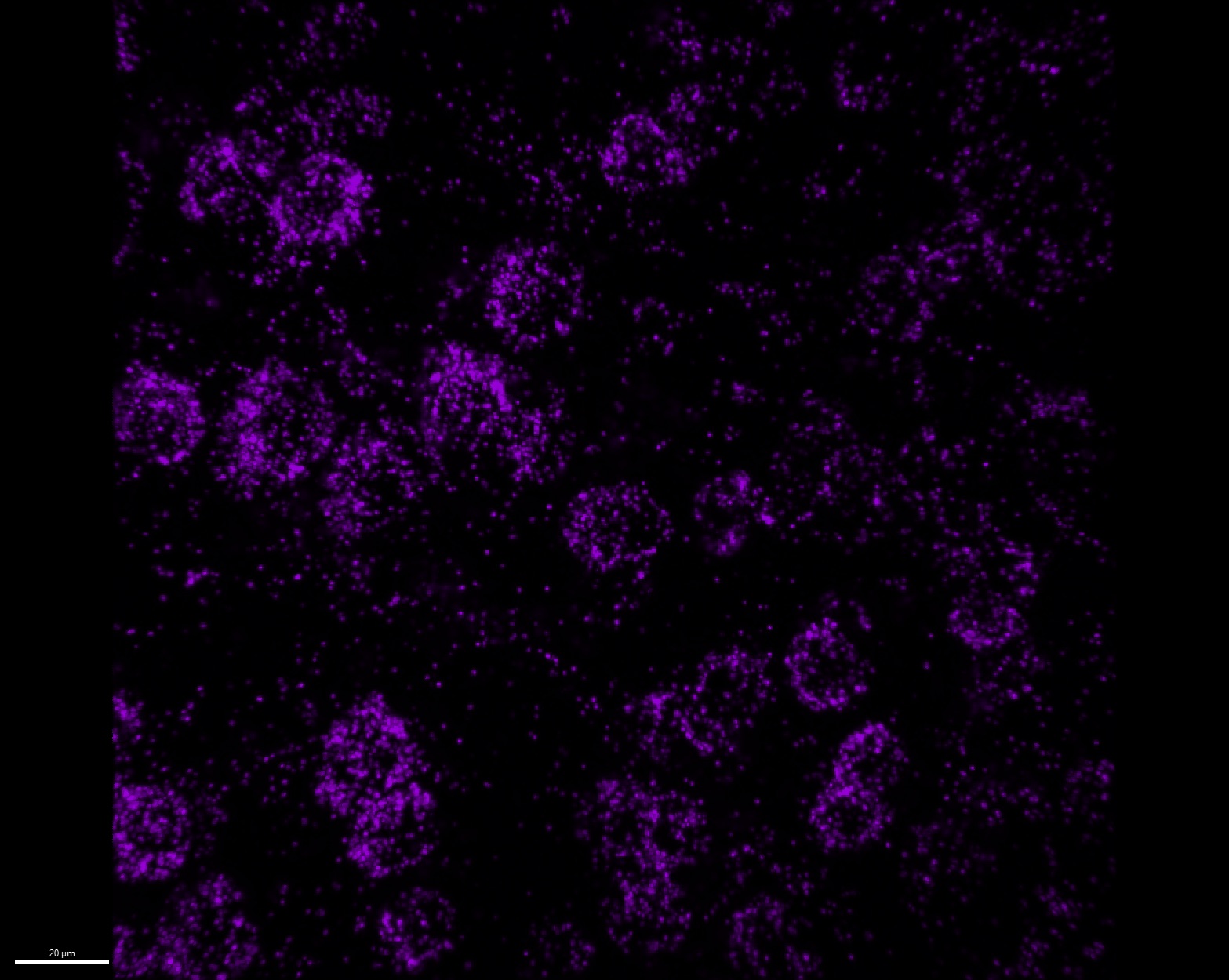

Supplement: Supplementary file 2. [file elife-102900-supp2.zip › Supplementary File 2/Raw RNAScope/5_2025-03-21_11.57.59_isg15.jpeg]

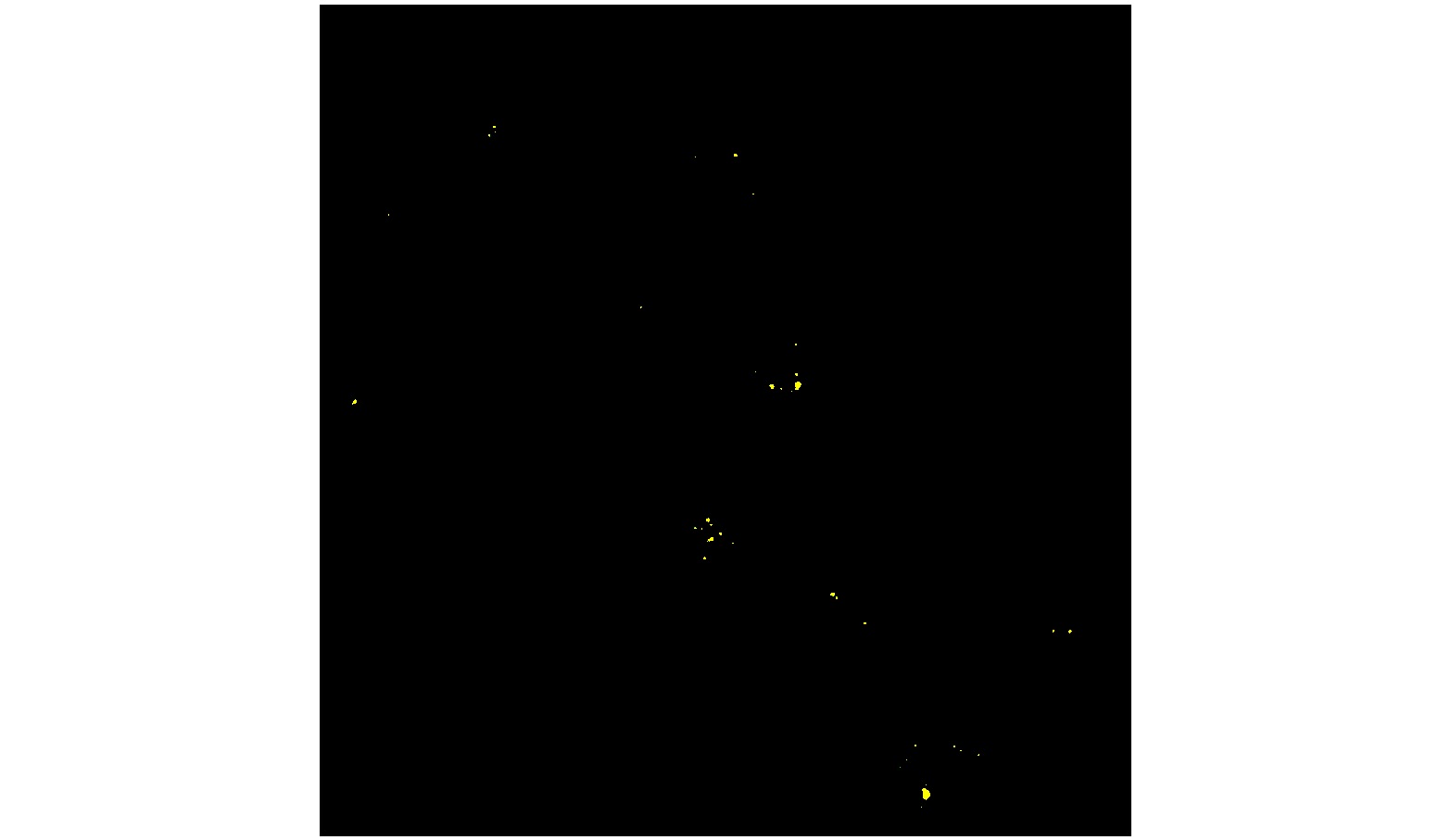

Supplement: Supplementary file 2. [file elife-102900-supp2.zip › Supplementary File 2/Raw RNAScope/1214_tamsham_60x_02-1_2024-07-30_15.00.14_2024-08-06T15-11-46.512.jpeg]

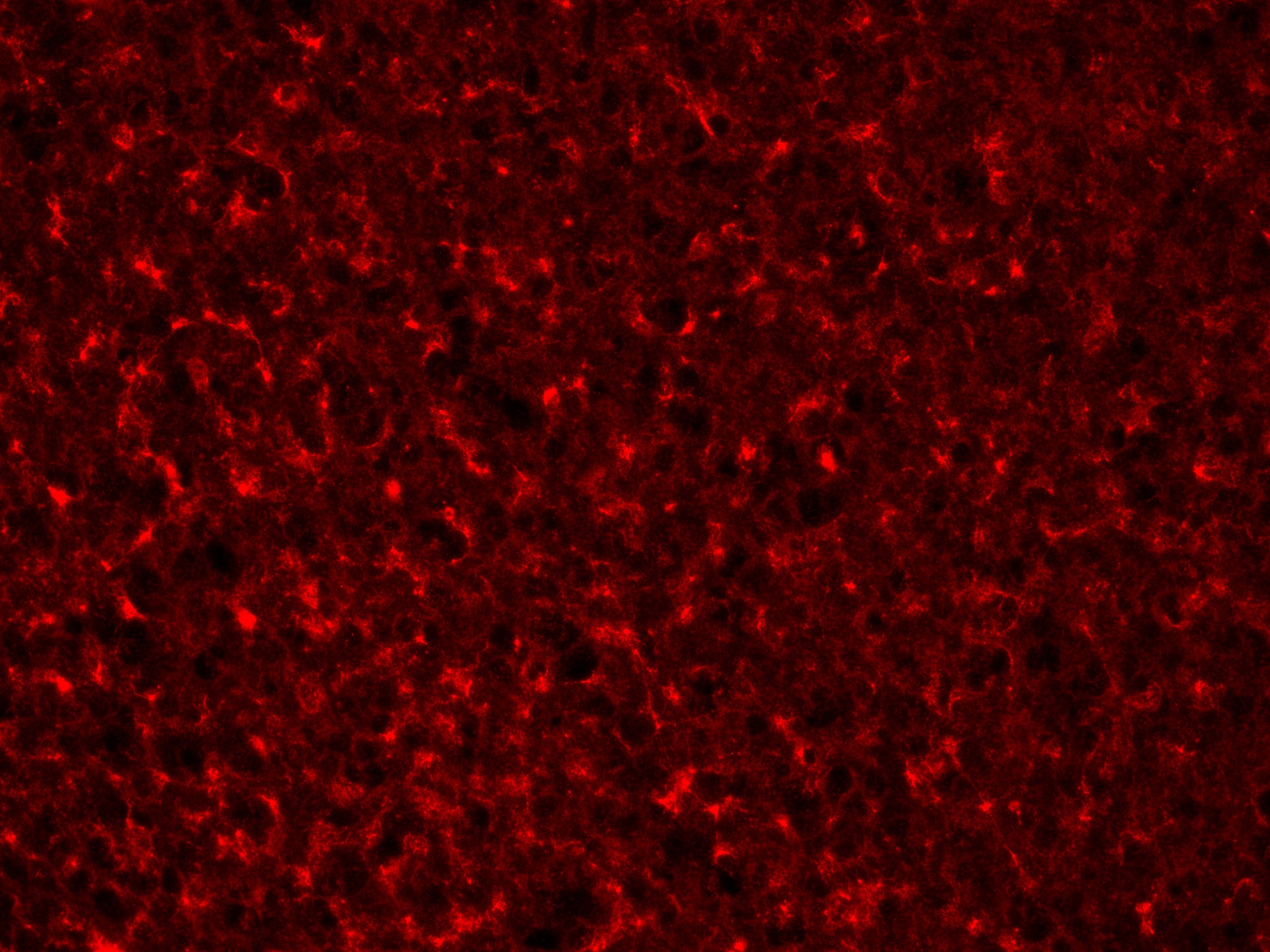

Supplement: Supplementary file 2. [file elife-102900-supp2.zip › Supplementary File 2/Raw RNAScope/1216 Iba.jpeg]

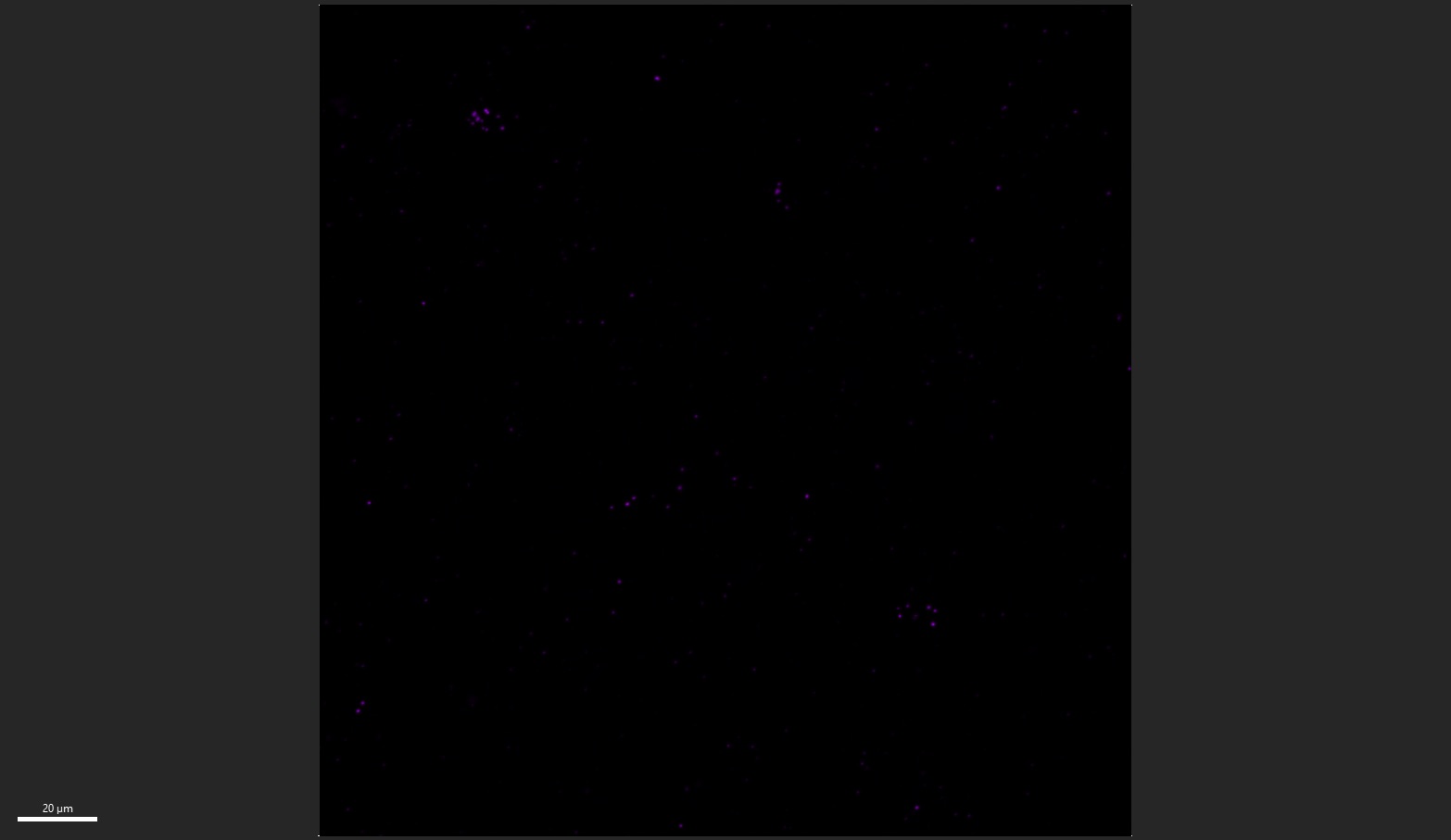

Supplement: Supplementary file 2. [file elife-102900-supp2.zip › Supplementary File 2/Raw RNAScope/1214_tamsham_60x_02-1_2024-07-30_15.00.14_2024-08-06T15-11-55.008.jpeg]

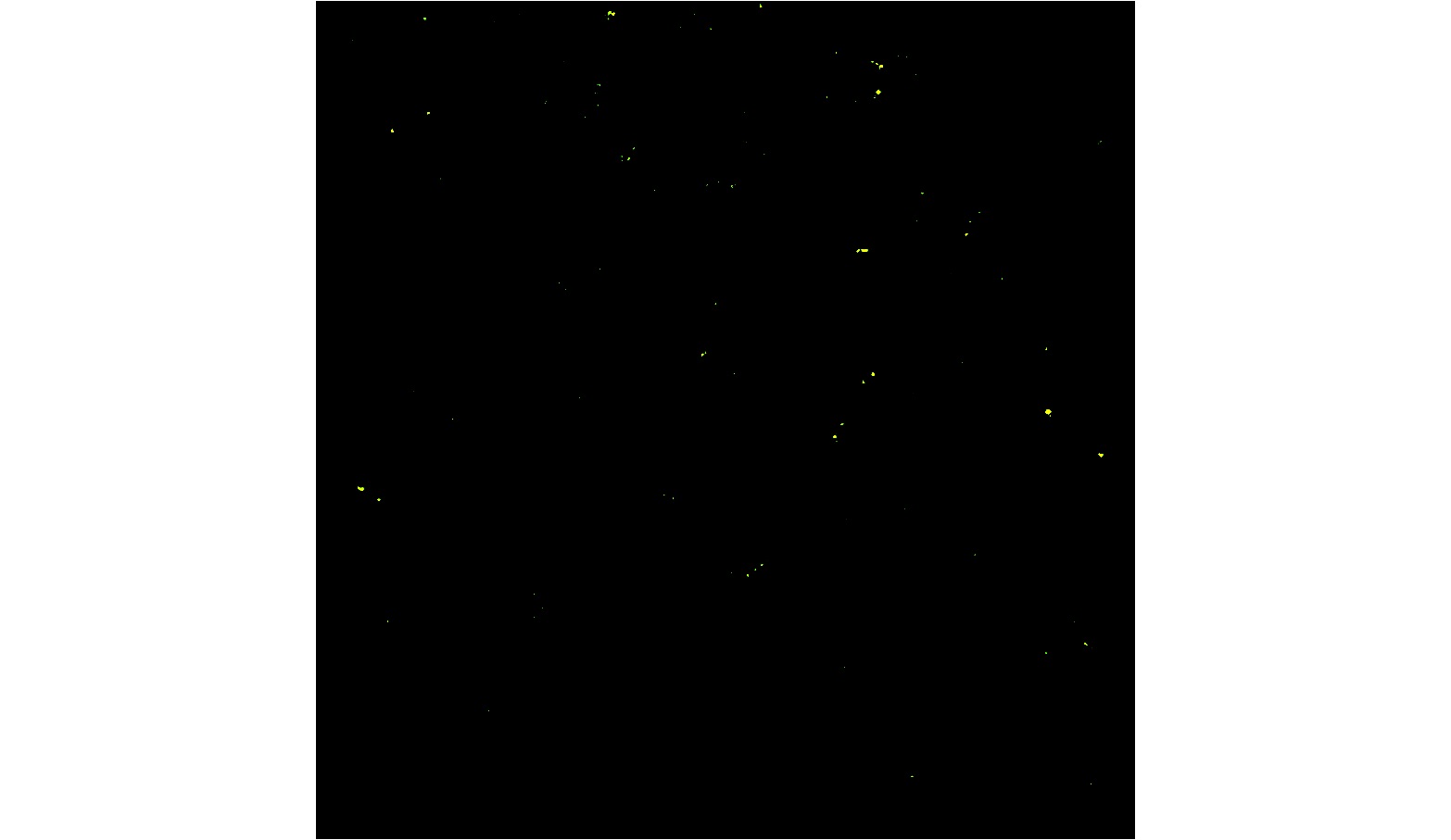

Supplement: Supplementary file 2. [file elife-102900-supp2.zip › Supplementary File 2/Raw RNAScope/1188_ict_d1113h_60x_04_2024-08-02_10.32.21_2024-08-05T16-40-59.493.jpeg]

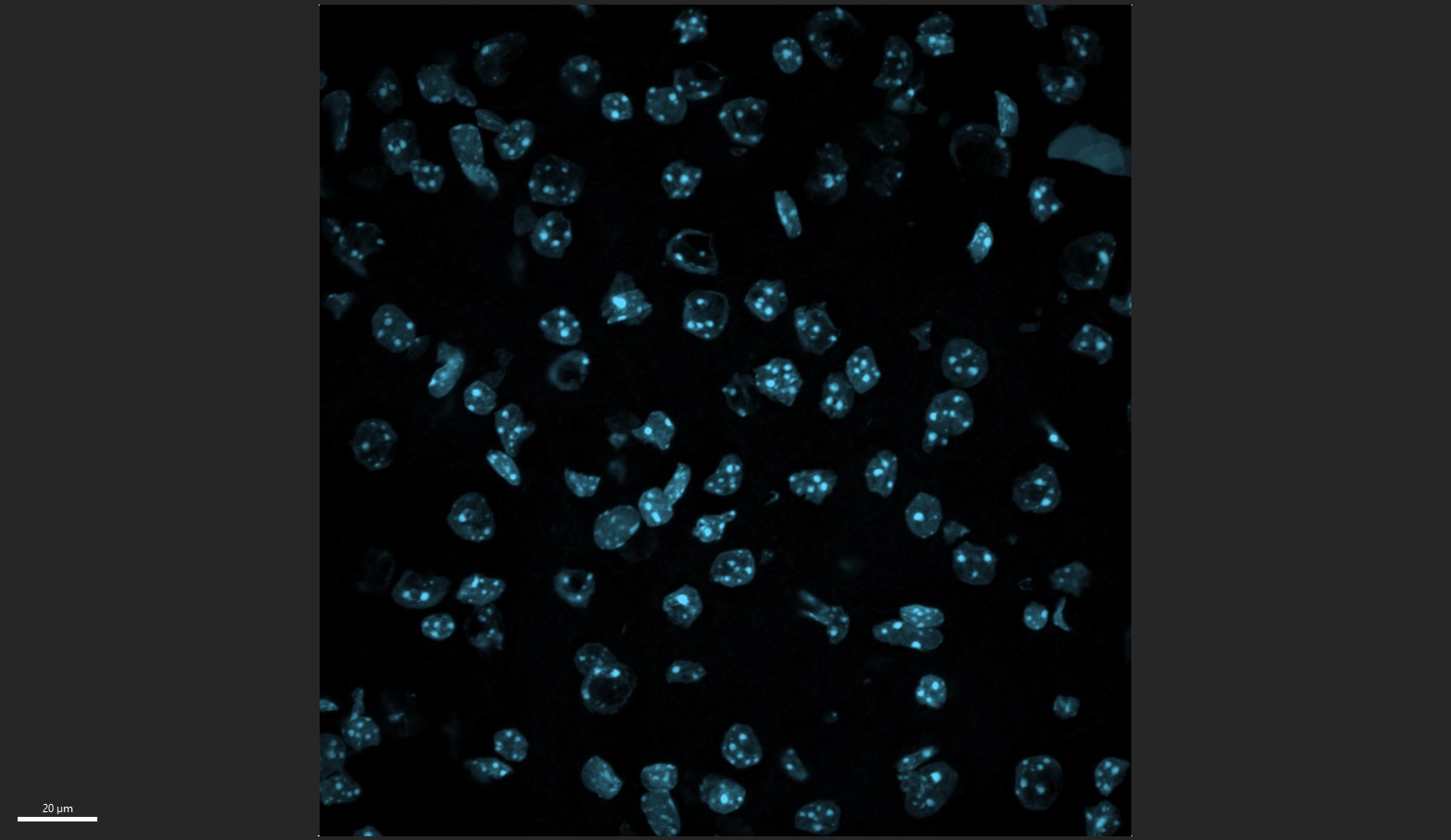

Supplement: Supplementary file 2. [file elife-102900-supp2.zip › Supplementary File 2/Raw RNAScope/1214_tamsham_60x_02-1_2024-07-30_15.00.14_2024-08-06T15-11-42.560.jpeg]

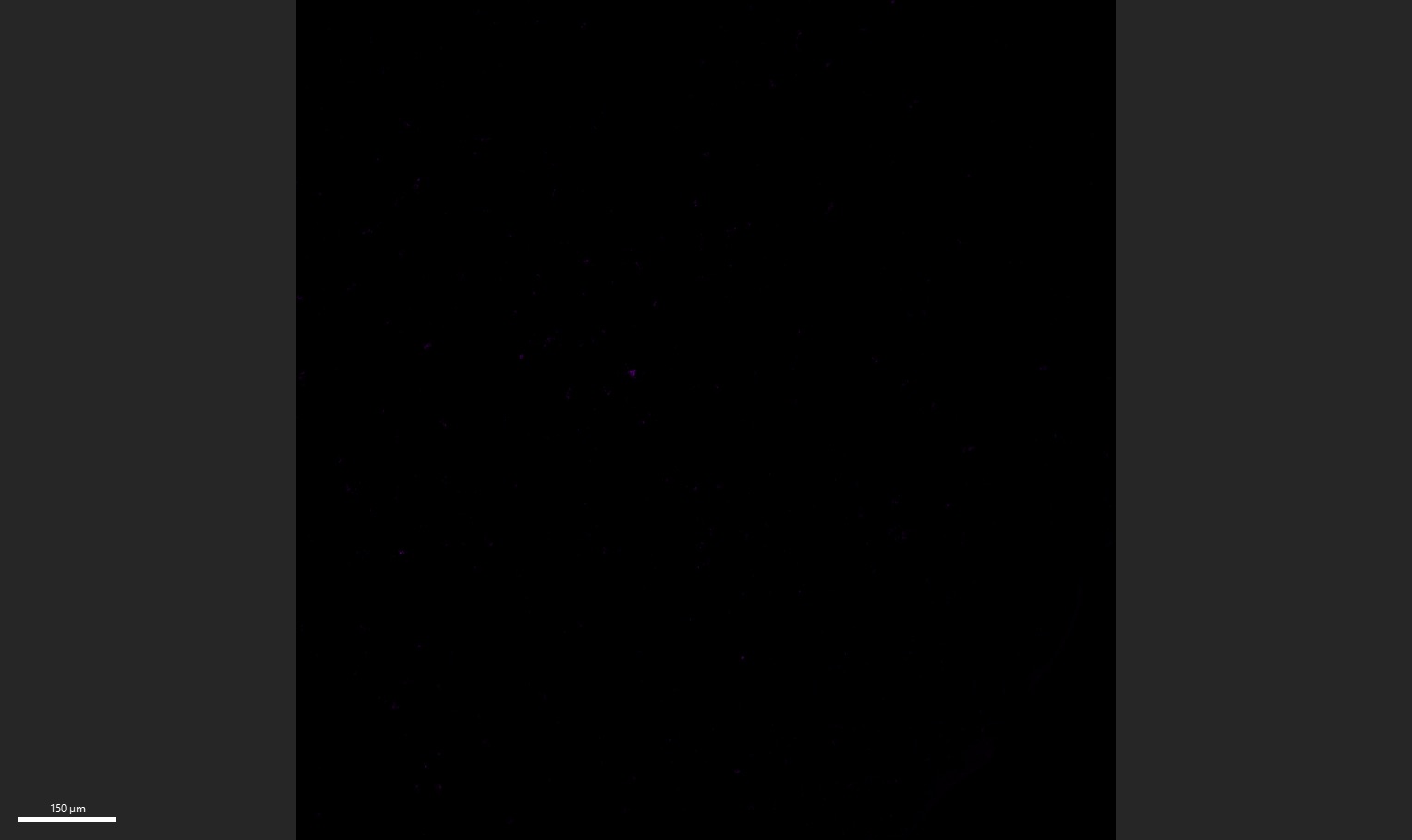

Supplement: Supplementary file 2. [file elife-102900-supp2.zip › Supplementary File 2/Raw RNAScope/1214_tamsham_10x_02_2024-07-30_14.13.16_2024-08-02T11-56-56.857.jpeg]

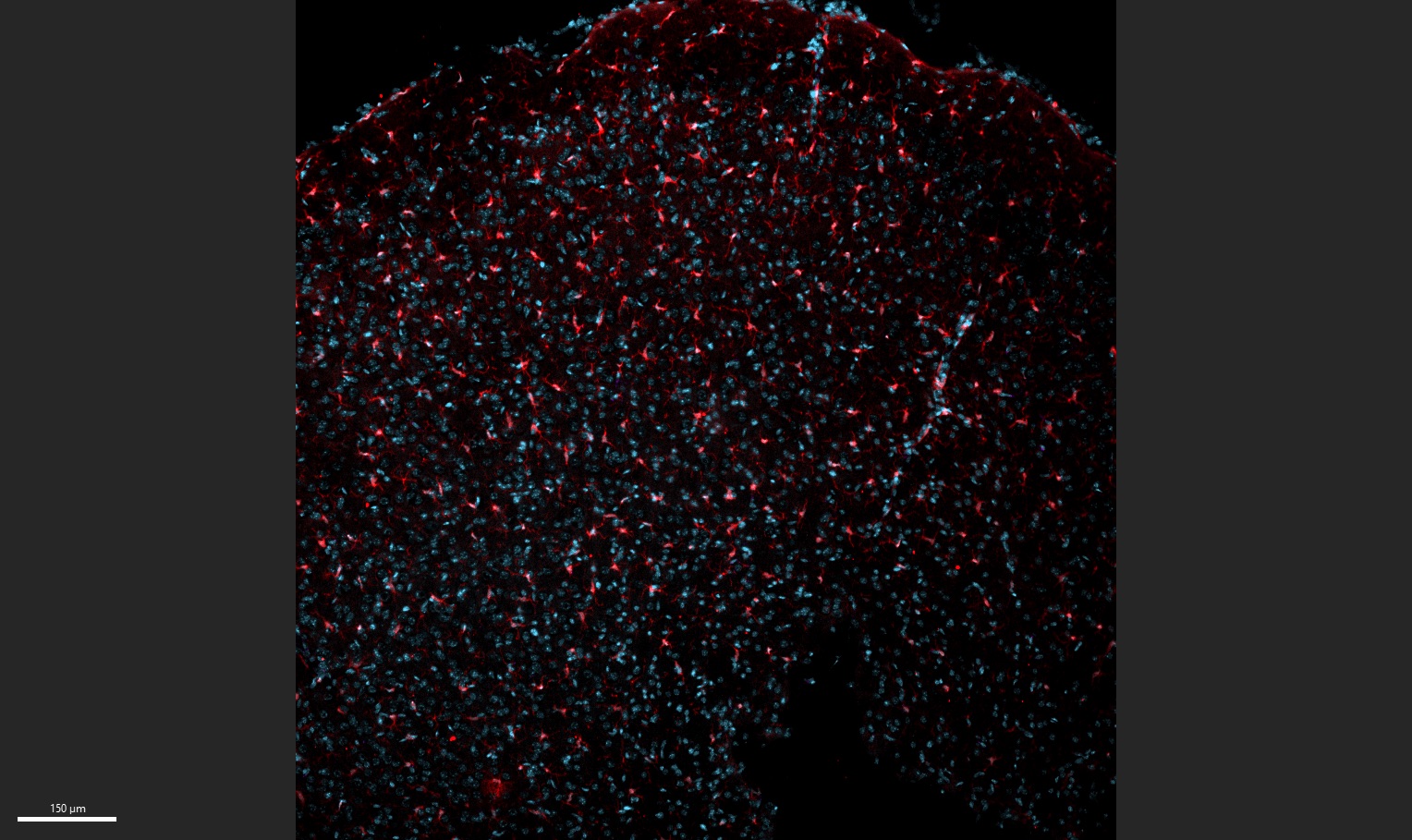

Supplement: Supplementary file 2. [file elife-102900-supp2.zip › Supplementary File 2/Raw RNAScope/1184_ict_wtb6_10x_02_2024-07-30_15.33.25_2024-08-02T11-59-11.393.jpeg]

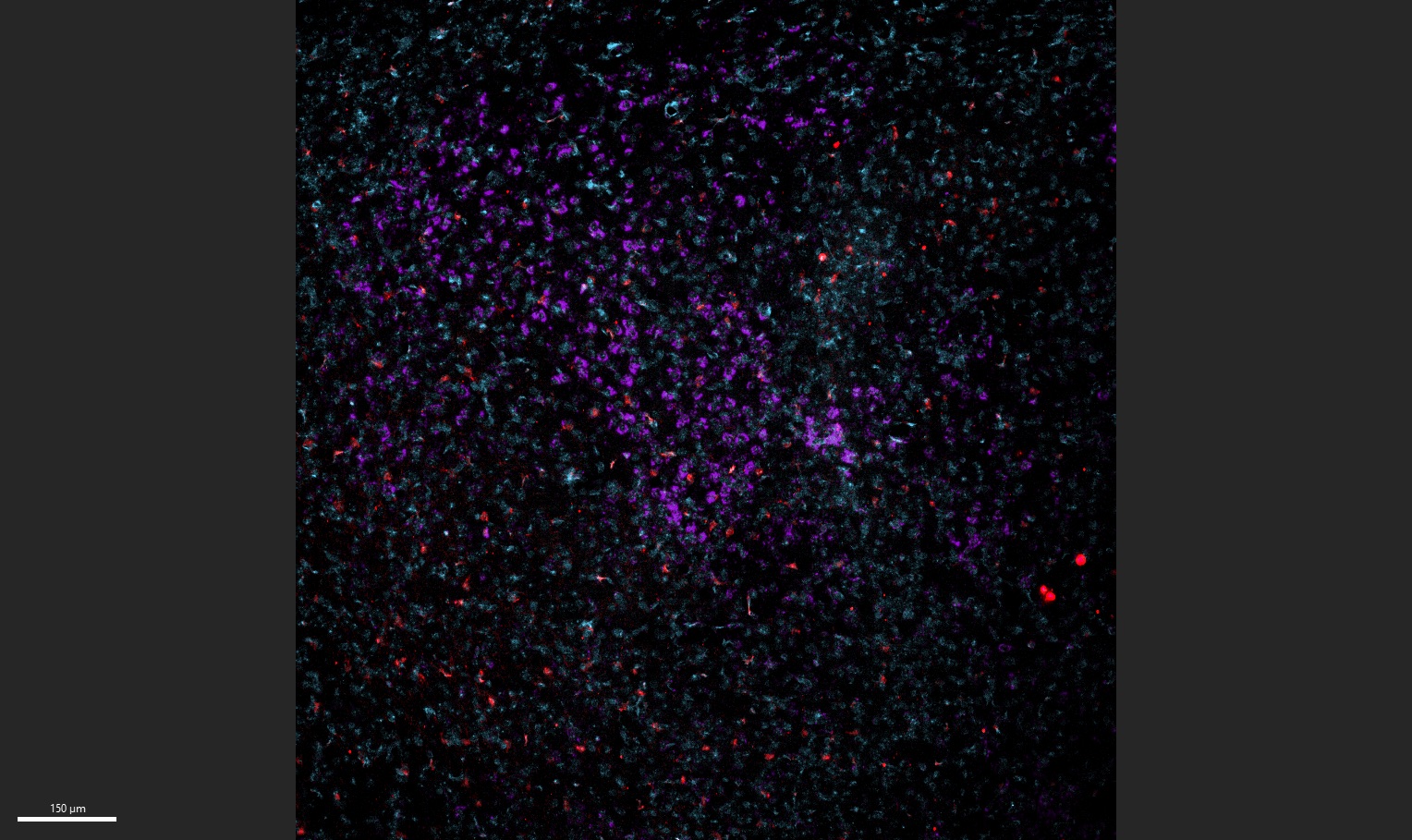

Supplement: Supplementary file 2. [file elife-102900-supp2.zip › Supplementary File 2/Raw RNAScope/1172_full_d1113h_10x_02_2024-07-30_14.00.56_2024-08-02T11-50-03.993.jpeg]

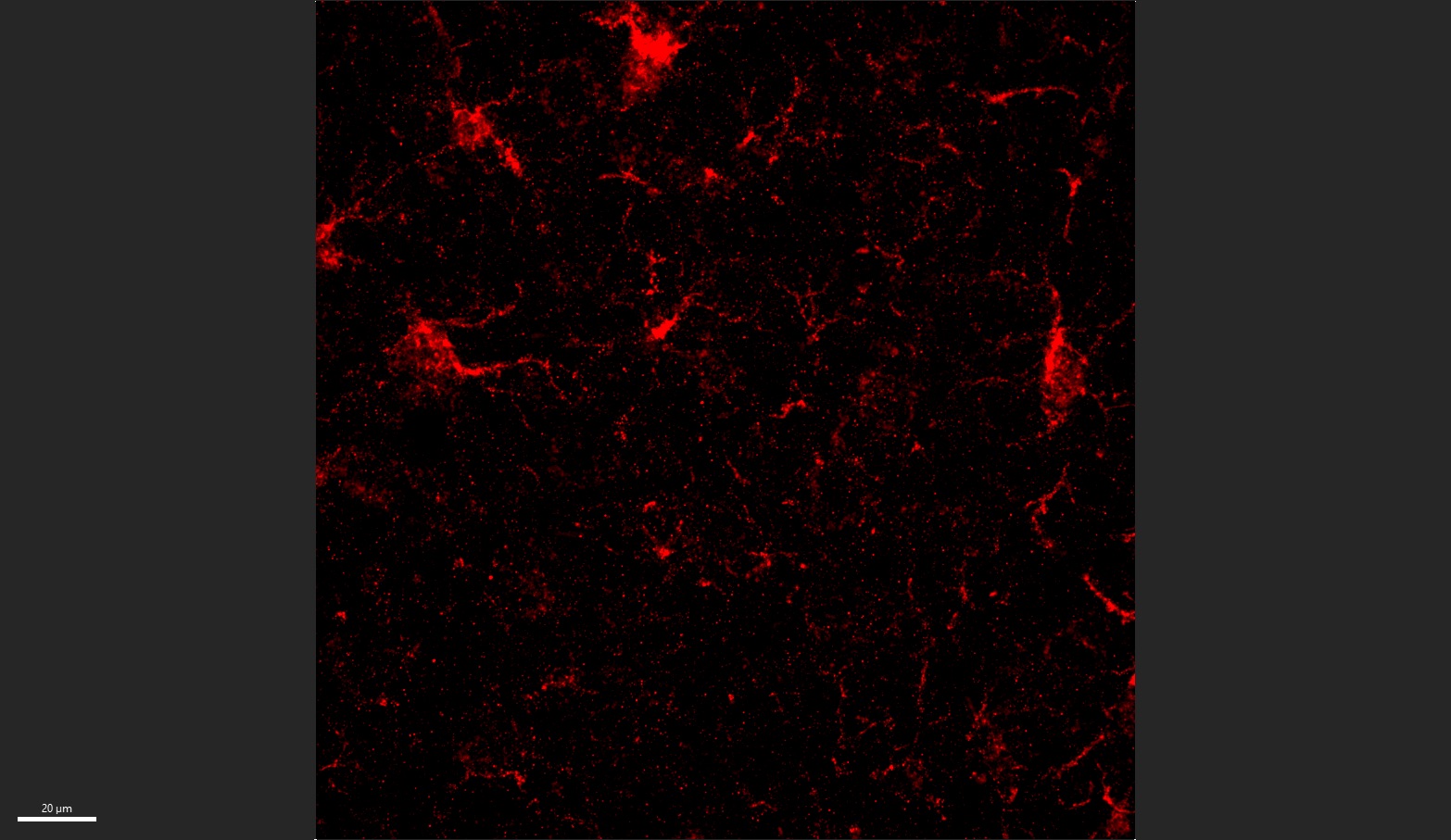

Supplement: Supplementary file 2. [file elife-102900-supp2.zip › Supplementary File 2/Raw RNAScope/1188_ict_d1113h_60x_04_2024-08-02_10.32.21_2024-08-05T16-41-04.861.jpeg]

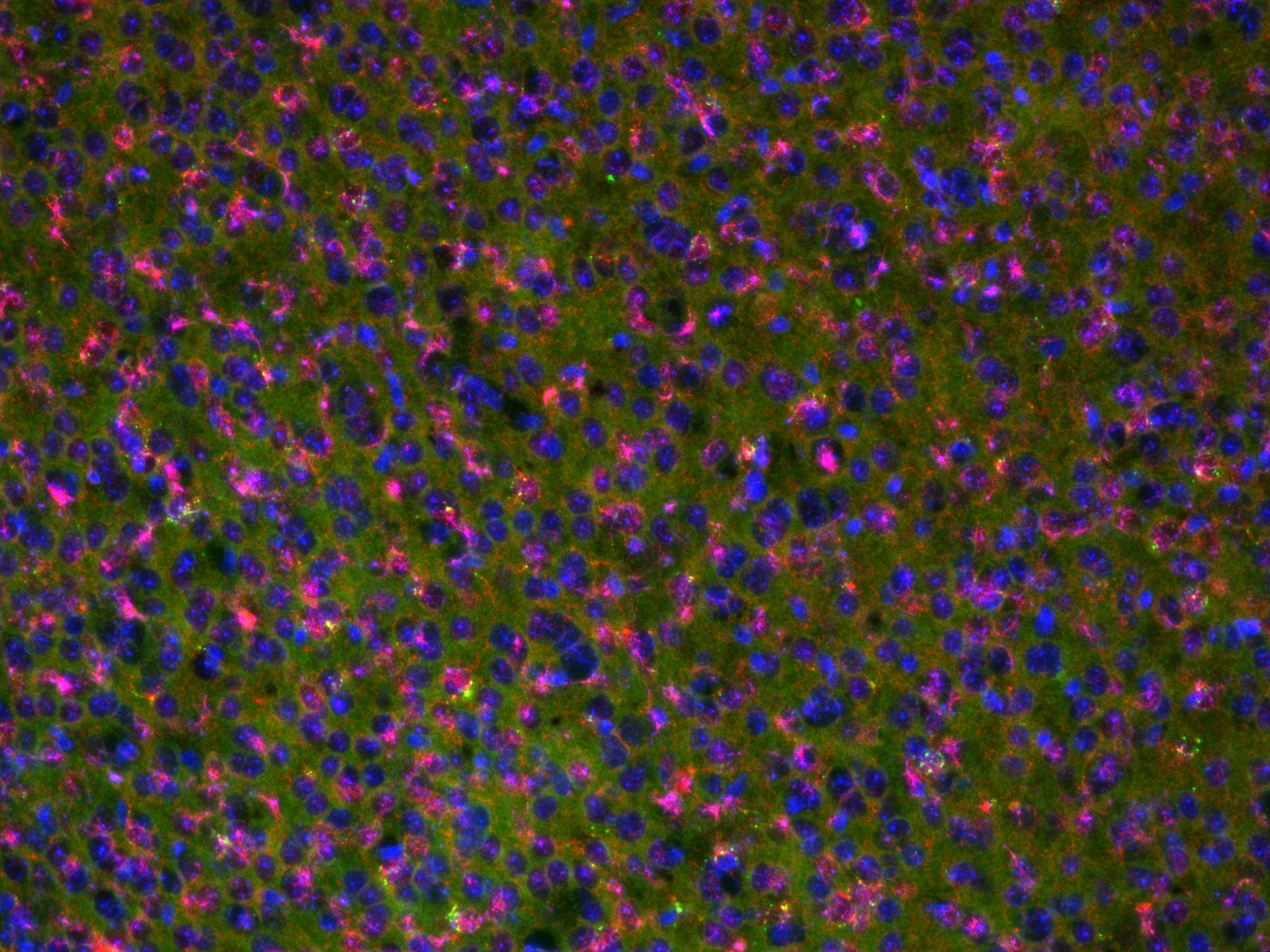

Supplement: Supplementary file 2. [file elife-102900-supp2.zip › Supplementary File 2/Raw RNAScope/1216 Overlay.jpeg]

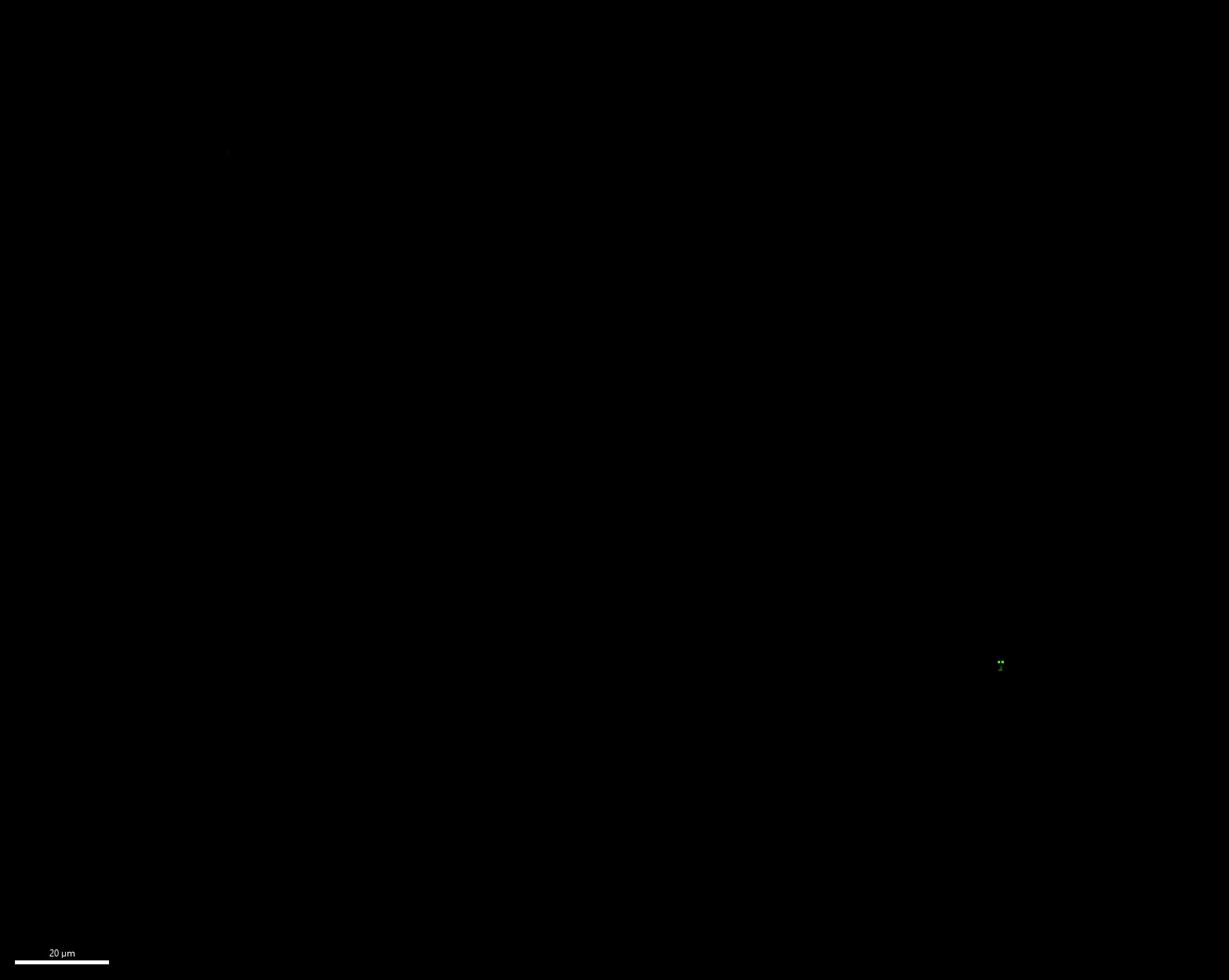

Supplement: Supplementary file 2. [file elife-102900-supp2.zip › Supplementary File 2/Raw RNAScope/5_2025-03-21_11.57.59_cre.jpeg]

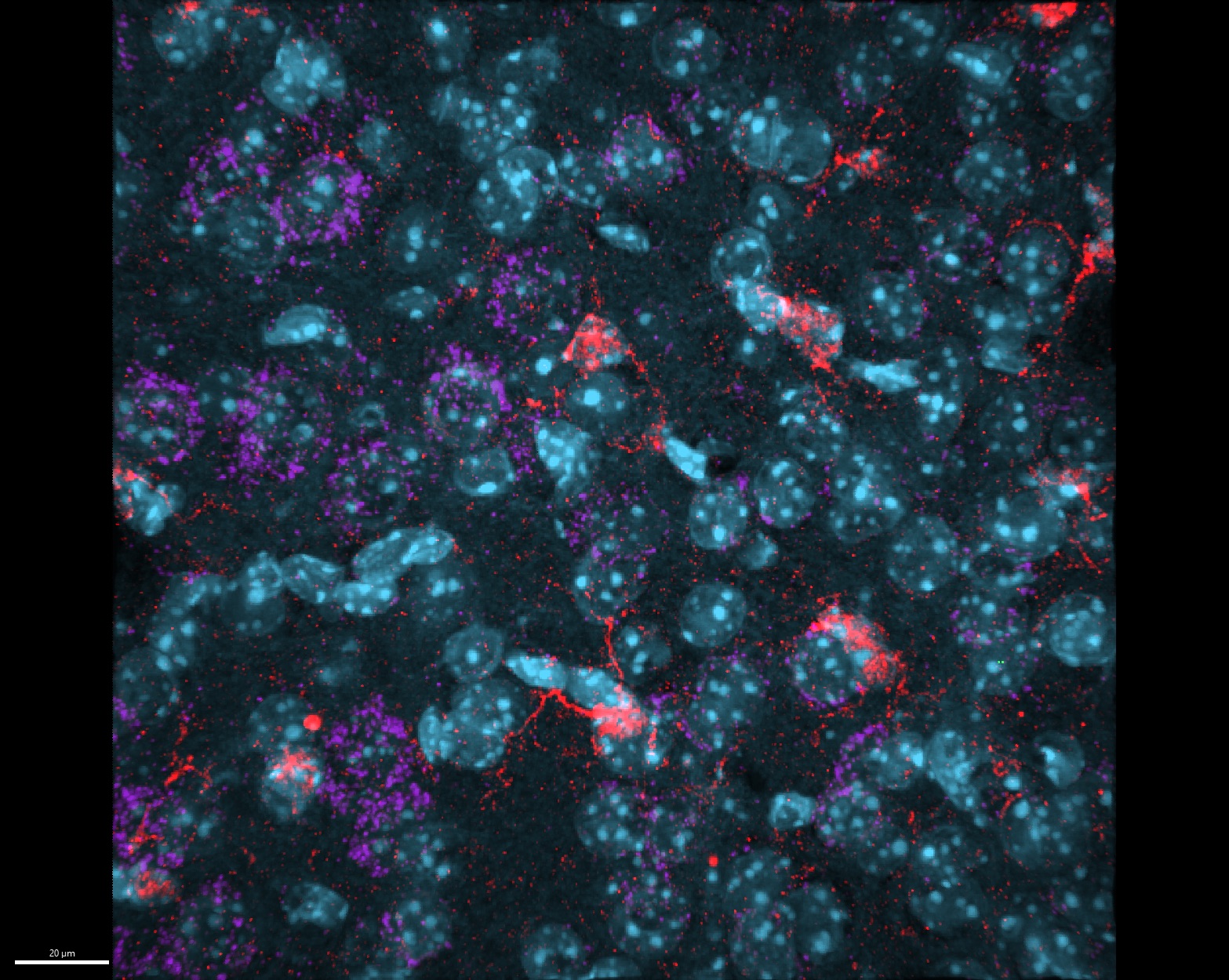

Supplement: Supplementary file 2. [file elife-102900-supp2.zip › Supplementary File 2/Raw RNAScope/5_2025-03-21_11.57.59_overlay.jpeg]

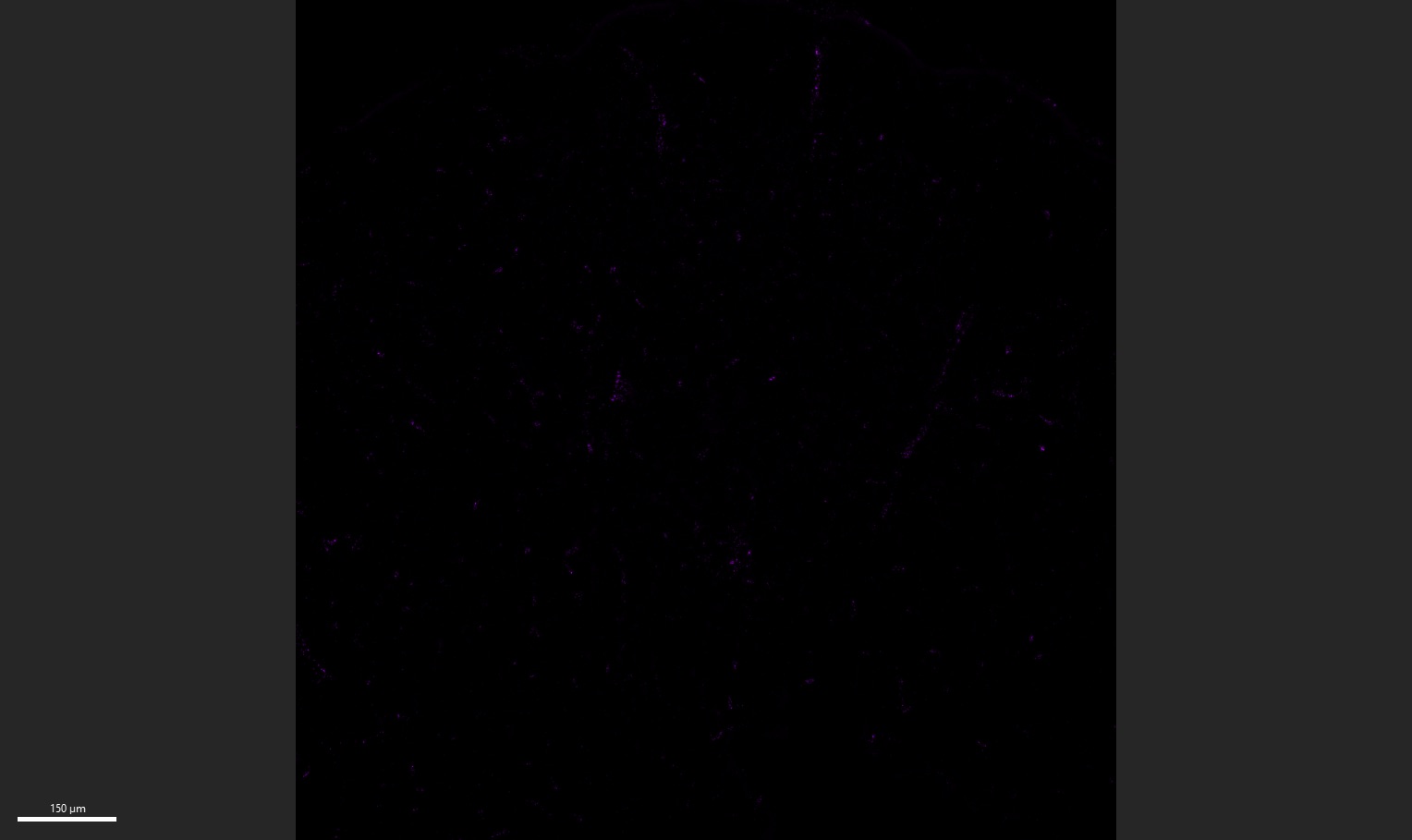

Supplement: Supplementary file 2. [file elife-102900-supp2.zip › Supplementary File 2/Raw RNAScope/1184_ict_wtb6_10x_02_2024-07-30_15.33.25_2024-08-02T11-59-29.458.jpeg]

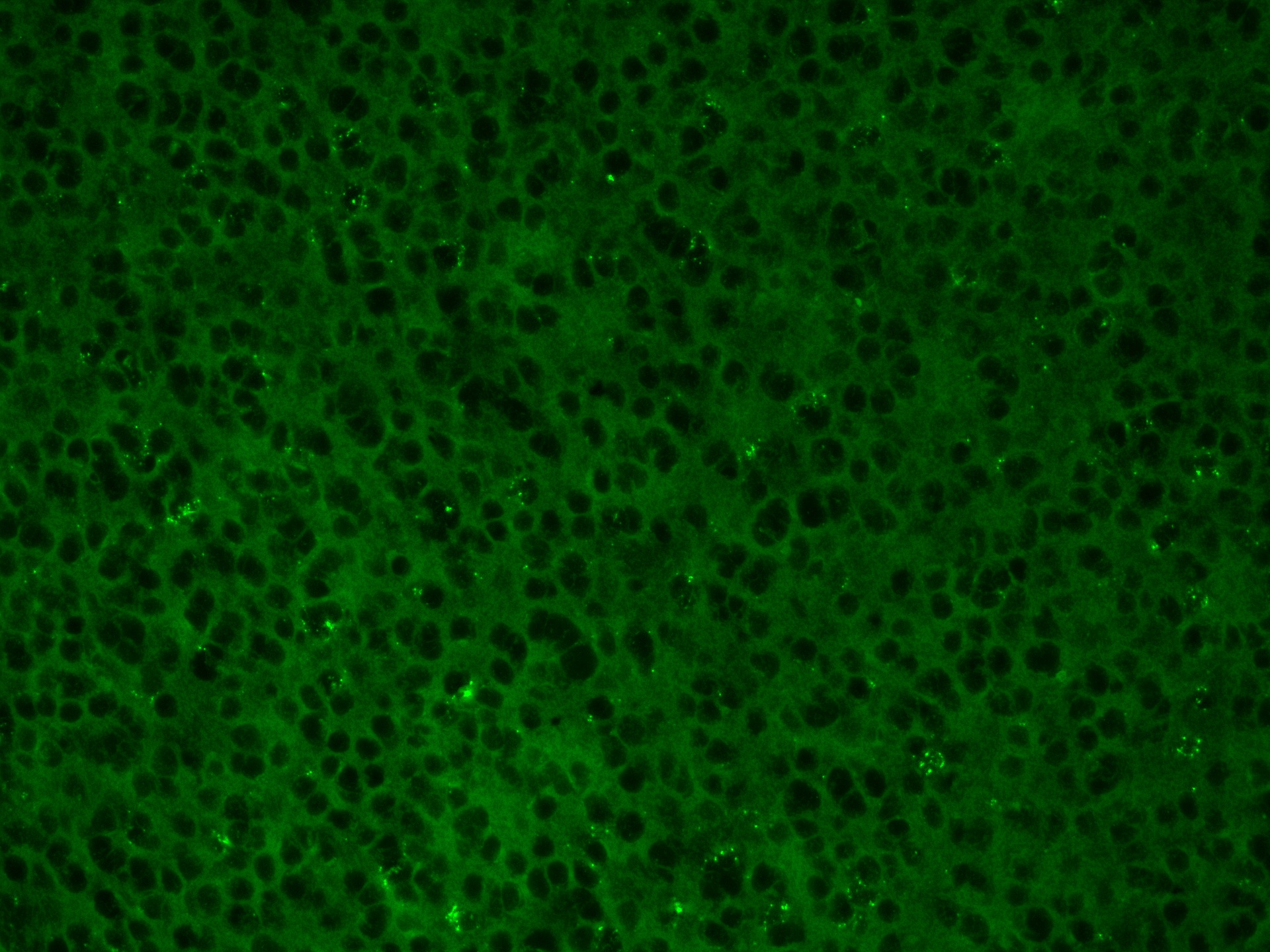

Supplement: Supplementary file 2. [file elife-102900-supp2.zip › Supplementary File 2/Raw RNAScope/1216 Cre.jpeg]

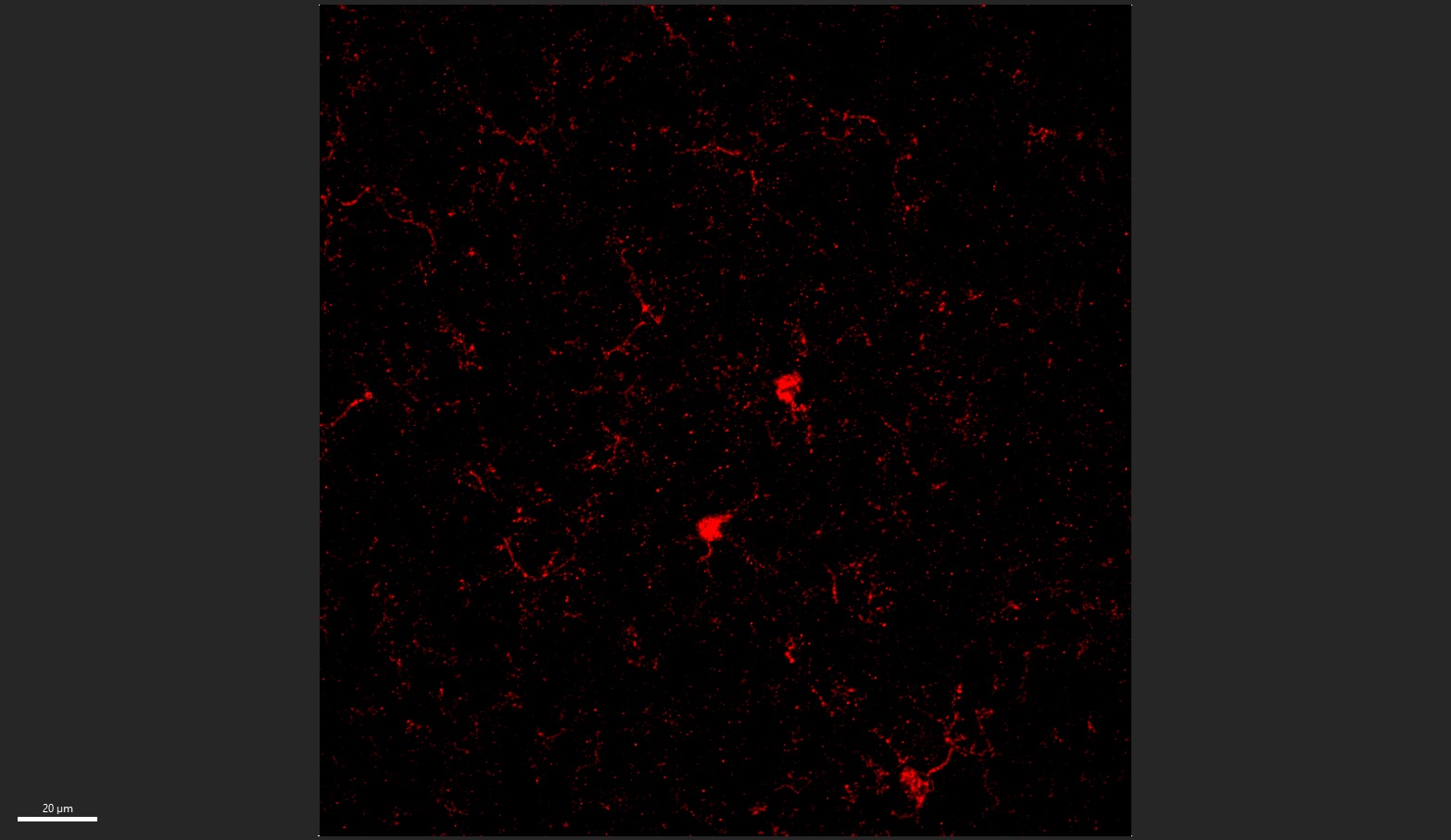

Supplement: Supplementary file 2. [file elife-102900-supp2.zip › Supplementary File 2/Raw RNAScope/1214_tamsham_60x_02-1_2024-07-30_15.00.14_2024-08-06T15-11-50.664.jpeg]

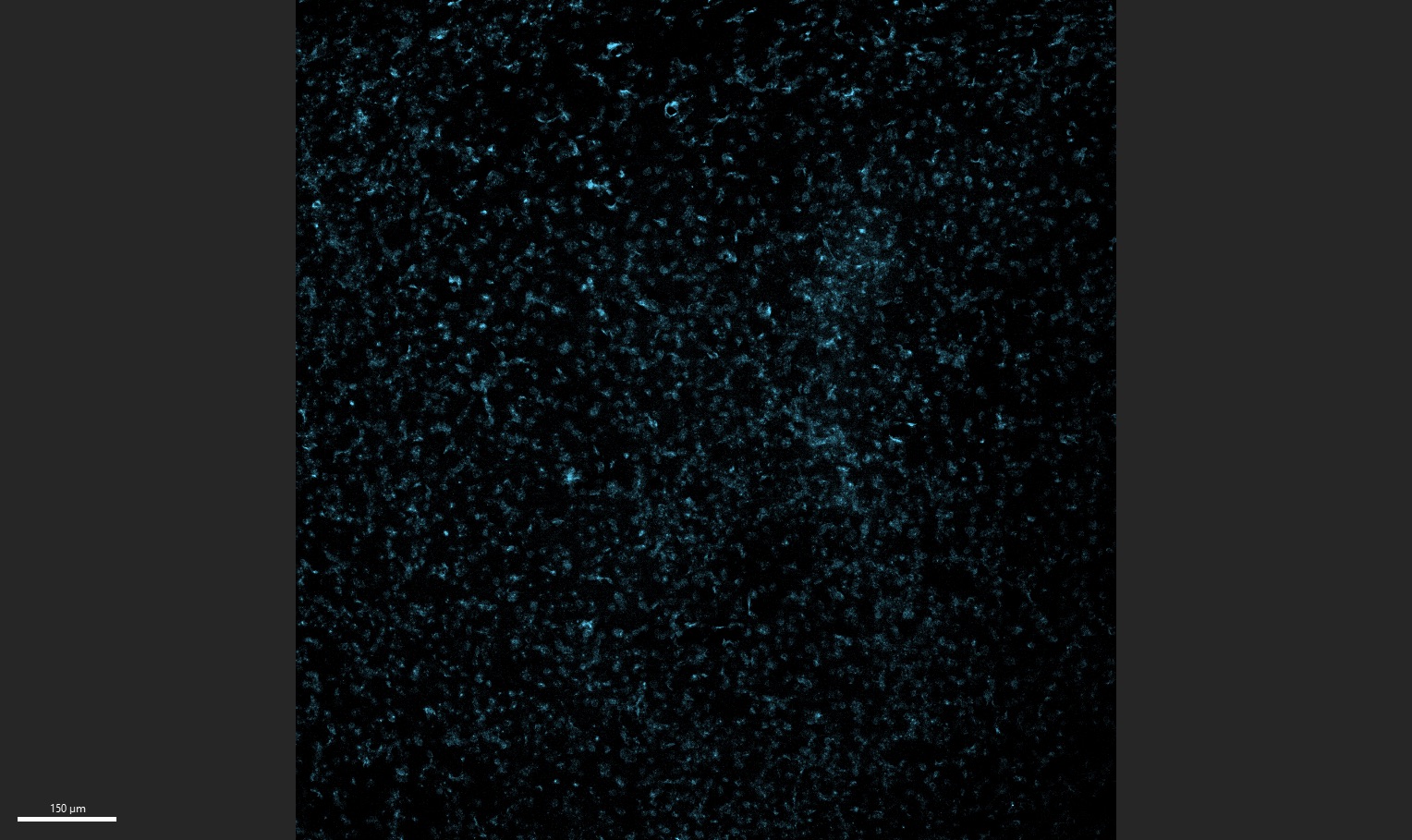

Supplement: Supplementary file 2. [file elife-102900-supp2.zip › Supplementary File 2/Raw RNAScope/1172_full_d1113h_10x_02_2024-07-30_14.00.56_2024-08-02T11-50-55.594.jpeg]

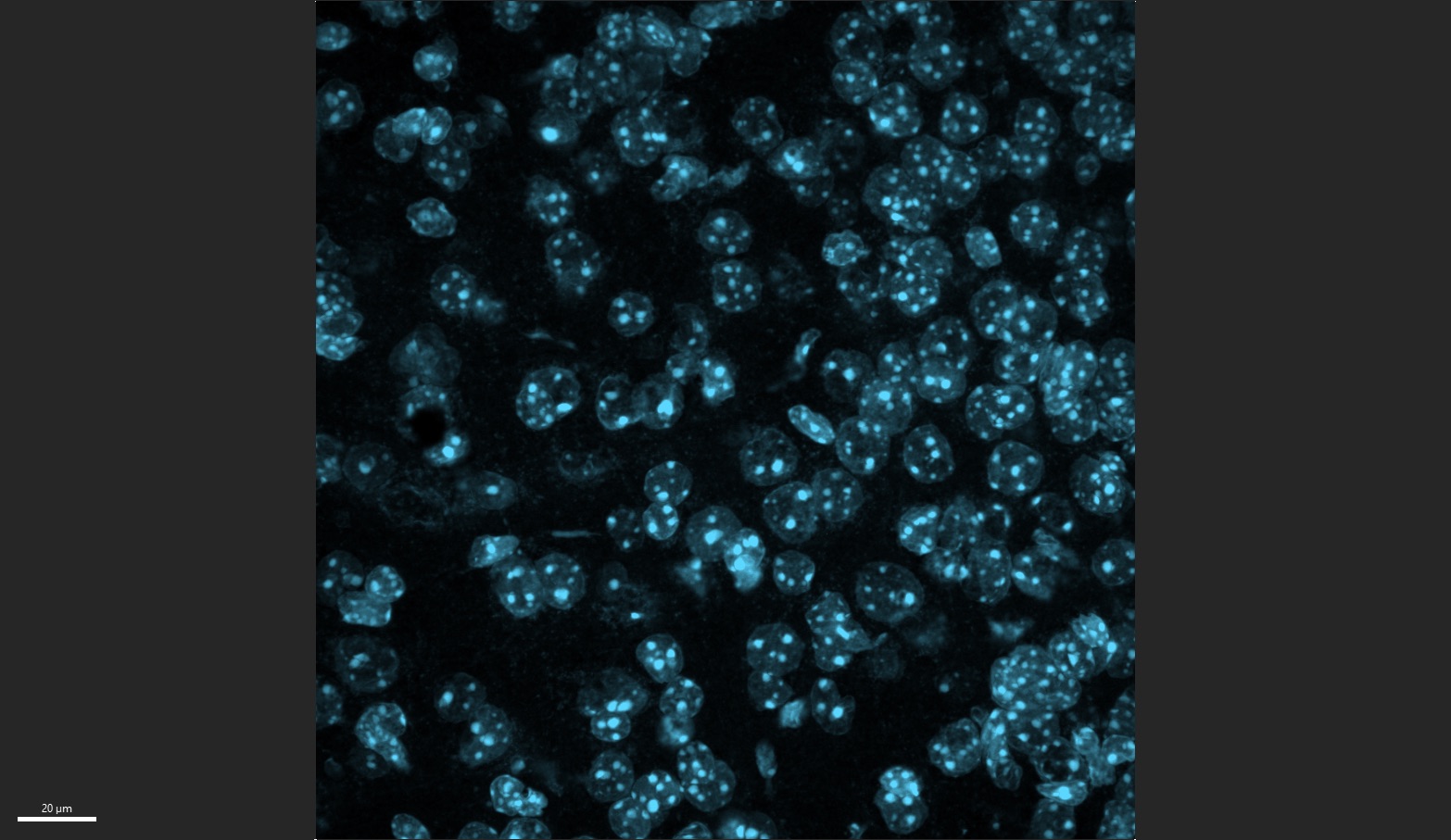

Supplement: Supplementary file 2. [file elife-102900-supp2.zip › Supplementary File 2/Raw RNAScope/1188_ict_d1113h_60x_04_2024-08-02_10.32.21_2024-08-05T16-40-54.278.jpeg]

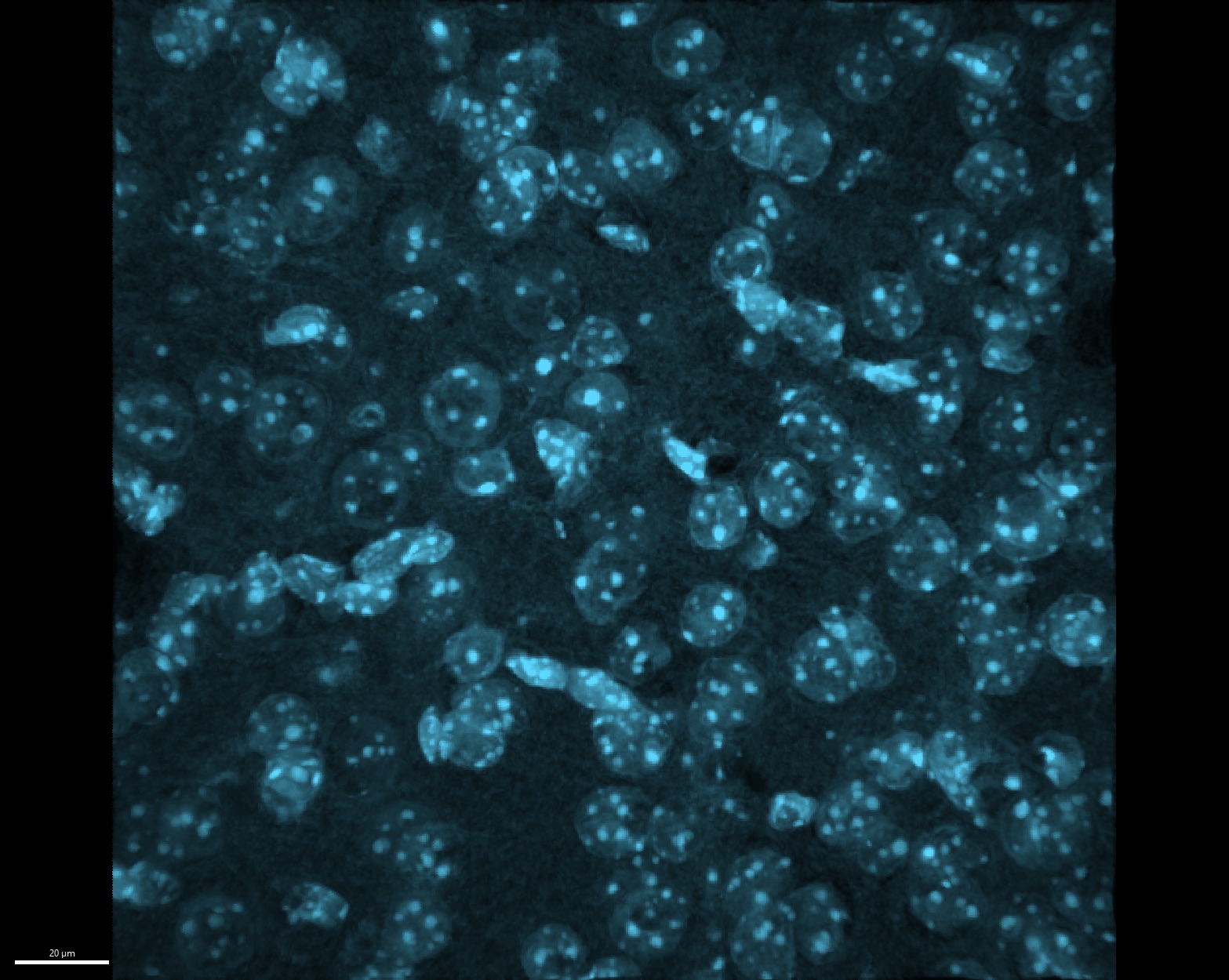

Supplement: Supplementary file 2. [file elife-102900-supp2.zip › Supplementary File 2/Raw RNAScope/5_2025-03-21_11.57.59_dapi.jpeg]

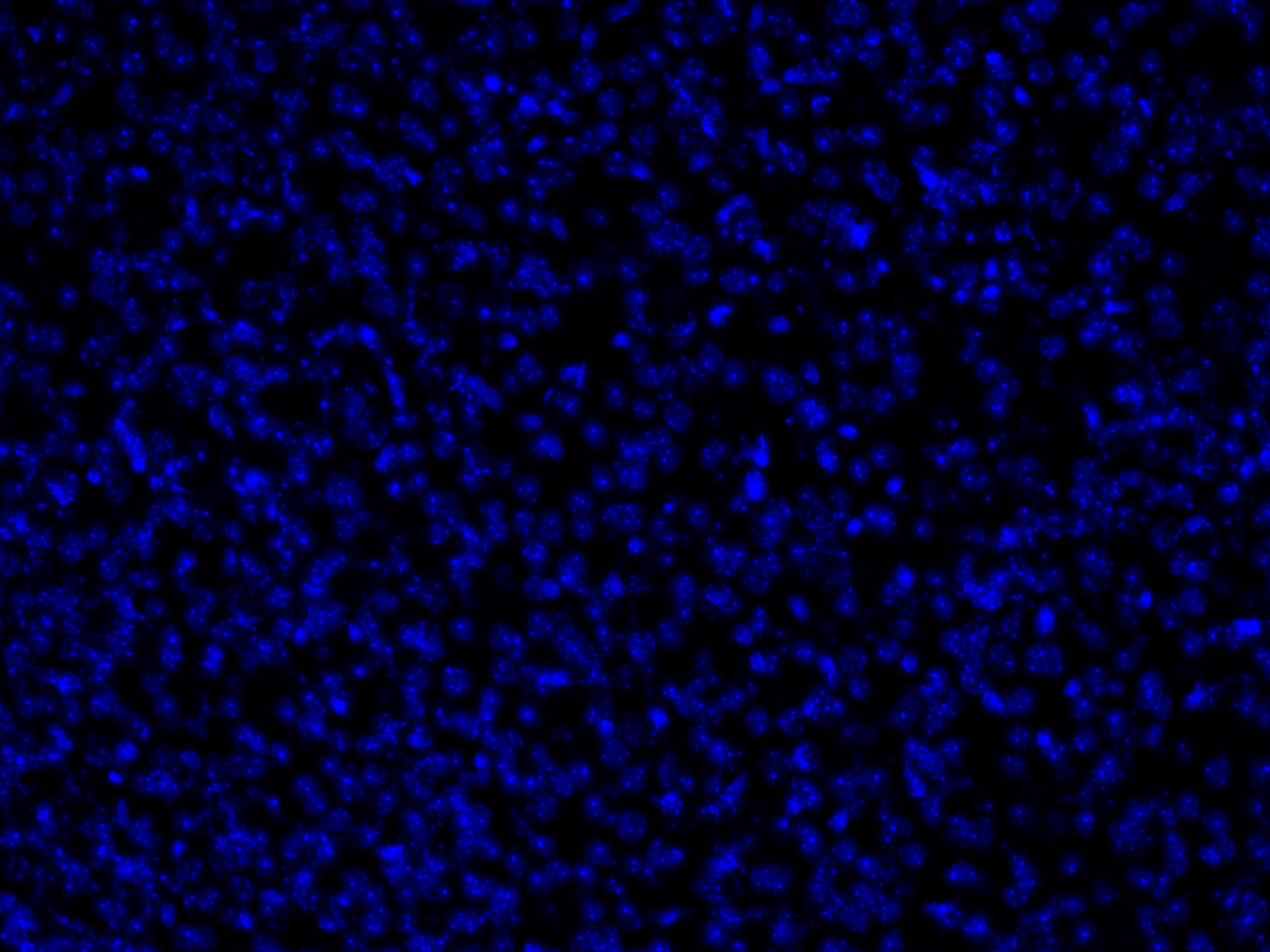

Supplement: Supplementary file 2. [file elife-102900-supp2.zip › Supplementary File 2/Raw RNAScope/1216 DAPI.jpeg]

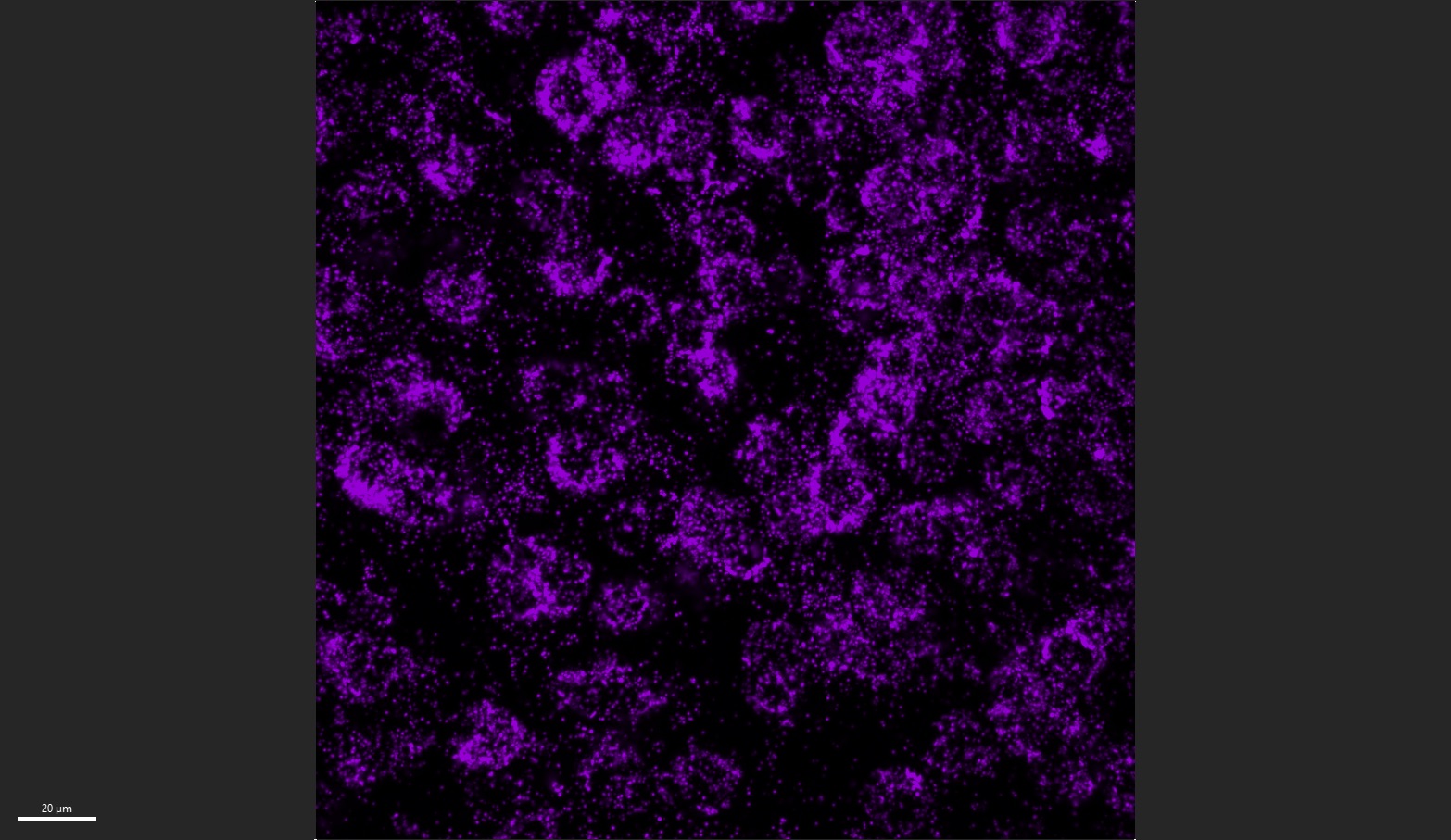

Supplement: Supplementary file 2. [file elife-102900-supp2.zip › Supplementary File 2/Raw RNAScope/1188_ict_d1113h_60x_04_2024-08-02_10.32.21_2024-08-05T16-41-10.237.jpeg]

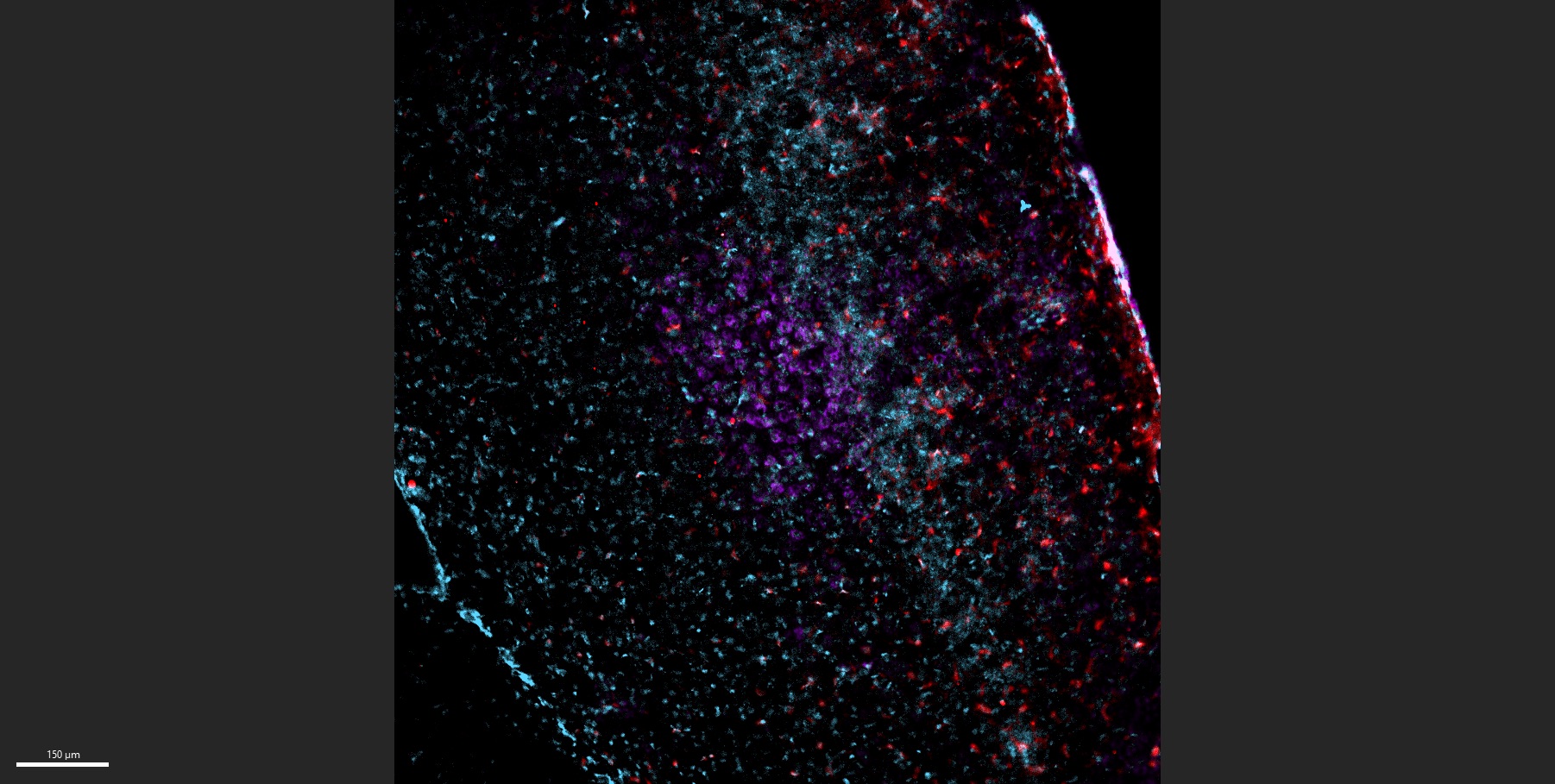

Supplement: Supplementary file 2. [file elife-102900-supp2.zip › Supplementary File 2/Raw RNAScope/1188_ict_d1113h_10x_04_2024-07-30_15.27.18_2024-08-05T16-43-30.013.jpeg]

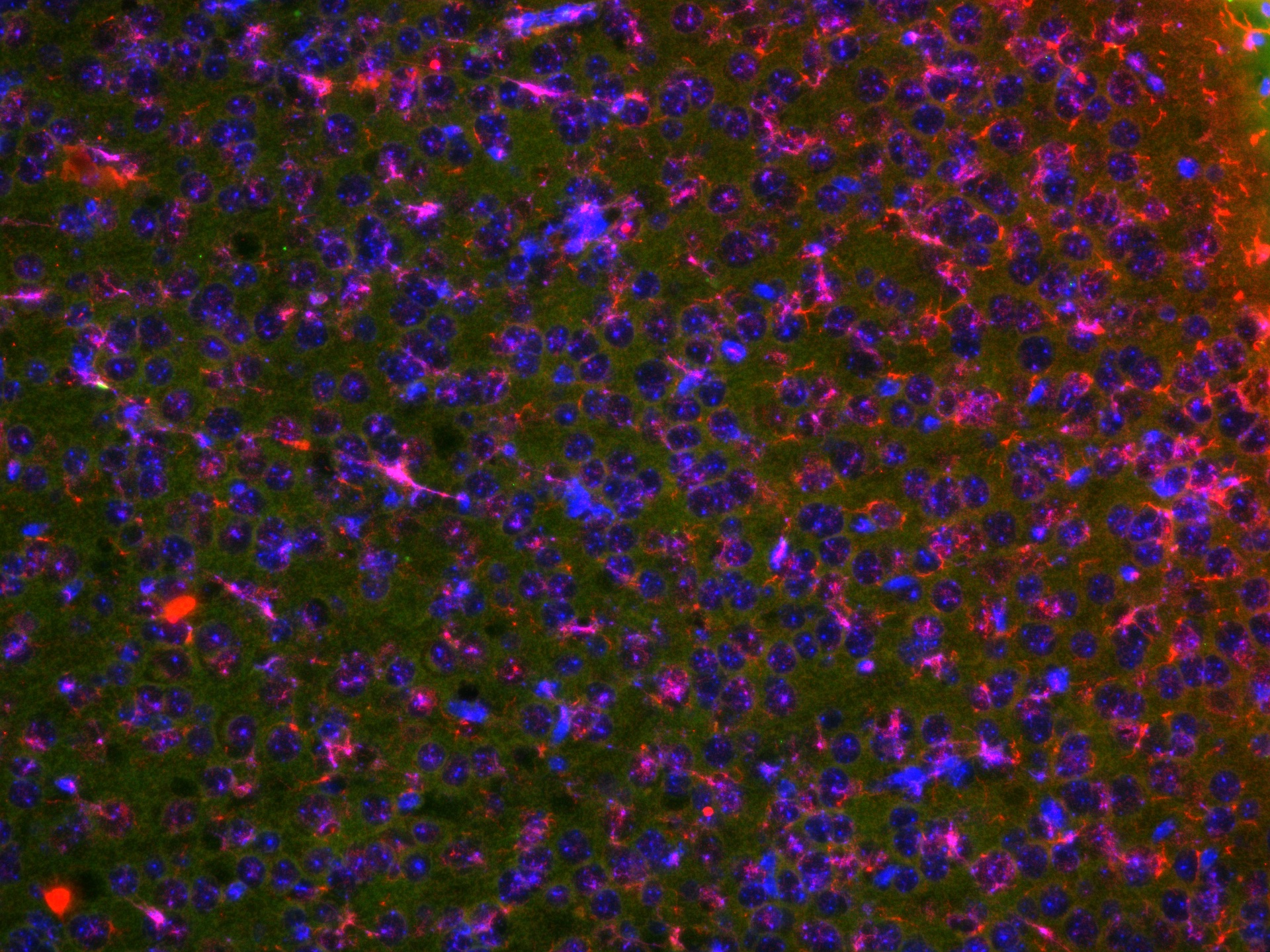

Supplement: Supplementary file 2. [file elife-102900-supp2.zip › Supplementary File 2/Raw RNAScope/1182 overlay.jpeg]

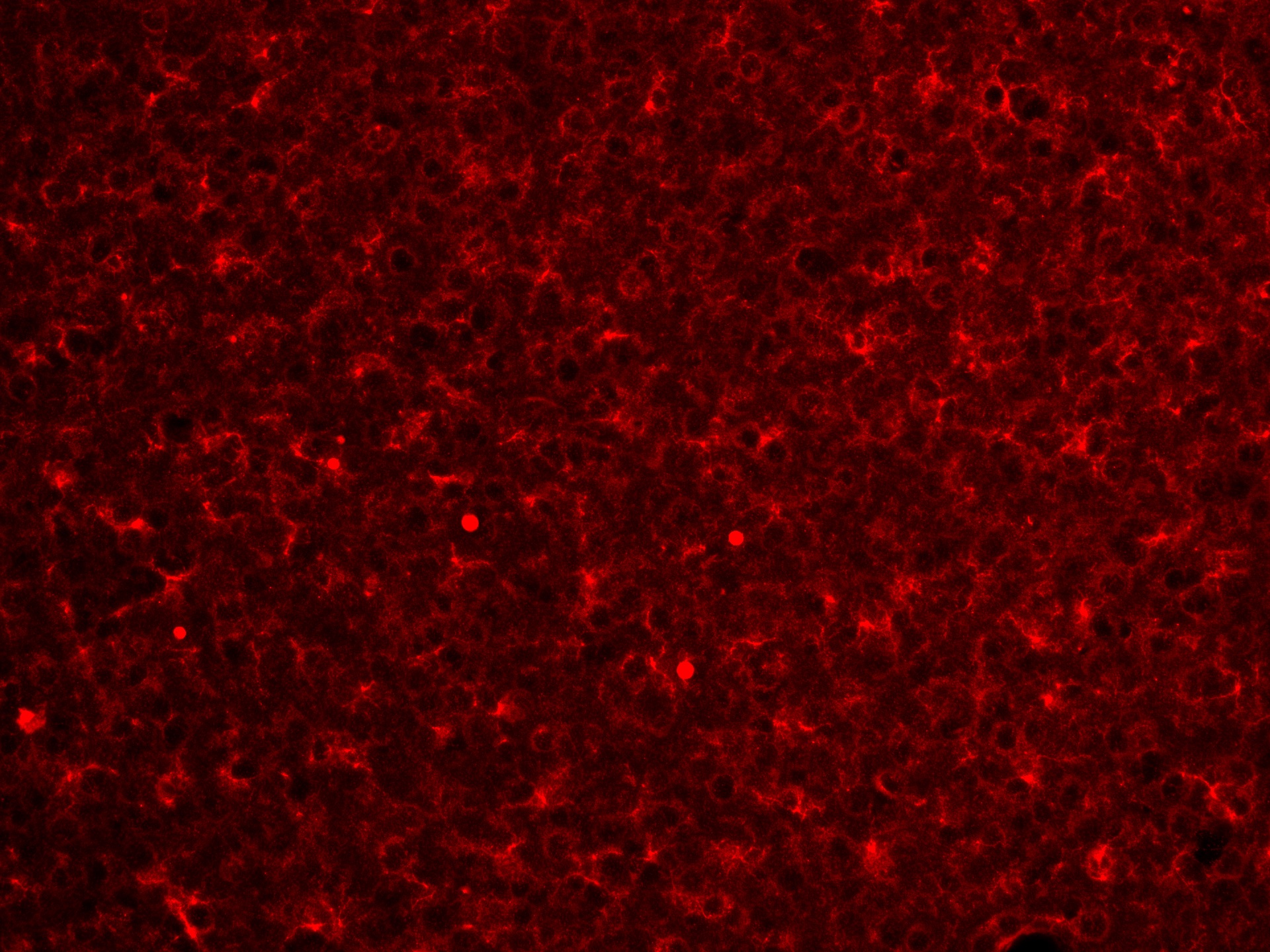

Supplement: Supplementary file 2. [file elife-102900-supp2.zip › Supplementary File 2/Raw RNAScope/1188 iba.jpeg]

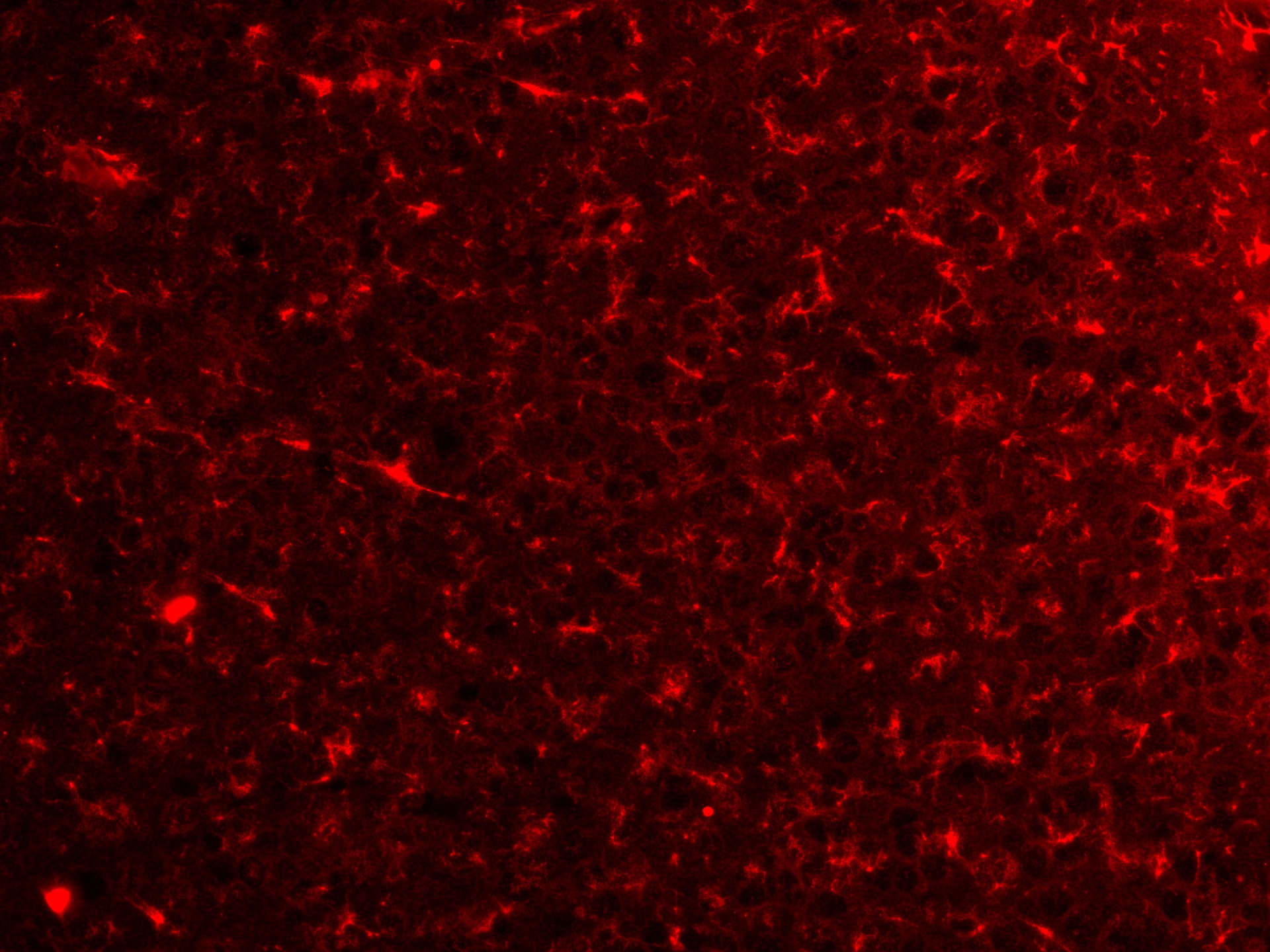

Supplement: Supplementary file 2. [file elife-102900-supp2.zip › Supplementary File 2/Raw RNAScope/1182 iba.jpeg]

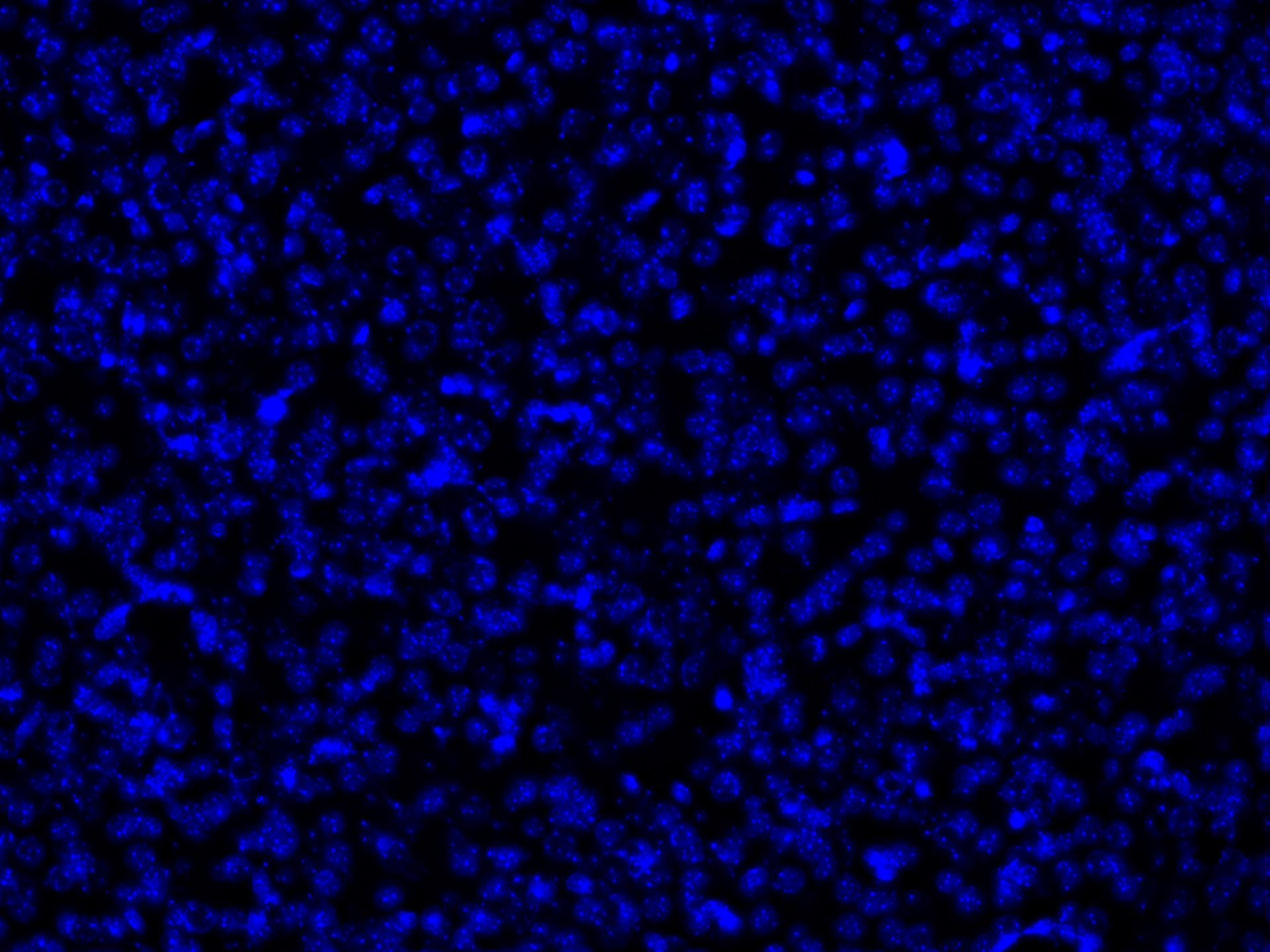

Supplement: Supplementary file 2. [file elife-102900-supp2.zip › Supplementary File 2/Raw RNAScope/1188 dapi.jpeg]

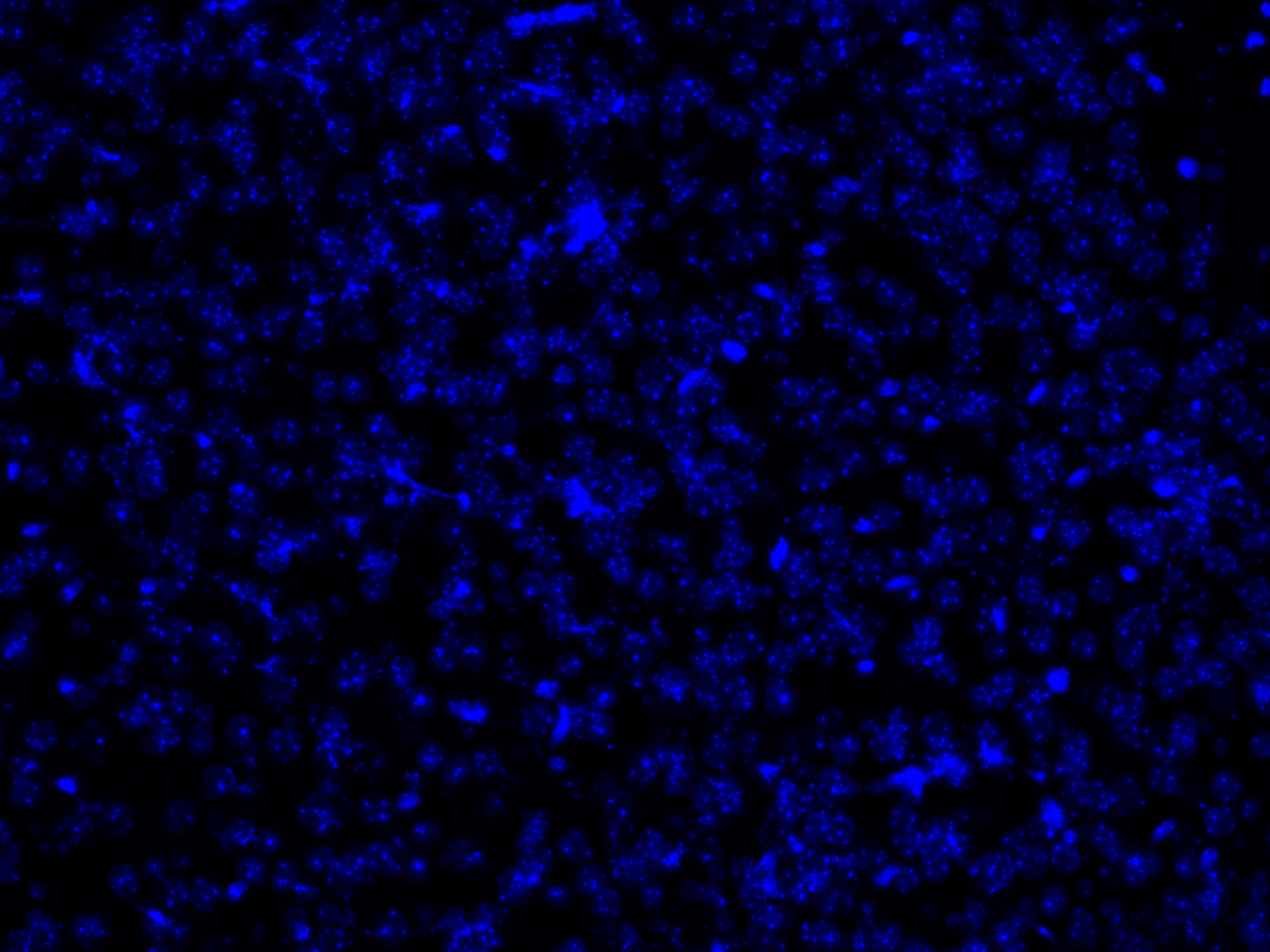

Supplement: Supplementary file 2. [file elife-102900-supp2.zip › Supplementary File 2/Raw RNAScope/1182 dapi.jpeg]

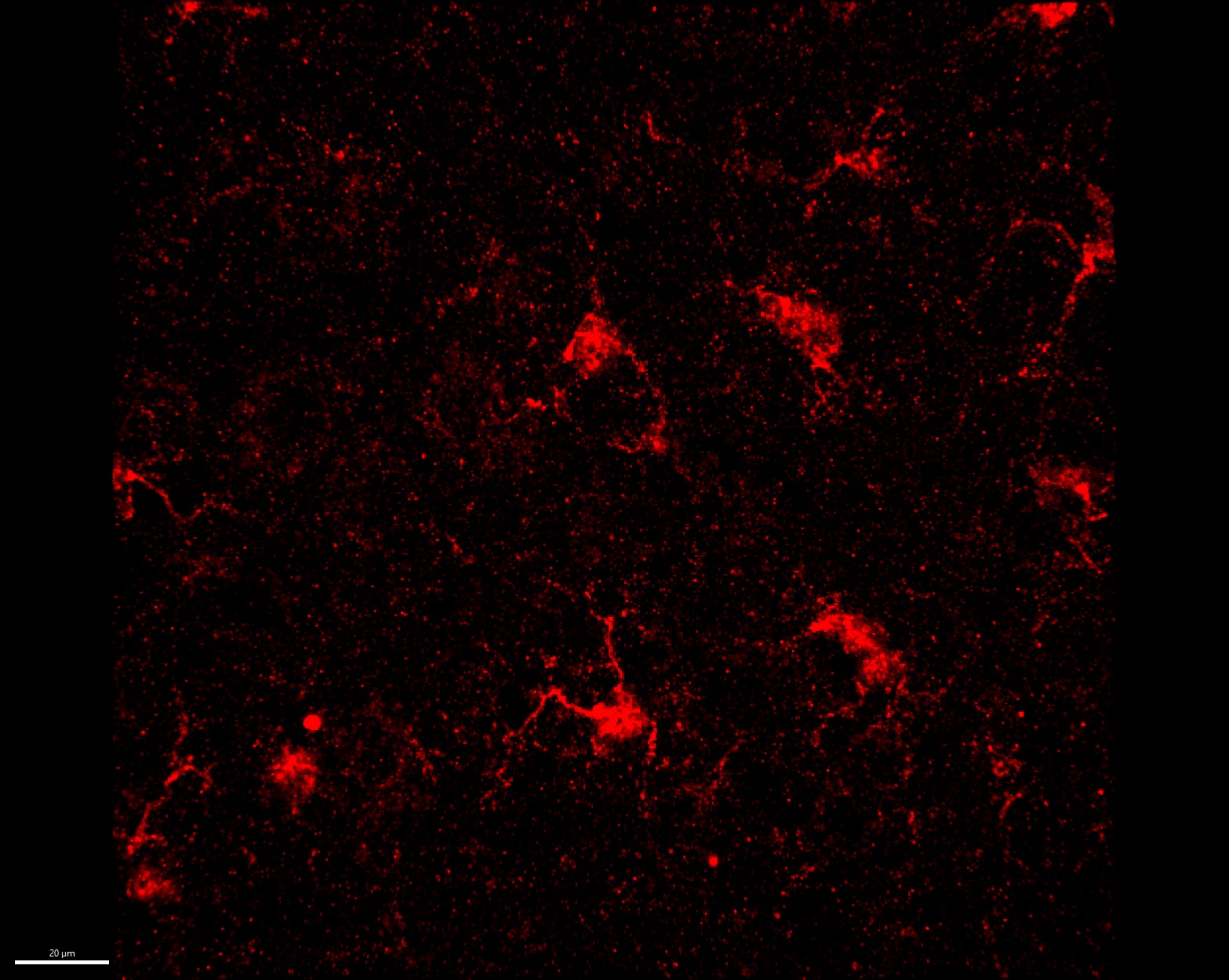

Supplement: Supplementary file 2. [file elife-102900-supp2.zip › Supplementary File 2/Raw RNAScope/5_2025-03-21_11.57.59_iba1.jpeg]

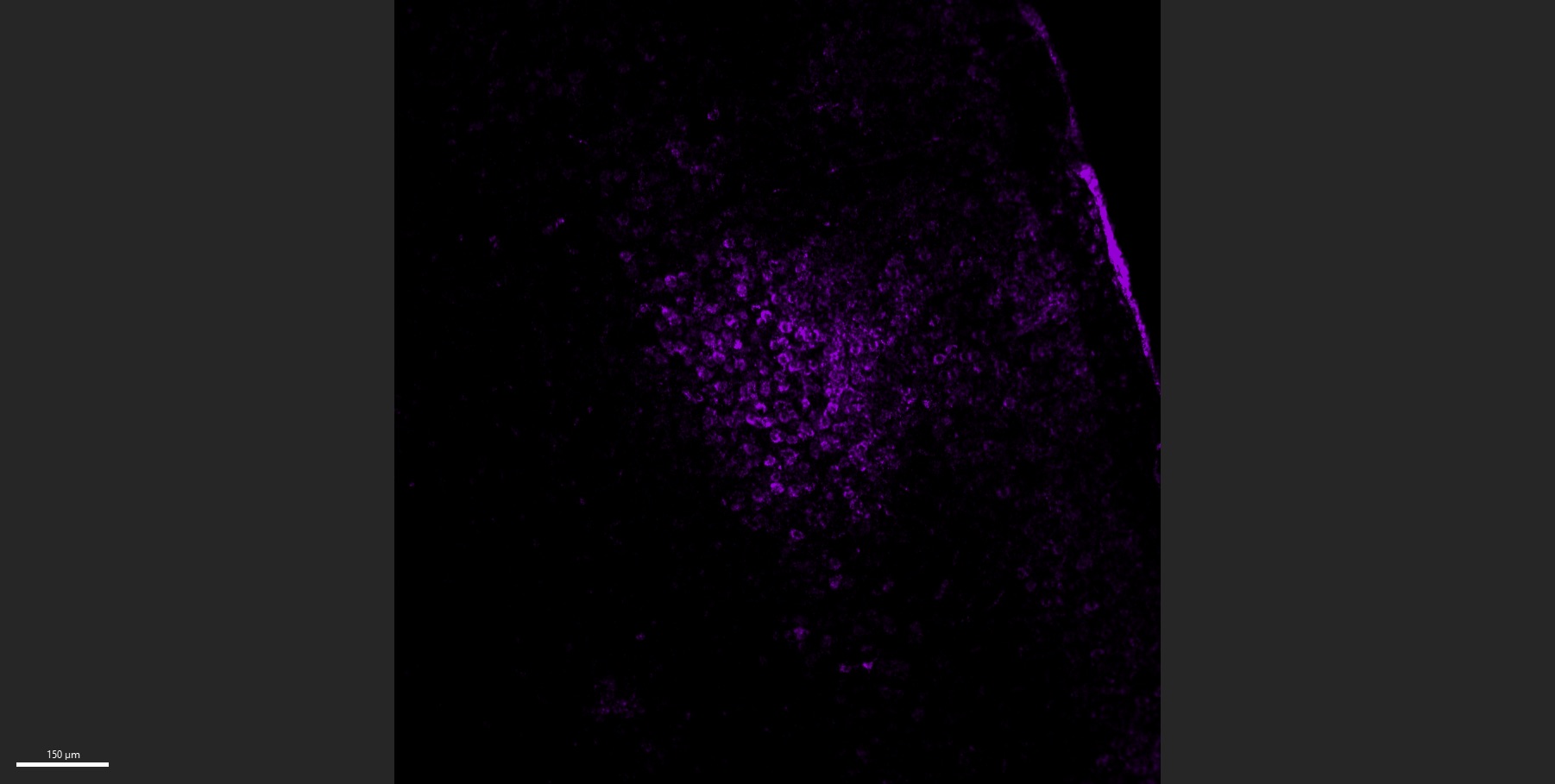

Supplement: Supplementary file 2. [file elife-102900-supp2.zip › Supplementary File 2/Raw RNAScope/1188_ict_d1113h_10x_04_2024-07-30_15.27.18_2024-08-05T16-43-46.645.jpeg]

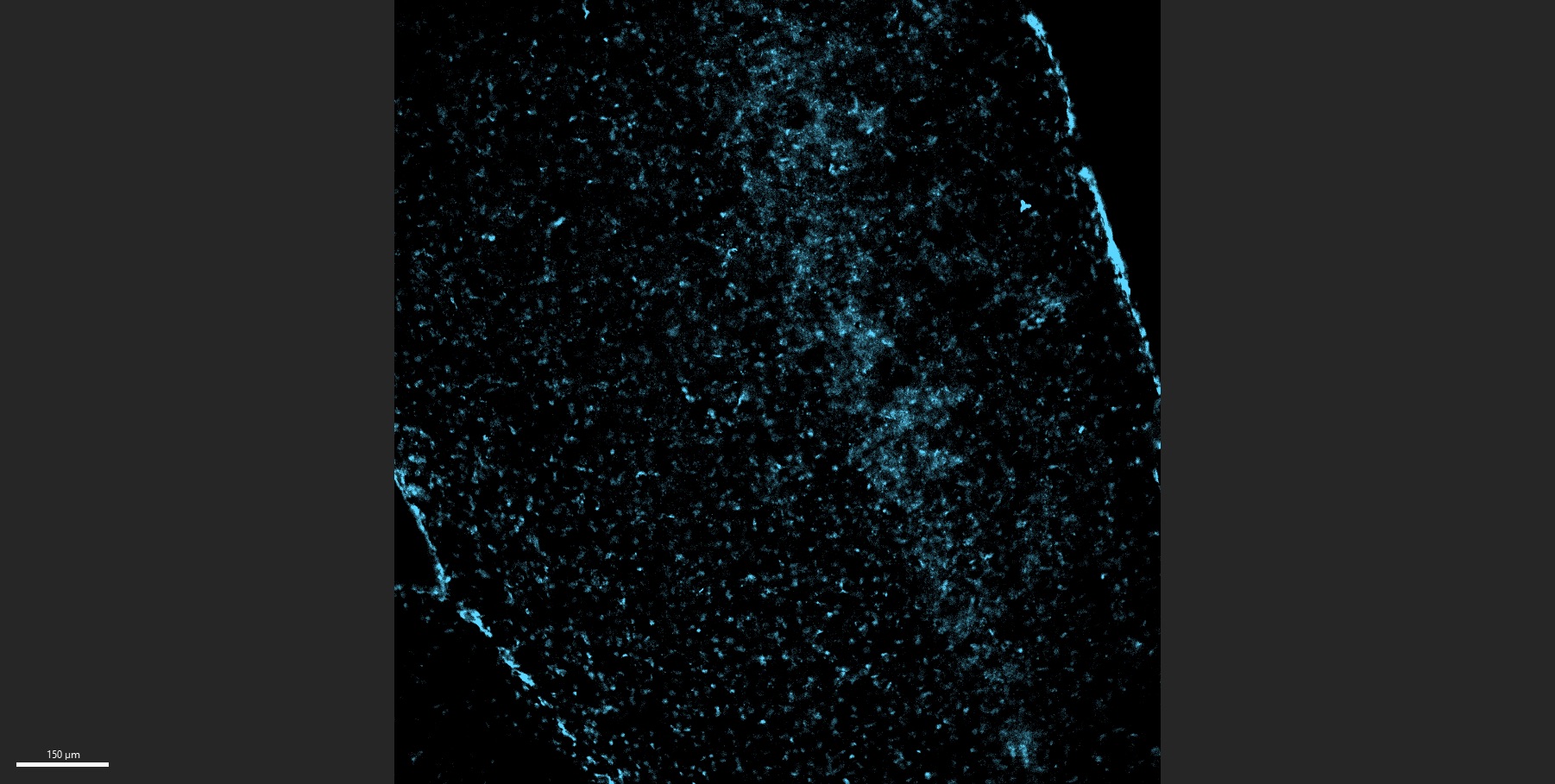

Supplement: Supplementary file 2. [file elife-102900-supp2.zip › Supplementary File 2/Raw RNAScope/1188_ict_d1113h_10x_04_2024-07-30_15.27.18_2024-08-05T16-43-37.068.jpeg]

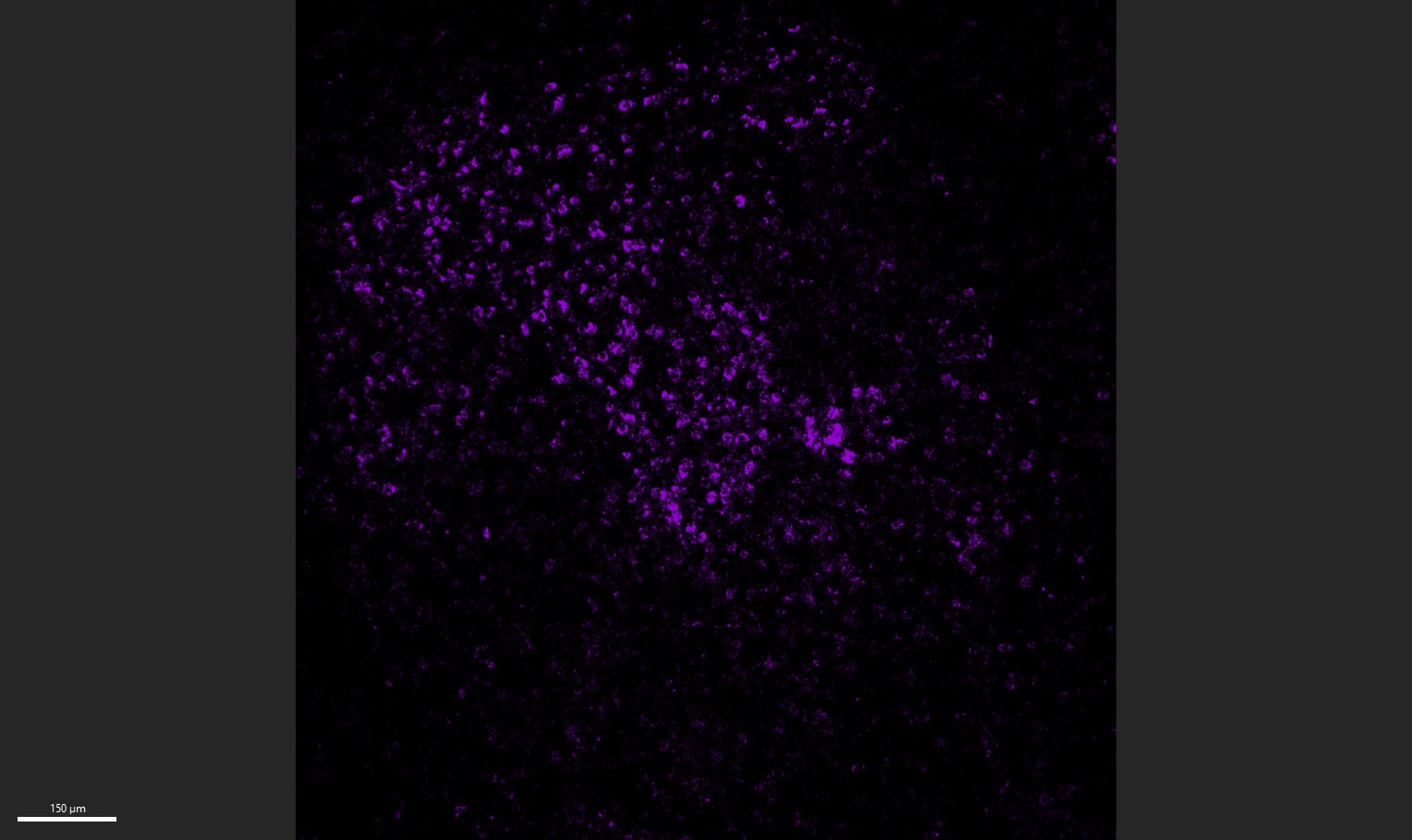

Supplement: Supplementary file 2. [file elife-102900-supp2.zip › Supplementary File 2/Raw RNAScope/1172_full_d1113h_10x_02_2024-07-30_14.00.56_2024-08-02T11-51-08.593.jpeg]

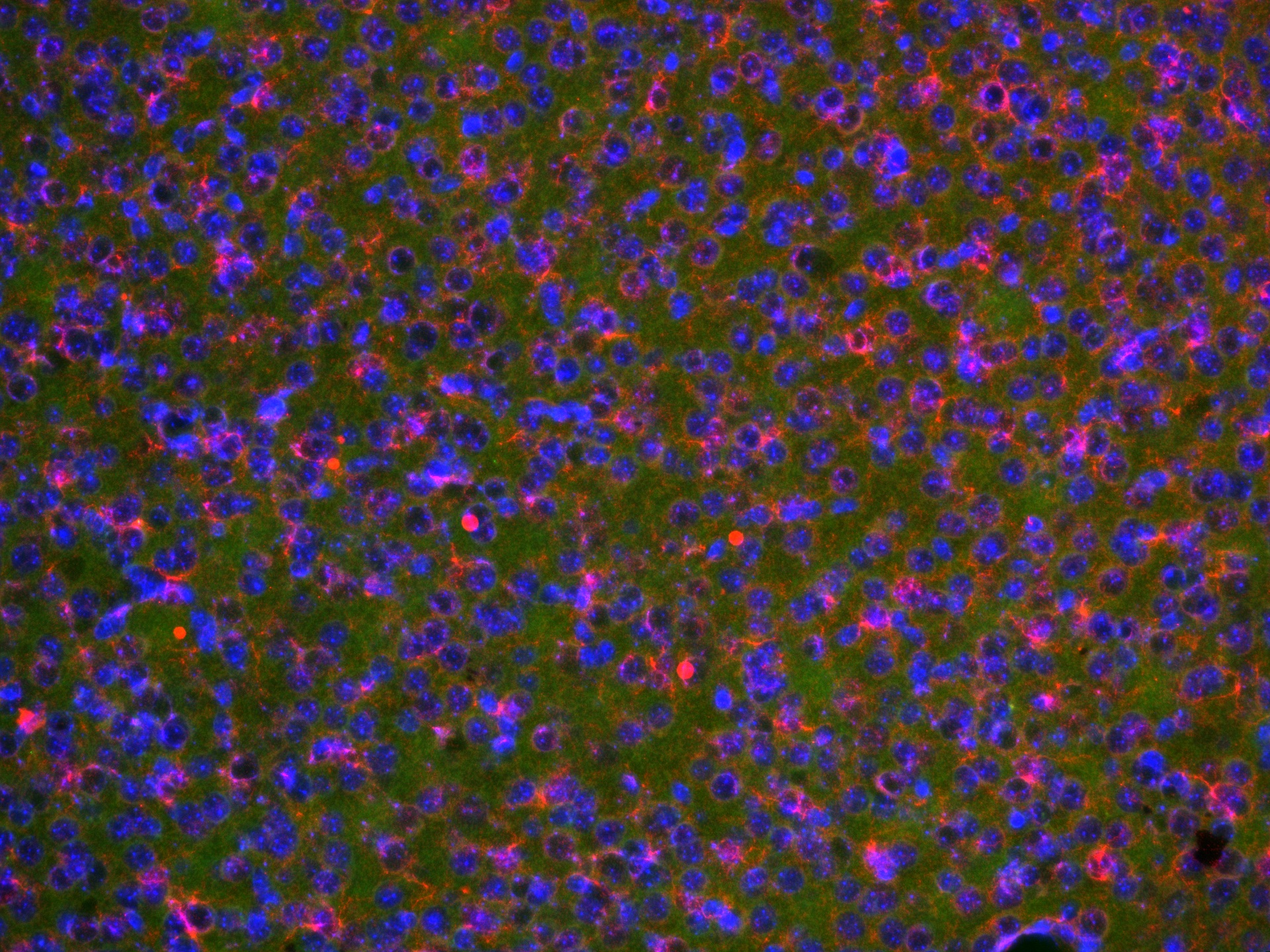

Supplement: Supplementary file 2. [file elife-102900-supp2.zip › Supplementary File 2/Raw RNAScope/1188 overlay.jpeg]

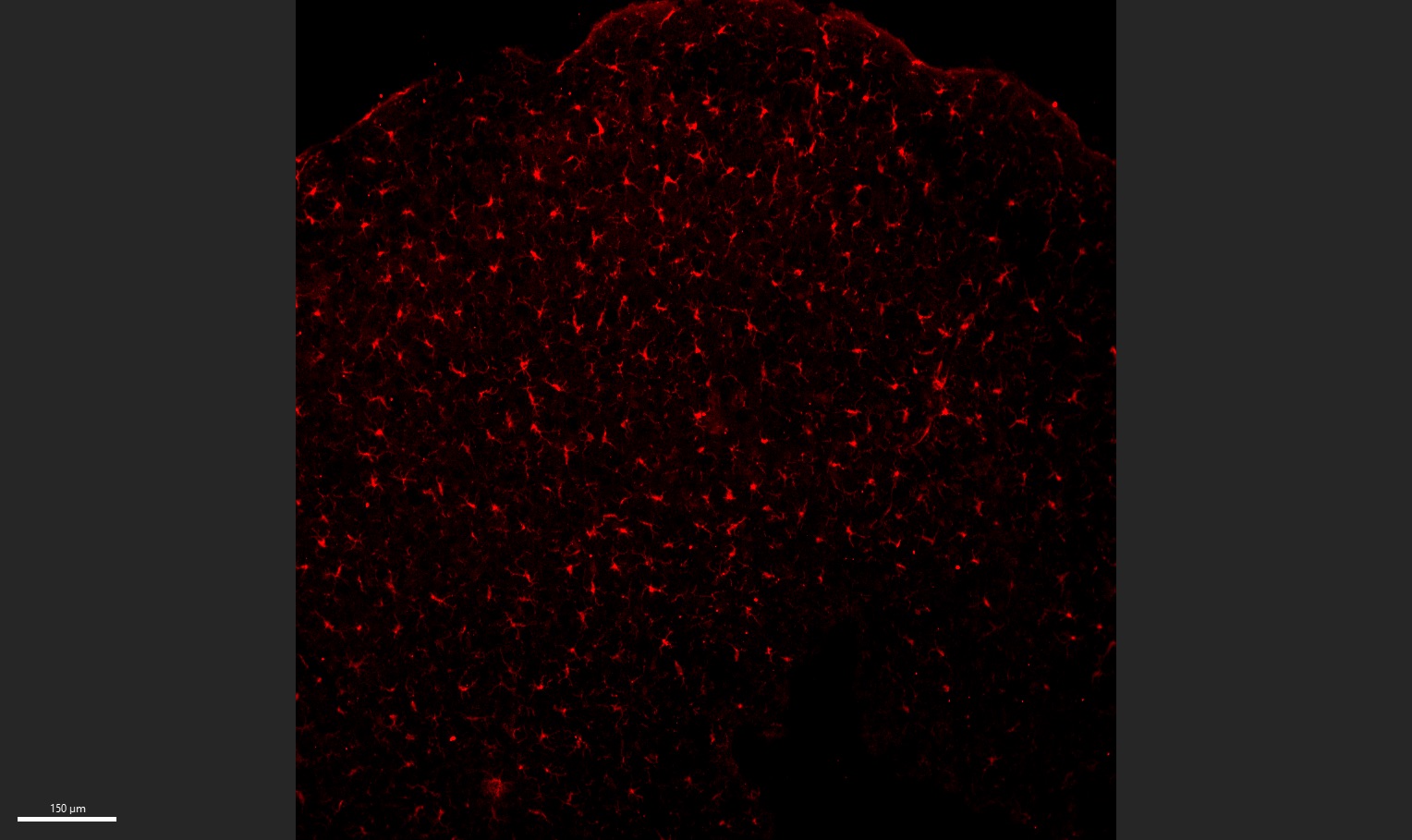

Supplement: Supplementary file 2. [file elife-102900-supp2.zip › Supplementary File 2/Raw RNAScope/1184_ict_wtb6_10x_02_2024-07-30_15.33.25_2024-08-02T11-59-23.169.jpeg]

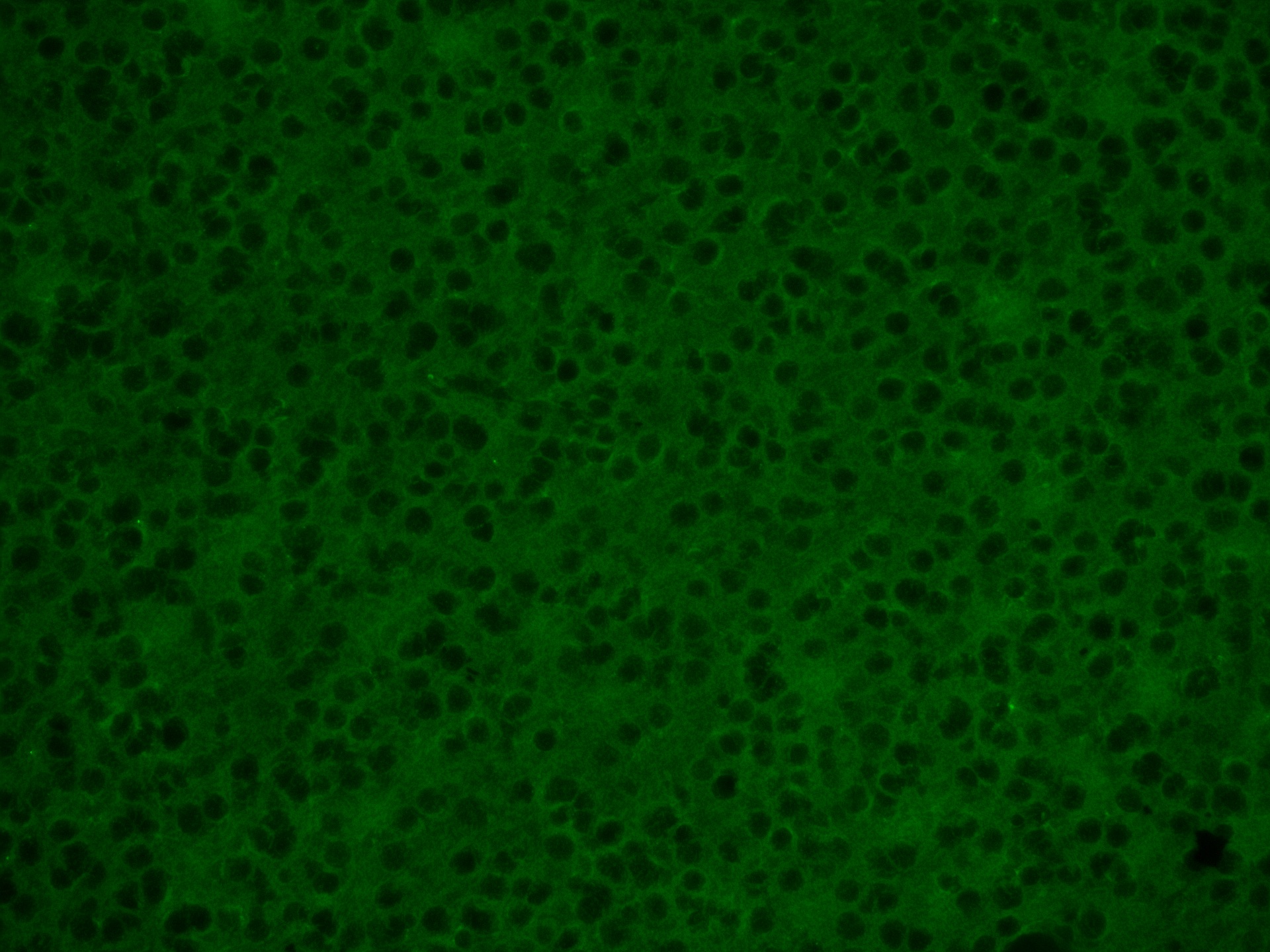

Supplement: Supplementary file 2. [file elife-102900-supp2.zip › Supplementary File 2/Raw RNAScope/1188 cre.jpeg]

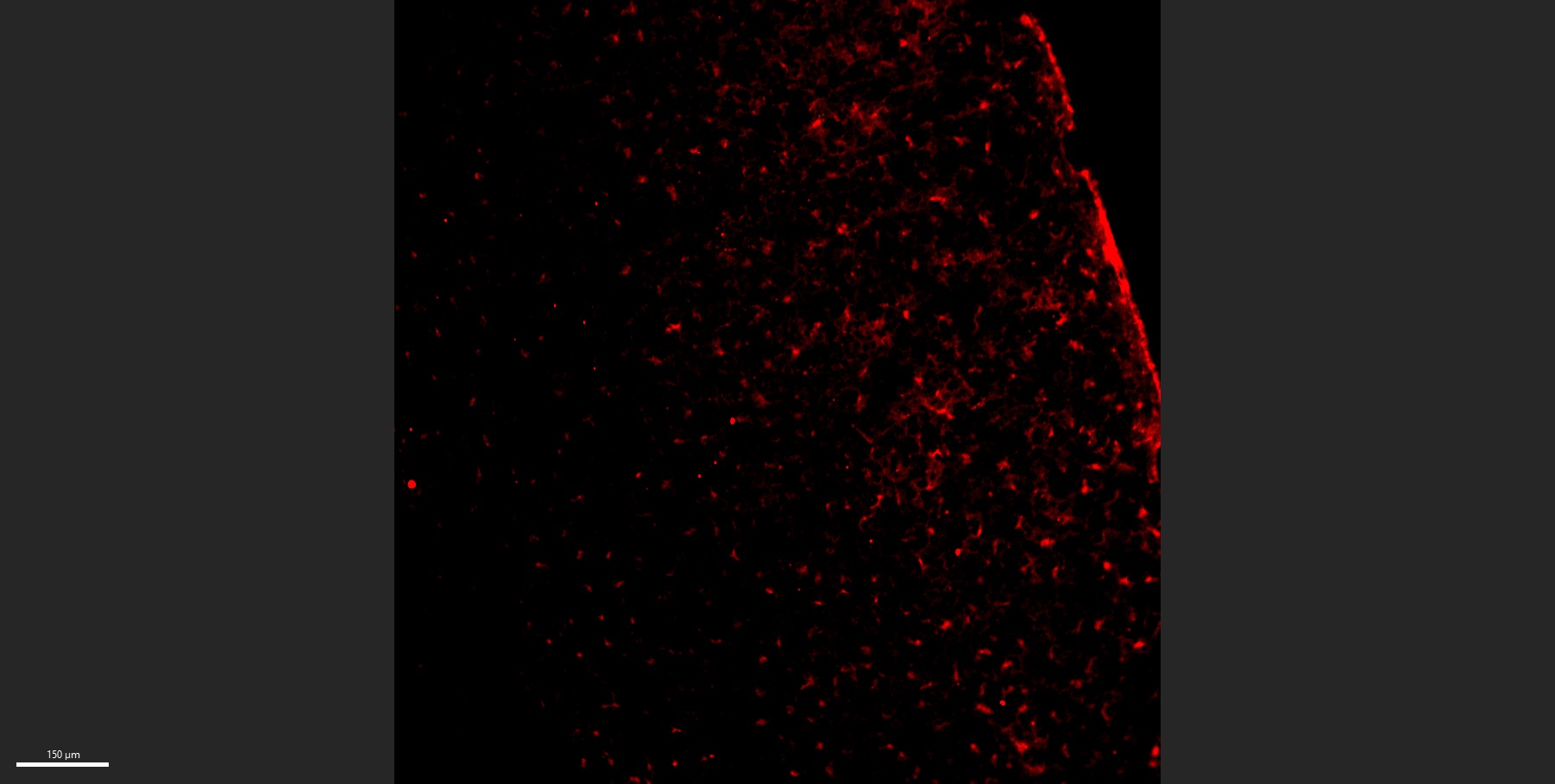

Supplement: Supplementary file 2. [file elife-102900-supp2.zip › Supplementary File 2/Raw RNAScope/1188_ict_d1113h_10x_04_2024-07-30_15.27.18_2024-08-05T16-43-41.989.jpeg]

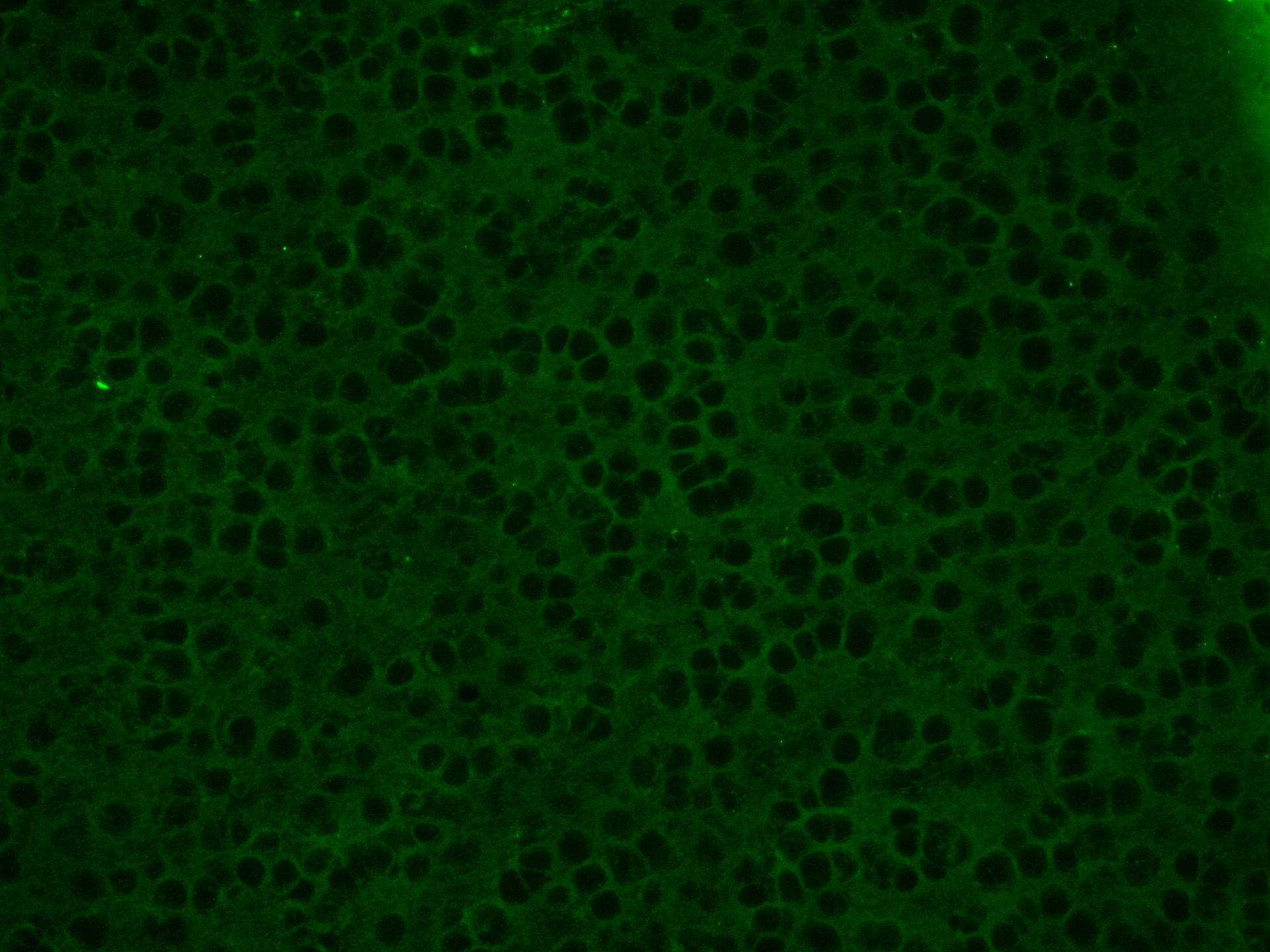

Supplement: Supplementary file 2. [file elife-102900-supp2.zip › Supplementary File 2/Raw RNAScope/1182 cre.jpeg]

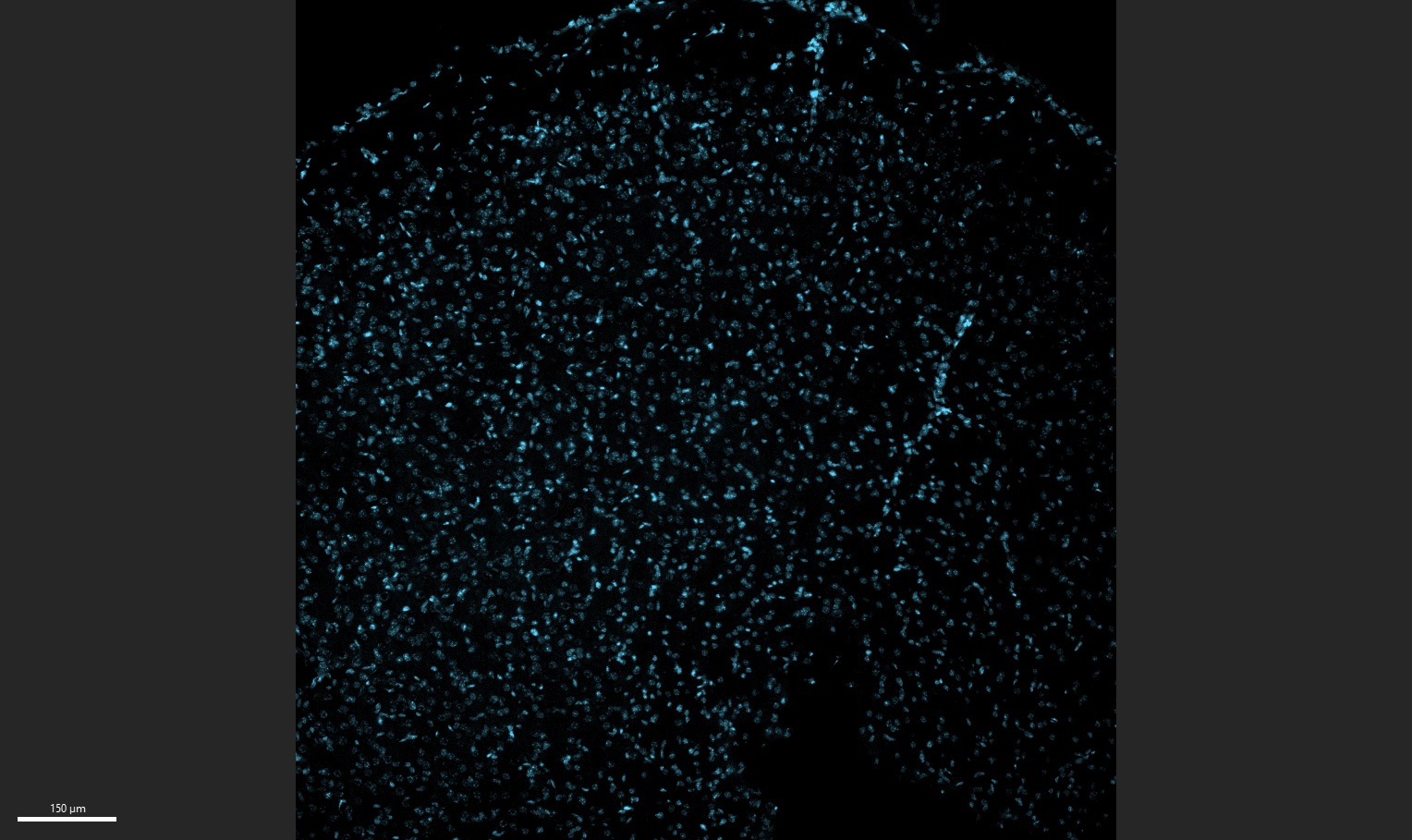

Supplement: Supplementary file 2. [file elife-102900-supp2.zip › Supplementary File 2/Raw RNAScope/1184_ict_wtb6_10x_02_2024-07-30_15.33.25_2024-08-02T11-59-17.313.jpeg]

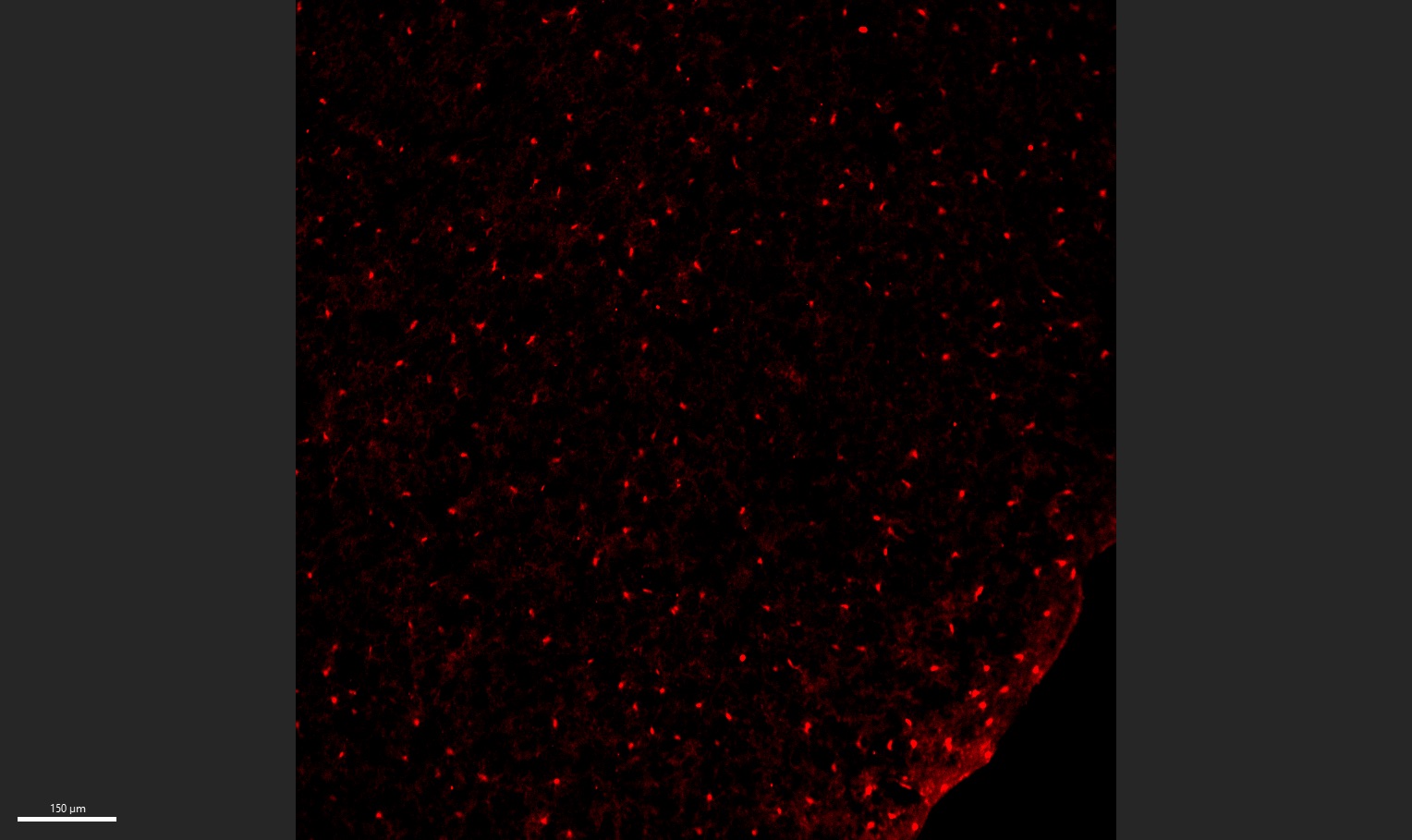

Supplement: Supplementary file 2. [file elife-102900-supp2.zip › Supplementary File 2/Raw RNAScope/1214_tamsham_10x_02_2024-07-30_14.13.16_2024-08-02T11-56-50.455.jpeg]

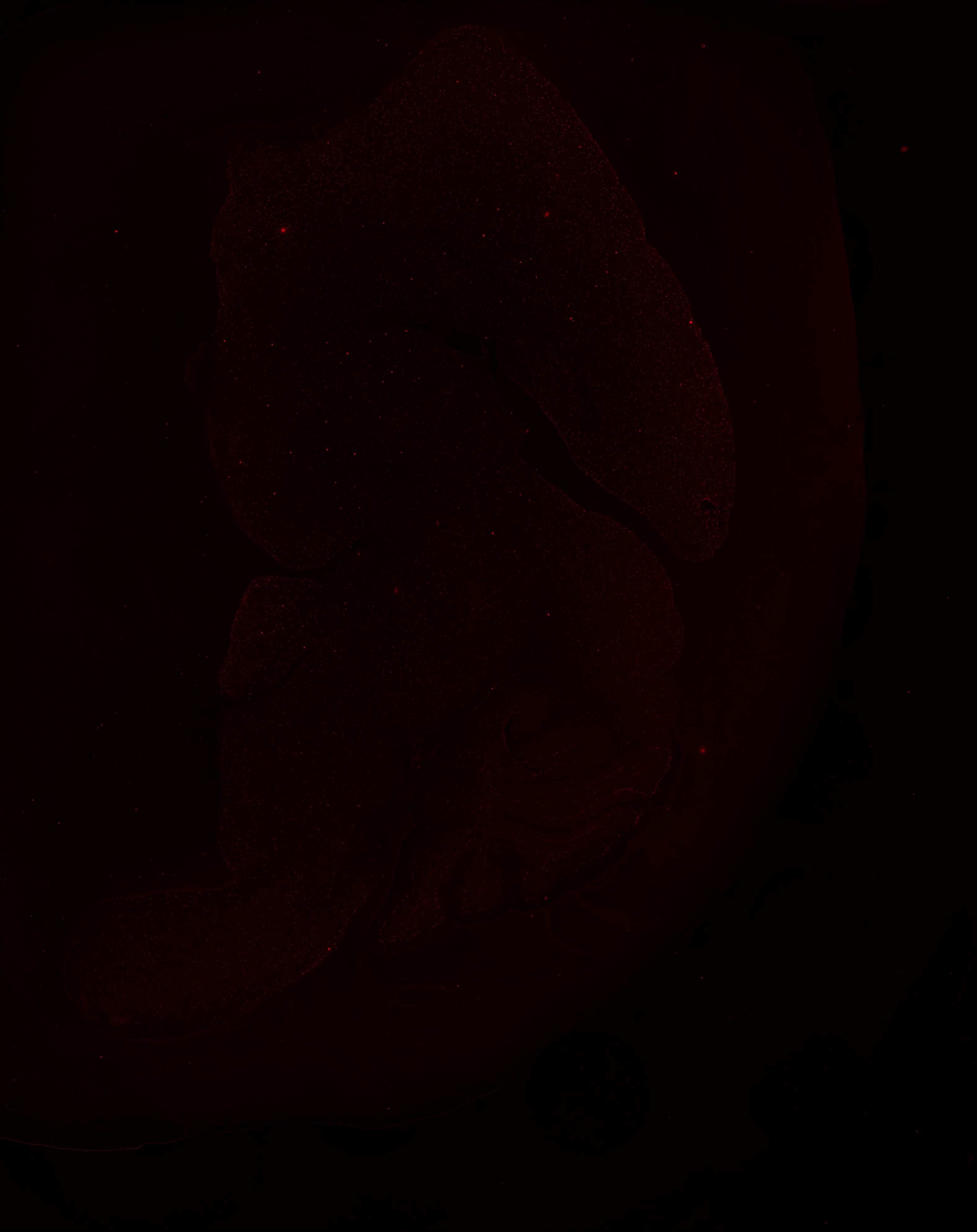

Supplement: Supplementary file 2. [file elife-102900-supp2.zip › Supplementary File 2/Raw Stitches/1144 ICT WT 13dpi 4x Stitch Iba.jpeg]

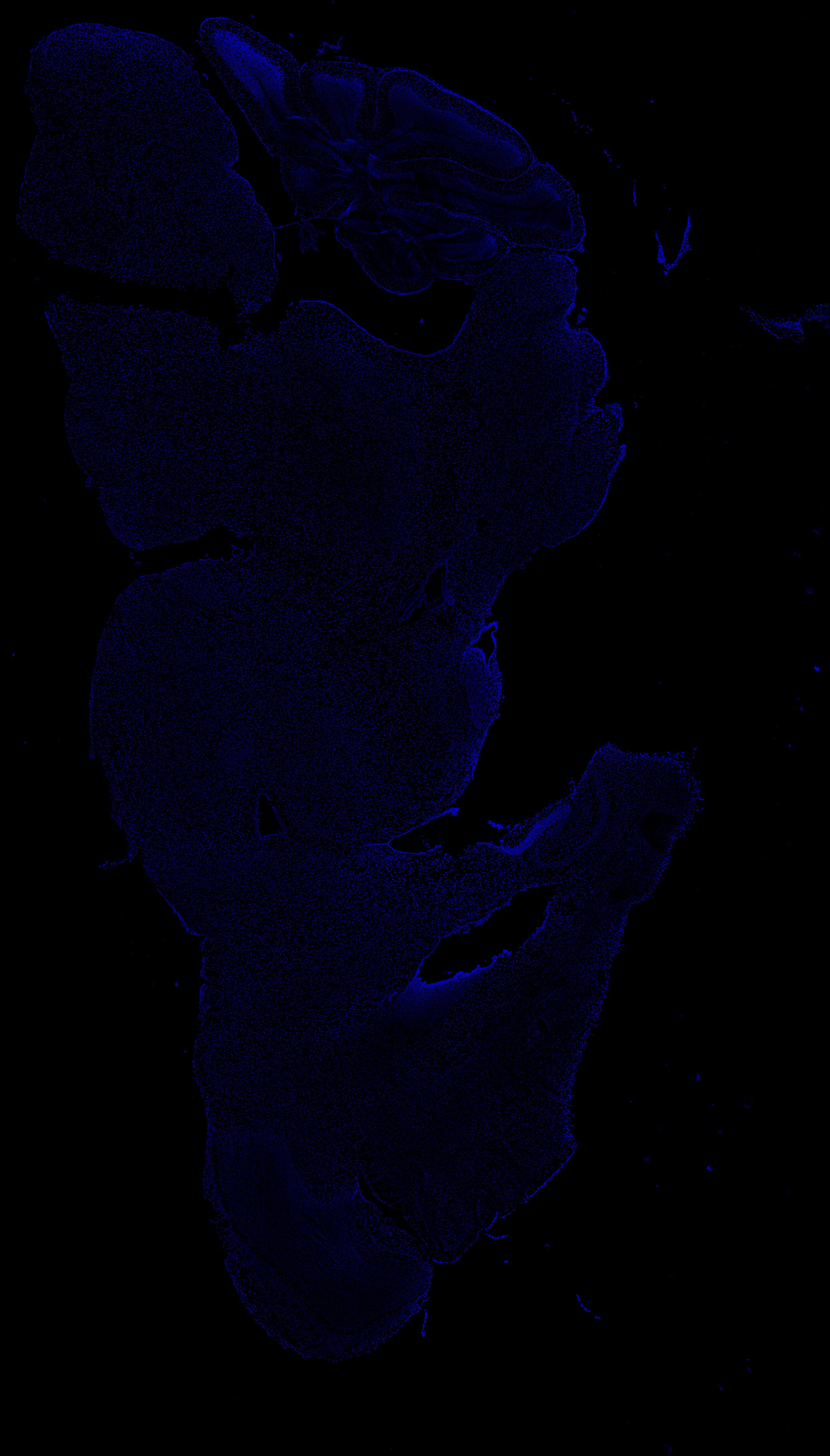

Supplement: Supplementary file 2. [file elife-102900-supp2.zip › Supplementary File 2/Raw Stitches/1071 Stitch DAPI.jpeg]

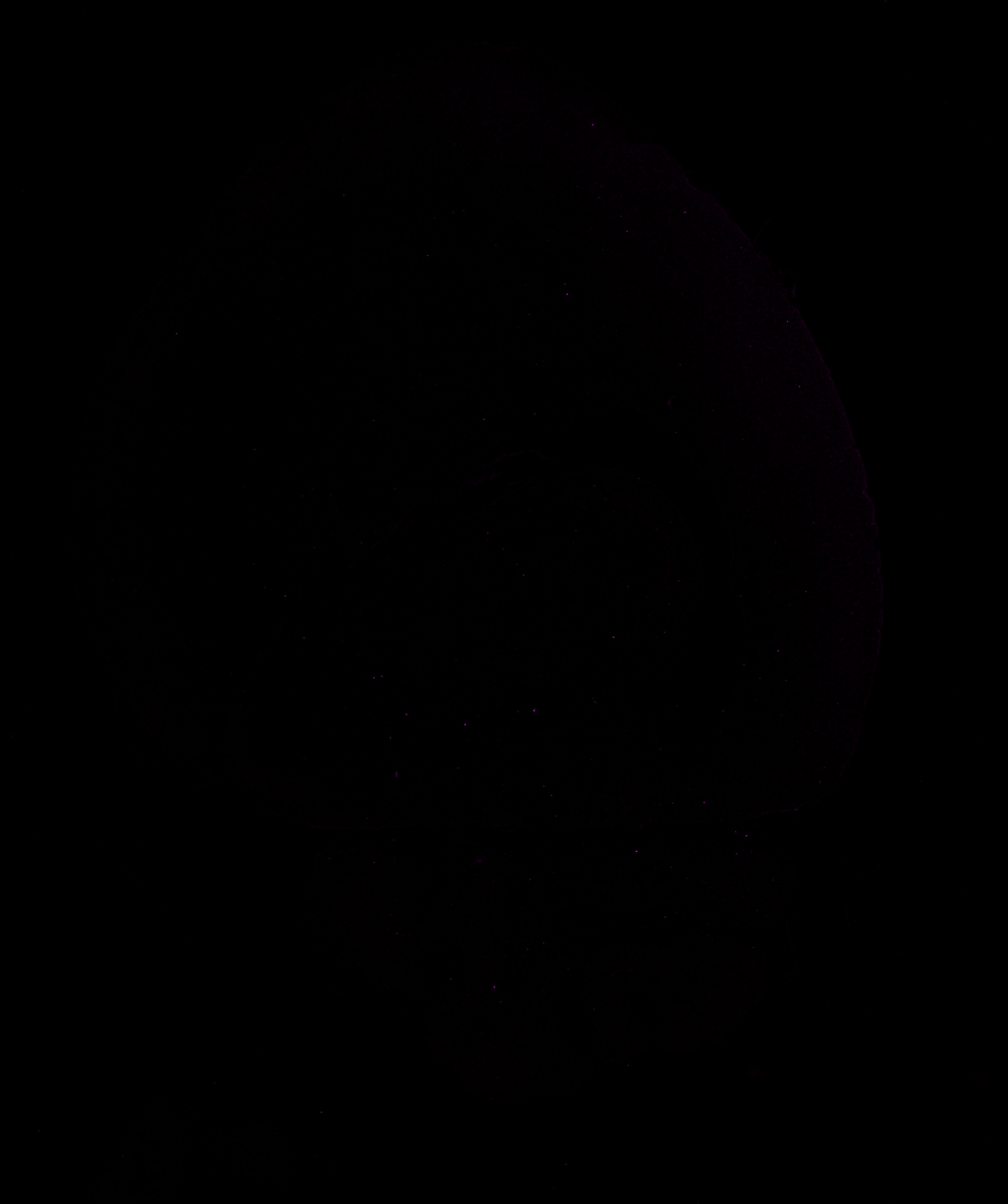

Supplement: Supplementary file 2. [file elife-102900-supp2.zip › Supplementary File 2/Raw Stitches/819 Stitch Isg.jpeg]

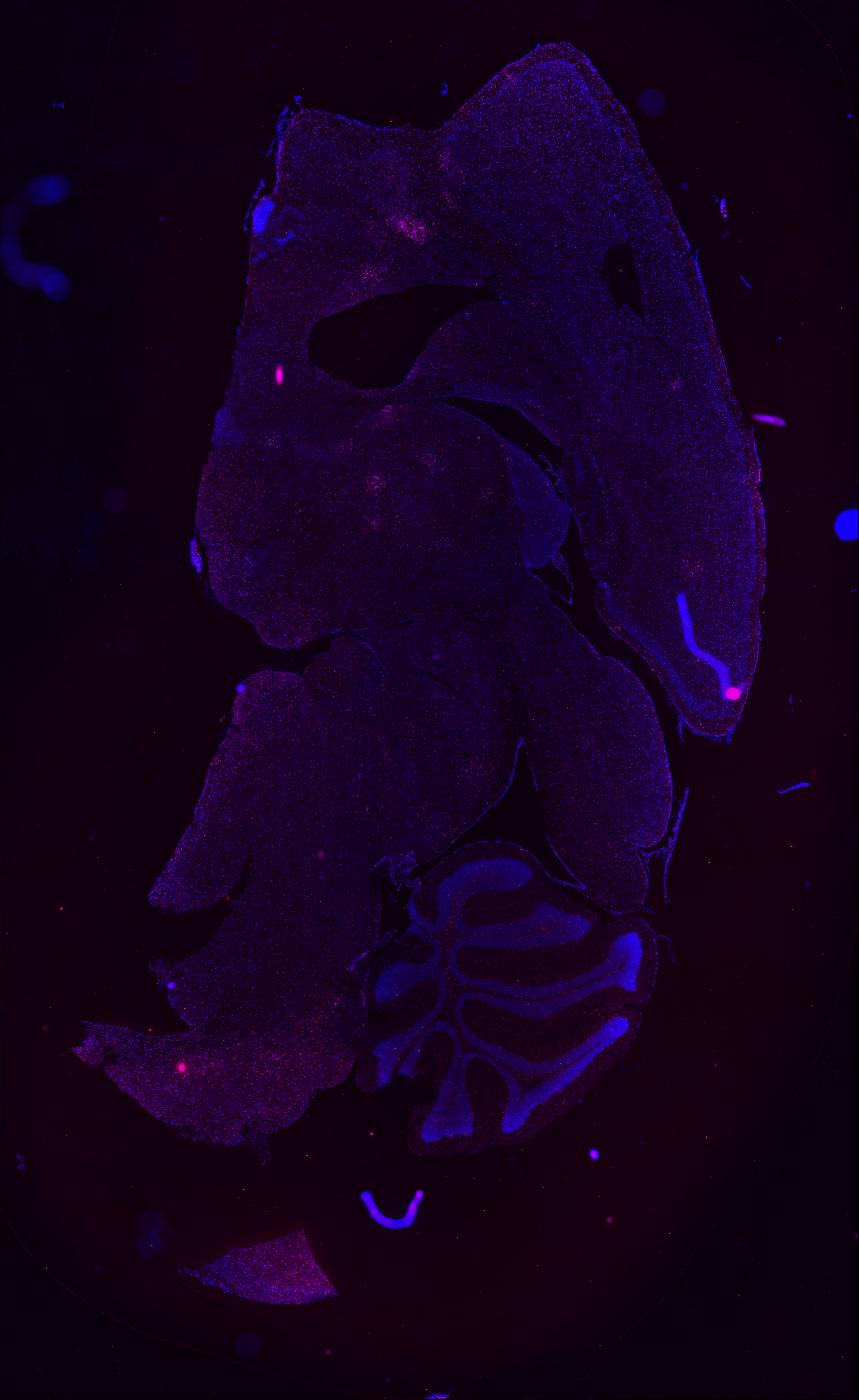

Supplement: Supplementary file 2. [file elife-102900-supp2.zip › Supplementary File 2/Raw Stitches/1186 ICT D1113H 27dpi 4x Stitch Overlay.jpeg]

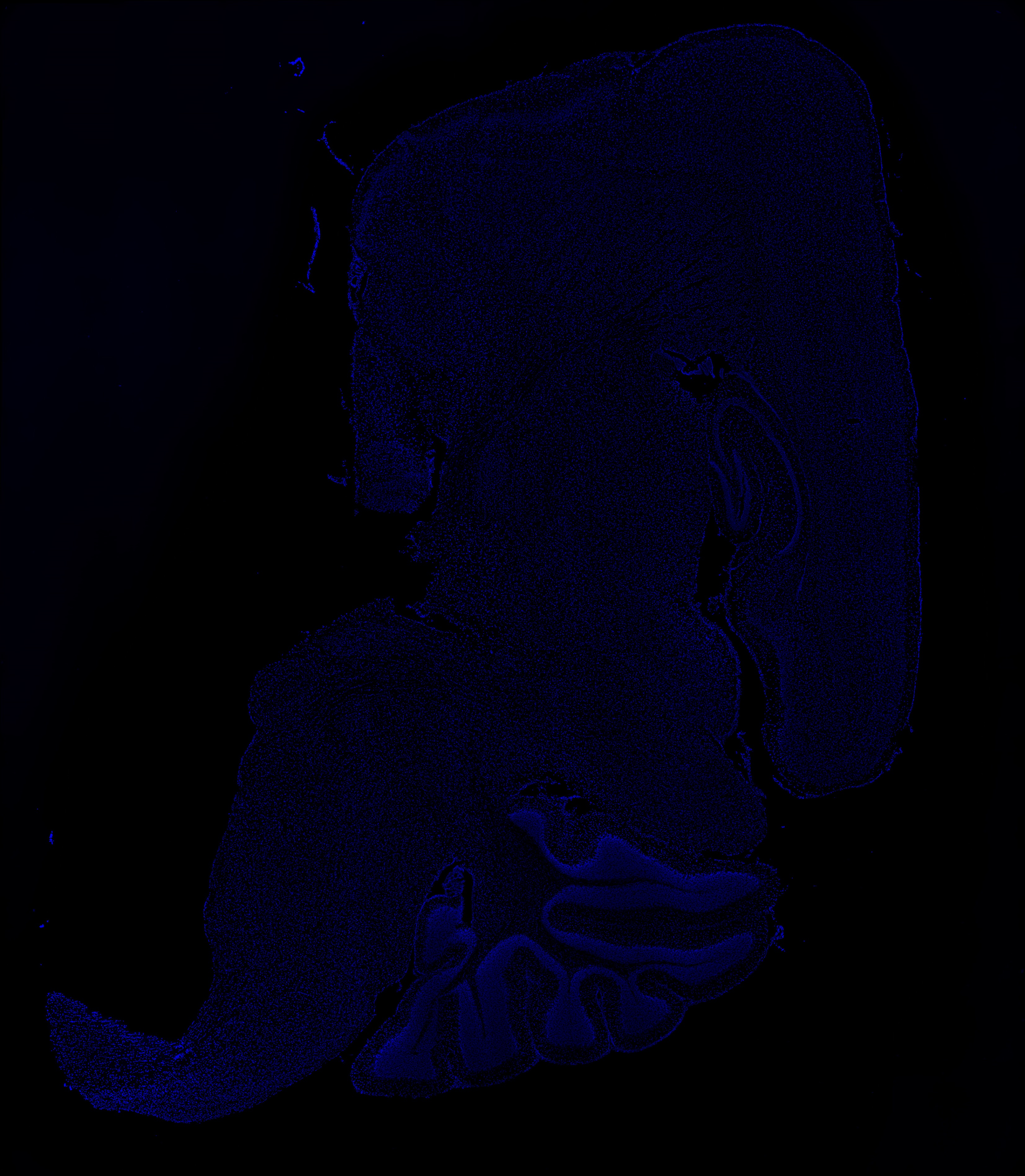

Supplement: Supplementary file 2. [file elife-102900-supp2.zip › Supplementary File 2/Raw Stitches/946 Stitch DAPI.jpeg]

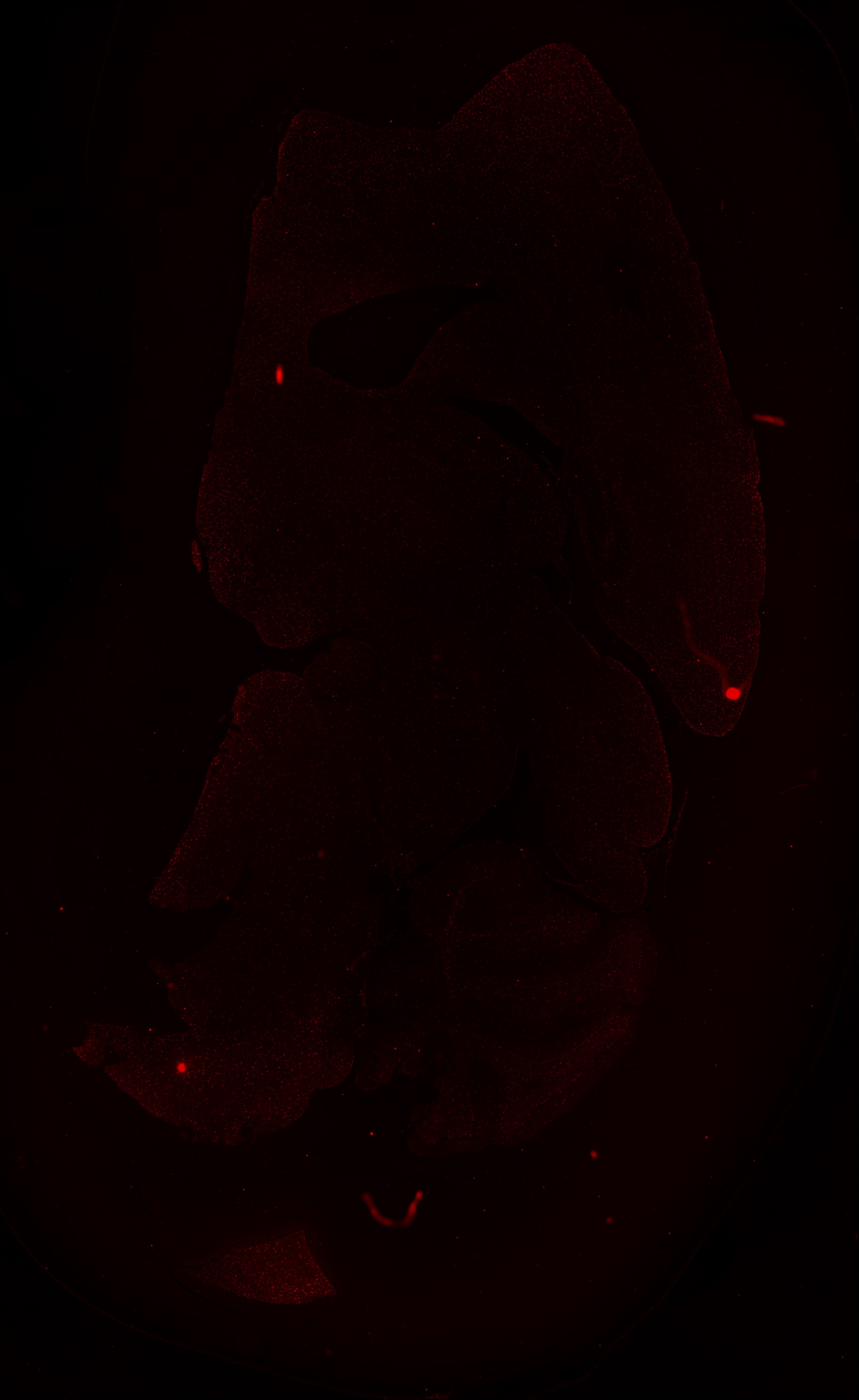

Supplement: Supplementary file 2. [file elife-102900-supp2.zip › Supplementary File 2/Raw Stitches/1186 ICT D1113H 27dpi 4x Stitch Iba.jpeg]

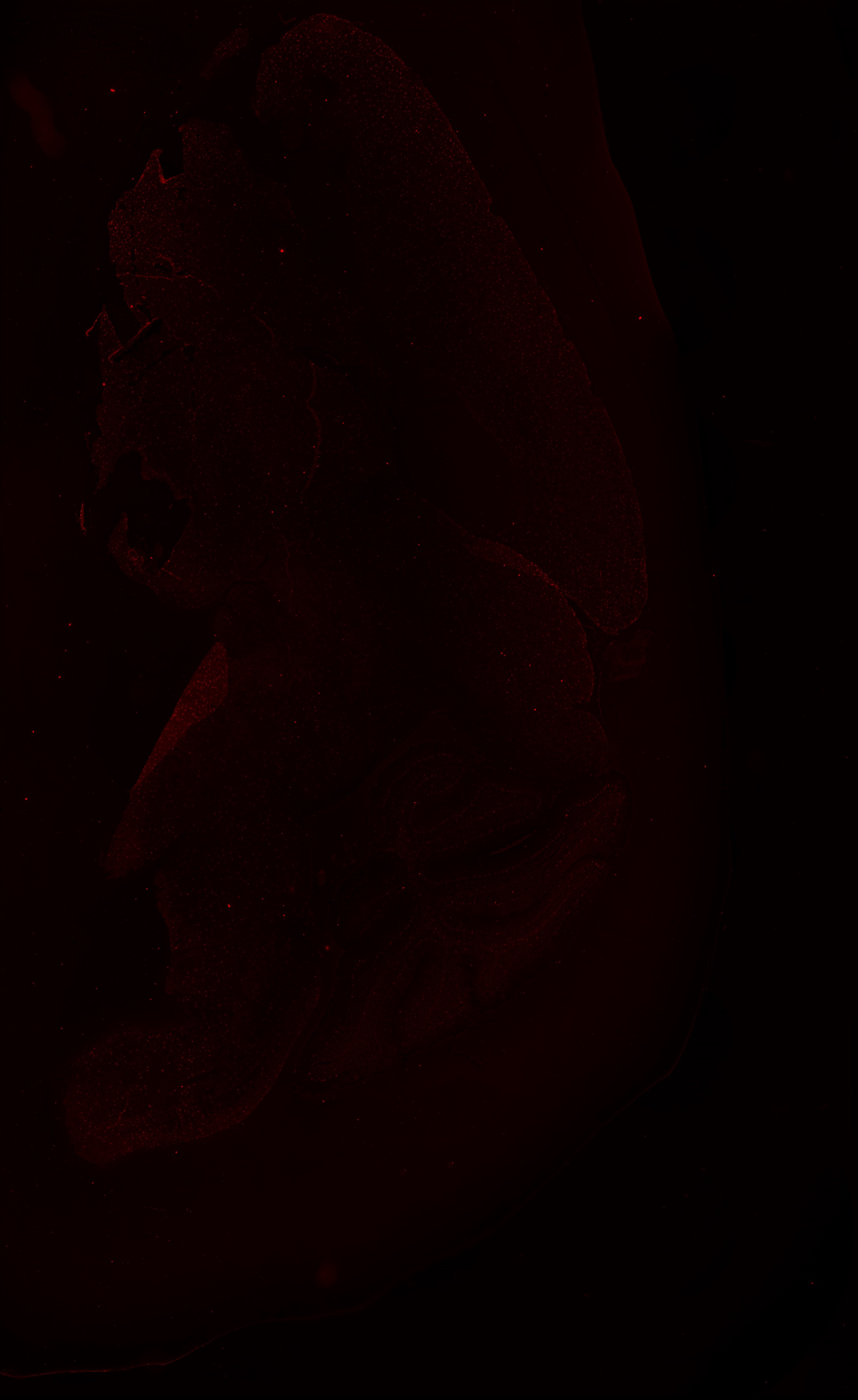

Supplement: Supplementary file 2. [file elife-102900-supp2.zip › Supplementary File 2/Raw Stitches/1152 Tam Sham 14dpi 4x Stitch Iba.jpeg]

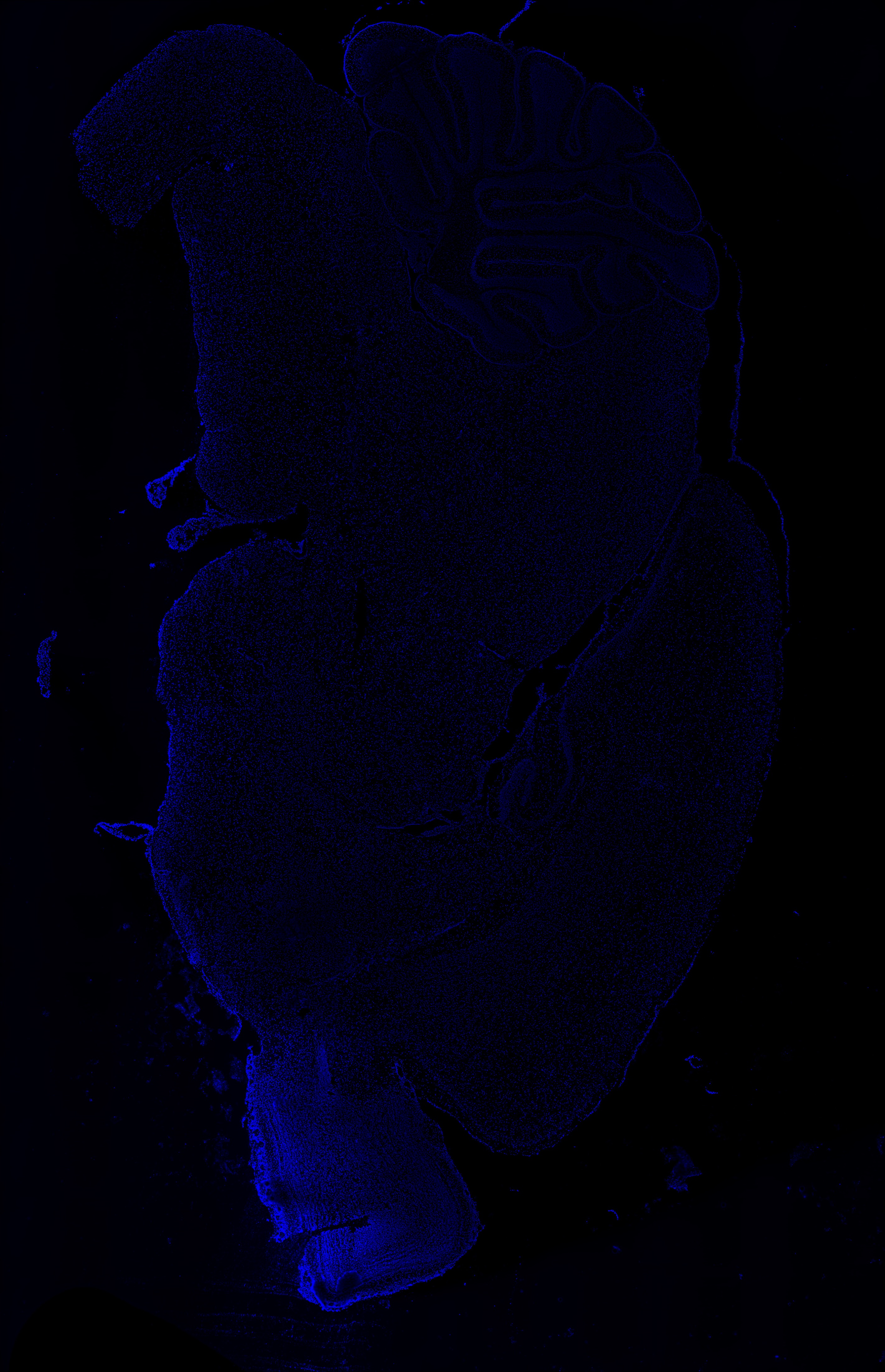

Supplement: Supplementary file 2. [file elife-102900-supp2.zip › Supplementary File 2/Raw Stitches/1083 Stitch DAPI.jpeg]

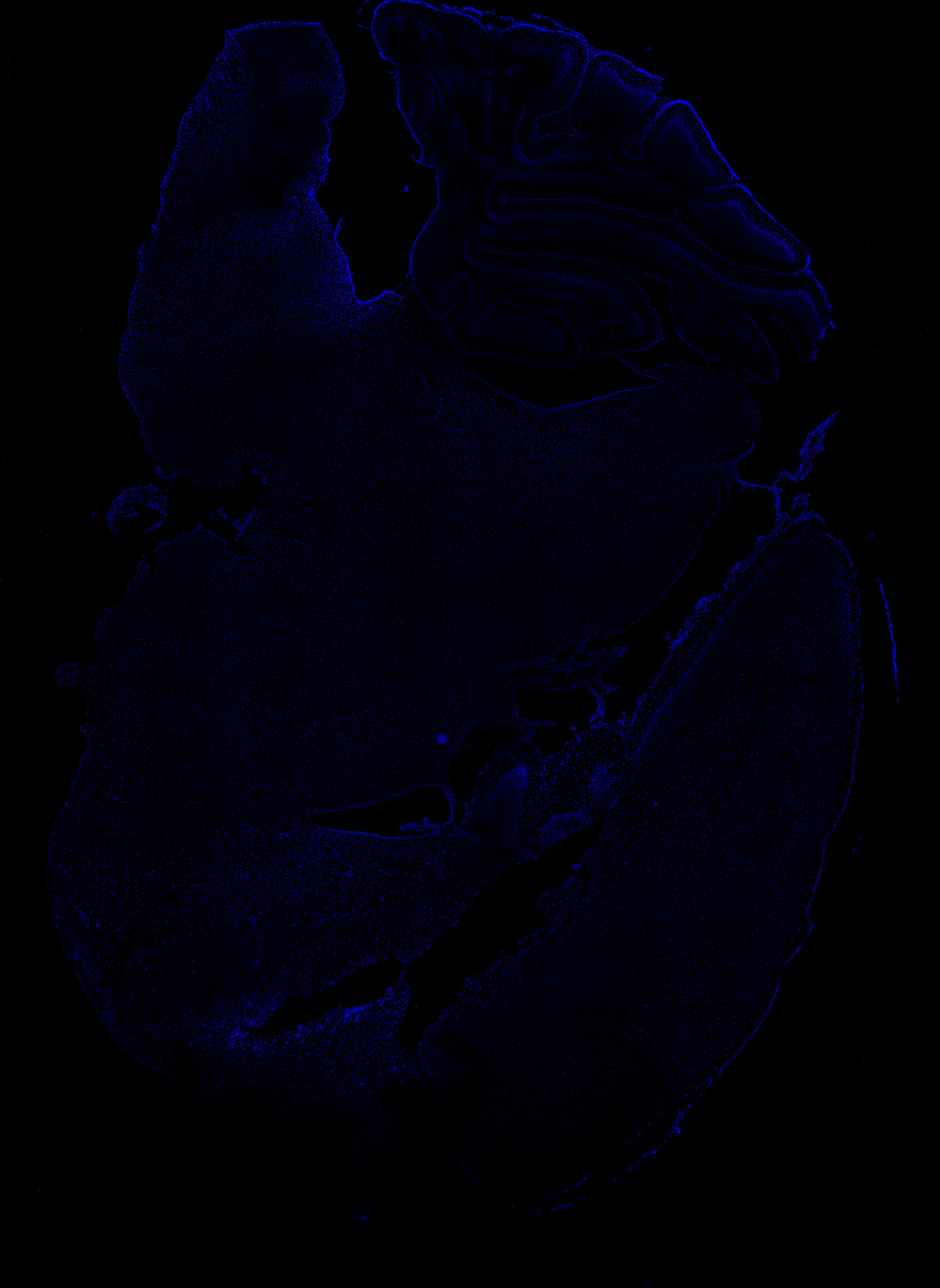

Supplement: Supplementary file 2. [file elife-102900-supp2.zip › Supplementary File 2/Raw Stitches/1239 Stitch DAPI.jpeg]

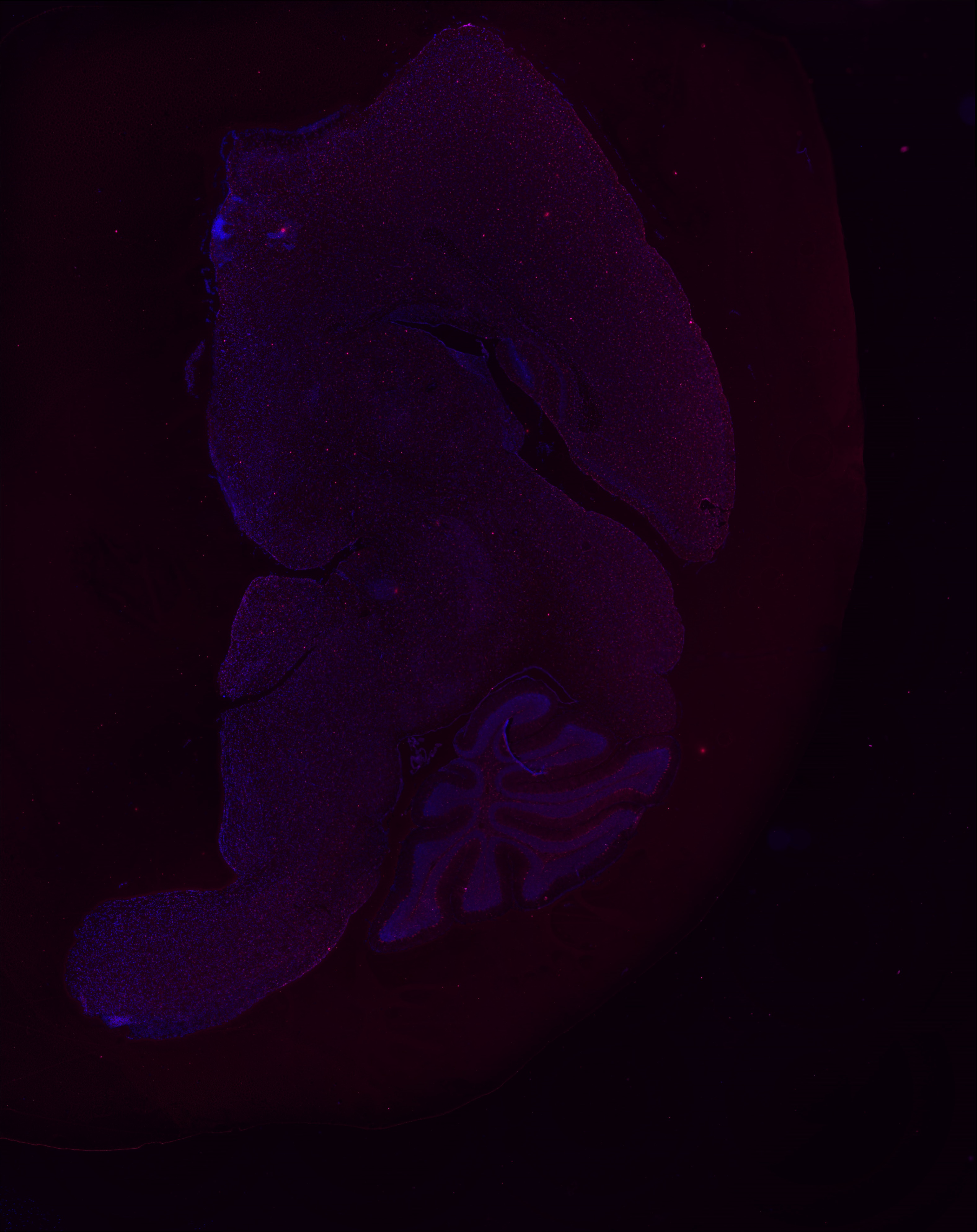

Supplement: Supplementary file 2. [file elife-102900-supp2.zip › Supplementary File 2/Raw Stitches/1144 ICT WT 13dpi 4x Stitch Overlay.jpeg]

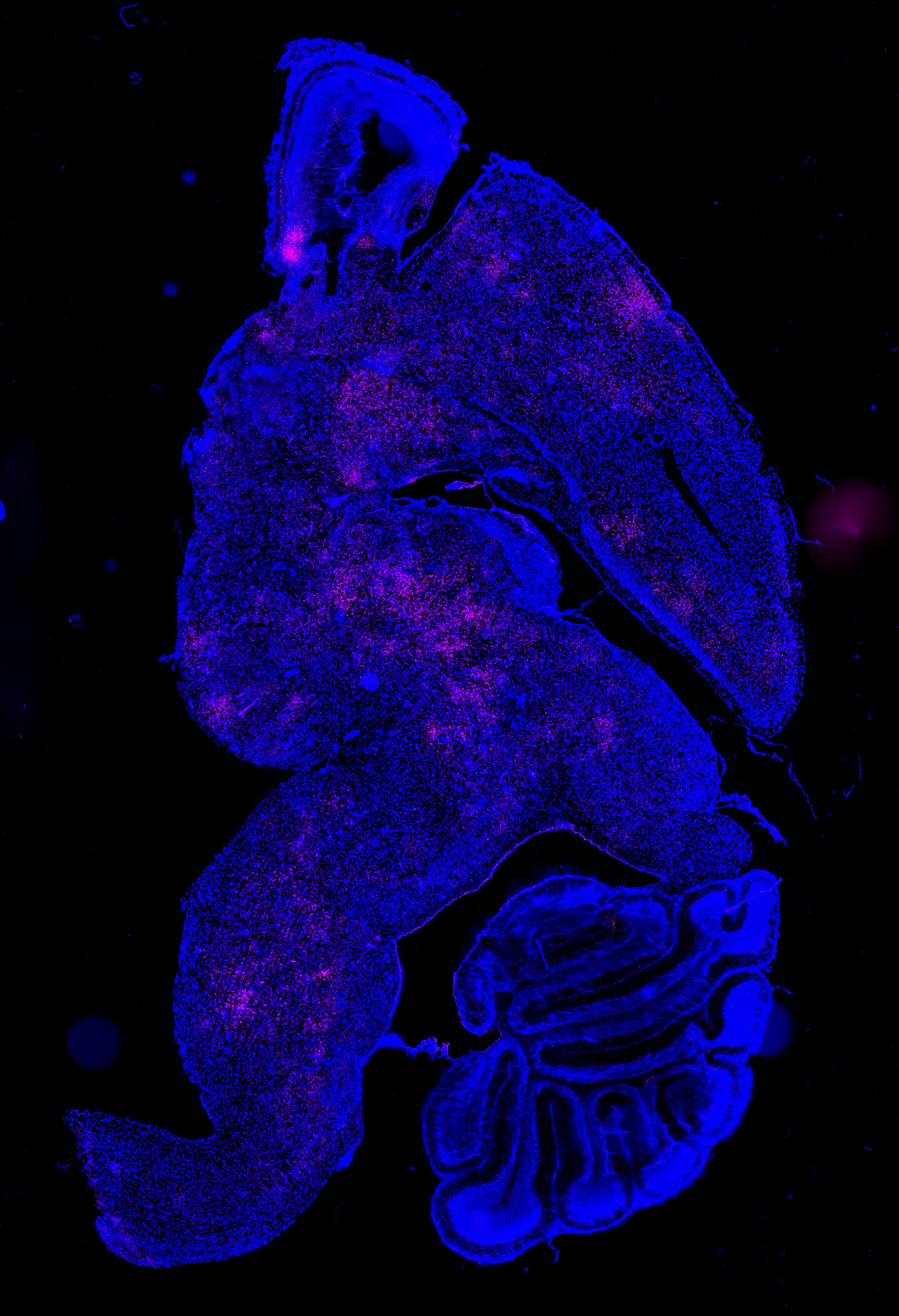

Supplement: Supplementary file 2. [file elife-102900-supp2.zip › Supplementary File 2/Raw Stitches/1264 Stitch Overlay 2.jpeg]

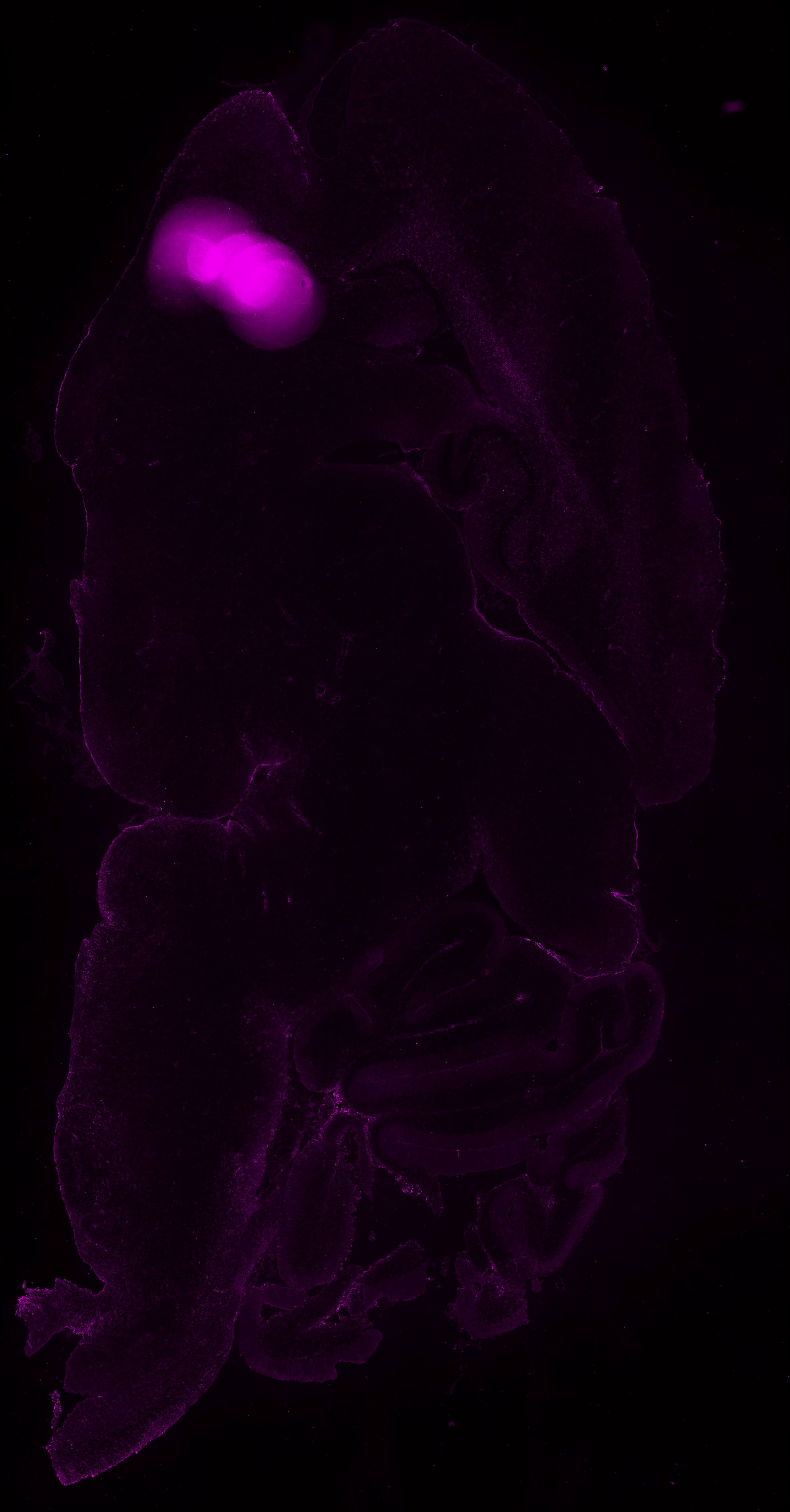

Supplement: Supplementary file 2. [file elife-102900-supp2.zip › Supplementary File 2/Raw Stitches/745.3 Stitch GFAP.jpeg]

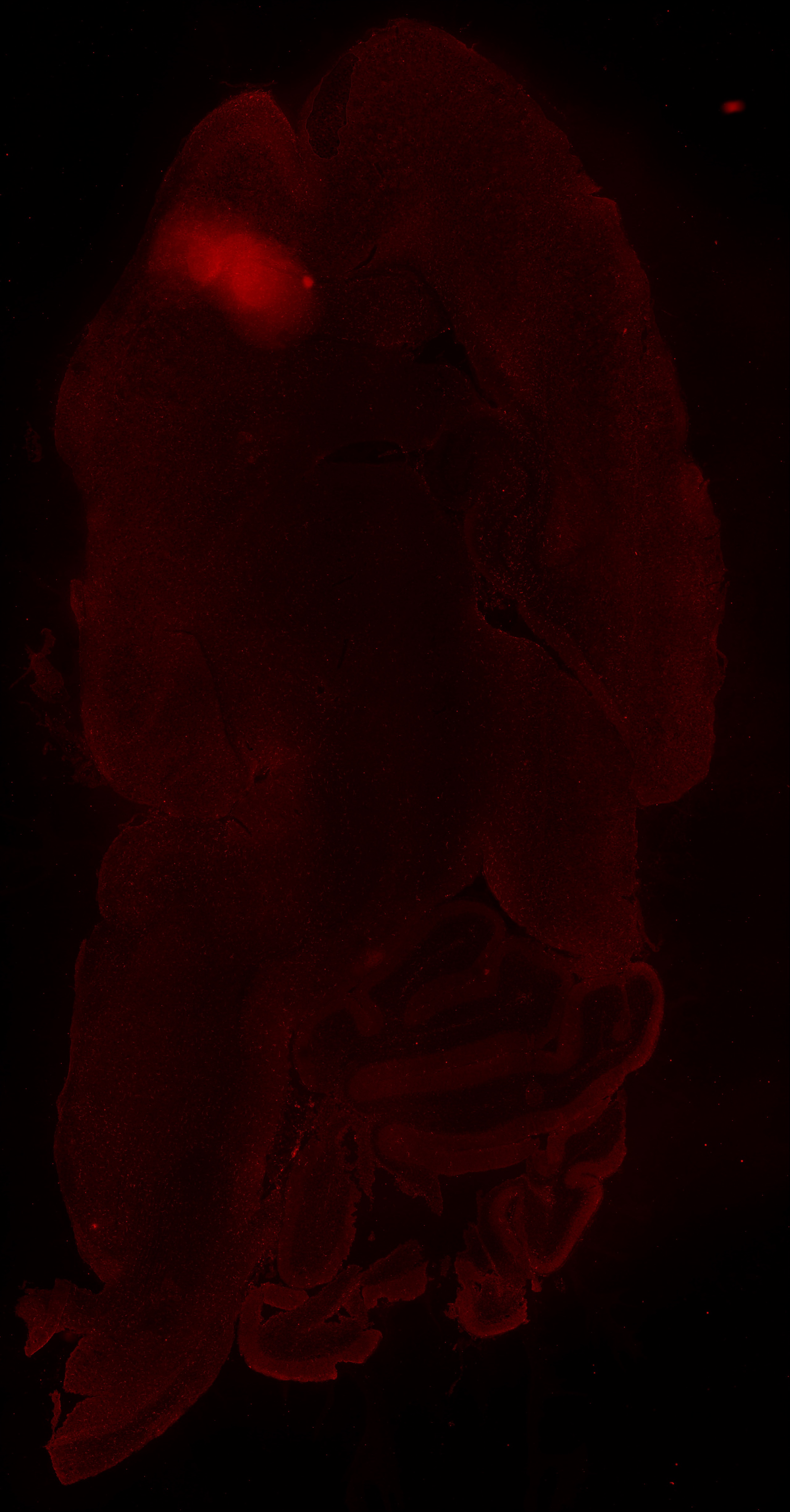

Supplement: Supplementary file 2. [file elife-102900-supp2.zip › Supplementary File 2/Raw Stitches/745.3 Stitch Iba1.jpeg]

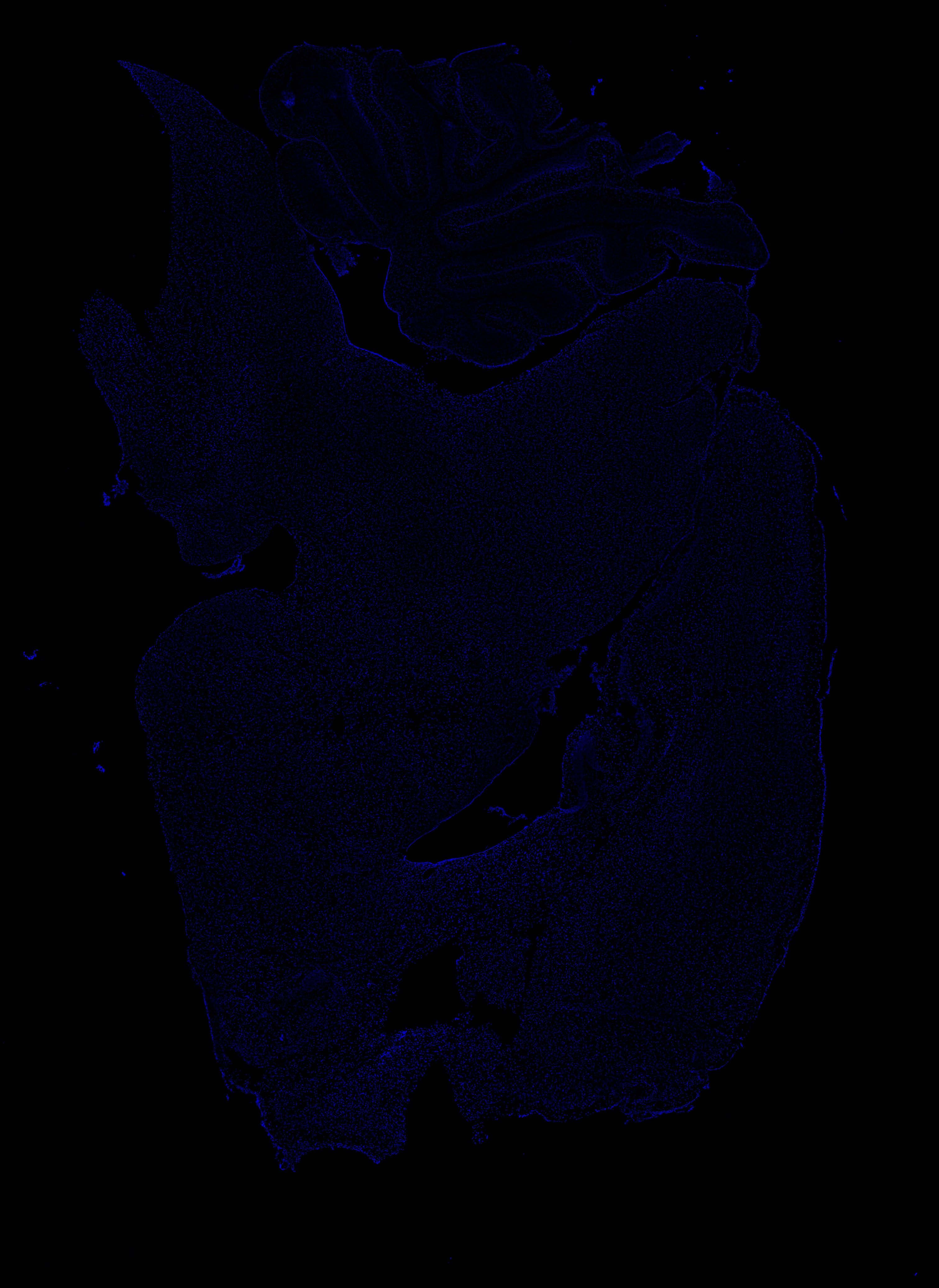

Supplement: Supplementary file 2. [file elife-102900-supp2.zip › Supplementary File 2/Raw Stitches/1237 Stitch DAPI.jpeg]

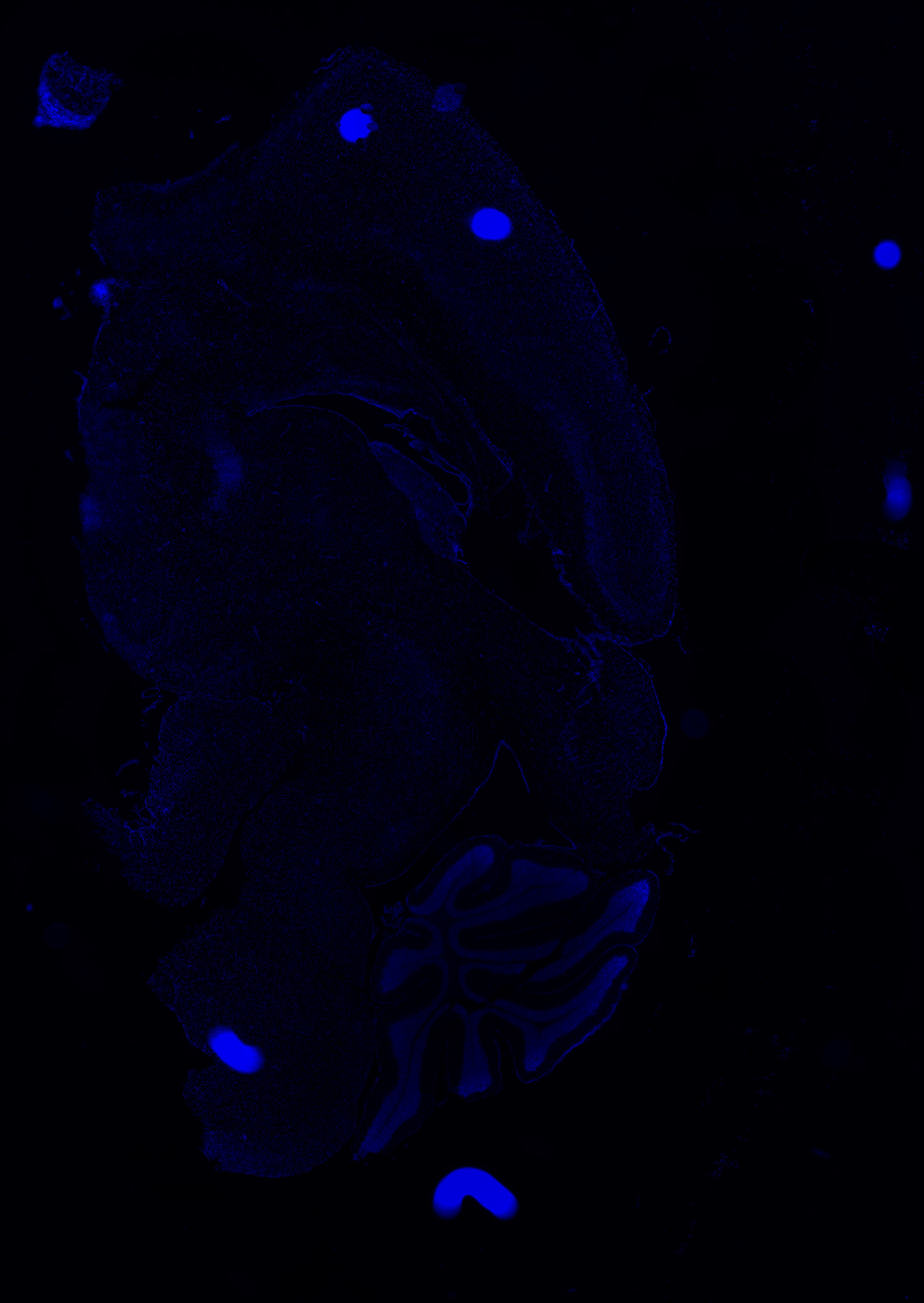

Supplement: Supplementary file 2. [file elife-102900-supp2.zip › Supplementary File 2/Raw Stitches/1138 ICT D1113H 13d 4x Stitch DAPI.jpeg]

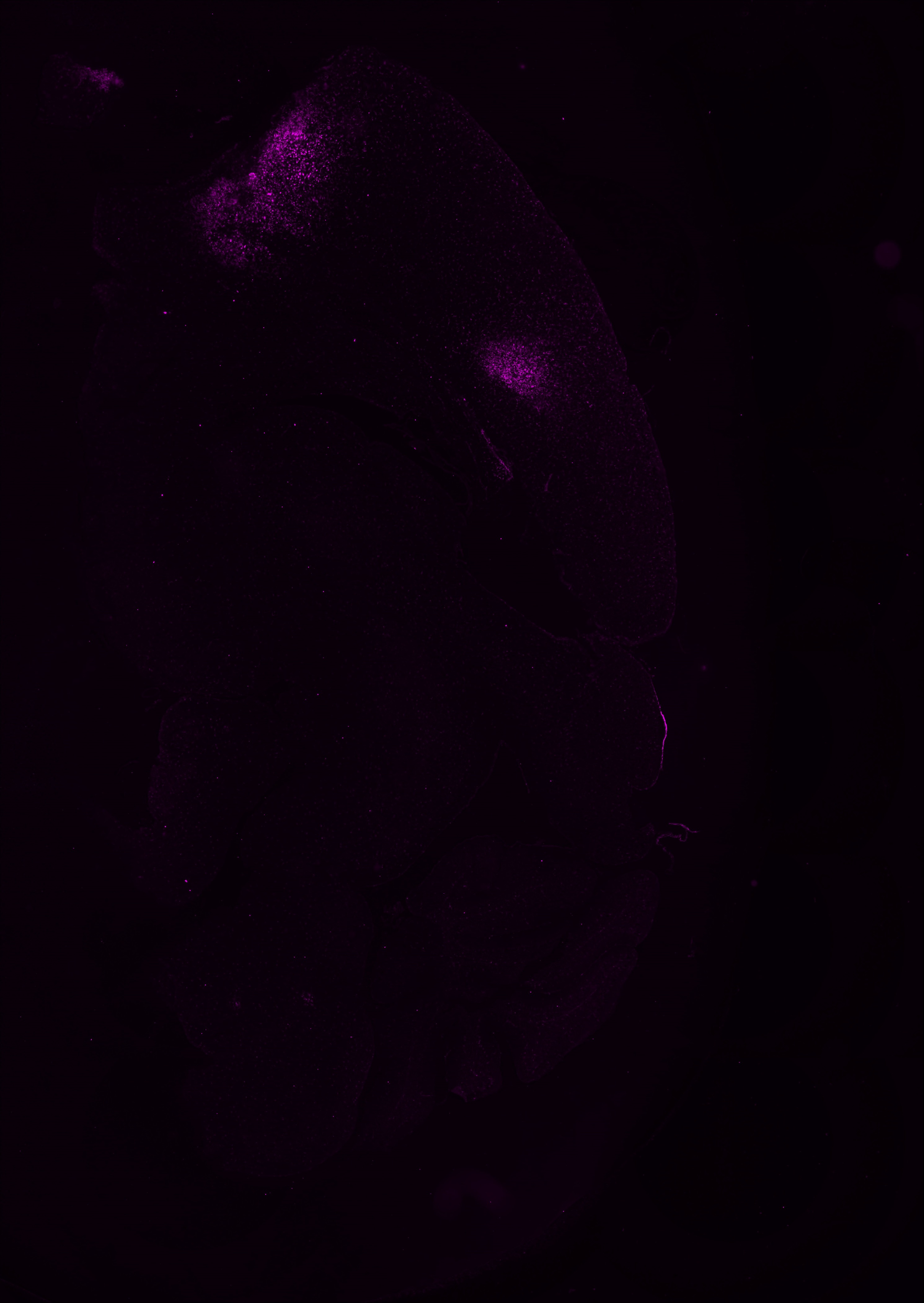

Supplement: Supplementary file 2. [file elife-102900-supp2.zip › Supplementary File 2/Raw Stitches/1138 ICT D1113H 13d 4x Stitch Isg.jpeg]

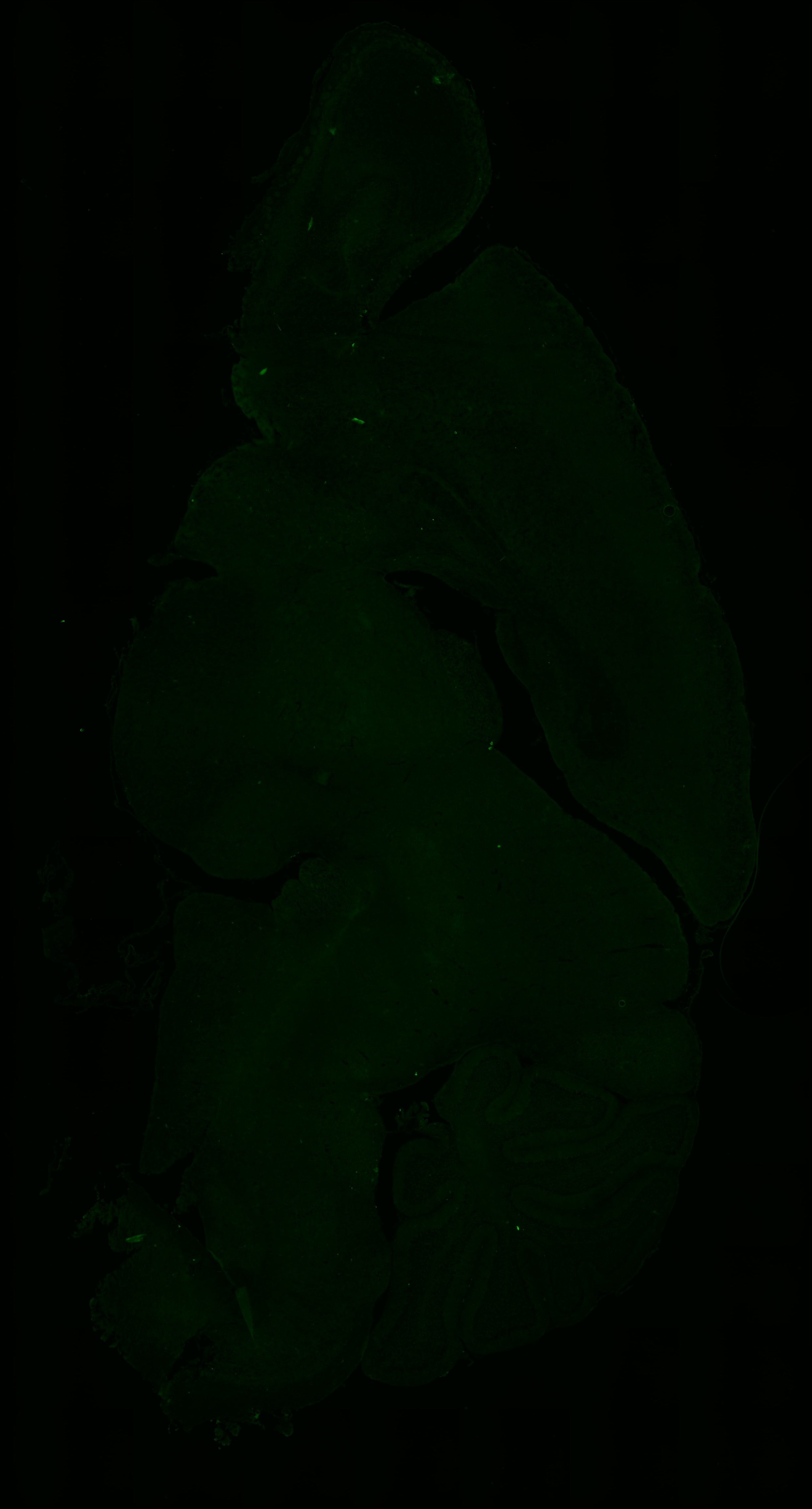

Supplement: Supplementary file 2. [file elife-102900-supp2.zip › Supplementary File 2/Raw Stitches/1017 Stitch GFP.jpeg]

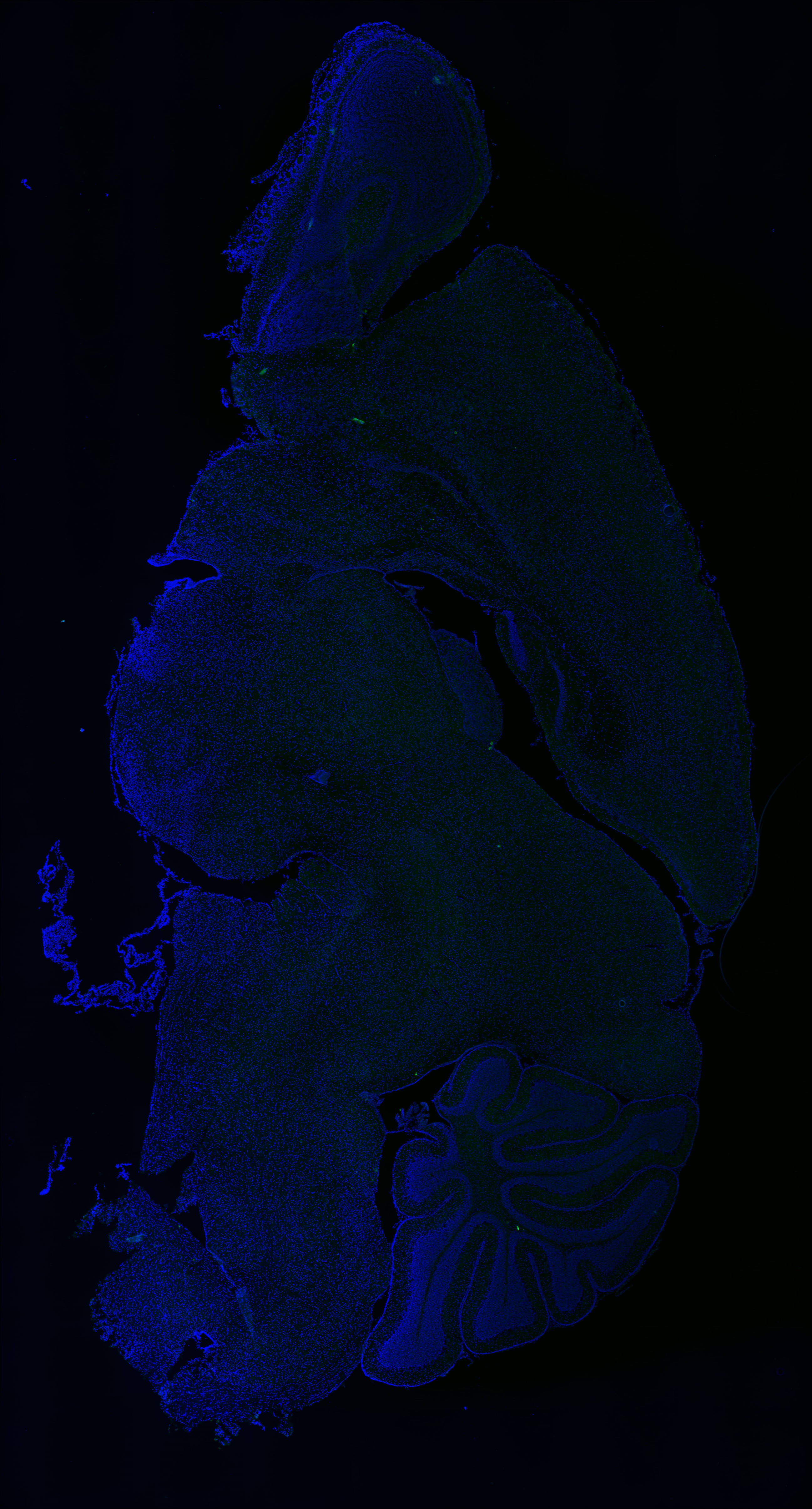

Supplement: Supplementary file 2. [file elife-102900-supp2.zip › Supplementary File 2/Raw Stitches/1017 Stitch Overlay.jpeg]

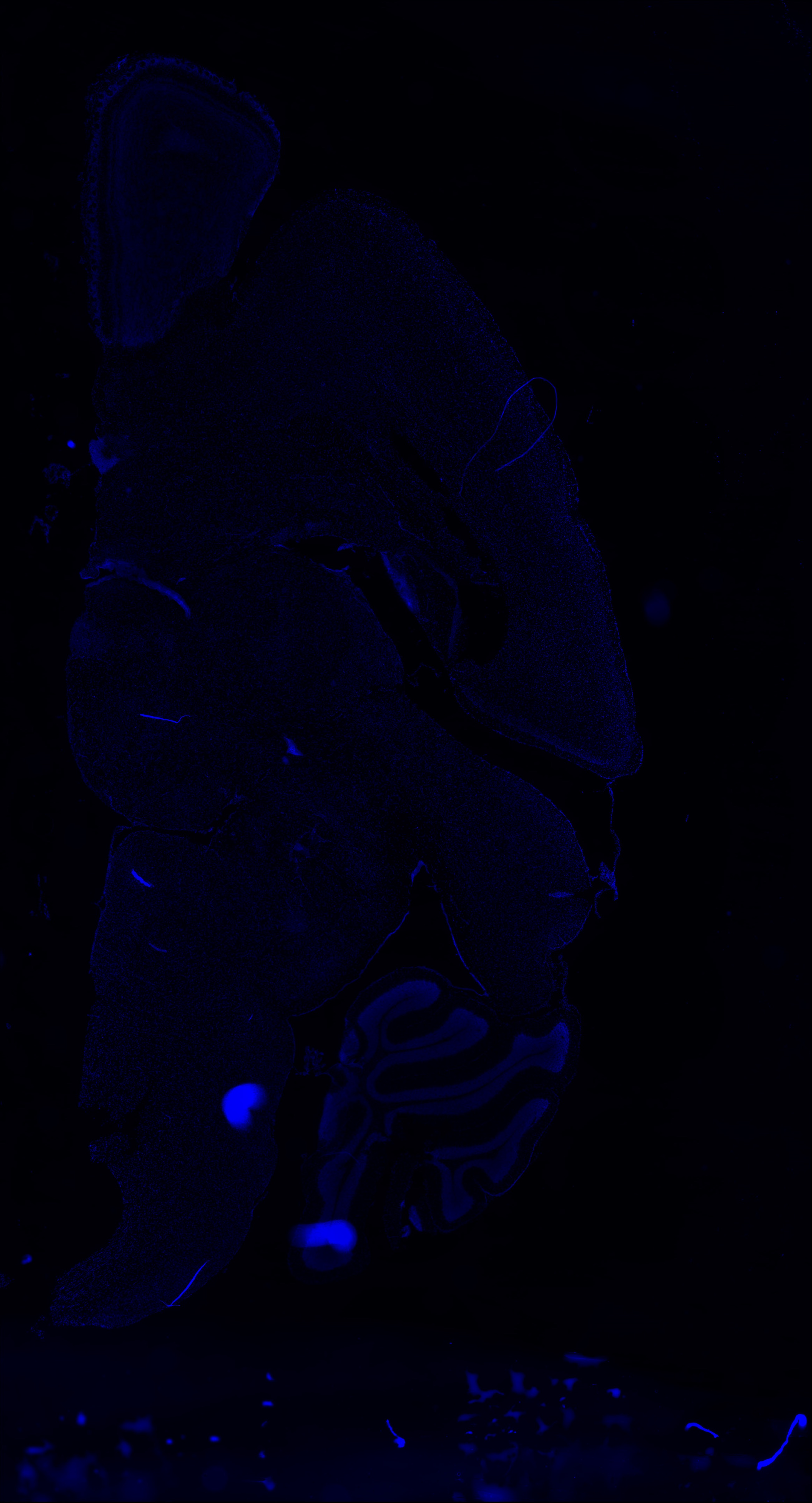

Supplement: Supplementary file 2. [file elife-102900-supp2.zip › Supplementary File 2/Raw Stitches/1180 Full D1113H 14d 4x Stitch DAPI.jpeg]

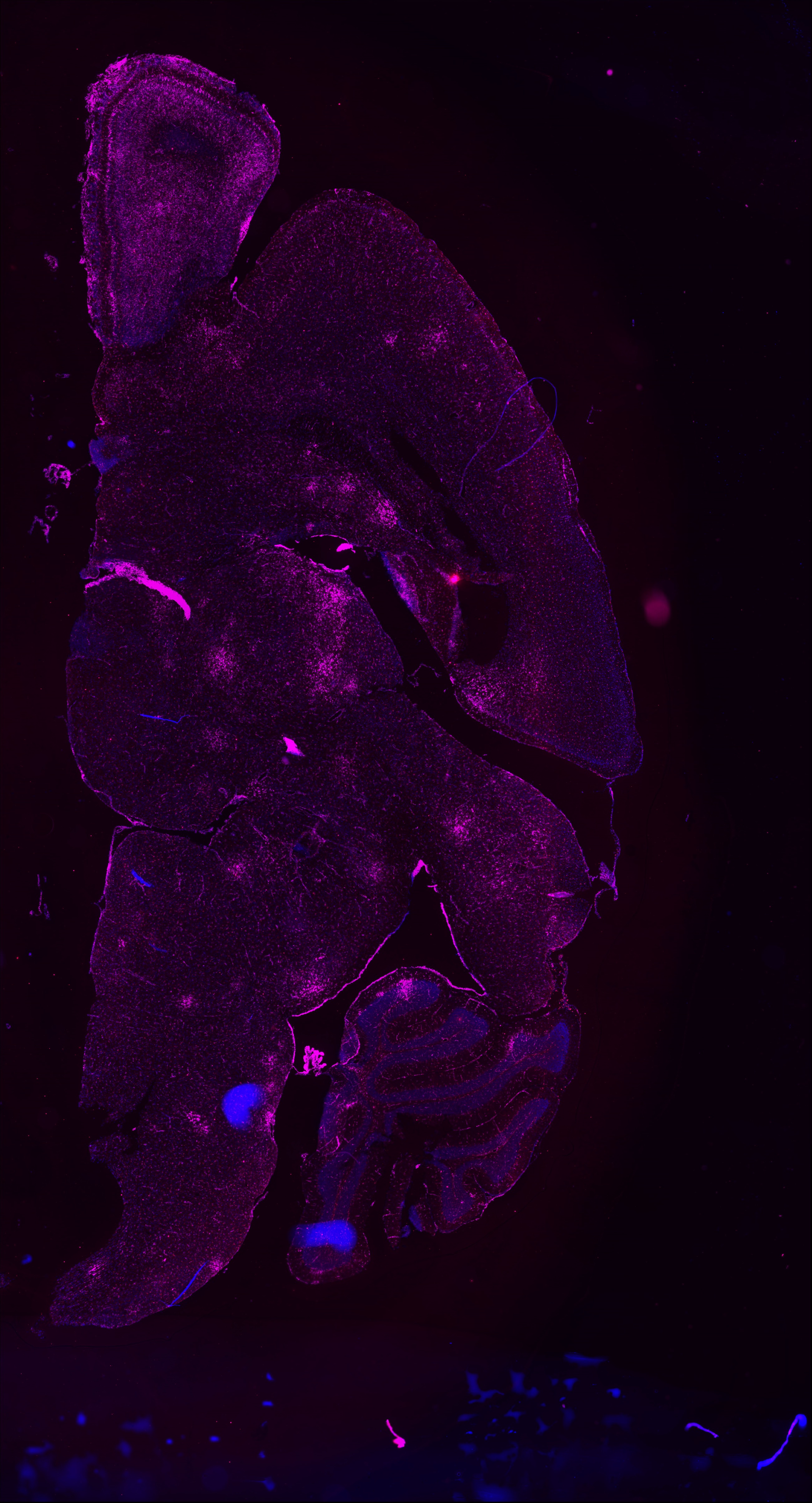

Supplement: Supplementary file 2. [file elife-102900-supp2.zip › Supplementary File 2/Raw Stitches/1180 Full D1113H 14d 4x Stitch Overlay.jpeg]

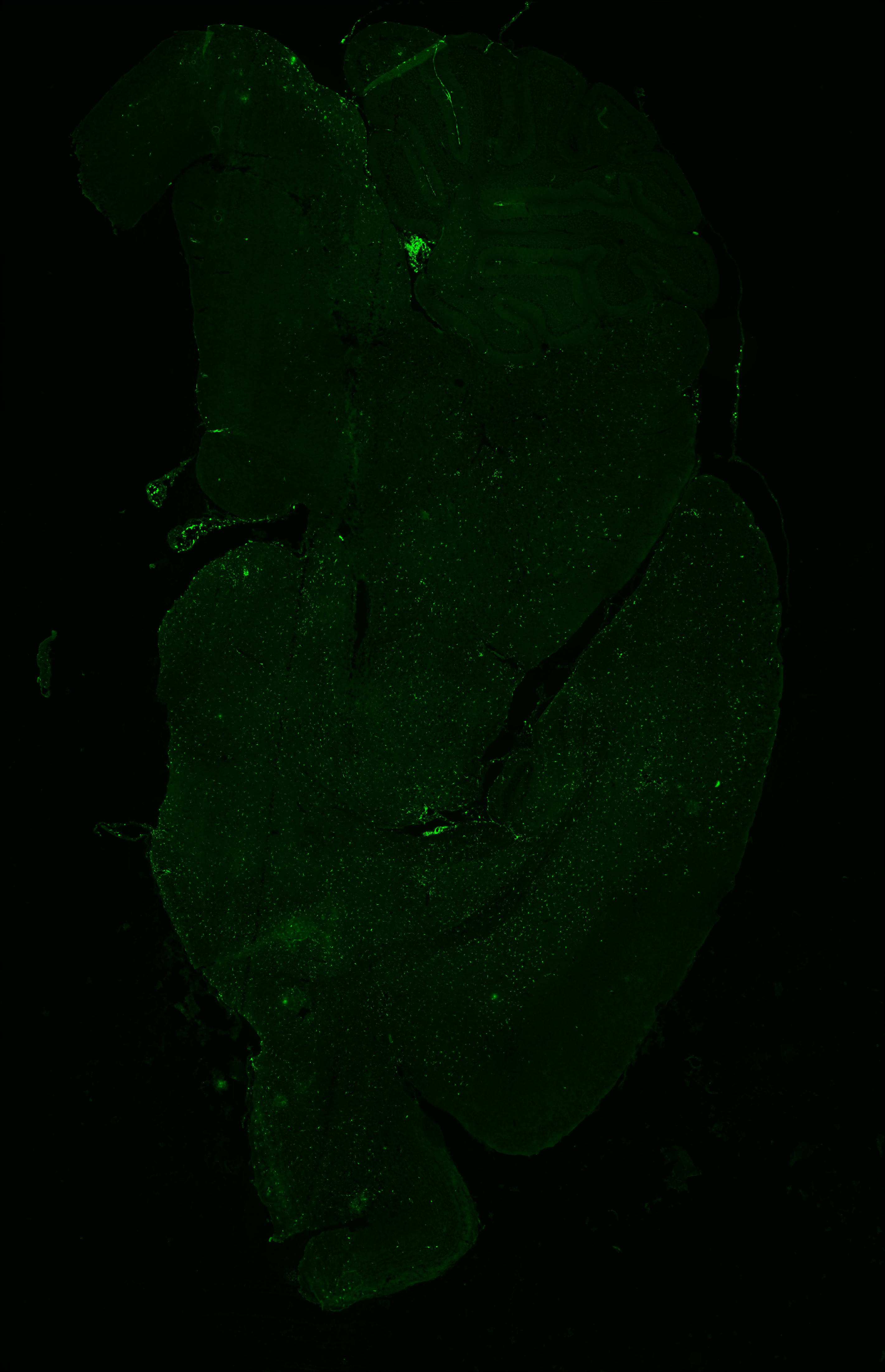

Supplement: Supplementary file 2. [file elife-102900-supp2.zip › Supplementary File 2/Raw Stitches/1083 Stitch GFP.jpeg]

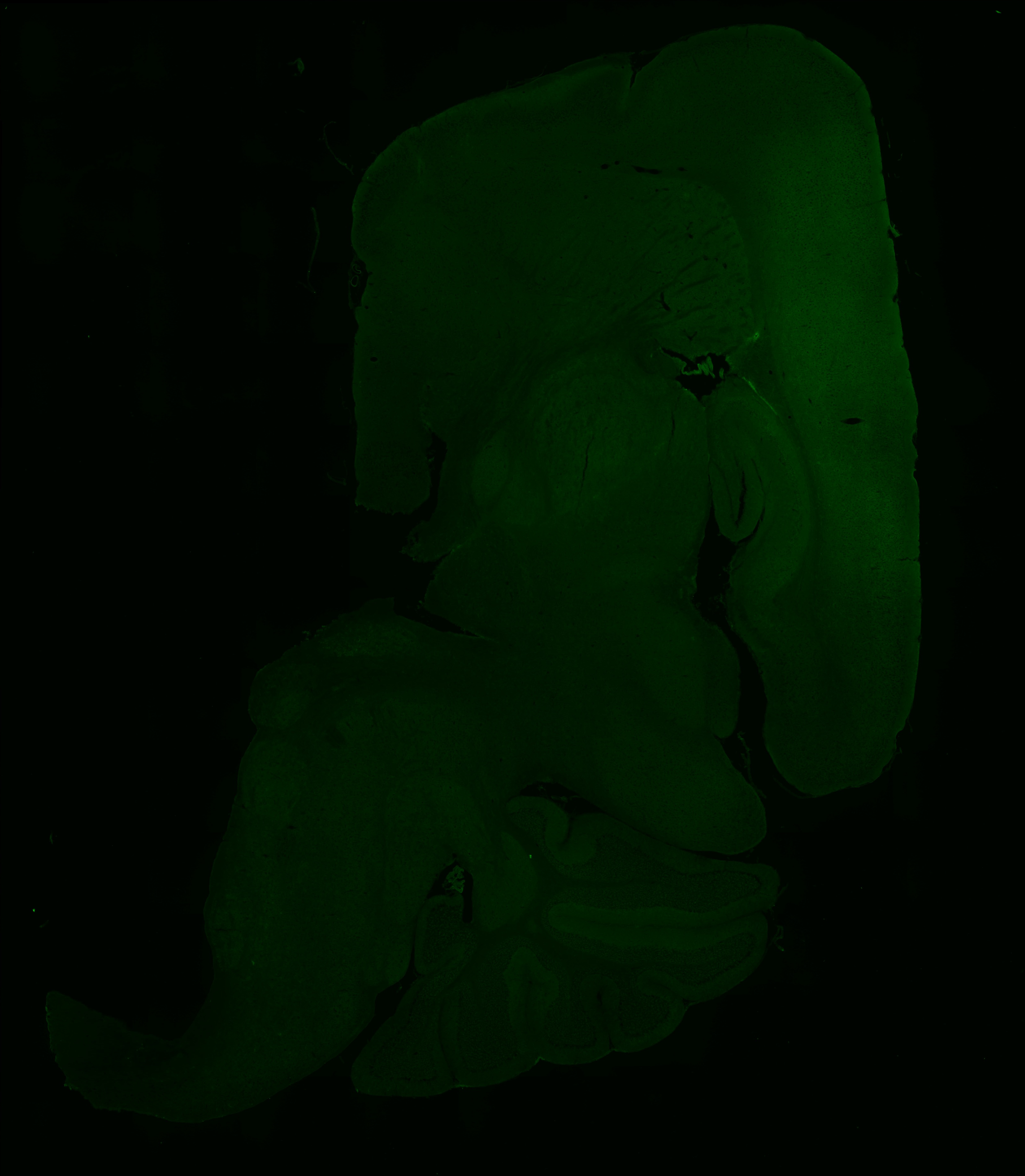

Supplement: Supplementary file 2. [file elife-102900-supp2.zip › Supplementary File 2/Raw Stitches/946 Stitch GFP.jpeg]

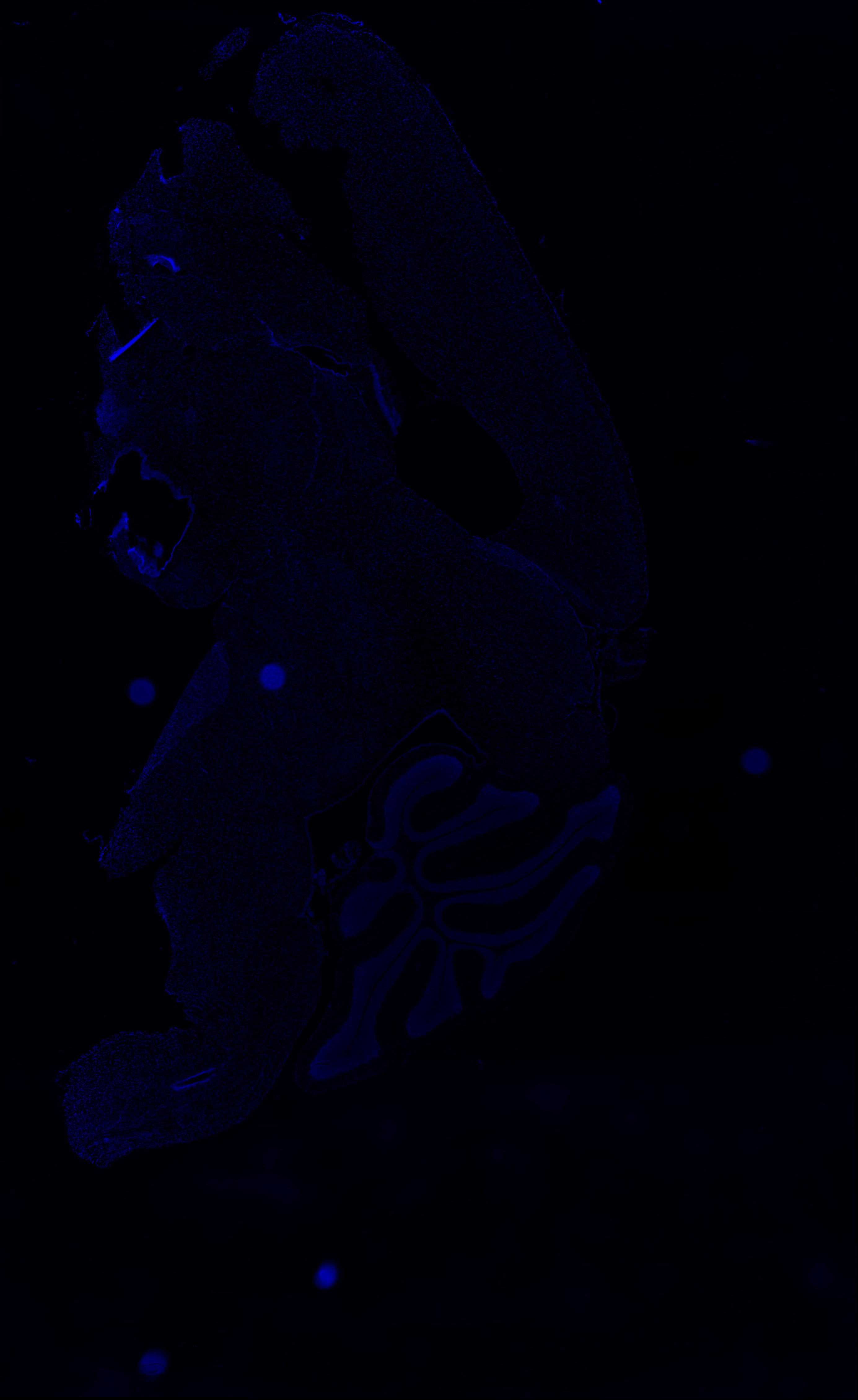

Supplement: Supplementary file 2. [file elife-102900-supp2.zip › Supplementary File 2/Raw Stitches/1152 Tam Sham 14dpi 4x Stitch DAPI.jpeg]

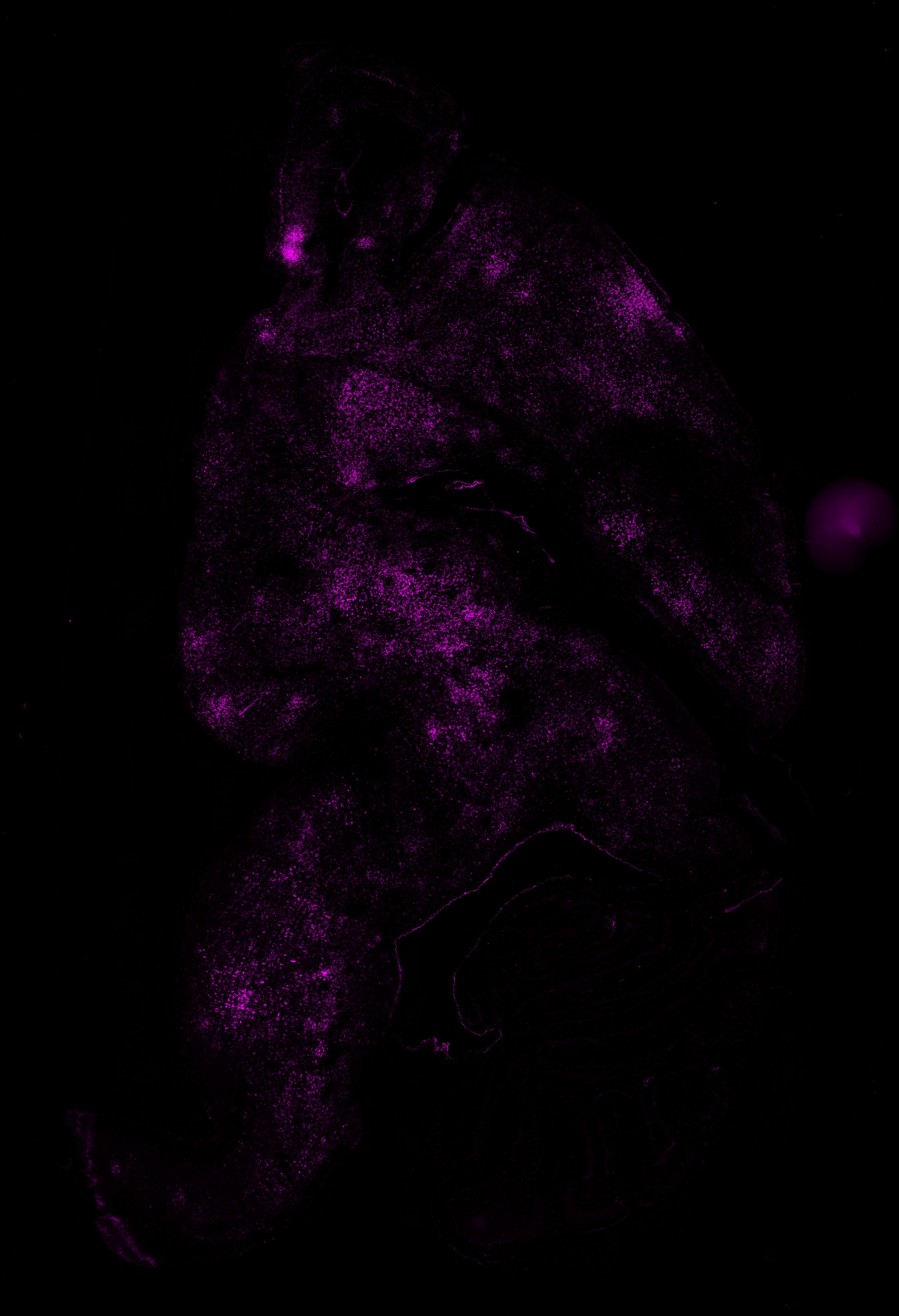

Supplement: Supplementary file 2. [file elife-102900-supp2.zip › Supplementary File 2/Raw Stitches/1264 Stitch Isg.jpeg]

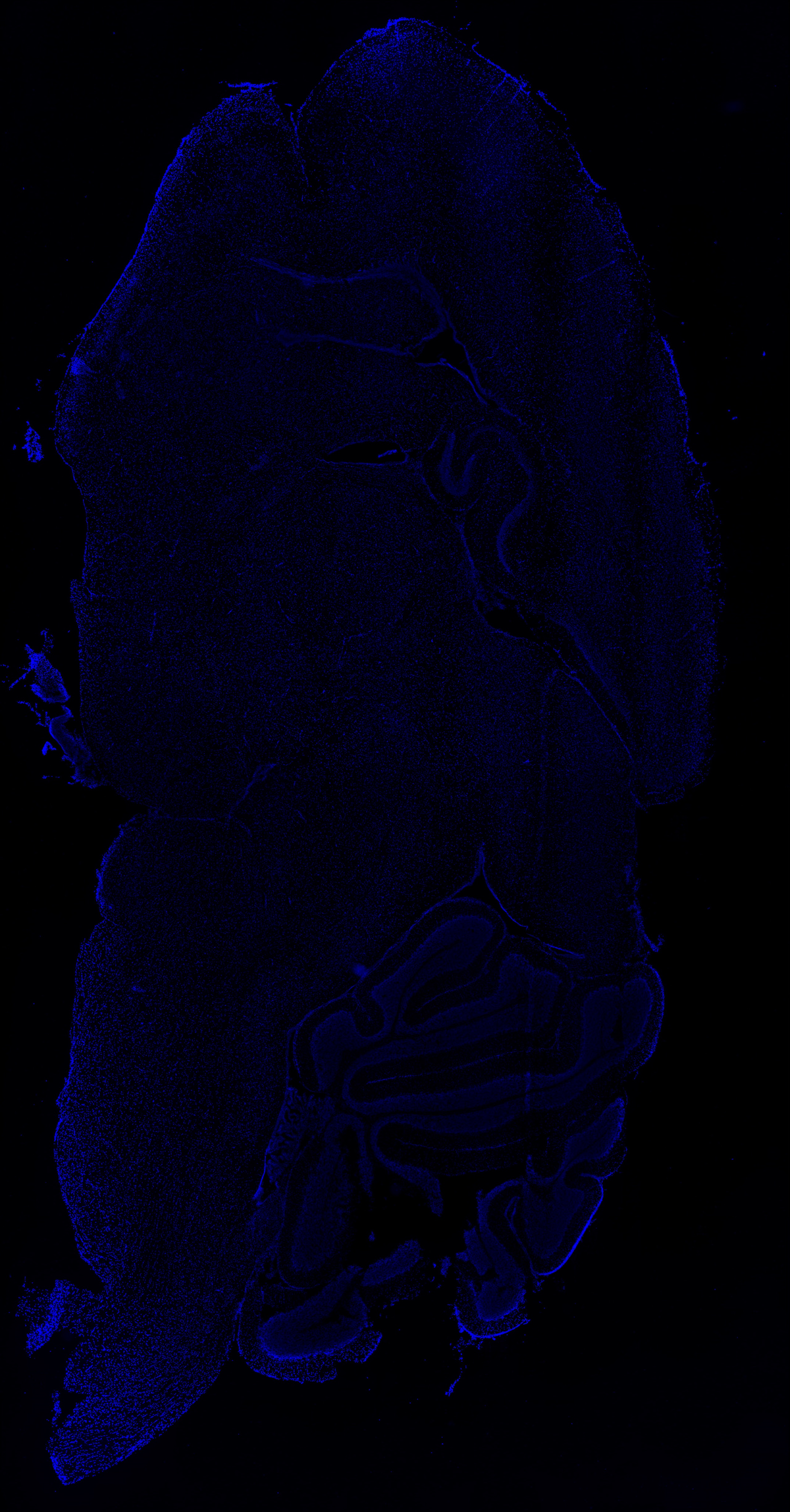

Supplement: Supplementary file 2. [file elife-102900-supp2.zip › Supplementary File 2/Raw Stitches/745.3 Stitch DAPI.jpeg]

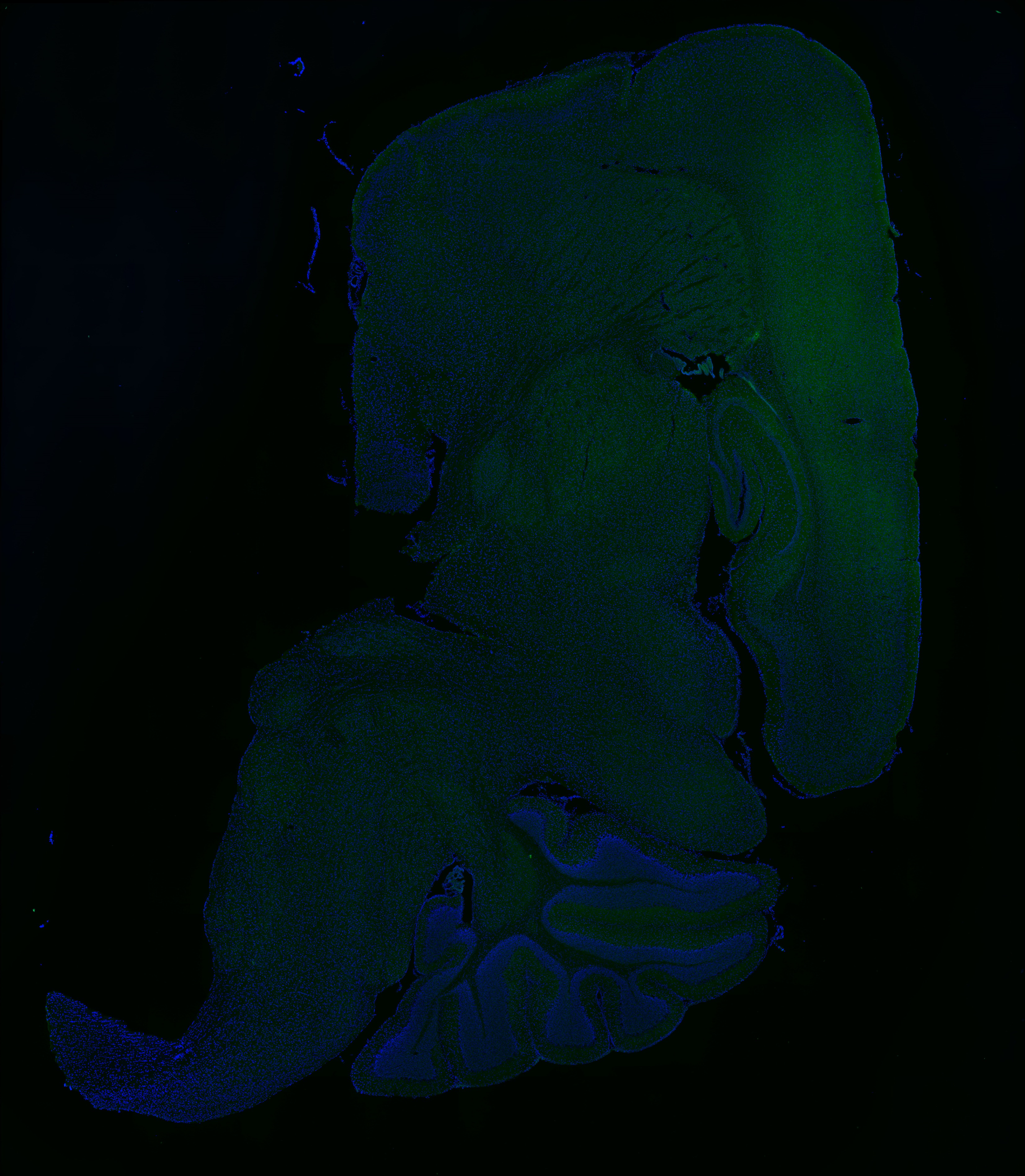

Supplement: Supplementary file 2. [file elife-102900-supp2.zip › Supplementary File 2/Raw Stitches/946 Stitch Overlay.jpeg]

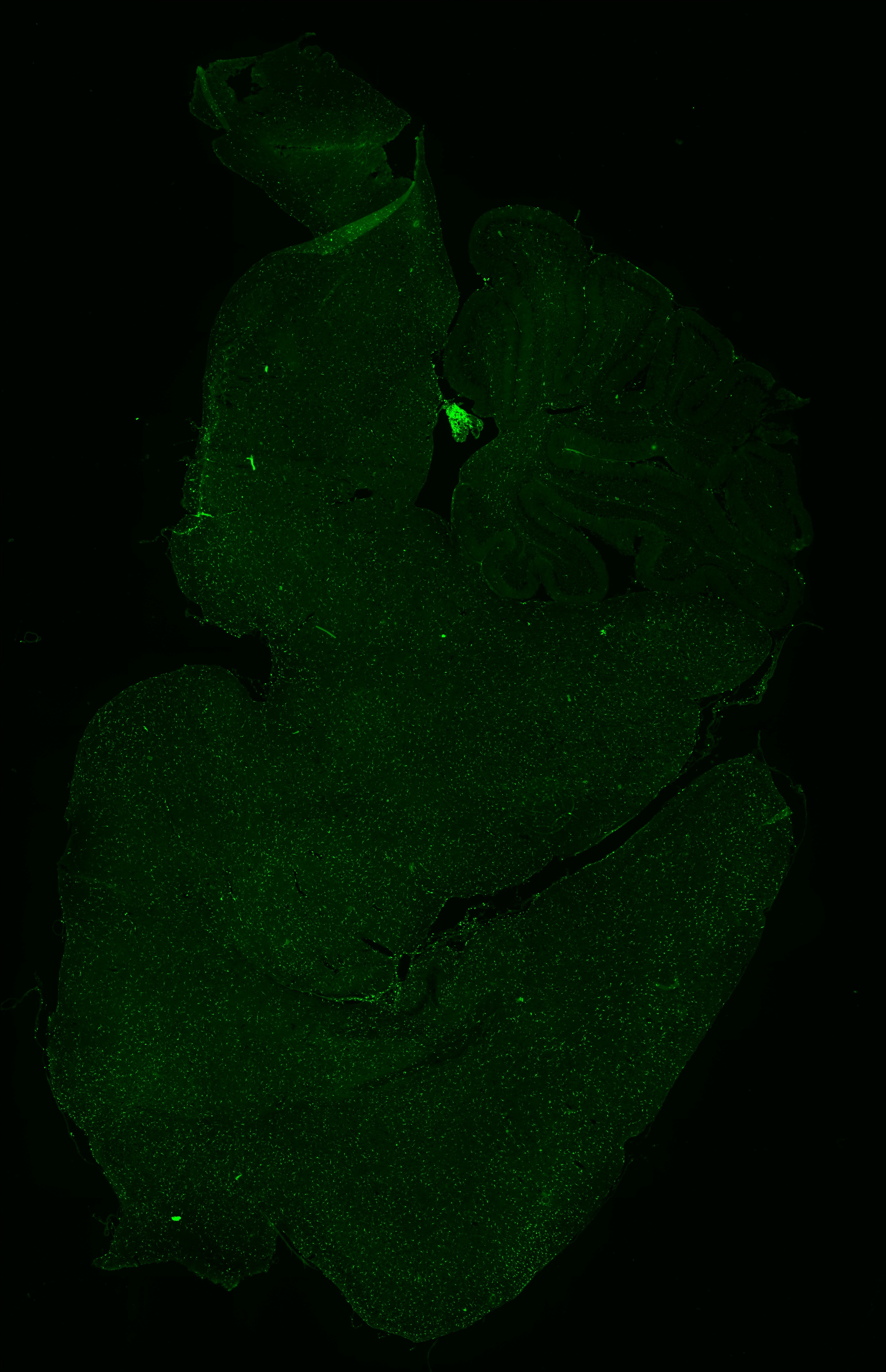

Supplement: Supplementary file 2. [file elife-102900-supp2.zip › Supplementary File 2/Raw Stitches/XY03 828 Stitch GFP.jpeg]

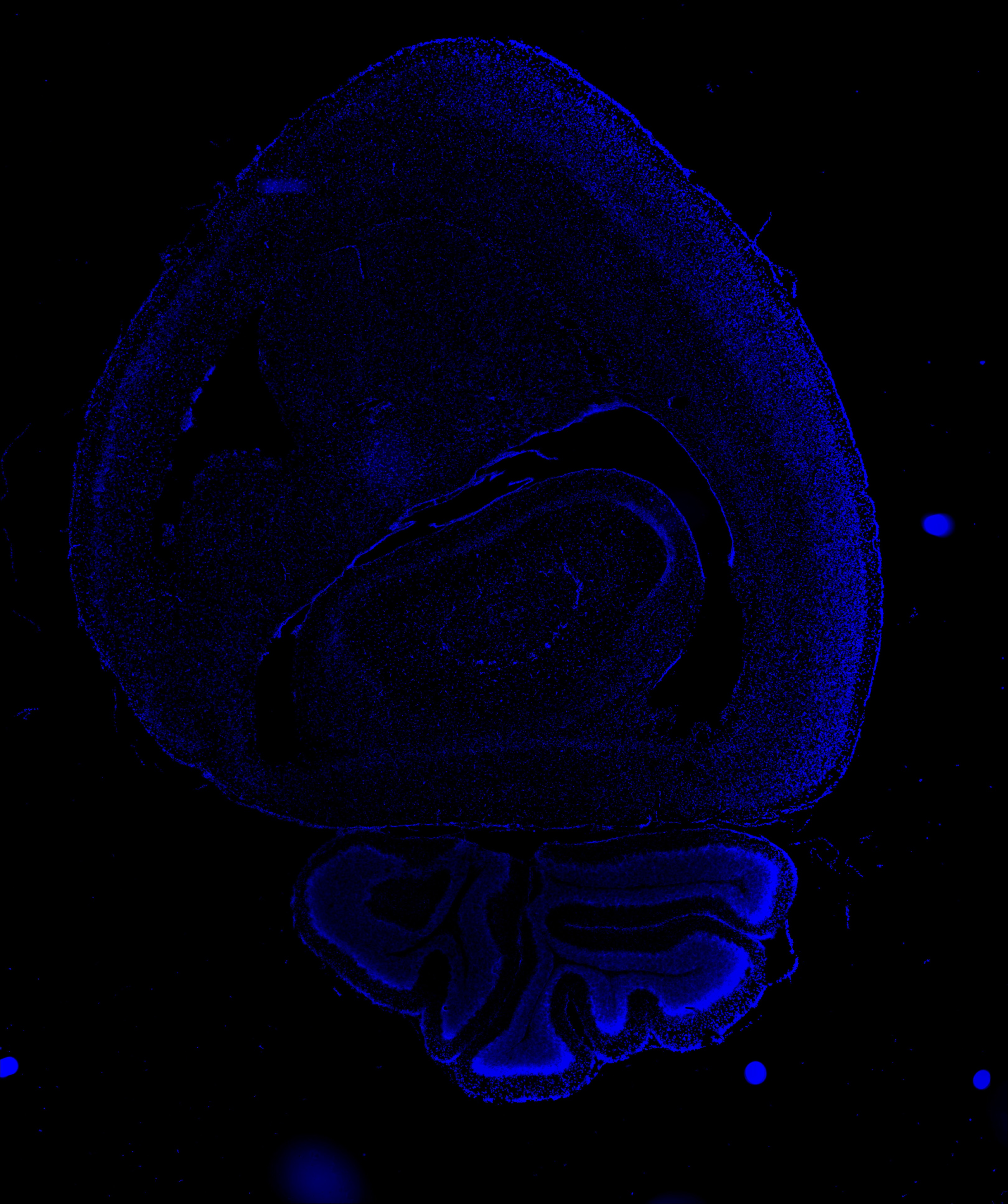

Supplement: Supplementary file 2. [file elife-102900-supp2.zip › Supplementary File 2/Raw Stitches/819 Stitch DAPI 2.jpeg]

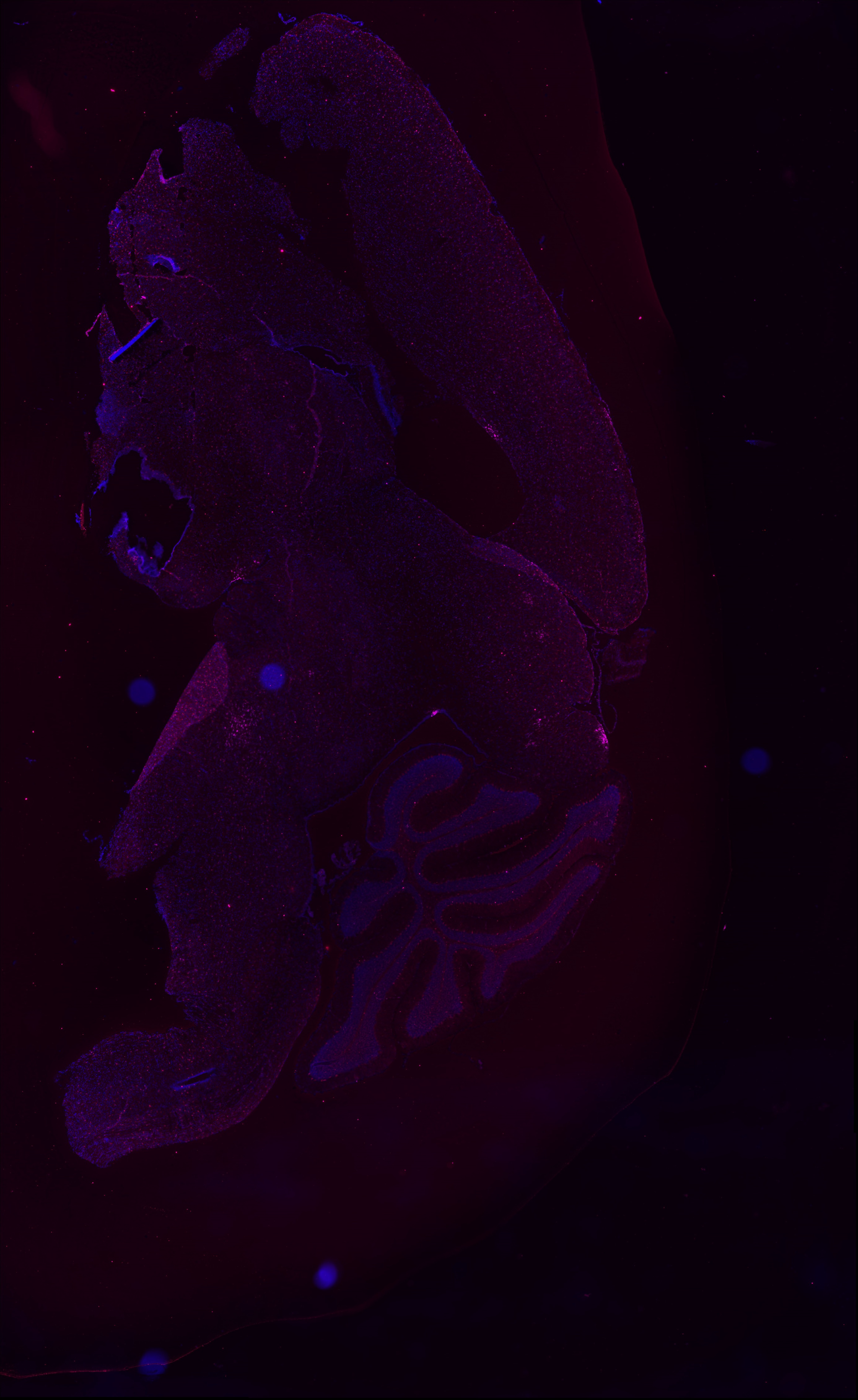

Supplement: Supplementary file 2. [file elife-102900-supp2.zip › Supplementary File 2/Raw Stitches/1152 Tam Sham 14dpi 4x Stitch Overlay.jpeg]

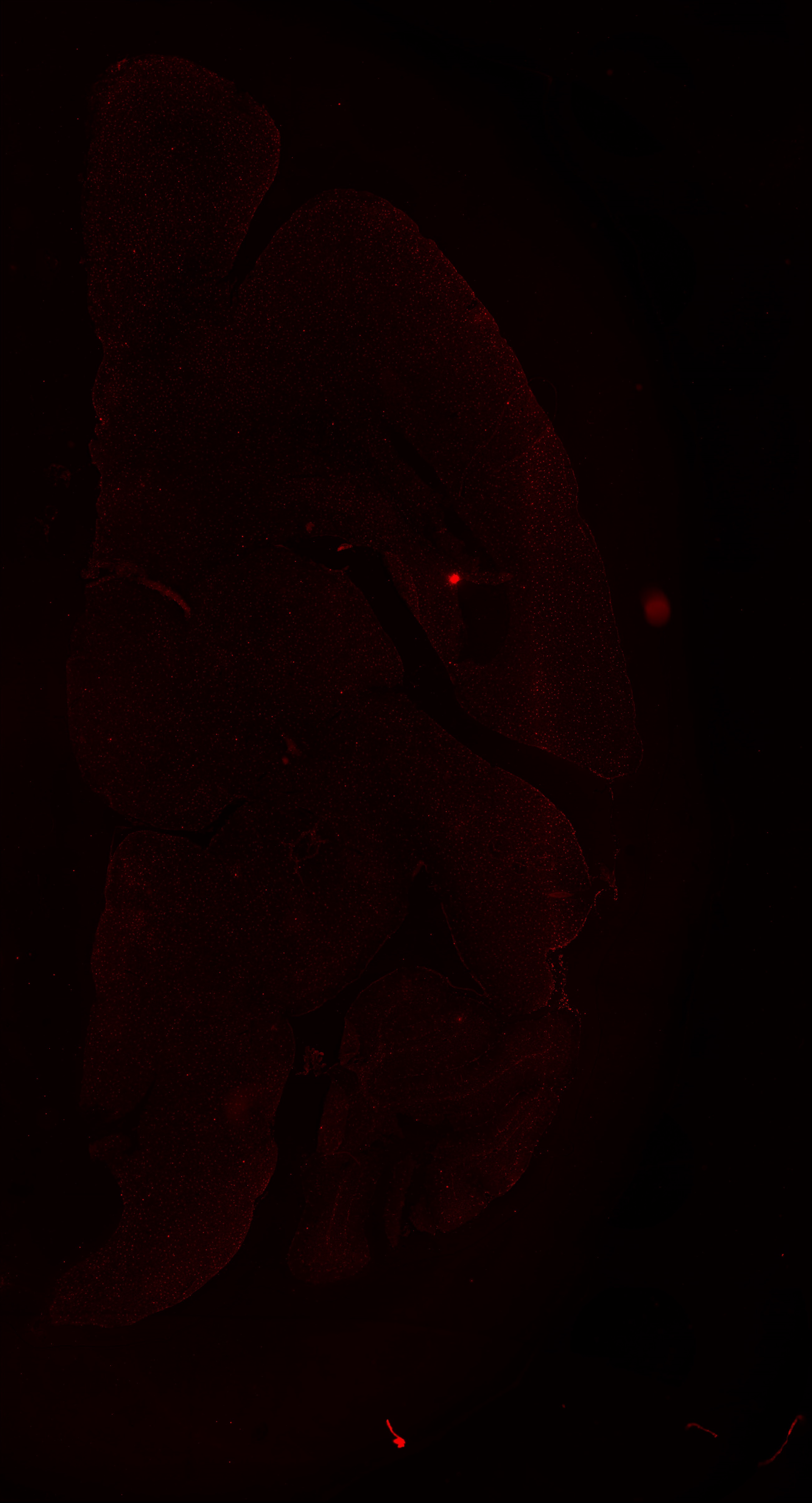

Supplement: Supplementary file 2. [file elife-102900-supp2.zip › Supplementary File 2/Raw Stitches/1180 Full D1113H 14d 4x Stitch Iba.jpeg]

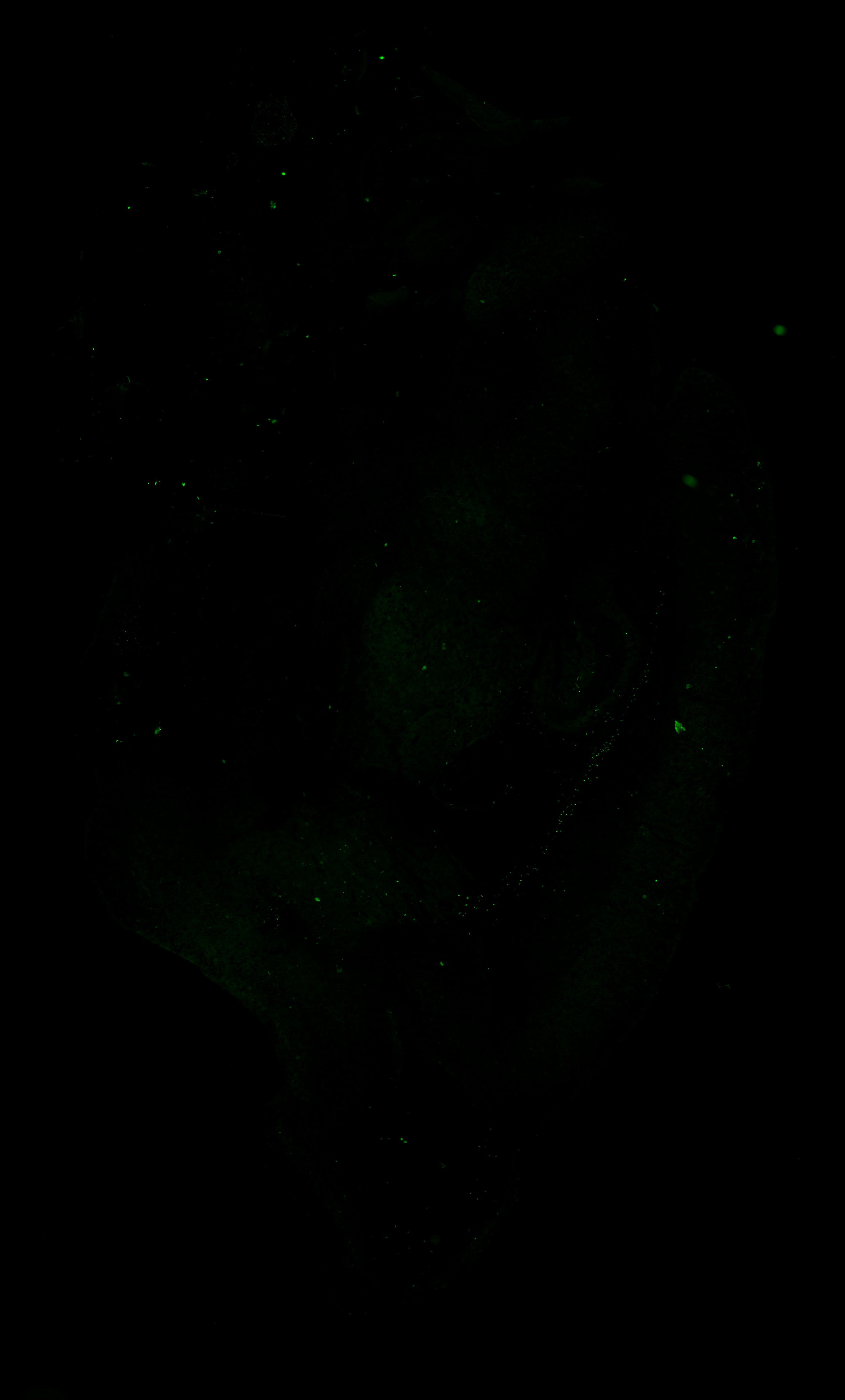

Supplement: Supplementary file 2. [file elife-102900-supp2.zip › Supplementary File 2/Raw Stitches/1224 Stitch GFP.jpeg]

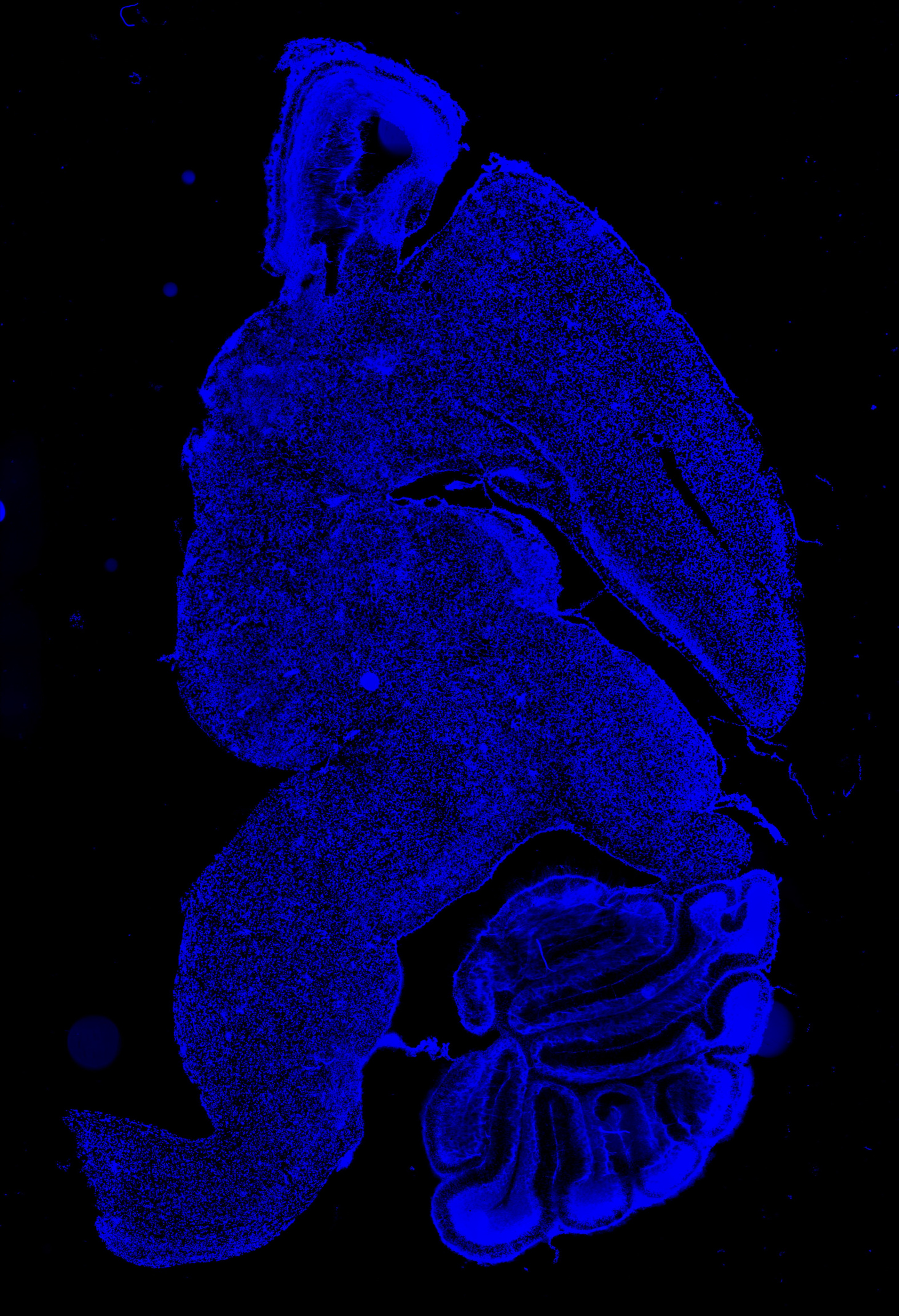

Supplement: Supplementary file 2. [file elife-102900-supp2.zip › Supplementary File 2/Raw Stitches/1264 Stitch DAPI 2.jpeg]

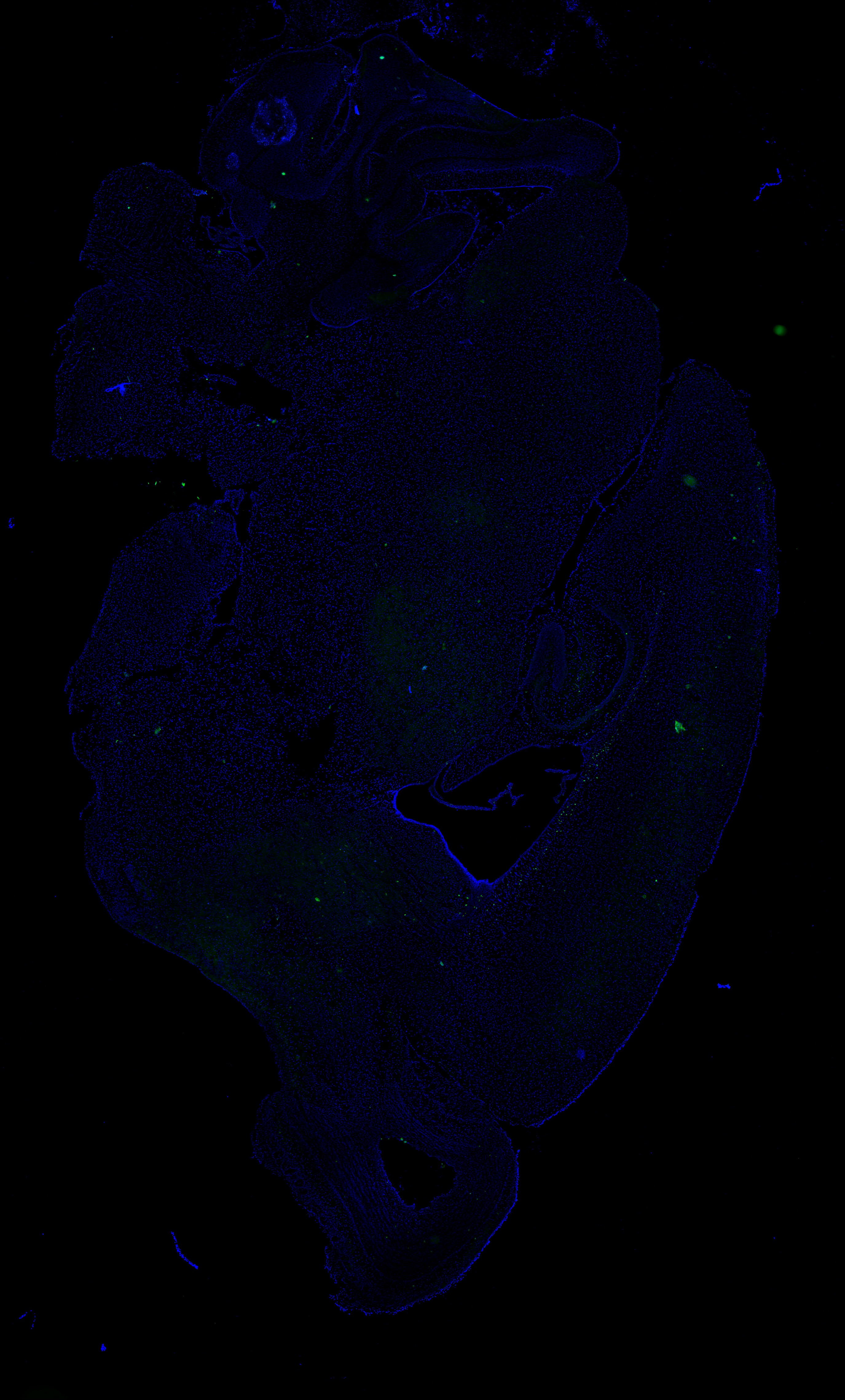

Supplement: Supplementary file 2. [file elife-102900-supp2.zip › Supplementary File 2/Raw Stitches/1224 Stitch Overlay.jpeg]

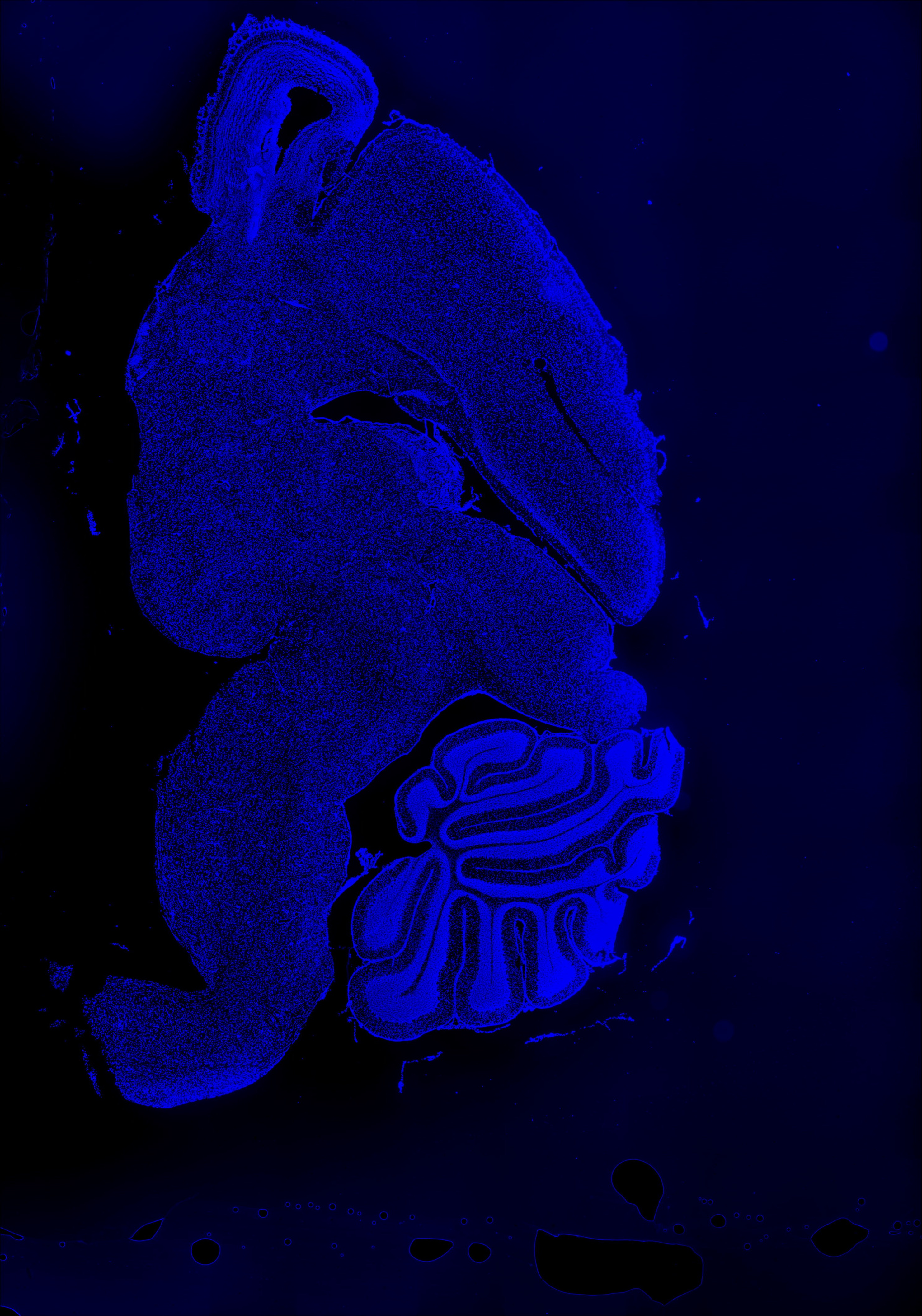

Supplement: Supplementary file 2. [file elife-102900-supp2.zip › Supplementary File 2/Raw Stitches/1264 Stitch DAPI.jpeg]

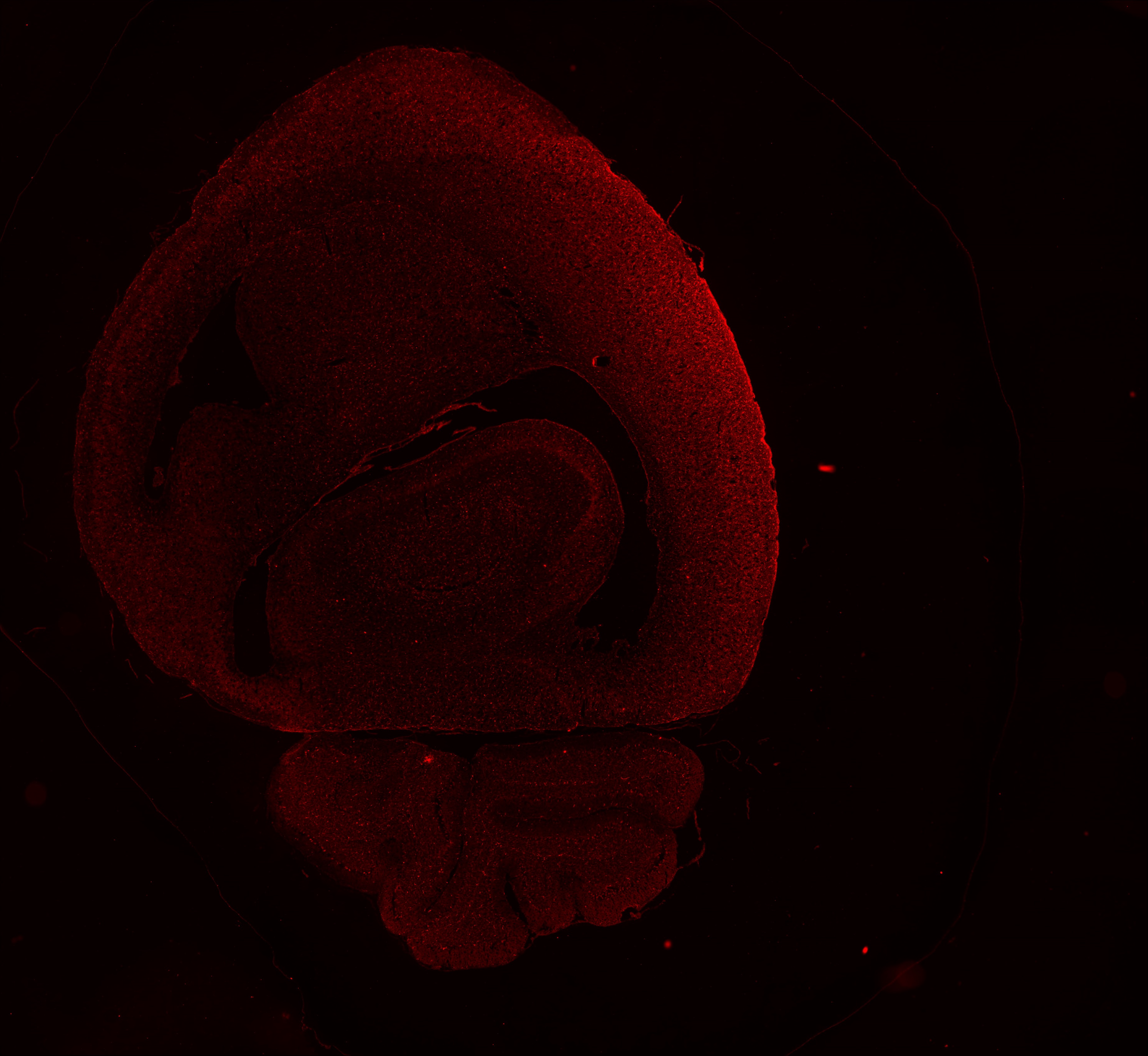

Supplement: Supplementary file 2. [file elife-102900-supp2.zip › Supplementary File 2/Raw Stitches/819 Stitch Iba.jpeg]

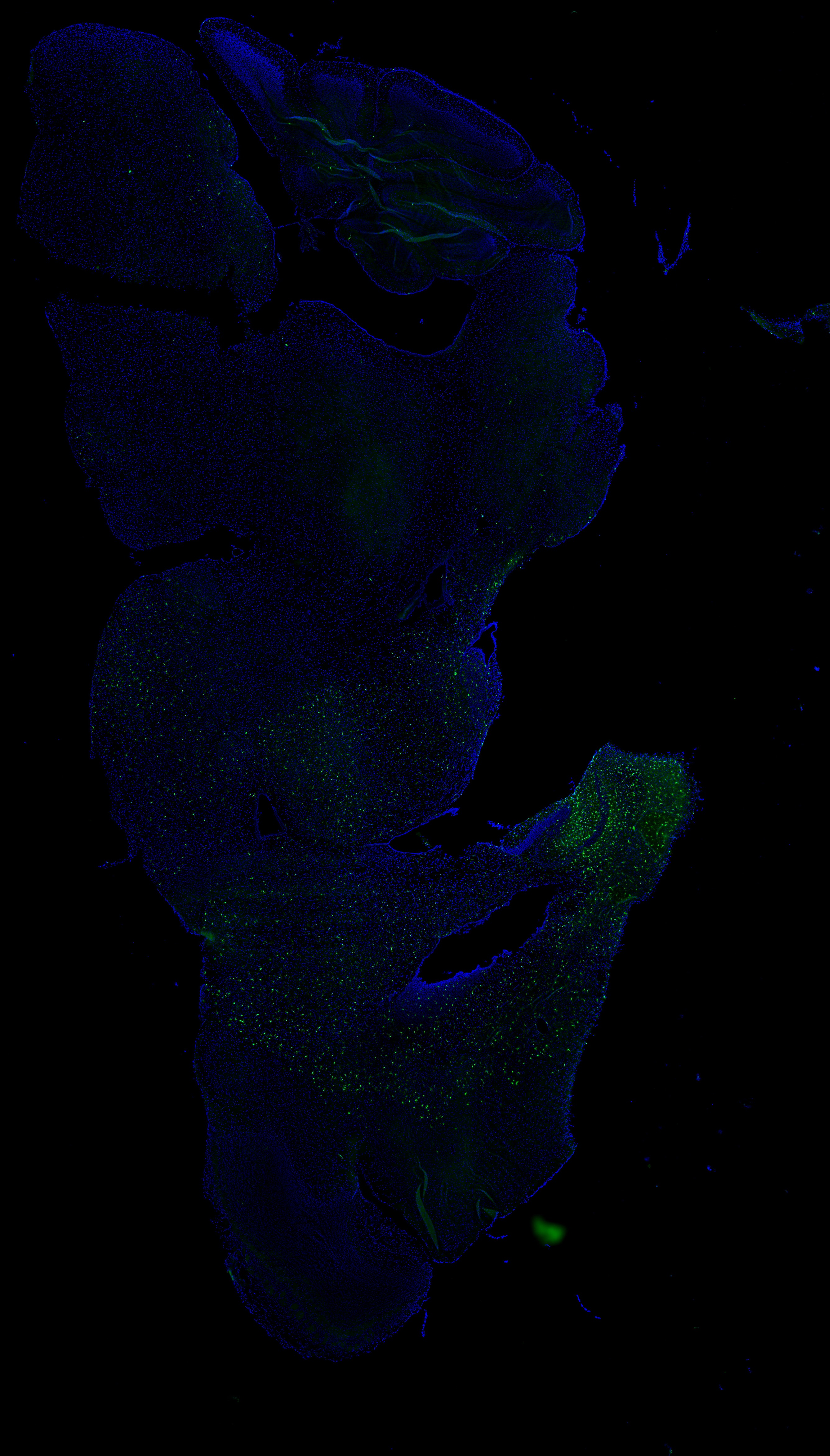

Supplement: Supplementary file 2. [file elife-102900-supp2.zip › Supplementary File 2/Raw Stitches/1071 Stitch Overlay.jpeg]

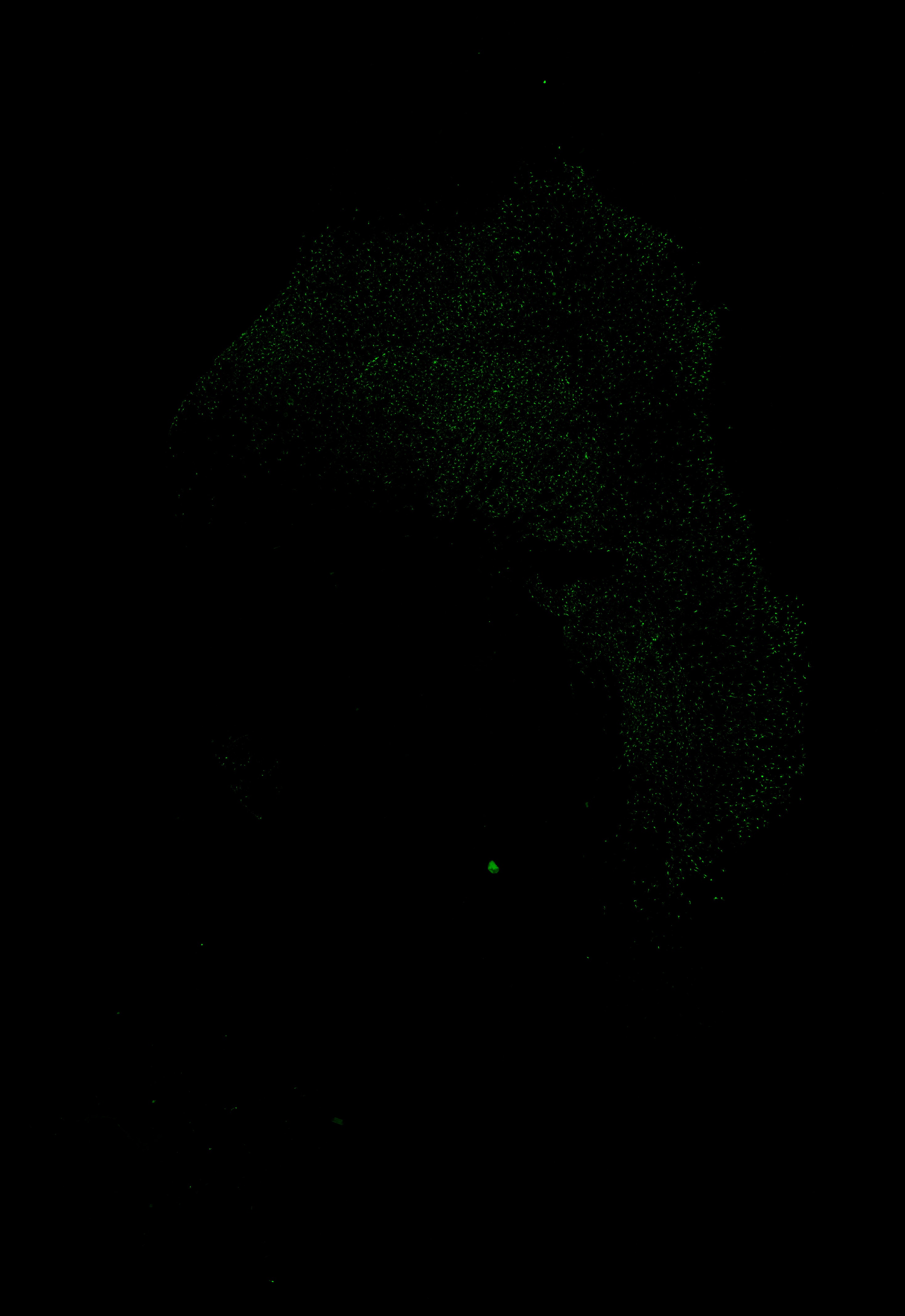

Supplement: Supplementary file 2. [file elife-102900-supp2.zip › Supplementary File 2/Raw Stitches/1076 Stitch GFP.jpeg]

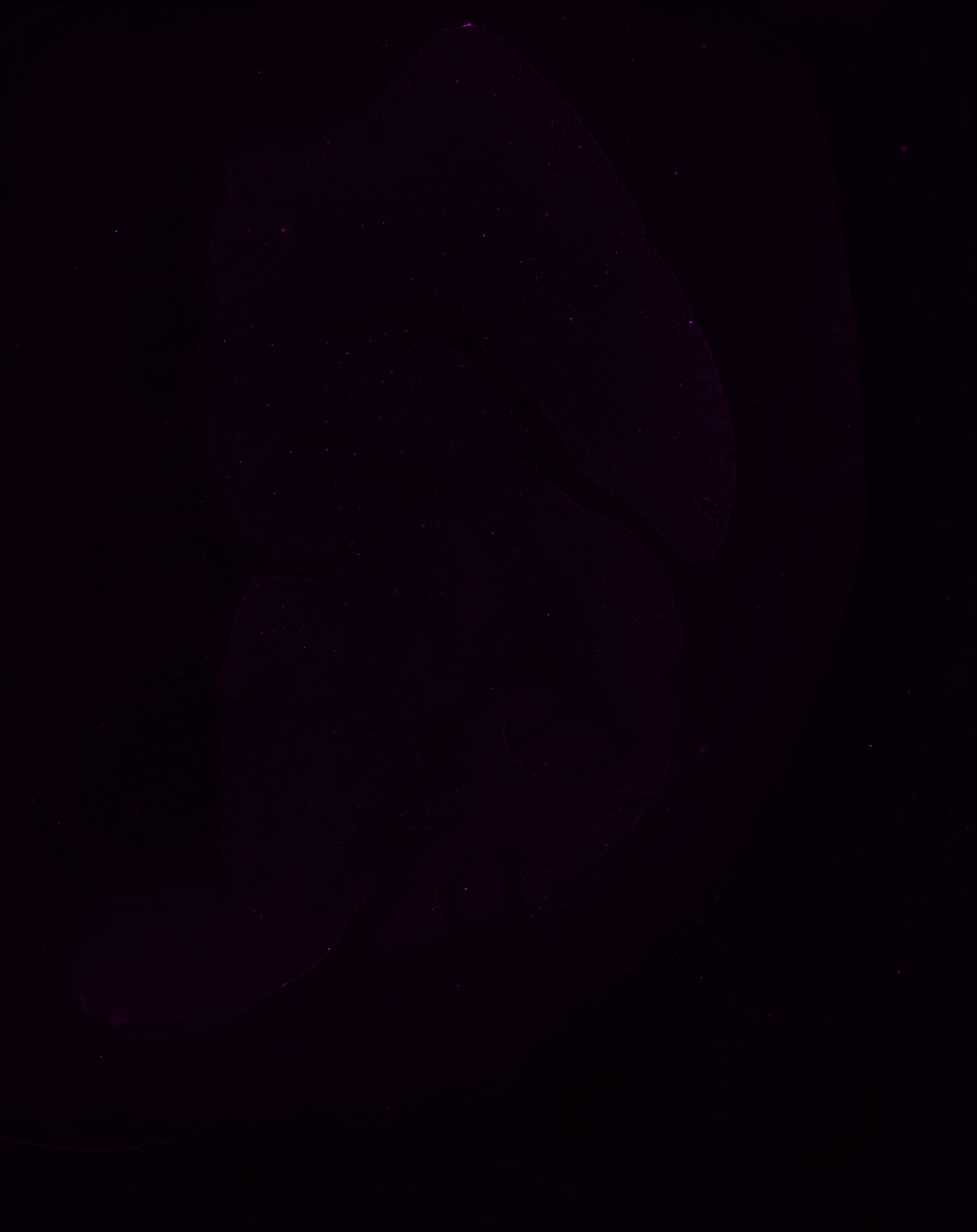

Supplement: Supplementary file 2. [file elife-102900-supp2.zip › Supplementary File 2/Raw Stitches/1144 ICT WT 13dpi 4x Stitch Isg.jpeg]

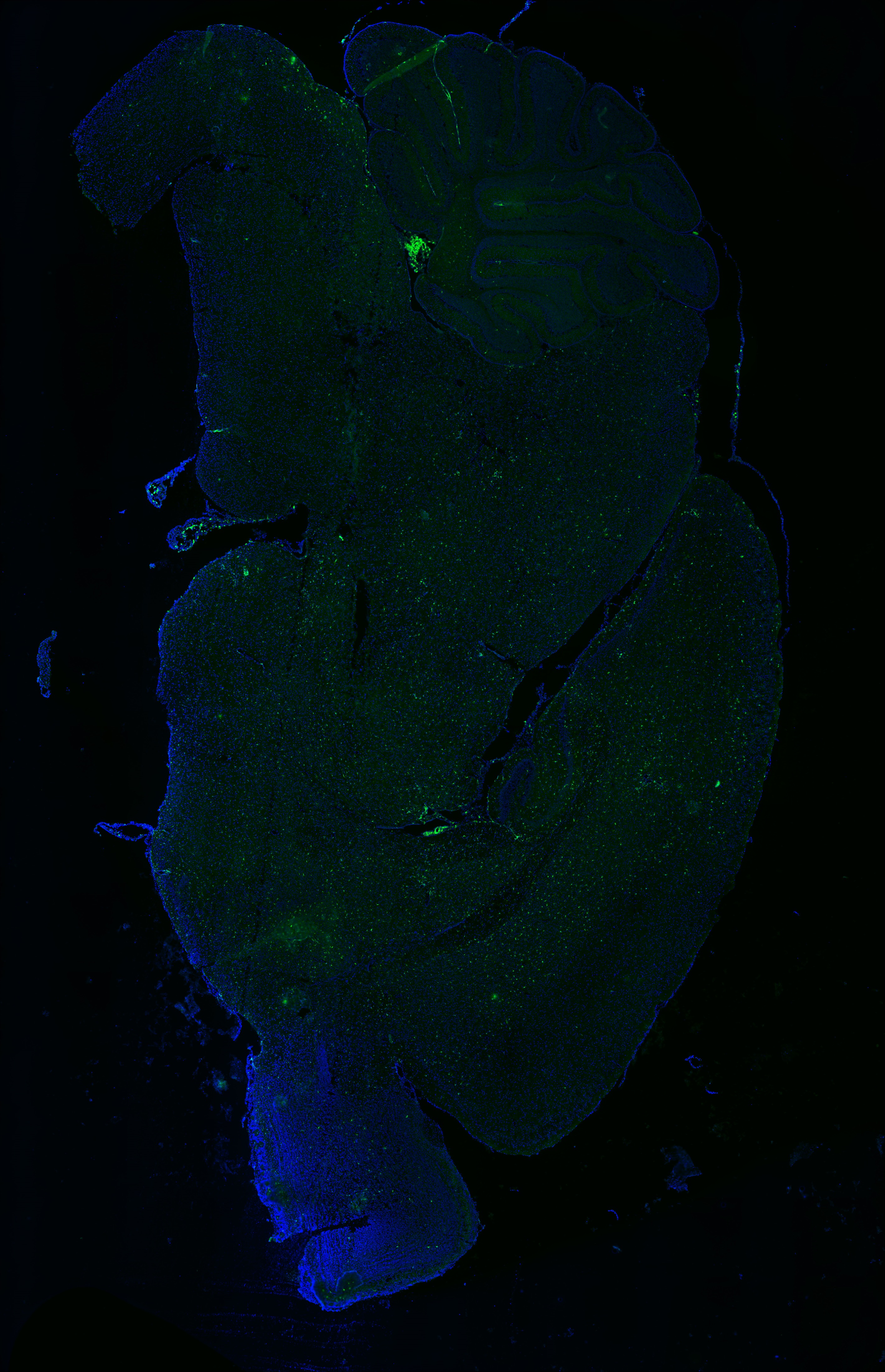

Supplement: Supplementary file 2. [file elife-102900-supp2.zip › Supplementary File 2/Raw Stitches/1083 Stitch Overlay.jpeg]

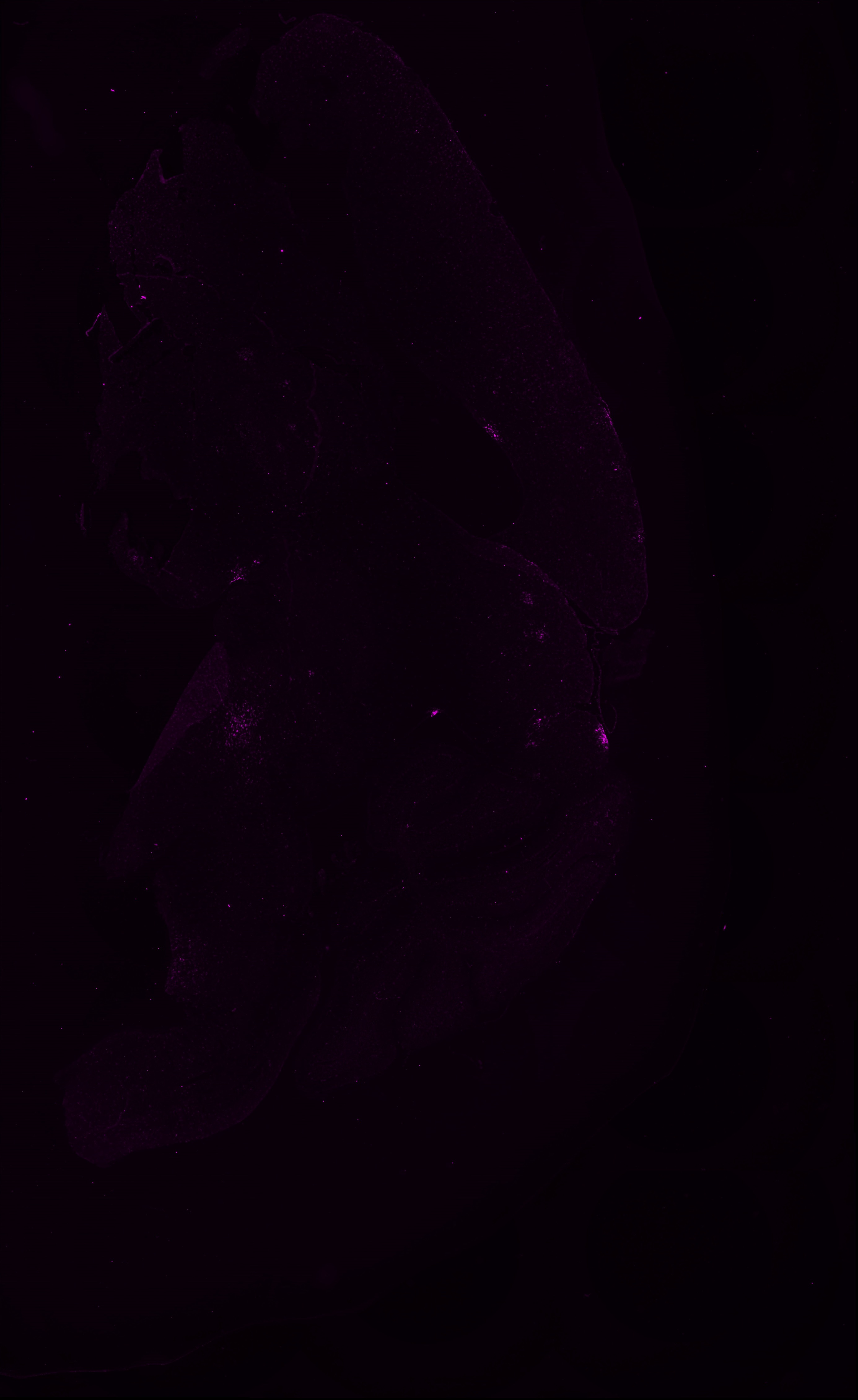

Supplement: Supplementary file 2. [file elife-102900-supp2.zip › Supplementary File 2/Raw Stitches/1152 Tam Sham 14dpi 4x Stitch Isg.jpeg]

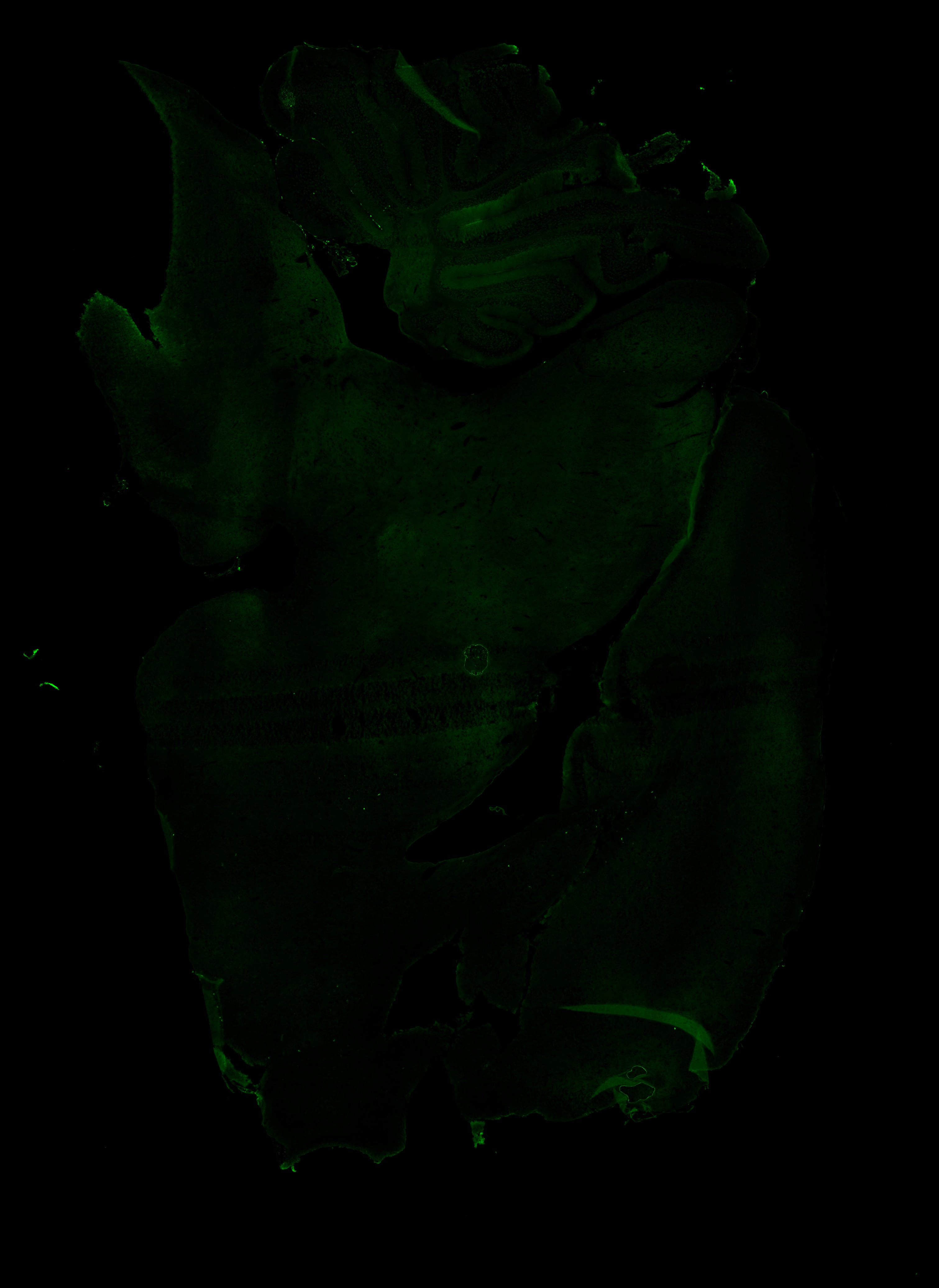

Supplement: Supplementary file 2. [file elife-102900-supp2.zip › Supplementary File 2/Raw Stitches/1237 Stitch GFP.jpeg]

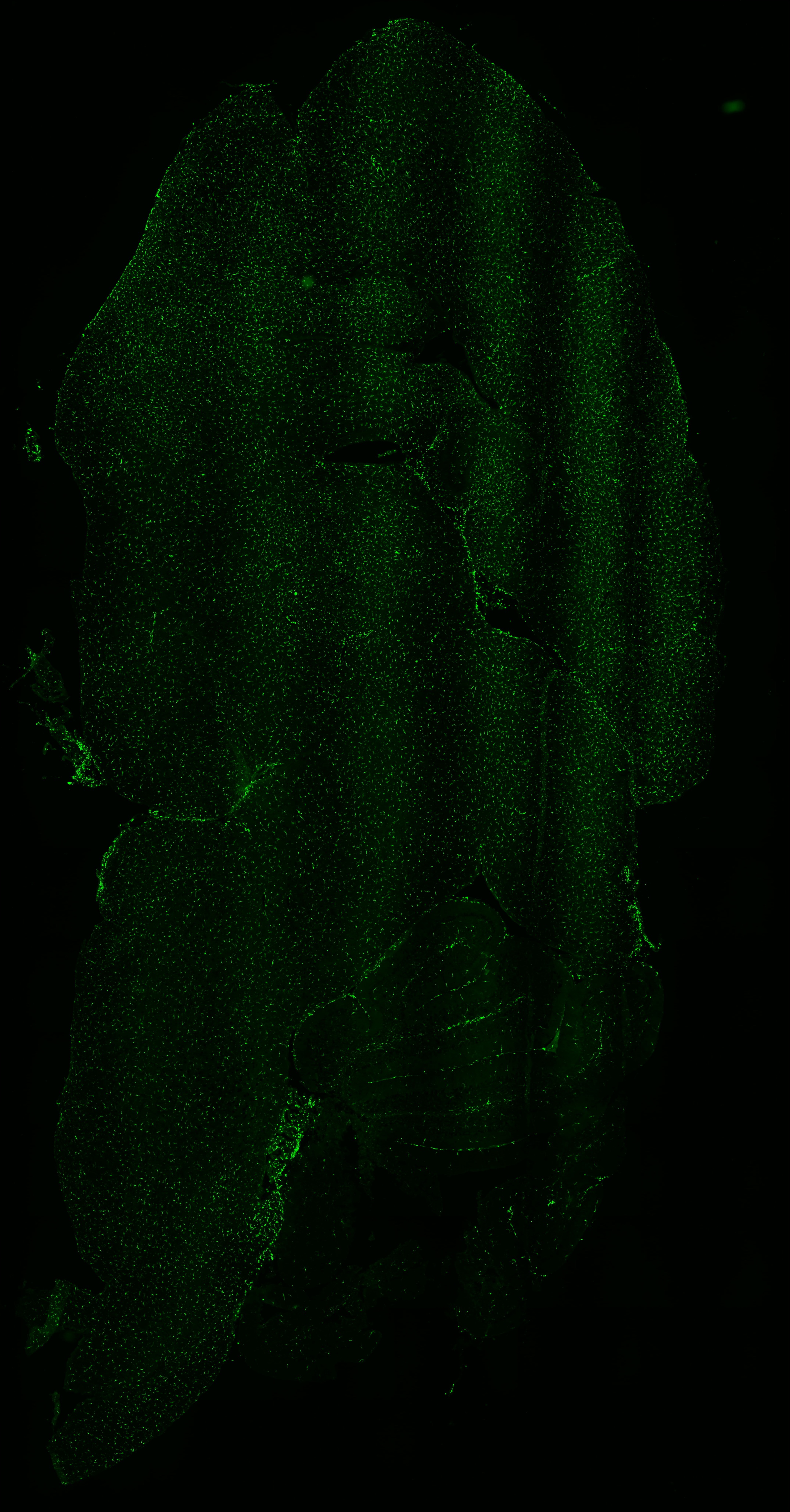

Supplement: Supplementary file 2. [file elife-102900-supp2.zip › Supplementary File 2/Raw Stitches/745.3 Stitch GFP.jpeg]

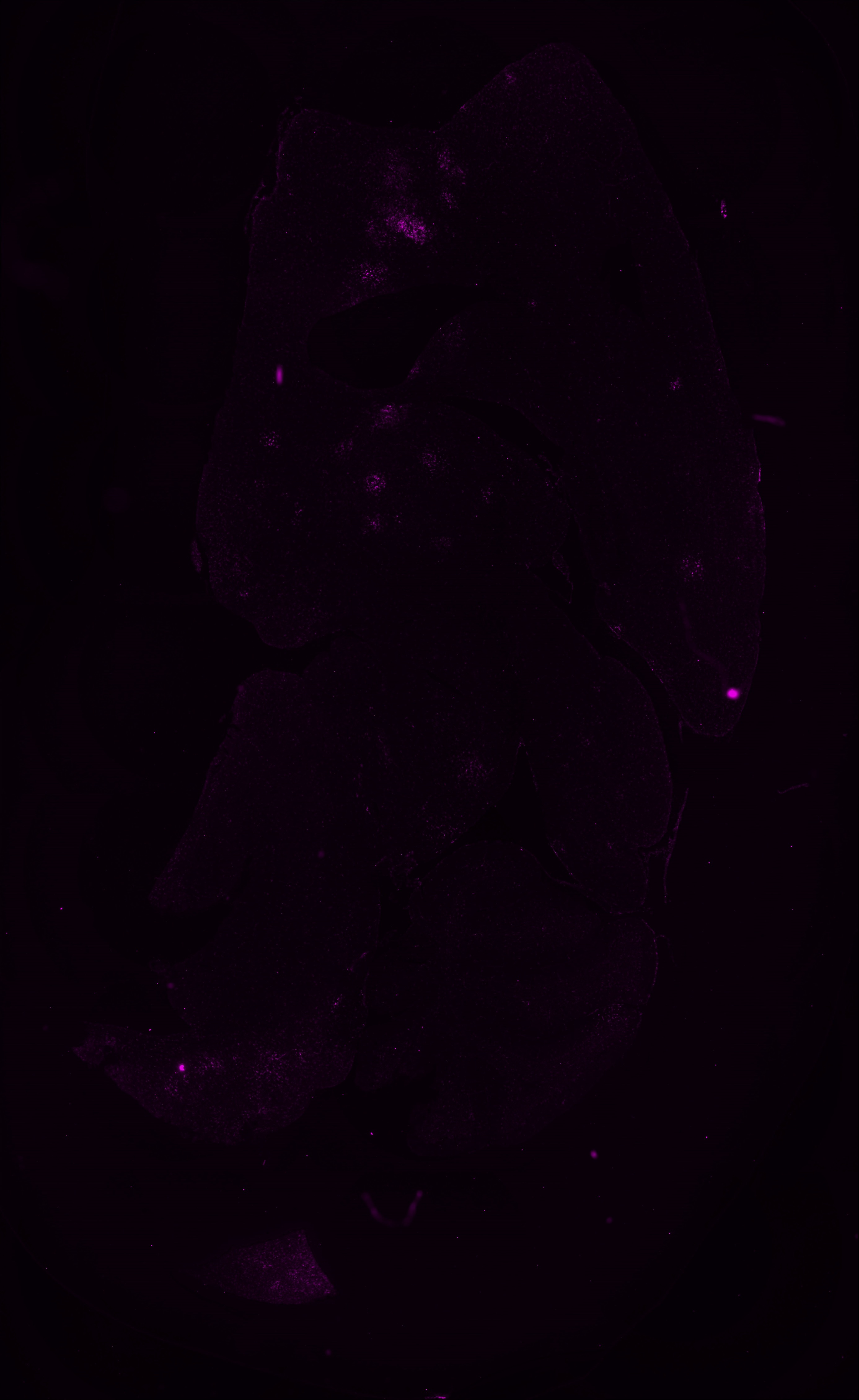

Supplement: Supplementary file 2. [file elife-102900-supp2.zip › Supplementary File 2/Raw Stitches/1186 ICT D1113H 27dpi 4x Stitch Isg.jpeg]

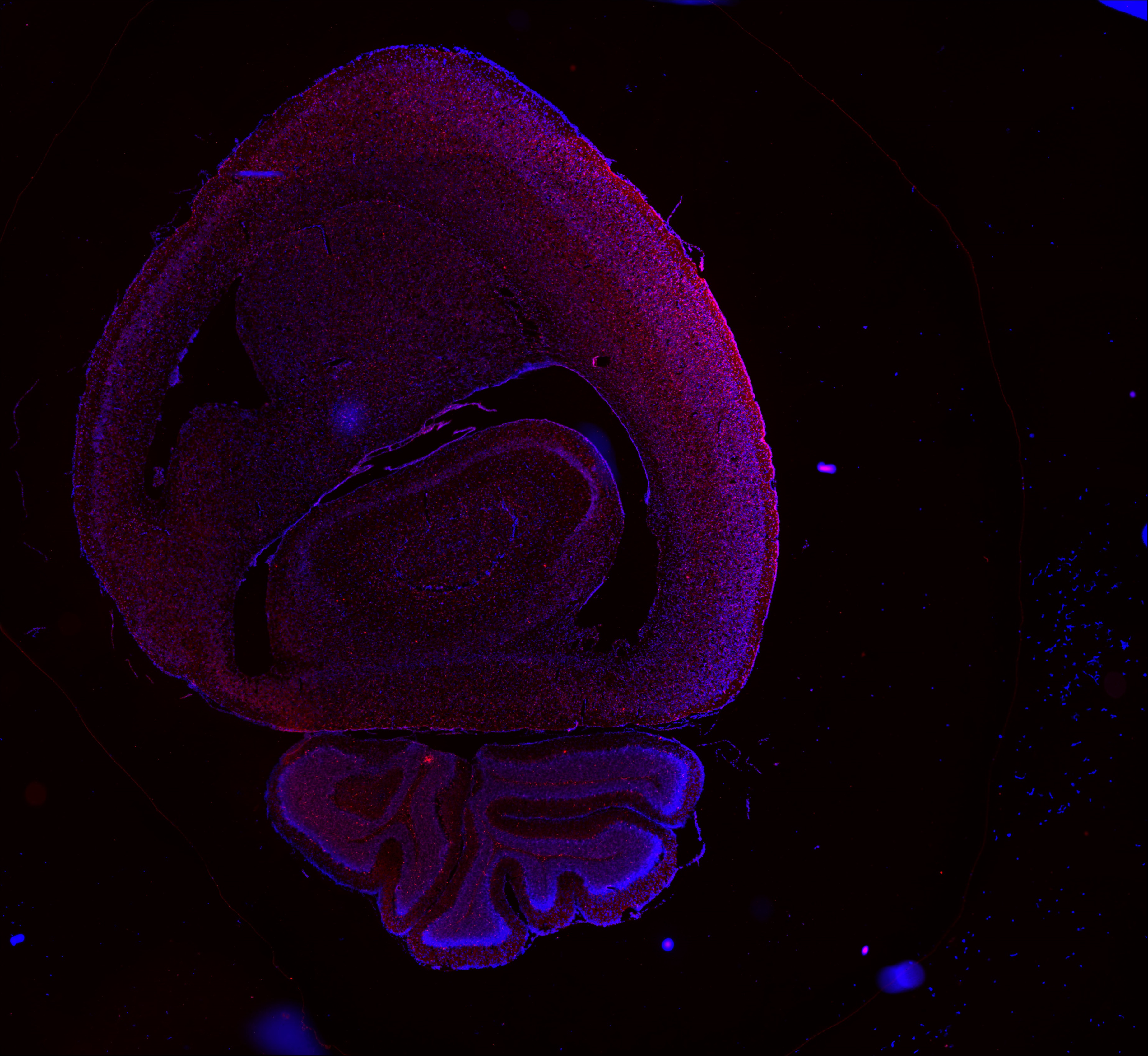

Supplement: Supplementary file 2. [file elife-102900-supp2.zip › Supplementary File 2/Raw Stitches/819 Stitch Overlay.jpeg]

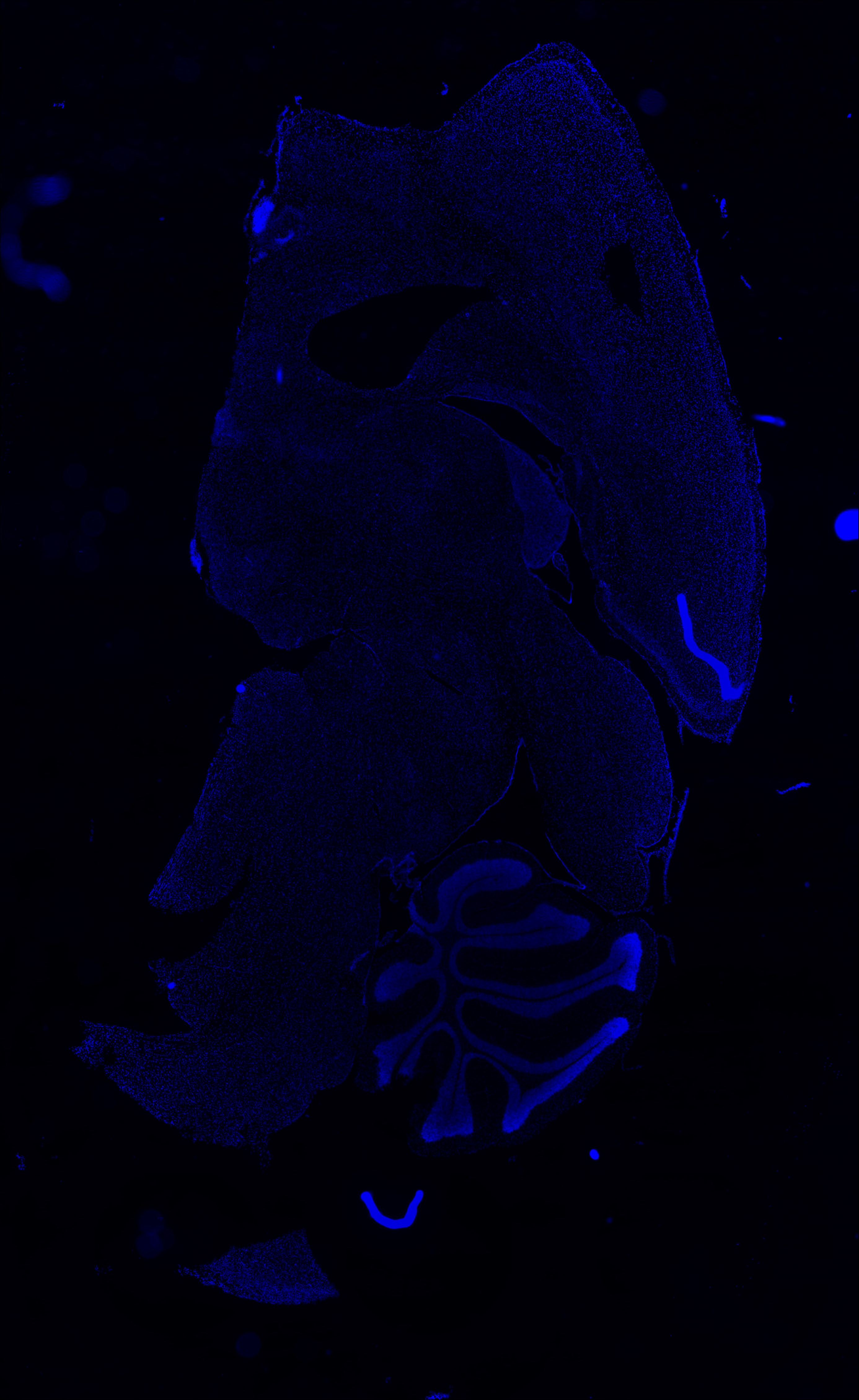

Supplement: Supplementary file 2. [file elife-102900-supp2.zip › Supplementary File 2/Raw Stitches/1186 ICT D1113H 27dpi 4x Stitch DAPI.jpeg]

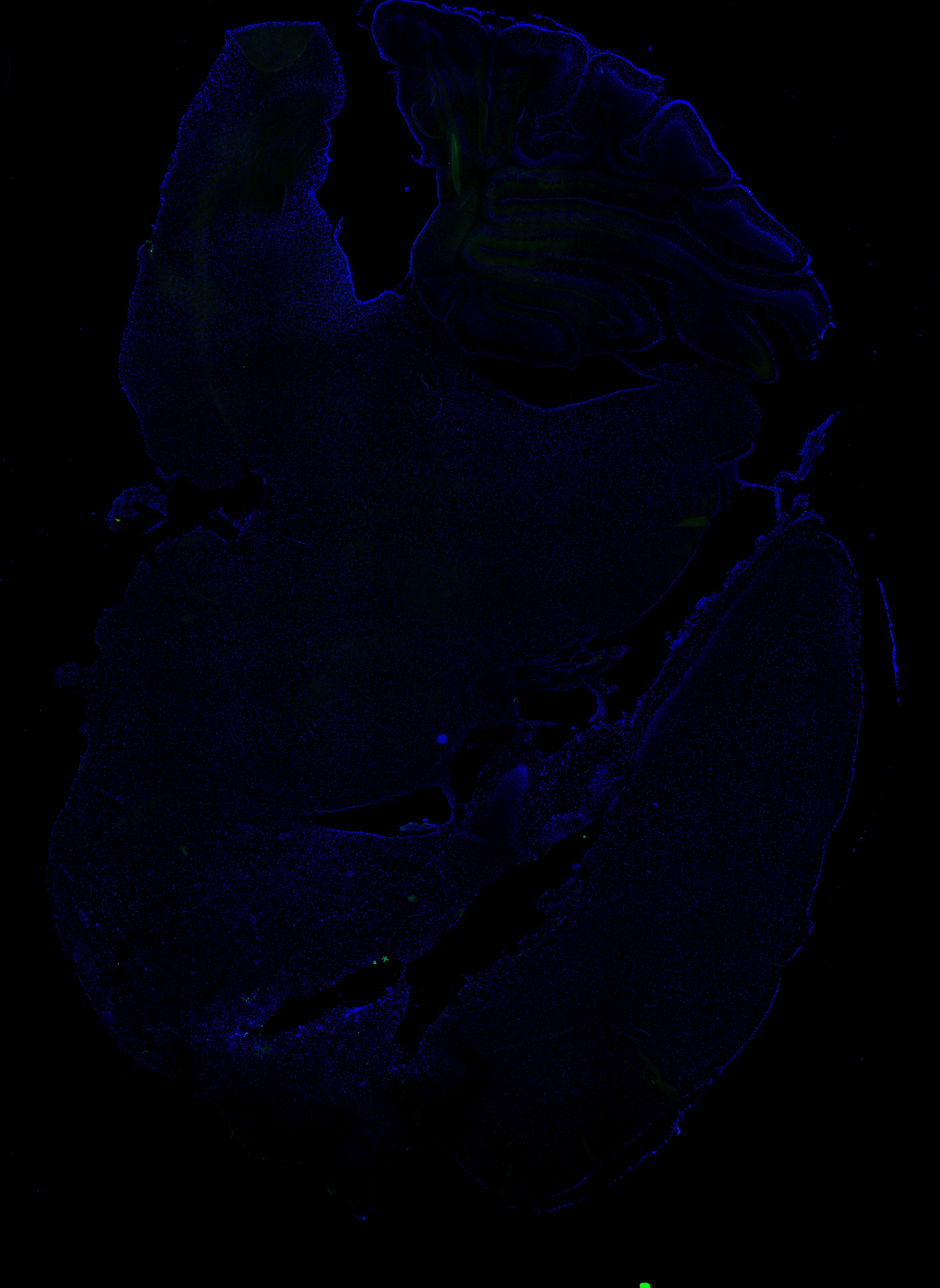

Supplement: Supplementary file 2. [file elife-102900-supp2.zip › Supplementary File 2/Raw Stitches/1239 Stitch Overlay.jpeg]
